# Si–O Bond Formation Mediated by an Osmium-Polyhydride: Dehydrogenative Silylation of Alcohols and Tandem Hydrosilylation/Dehydrogenative Silylation of Salicylaldehydes

**DOI:** 10.1021/acs.inorgchem.6c01007

**Published:** 2026-05-05

**Authors:** Iñigo V. Alli, Enrique Oñate, Montserrat Oliván

**Affiliations:** Departamento de Química Inorgánica - Instituto de Síntesis Química y Catálisis Homogénea (ISQCH) - Centro de Innovación en Química Avanzada (ORFEO−CINQA), 16765 Universidad de Zaragoza - CSIC, Zaragoza 50009, Spain

## Abstract

OsH_5_(SiHPh_2_)­(P^i^Pr_3_)_2_ (**1**) catalyzes the monoalcoholysis of diphenylsilane with a variety of alcohols. Density functional theory (DFT) calculations suggest that the reactions occur via a highly ordered transition state resulting from the nucleophilic attack of the alcohol to the silane coordinated to the osmium center in an η^1^-H–SiHPh_2_ fashion. The alcoholysis or aminolysis of the Si–H bond of **1** with 2-hydroxypyridine or 2-aminopyridine affords OsH_3_{κ^2^-*Si,N*-(SiPh_2_-E-py)}­(P^i^Pr_3_)_2_ (E = O (**4**), NH (**5**)). Analogously, OsH_4_(SiH_2_Ph)_2_(P^i^Pr_3_)_2_ (**2**) reacts with 2-hydroxypyridine and 2-aminopyridine to give OsH_3_{κ^2^-*Si,N*-(SiPh­(Epy)-E-py)}­(P^i^Pr_3_)_2_ (E = O (**6**), NH (**7**)), as a result of the alcoholysis or aminolysis, respectively, of both Si–H bonds of one of the phenylsilyl ligands. Additionally, **1** catalyzes the tandem hydrosilylation/dehydrogenative silylation of salicylaldehydes with diphenylsilane to afford silacycles. DFT calculations suggest that this process happens via an outer-sphere hydrogenation of the aldehyde moiety to give a diol and tetrahydride-silylene OsH_4_(=SiPh_2_)­(P^i^Pr_3_)_2_. Next, the silylative dehydrogenation of the Ph–OH function affords a silyl-O-functionalized pentahydride, which, upon the nucleophilic intramolecular attack of the benzylic OH group, gives the silacycle and intermediate OsH_4_(η^2^-H_2_)­(P^i^Pr_3_)_2_, which reacts with diphenylsilane, giving H_2_ and regenerating OsH_5_(SiHPh_2_)­(P^i^Pr_3_)_2_.

## Graphical abstract

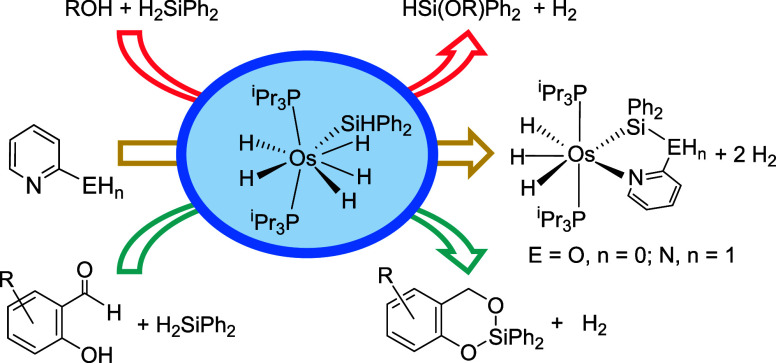

## Introduction

The dehydrogenative silylation of alcohols with hydrosilanes,^1^ in addition to the hydrosilylation of carbonyl compounds with hydrosilanes,^2^ constitutes one of the most powerful tools for the formation of silyl ethers, that have wide application as protective groups in organic synthesis^3^ and for the production of silicon-based polymeric materials in industry.^4^ Furthermore, this reaction generates hydrogen as a byproduct, which has also led to an increasing interest in the use of hydrosilanes as hydrogen storage materials.^5^ Although their hydrogen storage capacity (i.e., their weight percentage of hydrogen) might be considered relatively low, hydrosilanes stand out because they are usually easy to handle and store and, unlike other chemical hydrogen carriers, their recyclability is possible.^6^

The alcoholysis of the Si–H bonds of hydrosilanes is a thermodynamically favored but kinetically slow process, requiring the use of a catalyst for an effective release of molecular hydrogen. Although the reaction had been studied with various transition metal complexes as catalysts,^1a,7^ it was the work of Luo and Crabtree the one that marked a turning point in the field.^8^ In this seminal contribution these authors reported that cationic dihydride [IrH_2_(MeOH)_2_(PPh_3_)_2_]^+^ is an extremely active catalyst for the methanolysis of triethylsilane through a mechanism that involves the outer-sphere nucleophilic attack of the alcohol to the silicon atom of a coordinated silane. The attack gives rise to a trihydride intermediate and a silylium cation stabilized by the alcohol, that react to afford an iridium-dihydride-dihydrogen complex and the silyl ether. The displacement of the coordinated molecular hydrogen by a new silane molecule would close the cycle (Scheme 1) for further alcoholysis. Since then, it has been demonstrated that a plethora of complexes, mostly bearing a cationic metal center^9^ but also neutral species,^10^ catalyze this reaction, and that of hydrolysis of silanes, via a related mechanism. In further theoretical studies carried out with the system [Fe­(Cp)­(CO)­(PR_3_)]^+^ it was shown that the conversion of the σ-silane derivative into the dihydrogen complex could occur in a single step, avoiding the hydride intermediate.^11^ Furthermore, the alcoholysis of Si–H bonds in silyl complexes bearing hydrogen substituents on the silicon atom is a useful tool to derivatize silicon while leaving the metal–silyl bond intact.^12^

**Scheme 1:**
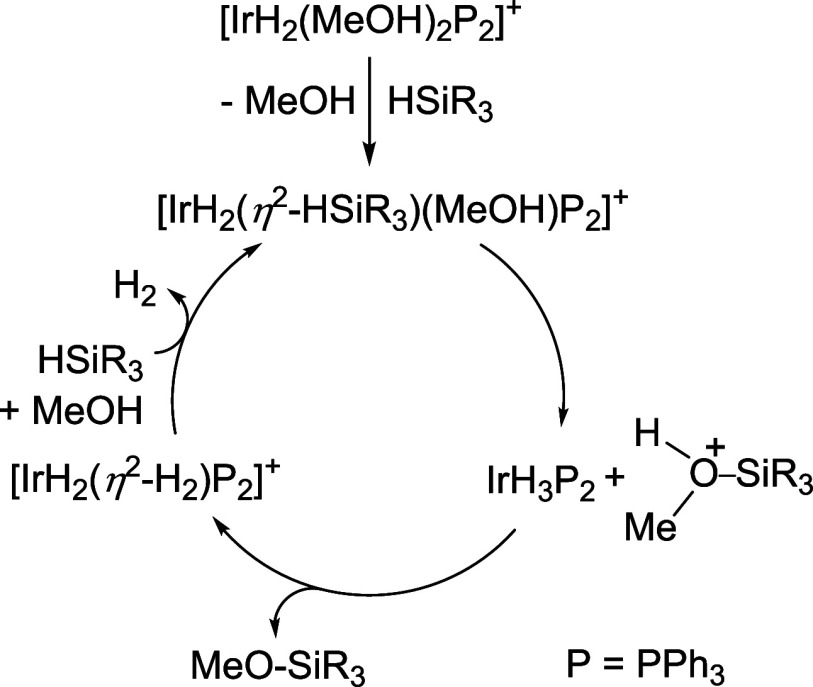
Mechanism for the Alcoholysis of Silanes Catalyzed by [IrH_2_(MeOH)_2_(PPh_3_)_2_]^+^

Tandem catalytic [*n* + 1] (*n* = 4, 5) annulative processes using dihydrosilanes are one of the synthetic strategies that can be employed for the synthesis of silacycles.^13^ In these reactions the dihydrosilane has two roles, allowing the sequential combination of two different processes to afford the silacycles. The majority of these tandem reactions require a catalyst for each reaction^14^ and the examples where a single catalyst plays a dual role are scarce. Thus, different rhodium systems catalyze tandem carbonyl hydrosilylation/olefin hydrosilylation of α,β-unsaturated aldehydes,^15^ dehydrogenative OH silylation/olefin hydrosilylation of allylic alcohols,^15,16^ dehydrogenative OH silylation/carbonyl hydrosilylation of α-ketoalcohols,^15,17^ and dehydrogenative OH silylation/dehydrogenative silylation of C­(sp^2^)-H bonds of benzylic alcohols^18^ to afford five- and six-membered oxasilacycles and imine hydrosilylation/dehydrogenative silylation of C­(sp^2^)-H bonds of *N*-phenyl benzaldimines that leads to azasilacycles (Scheme 2a).^18^ Furthermore, two iridium based systems have been used to prepare ring-fused oxasilacycles combining dehydrogenative OH silylation and C­(sp^2^)-H dehydrogenative silylation (Scheme 2b),^19^ while the Pt_2_(dba)_3_/RuPhos (RuPhos = 2-dicyclohexylphosphino-2’,6’-diisopropoxybiphenyl) system catalyzes the tandem dehydrogenative OH silylation/hydrosilylation of internal alkynes bearing an alcohol or silanol group with dihydrosilanes to afford six-membered silacycles (Scheme 2c).^20^

**Scheme 2:**
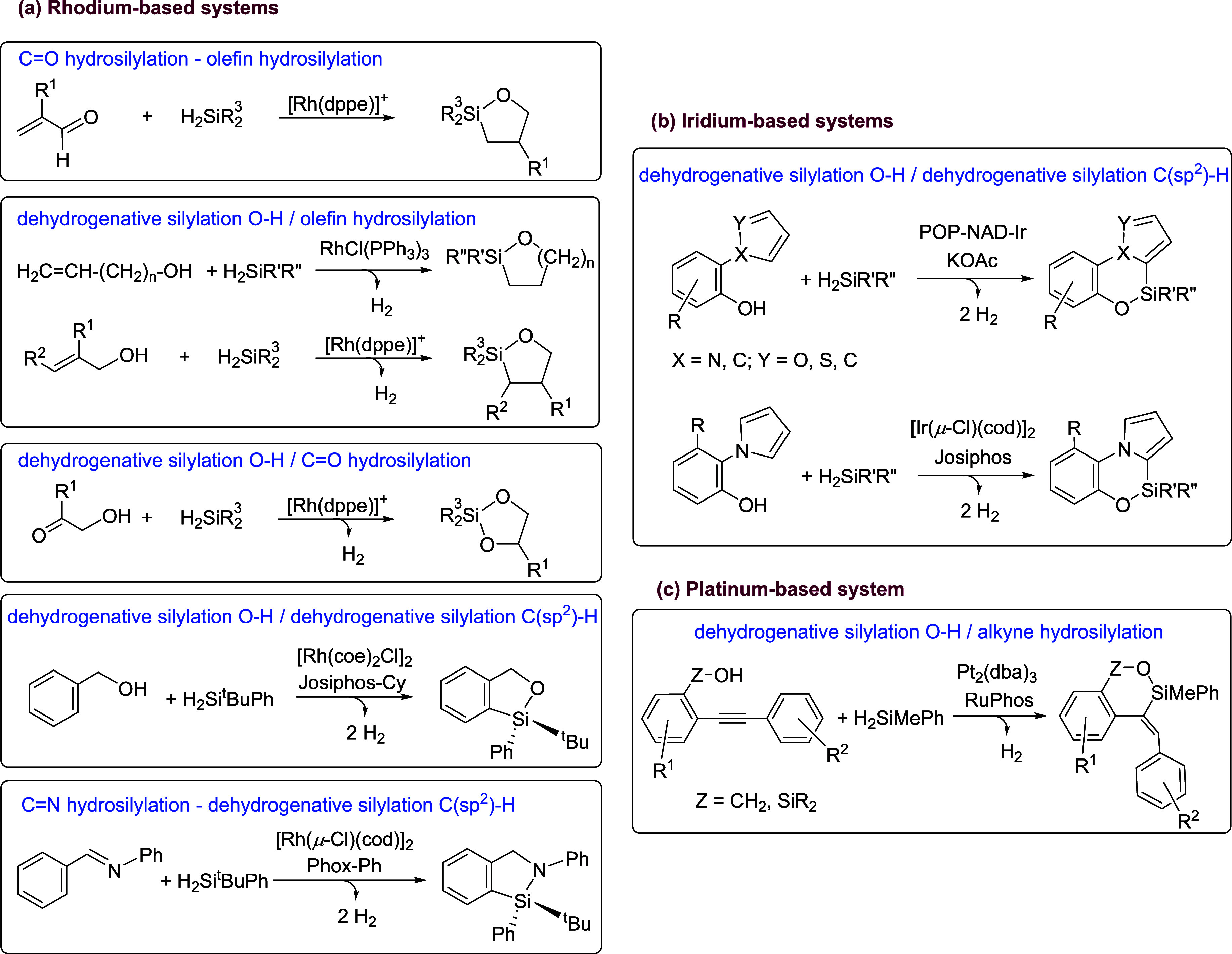
Tandem [*n* + 1] (*n* = 4, 5) Annulative Processes Using Dihydrosilanes Catalyzed by a Single Catalyst

Polyhydride derivatives have wide application in catalysis,^21^ and particularly in processes of hydrogenation and dehydrogenation. Among the latter, largely driven by the use of molecular dihydrogen as an energy carrier,^22^ dehydrogenation reactions of alkanes,^23^ amines,^24^ alcohols,^25^ formic acid,^26^ ammonia–borane, and amine–borane^27^ stand out. Therefore, it is surprising that despite the relevance that iridium-polyhydride derivatives have as intermediates in the dehydrogenative silylation reaction depicted in Scheme 1, the use of other polyhydrides in this process has been overlooked.

We have recently reported that hexahydride osmium­(VI) complex OsH_6_(P^i^Pr_3_)_2_ reacts with diphenylsilane and phenylsilane to afford pentahydride-silyl-osmium­(VI) OsH_5_(SiHPh_2_)­(P^i^Pr_3_)_2_ (**1**) and tetrahydride-bis­(silyl)-osmium­(VI) OsH_4_(SiH_2_Ph)_2_(P^i^Pr_3_)_2_ (**2**), respectively (Scheme 3).^28^ Interestingly, complex **1** catalyzes the hydrosilylation of carbonyl groups of a variety of aldehydes and ketones. In addition to the hydrosilylation products, the formation of dehydrogenative silylation derivatives, along with the concomitant production of hydrogen, is observed when enolyzable ketones are used.

**Scheme 3:**
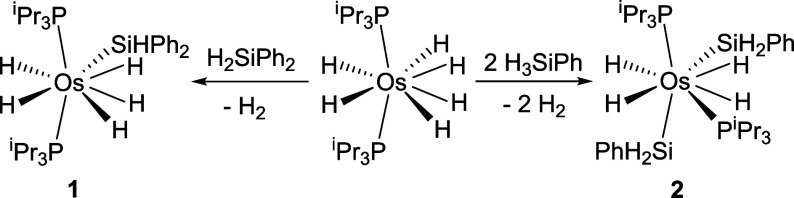
Reactions of OsH_6_(P^i^Pr_3_)_2_ with H_2_SiPh_2_ and H_3_SiPh

Based on this precedent, we decided to investigate the silylative dehydrogenation of alcohols with diphenylsilane using complex **1** as catalyst precursor. In this contribution we report its performance in this process, its ability to catalyze the synthesis of six-membered silacycles via tandem hydrosilylation/dehydrogenative silylation of salicylaldehydes with diphenylsilane, as well as the study of both mechanisms by DFT calculations. In addition, we show that the alcoholysis or aminolysis of the Si–H bonds of complexes **1** and **2** with alcohols or amines bearing a coordinating group such as pyridine leads to complexes with bidentate κ^2^-*Si*,*N* ligands.

## Results and Discussion

### Monoalcoholysis of Diphenylsilane

The reaction conditions for the monoalcoholysis of diphenylsilane were optimized using 0.28 M solutions of phenol and the silane in toluene (1 mL), variable amounts of catalyst precursor **1**, and different temperatures. The progress of the reactions was monitored by measuring the pressure of the hydrogen generated. At 80 °C and using 0.014 M solutions of **1** (5 mol %), phenol is quantitatively converted to HSi­(OPh)­Ph_2_ in 21 min, while when the catalyst loading is reduced to 2.5 mol % the time required increases to 80 min. At 60 °C and 5 mol % of **1** the reaction takes 750 min to completion. Therefore, we decided to use 5 mol % of catalyst **1**, 0.28 M solutions of diphenylsilane and of the corresponding alcohol in toluene (1 mL) and 80 °C to perform our study. Under these conditions, complex **1** is an efficient catalyst precursor for the dehydrogenative coupling of diphenylsilane with a variety of aromatic and aliphatic alcohols affording selectively the desired monosilylated products HSi­(OR)­Ph_2_ in good to excellent yields (Table 1).

**Table 1:**
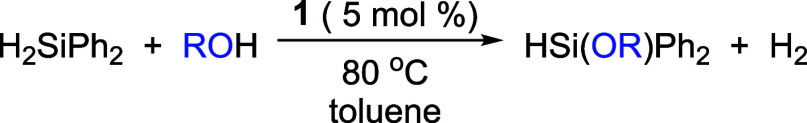
Monoalcoholysis of Diphenylsilane Catalyzed by OsH_5_(SiHPh_2_)­(P^i^Pr_3_)_2_ (**1**)­^a^

entry	alcohol	product	time (min)	conversion (isolated yield)
1	PhOH	HSi(OPh)Ph_2_	21	>99% (51%)
2	4-MeO-PhOH	HSi(OPh-4-OMe)Ph_2_	20	>99% (70%)
3	4-^t^Bu-PhOH	HSi(OPh-4-^t^Bu)Ph_2_	16	>99% (66%)
4	4-Cl-PhOH	HSi(OPh-4-Cl)Ph_2_	21	>99% (72%)
5	4-CF_3_–PhOH	HSi(OPh-4-CF_3_)Ph_2_	38	>99% (70%)
6	2-OH-py			no reaction
7	PhCH_2_OH	HSi(OCH_2_Ph)Ph_2_	8	>99% (95%)
8	^n^PrOH	HSi(O^n^Pr)Ph_2_	25	>99% (67%)
9	^n^BuOH	HSi(O^n^Bu)Ph_2_	25	>99% (67%)
10	^n^PrCH(OH)Et	HSi{OCH(^n^Pr)Et}Ph_2_	100	>99% (74%)
11	CyOH	HSi(OCy)Ph_2_	47	>99% (60%)
12	CyCH(OH)CH_3_	HSi{OCH(CH_3_)Cy}Ph_2_	30	>99% (72%)
13	^t^BuOH	HSi(O^t^Bu)Ph_2_	113	>99% (69%)

a Conditions: **1** (0.014 mmol), H_2_SiPh_2_ (0.28 mmol), alcohol (0.28 mmol), and toluene (1 mL). The final time of the reaction was considered the one where hydrogen evolution had ceased. The conversion yields were determined by ^1^H NMR of the crude reaction mixture using 1,4-dioxane as internal standard; isolated yields are given in parentheses.

In all cases, except for 2-hydroxypyridine, quantitative evolution of hydrogen was observed and the formation of the monoalcoholysis products was confirmed by ^1^H NMR spectroscopy. For *para*-substituted phenols, the reactions are little sensitive to the electronic nature of the substituents in *para* position. Phenols having electron-donating groups such as methoxy and *t*-butyl groups react slightly faster (entries 2 and 3) than those bearing electron-withdrawing ones (entries 4 and 5). The absence of reaction when 2-hydroxypyridine is used was attributed to the deactivation of the catalyst via heteroatom chelation, as was confirmed by the stoichiometric reaction carried out (*vide infra*). In the case of aliphatic alcohols, we have used primary, secondary, and tertiary alcohols. The reactions with primary alcohols such as benzyl alcohol, propan-1-ol, and butan-1-ol (entries 7–9) require less time than those of secondary (entries 10–11) and tertiary alcohols (entry 13), suggesting that the diphenylsilane monoalcoholysis catalyzed by complex **1** depends on the nucleophilicity of the used alcohol, and that the alcohol attack to the silicon atom is the rate-determining step of the processes.^5d,29^

The robustness of this catalytic system was confirmed through successive additions of benzyl alcohol. The sequential reaction profiles obtained showed that it could be used for at least ten consecutive runs without significant drop in activity and decrease in the yield of the monoalcoholysis product (Figure 1).

**Figure 1:**
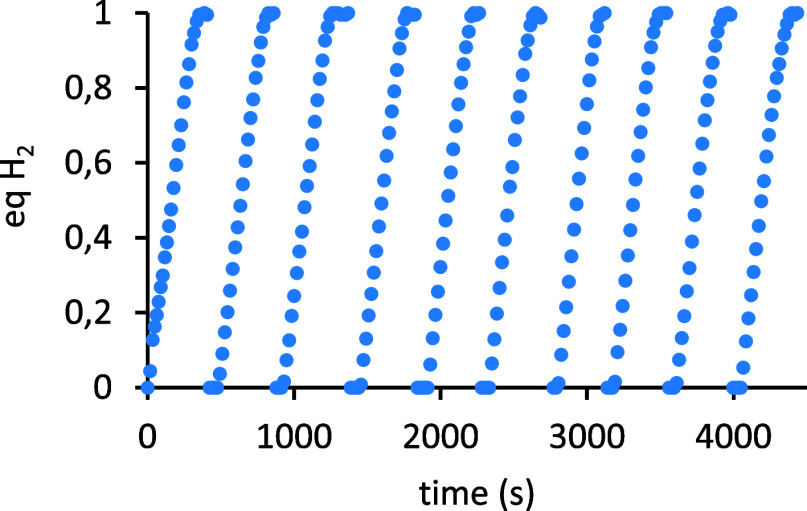
Reaction profiles obtained by successive additions of 0.256 mmol of benzyl alcohol to a toluene solution containing 0.014 mmol of compound **1** and 2.96 mmol of diphenylsilane. Hydrogen production calculated by monitoring of the pressure evolution using a pressure transducer (Man on the Moon X102 kit).

Other secondary silanes, such as methylphenylsilane, can be used in this reaction. Thus, under the same experimental conditions, this silane reacts with phenol, benzyl alcohol, and cyclohexanol to afford HSi­(OPh)­MePh, HSi­(OCH_2_Ph)­MePh, and HSi­(OCy)­MePh after 60, 50, and 90 min, respectively (isolated yields 72, 72, and 70%).

NMR experiments were performed in order to obtain information about the reaction mechanism. For this purpose, the monoalcoholysis of phenol with diphenylsilane using 20 mol % of **1** in benzene-*d*_6_ was monitored by ^1^H and ^31^P­{^1^H} NMR spectroscopies. In the ^1^H NMR spectra, in addition to the resonances of the monoalcoholysis product and those distinctive for **1** (^1^H: δ −9.80 (t, ^2^ *J*_H–P_ = 10.2 Hz); ^31^P­{^1^H}: δ 46.1 (s)),^28^ a triplet (^2^ *J*_H–P_ = 9.4 Hz) at −9.47 ppm is observed, while the ^31^P­{^1^H} NMR spectra contain a singlet at 44.6 ppm (Figures S32 and S33). We tentatively proposed that these resonances could belong to complex OsH_5_{Si­(OPh)­Ph_2_}­(P^i^Pr_3_)_2_, closely related to pentahydride **1**. This has been confirmed upon reaction of complex **1** with 1.05 equiv of phenol. Thus, treatment of toluene solutions of **1** with this alcohol, at 80 °C, for 5 h leads to pentahydride derivative OsH_5_{Si­(OPh)­Ph_2_}­(P^i^Pr_3_)_2_ (**3**) (Scheme 4). This compound is the result of the alcoholysis of the Si–H bond and was isolated as a white solid in good yield and characterized by HRMS, elemental analysis, IR, and ^1^H, ^13^C­{^1^H}, ^31^P­{^1^H}, and ^29^Si­{^1^H} NMR spectroscopy. Furthermore, its structure was confirmed by X-ray diffraction analysis. A view of its molecular geometry is depicted in Figure 2a. Similarly to other eight-coordinate osmium-polyhydrides,^28,30^ the coordination geometry of complex **3** around the osmium center can be rationalized as derived from a distorted dodecahedron, defined by two intersecting BAAB orthogonal (87.51°) trapezoidal planes (Figure 2b). One of them contains the atoms P(1), P(2), H(04), and H(05) (maximum deviation 0.235 Å for H(04)), while the other one is formed by the silicon atom and hydrides H(01), H(02), and H(03) (maximum deviation 0.17 Å for H(01)). The distances of the silicon atom to the nearest hydride ligands, 2.01(4) and 2.29(4) Å, are in the range reported for secondary interactions between silicon and hydrogen atoms (SiSHA),^31^ but the analysis of the bond situation by DFT using the Atoms in Molecules (AIM) theory reveals the absence of bond critical points (BCPs) between the silicon atom and the hydride ligands of pentahydride **3** (Figure S4).

**Scheme 4:**
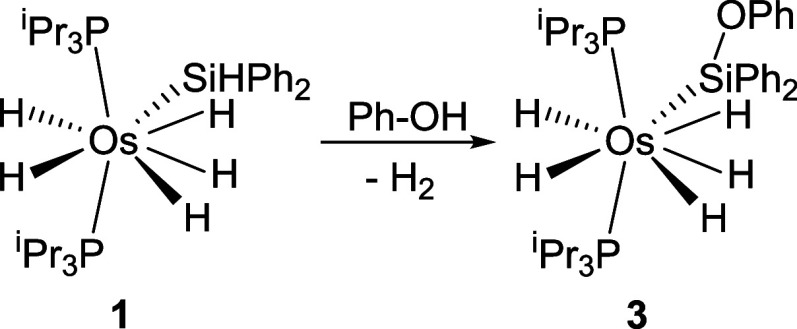
Reaction of **1** with Phenol

**Figure 2:**
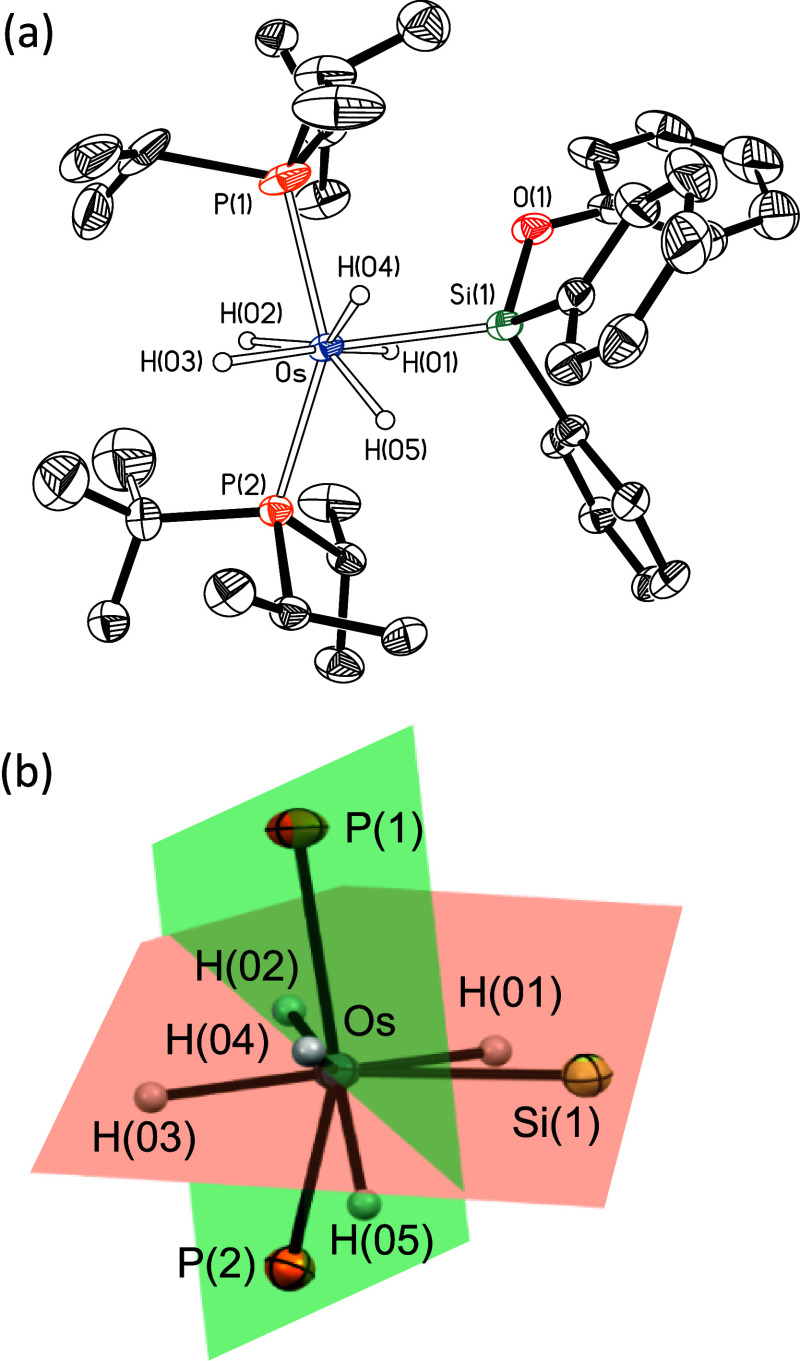
(a) Molecular diagram of complex **3** (displacement ellipsoids shown at 50% probability). All hydrogen atoms (except the hydrides) are omitted for clarity. Selected bond distances (Å) and angles (°): Os–P(1) = 2.3565(9), Os–P(2) = 2.3707(8), Os–Si(1) = 2.4051(10), Si(1)–O(1) = 1.697(3); P(1)–Os–P(2) = 143.90(4), Si(1)–Os–P(1) = 96.24(4), Si(1)–Os–P(2) = 113.17(3). (b) View of the two intersecting BAAB orthogonal trapezoidal planes.

The structure of this complex is not rigid in solution and, accordingly, the high field region of its ^1^H NMR spectrum in benzene-*d*_6_ recorded at 298 K contains only a triplet at −9.47 (^2^ *J*_H–P_ = 9.4) ppm, and just a slight broadening of this resonance is observed if the sample temperature is lowered up to 183 K in toluene-*d*_8_. This indicates that the hydride ligands are involved in thermally activated position exchange processes and that their activation barriers are very low. In agreement to its pentahydride nature, it displays a 300 MHz *T*_1_(min) value of 145 ± 5 ms at 233 K for the hydride resonances. Other characteristic features of this compound are singlets at 44.6 and 18.2 ppm in the ^31^P­{^1^H} and ^29^Si­{^1^H} NMR spectra, respectively.

Since the stoichiometric reaction does not shed much light on the mechanism, we performed DFT (B3LYP-D3/SDD­(f)-631g**) calculations on the monoalcoholysis of diphenylsilane using phenol as alcohol model, 298.15 K, 1 atm, and considering the implicit solvent effect by means of the Polarizable Continuum Method (benzene). Since the loss of hydrogen from pentahydride-silyl complex **1** is not observed experimentally, we have investigated different pathways starting from the intermolecular attack of phenol to **1**. This nucleophilic attack of the alcohol to the silicon atom can take place in back-side or front-side fashions with respect to the leaving group.^10^ The back-side attack would give the phenol stabilized silylium cation [HSi­(HOPh)­Ph_2_]^+^ and anionic pentahydride intermediate [OsH_5_(P^i^Pr_3_)_2_]^−^, which turns to be a stronger base than the alcohol itself, making this a nonproductive pathway. However, and despite the higher steric congestion, the front-side attack, where phenol approaches the silicon center from the same side as the metal fragment, would lead to a highly ordered low-energy transition state (**TS**_**1‑A**_ in Figure 3b). This transition state, situated 26.0 kcal mol^–1^ over **1** (Figure 3a), implies the nucleophilic attack of the alcohol to the silane coordinated to the metal center in an η^1^-H–SiHPh_2_ fashion. Although the sigma intermediate OsH_4_(η^1^-H–SiHPh_2_)­(P^i^Pr_3_)_2_ is not observed as a minimum on the potential energy surface, the approach of the alcohol to **1** induces the elimination of diphenylsilane from **1** and its coordination in an η^1^ fashion. Transition state **TS**_**1‑A**_ provides concerted and synchronous Si–H bond cleavage, Si–O bond formation, and Os-(η^2^–H_2_) formation that directly leads to the monoalcoholysis product and the intermediate OsH_4_(η^2^-H_2_)­(P^i^Pr_3_)_2_ (**A**). The displacement of the coordinated molecular hydrogen from **A** by an incoming diphenylsilane molecule renders **1** through transition state **TS**_**A‑1**_, located 11.6 kcal mol^–1^ above **1**. Further support for this mechanistic proposal arises from the substitution of phenol by benzylic alcohol. In agreement with the experimental observations, and with the higher nucleophilicity of benzylic alcohol, the activation barrier decreases from 26.0 to 20.7 kcal mol^–1^ (Figure S1). In the case of the stoichiometric reaction of complex **1** with phenol, the released HSi­(OPh)­Ph_2_ would react with intermediate **A** to afford complex **3** via a transition state related to **TS**_**A‑1**_ situated 18.4 kcal mol^–1^ above **A** (Figure S2).

**Figure 3:**
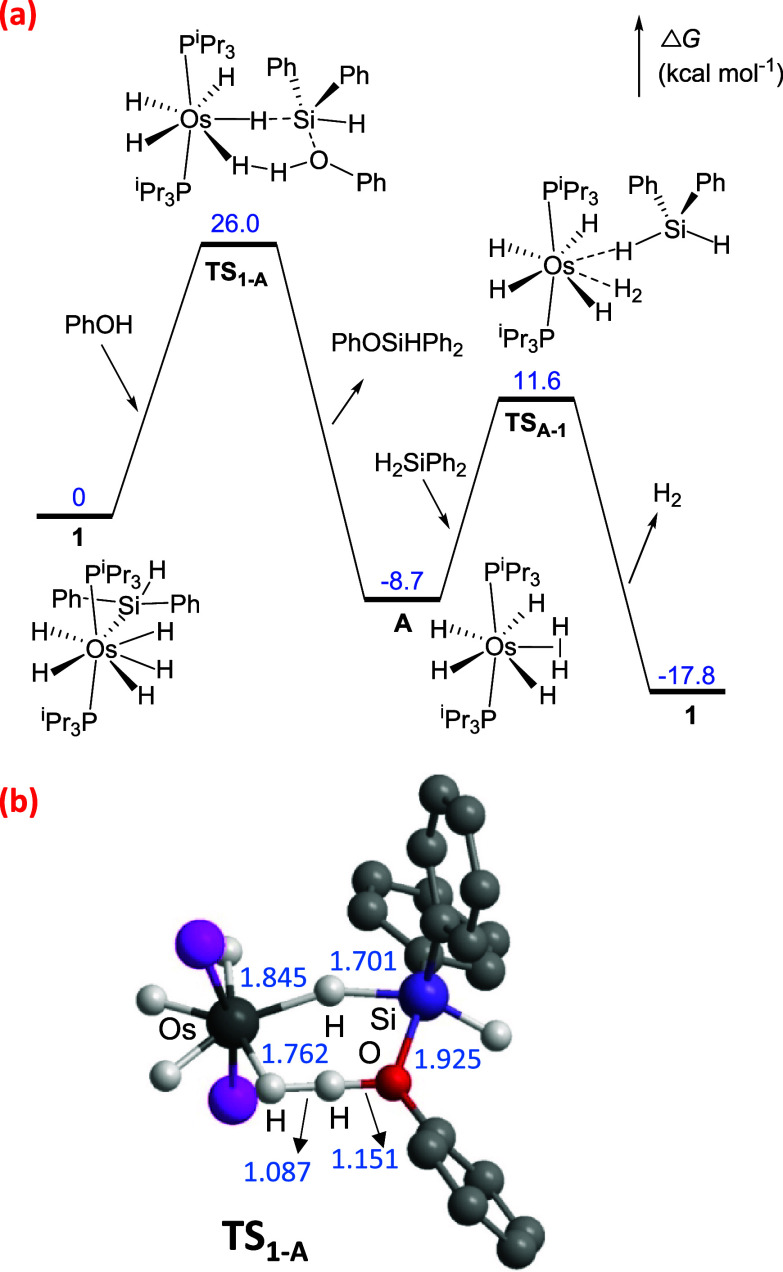
(a) Energy profile (kcal mol^–1^) for the dehydrogenative coupling of phenol and diphenylsilane catalyzed by OsH_5_(SiHPh_2_)­(P^i^Pr_3_)_2_ (**1**). (b) Transition state **TS**_**1‑A**_ showing selected distances (Å) between atoms (phosphine substituents and hydrogens of the phenyl rings have been omitted for clarity).

### Reactions of Complex **1** with 2-Hydroxy- and 2-Aminopyridine

In an attempt to gain some understanding of why the alcoholysis of 2-hydroxypyridine does not work, we performed the stoichiometric reaction of pentahydride **1** with 2-hydroxypyridine. Thus, treatment at 80 °C of toluene solutions of **1** with 1.2 equiv of 2-hydroxypyridine leads after 3 h to the trihydride derivative OsH_3_{κ^2^-*Si,N*-(SiPh_2_-O-py)}­(P^i^Pr_3_)_2_ (**4**). Under similar conditions, its reaction with 2-aminopyridine affords OsH_3_{κ^2^-*Si,N*-(SiPh_2_-NH-py)}­(P^i^Pr_3_)_2_ (**5**). These compounds are the result of the alcoholysis (**4**) and aminolysis (**5**), respectively, of the Si–H bond of the silyl group of complex **1**, release of two equiv of hydrogen, and coordination of the pyridine to the osmium center (Scheme 5).

**Scheme 5:**
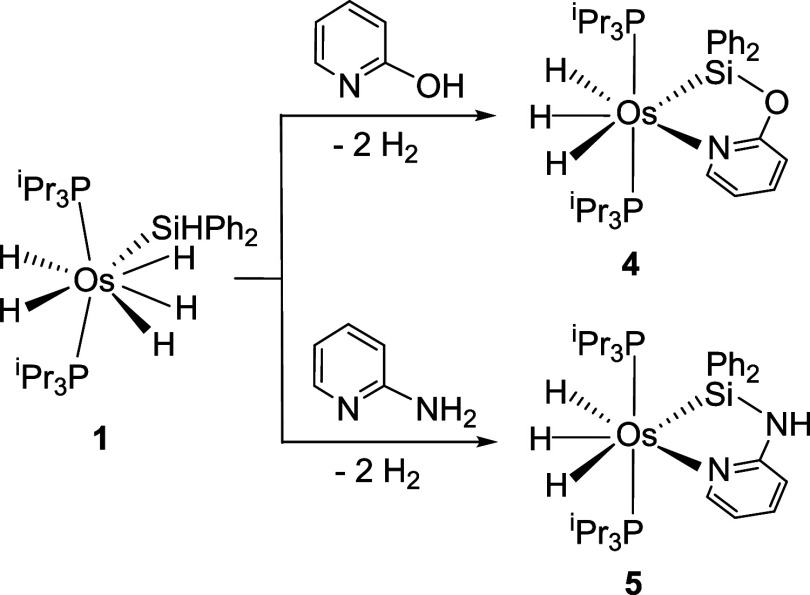
Reactions of 1 with 2-Hydroxypyridine and 2-Aminopyridine

Complexes **4** and **5** were isolated as white solids in good yields (78–65%) and were characterized by HRMS, elemental analysis, IR, and ^1^H, ^13^C­{^1^H}, ^31^P­{^1^H}, and ^29^Si­{^1^H} NMR spectroscopy. Complex **4** was further characterized by an X-ray crystallographic study, that confirms the formation of the five-membered metalaring containing a chelating κ^2^-*Si*,*N* ligand (Figure 4). The coordination geometry around the osmium atom can be rationalized as a distorted pentagonal bipyramid with the two phosphorus atoms of the phosphine ligands occupying axial positions (P(1)–Os–P(2) = 159.679(11)°). The osmium sphere is completed by the bidentate pyridine-2-yloxy-silyl ligand (Si(1)–Os–N(1) = 77.59(3)°) and the hydride ligands. The Os–Si(1) distance (2.3783(3) Å) is slightly shorter than that found in complex **3** (2.4051(10) Å), while the Si(1)–O(1) separation (1.7316(10) Å) is longer than the related parameter in **3** (1.697(3) Å). This could be due to some participation of the base-stabilized silylene resonance form **II** in addition to the silyl form **I** (Scheme 6) to the structure of **4** and **5**. This is also supported by the sume of the angles around the silicon atom excluding those forming with the oxygen atom, 348° (335.64° for **3**), value intermediate between tetrahedral (329°) and trigonal (360°) angles.

**Figure 4:**
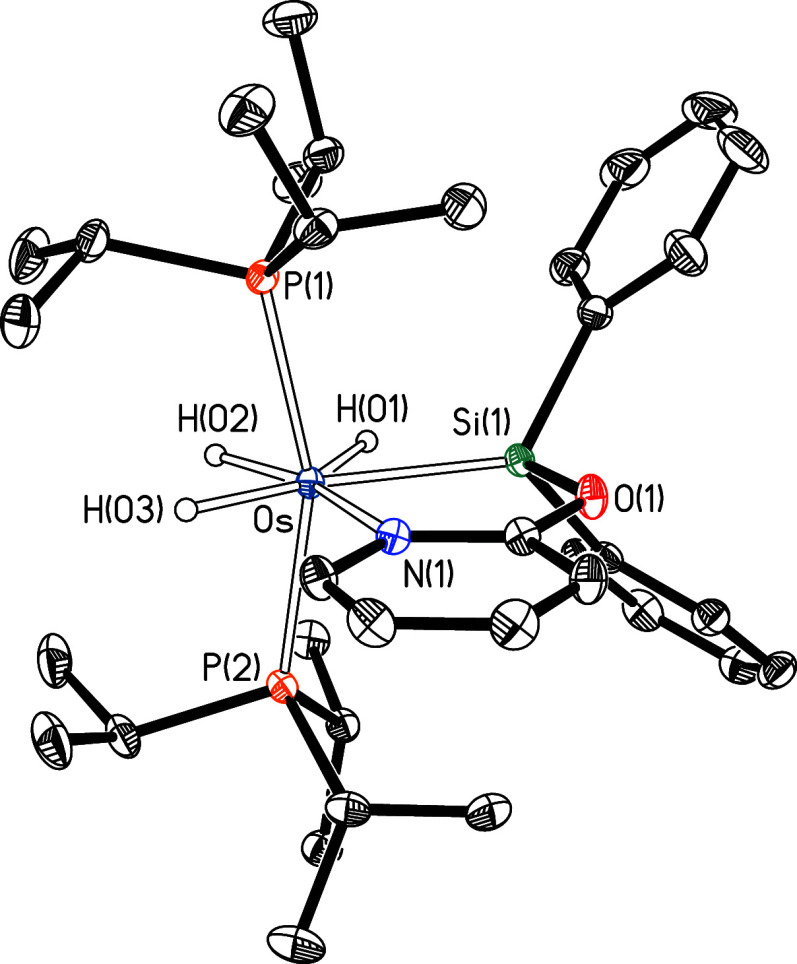
Molecular diagram of complex **4** (displacement ellipsoids shown at 50% probability). All hydrogen atoms (except the hydrides) are omitted for clarity. Selected bond distances (Å) and angles (°): Os–P(1) = 2.3471(3), Os–P(2) = 2.3458(3), Os–Si(1) = 2.3783(3), Os–N(1) = 2.1902(10), Si(1)–O(1) = 1.7316(10); P(1)–Os–P(2) = 159.679(11), Si(1)–Os–N(1) = 77.59(3), Si(1)–Os–P(1) = 99.568(12), Si(1)–Os–P(2) = 100.678(12), N(1)–Os–P(1) = 93.32(3), N(1)–Os–P(2) = 92.71(3).

**Scheme 6:**
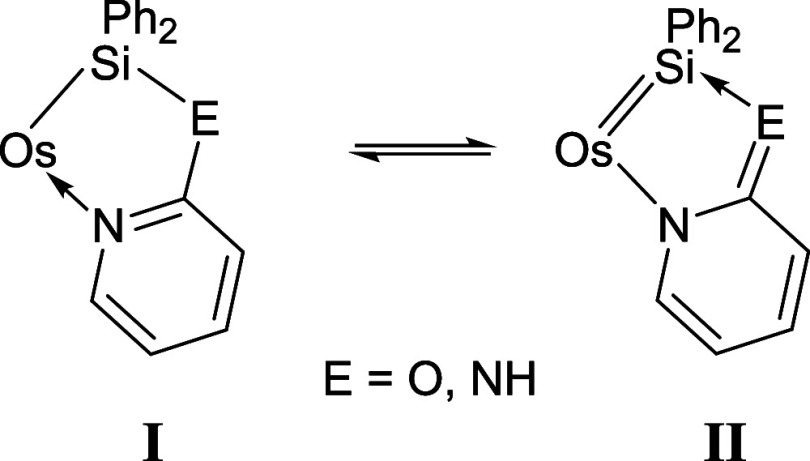
Resonance Forms for the Five-Membered-Ring of Complexes **4** and **5**

To gain more information about the nature of the Os–Si bonds and the participation of resonance form **II** into the structures of **4** and **5**, theoretical calculations were carried out at the DFT level BP86-D3/def2TZVPP. Natural Bonding Orbital (NBO) analysis of both complexes reveals that the Wiberg Bond Indexes (WBIs) of the Os–Si bonds are 0.46 and 0.45, respectively (Table 2 and Chart 1), suggesting a moderately strong bond, typical of silyl ligands (0.35–0.70).^32^ Topological analysis of the electron density located BCPs associated with the Os–Si bonds. The AIM analysis at these BCPs shows values of ∇^2^ρ­(r) (e·Å^–3^) slightly negatives, and values of the ratio between the electronic potential and kinetic electronic energy densities, |V­(r)|/G­(r), of 2.6, typical for shared shell (covalent) bonds. These data, along with those of their own values of electron densities, ρ­(r), are consistent with highly polarized covalent osmium-silyl bonds. The relatively large differences between the natural charges of Os and Si (Δq­(Os–Si) ≈ 2.3–2.4) are also more typical of metal-silyl complexes than of base-stabilized metal-silylene derivatives, in which the degree of polarization is lower.^33^ However, NBO calculations of the second-order stabilization energies in both complexes, E,^2^ best describe the Si–E (E = O, NH) bonds as a donor–acceptor interaction between a lone pair (LP) of electrons of the E atoms and an empty nonbonding valence orbital (LV) on the silicon atom. These results suggest a minor contribution of the base-stabilized silylene resonance form to the description of the Os–Si bonds in **4** and **5**.

**Table 2:** NBO and AIM Selected Properties for Complexes 4 (E = O) and 5 (E = N)

**Os–Si bond**									
comp.	d(Os–Si)_exp_ (Å)	d(Os–Si)_DFT_(Å)	Δq(Os–Si)(e)	WBI (Os–Si)	∇^2^ρ(r) (e Å^–3^)	ρ(r)	ε(r)	|V(r)|/G(r)	
**4**	2.3783(3)	2.3975	2.38	0.464	–0.074	0.093	0.076	2.60	
**5**		2.4106	2.28	0.449	–0.067	0.091	0.107	2.56	
**Si–E bond**									
comp.	d(Si-E)_exp_ (Å)	d(Si-E)_DFT_ (Å)	Δq(Si-E) (e)	WBI (Si-E)	∇^2^ρ(r) (e Å^–3^)	ρ(r)	ε(r)	|V(r)|/G(r)	E^(2)^ LP(E)→LV(Si)(kcal mol^–1^)
**4**	1.732(1)	1.770	2.32	0.459	0.471	0.104	0.009	1.24	135.51
**5**		1.809	2.40	0.560	0.397	0.108	0.052	1.31	174.39

**Chart 1:**
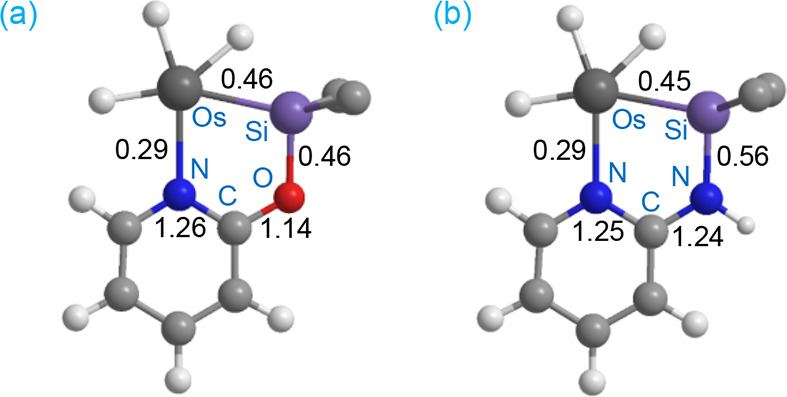
Wiberg Bond Indices for the Bonds of the Five-Membered Chelate Rings κ^2^-(*Si*,*N*) of complexes **4** (a) and **5** (b)

The NMR spectra of complexes **4** and **5** are consistent with the structure shown in Figure 4. As expected for three inequivalent hydride ligands, the ^1^H NMR spectra registered at room temperature in benzene-*d*_6_ contain three hydride resonances at around −4.30 (H_a_), −12.80 (H_b_), and −14.20 (H_c_) ppm (Figure 5a shows those of complex **5**). At this temperature, the H_a_ signals appear as triplets (^2^ *J*_H–P_ ≈ 23.6 Hz), while resonances H_b_ and H_c_ are observed as broad signals, that upon lowering the temperature appear as doublets of triplets (toluene-*d*_8_, 243 K; H_b_: ^2^ *J*_H–H_ = 5.4, ^2^ *J*_H–P_ = 6.0 (**4**); ^2^ *J*_H–H_ = 9.6, ^2^ *J*_H–P_ = 6.5 (**5**). H_c_: ^2^ *J*_H–H_ = 5.4, ^2^ *J*_H–P_ = 18.6 (**4**); ^2^ *J*_H–H_ = 9.6, ^2^ *J*_H–P_ = 19.8 (**5**); Figure 5b). At 343 K (**4**) and 323 K (**5**), coalescence between hydride resonances H_b_ and H_c_ takes place, and the activation barrier for the exchange between them is 15.5 kcal mol^–1^ for **4** and 14.5 kcal mol^–1^ for **5**. The *T*_1_(min) values for the hydride resonances (see Experimental Section) support the trihydride character of these complexes and suggest that the central atom of the OsH_3_ unit is H_c_. The NOE (nuclear Overhauser effect) spectrum recorded for complex **5** (Figure S45) shows that hydride H_a_ is that situated *cis* to the pyridyl ring, and *transoid* to the silicon atom. In agreement with this, in the ^1^H­{^31^P} NMR spectrum recorded at 243 K, the H_a_ resonance appears as a singlet flanked by ^29^Si satellites (^2^ *J*_H–Si_ = 32 Hz; Figure 5c).^34^ In agreement with the equivalence of the phosphine ligands, the ^31^P­{^1^H} NMR spectra in toluene-*d*_8_ shows singlets around 29 ppm, which are temperature invariant between 373 and 193 K. The ^29^Si­{^1^H} NMR spectra contain triplets (*J*_Si–P_ ≈ 6.5 Hz) at 55.9 (**4**) and 30.3 ppm (**5**). These values are displaced, respectively, 33.7 and 12.1 ppm downfield compared to the resonance of complex **3**, pointing out to the contribution of the silylene resonance form to the overall structure of these derivatives.

**Figure 5:**
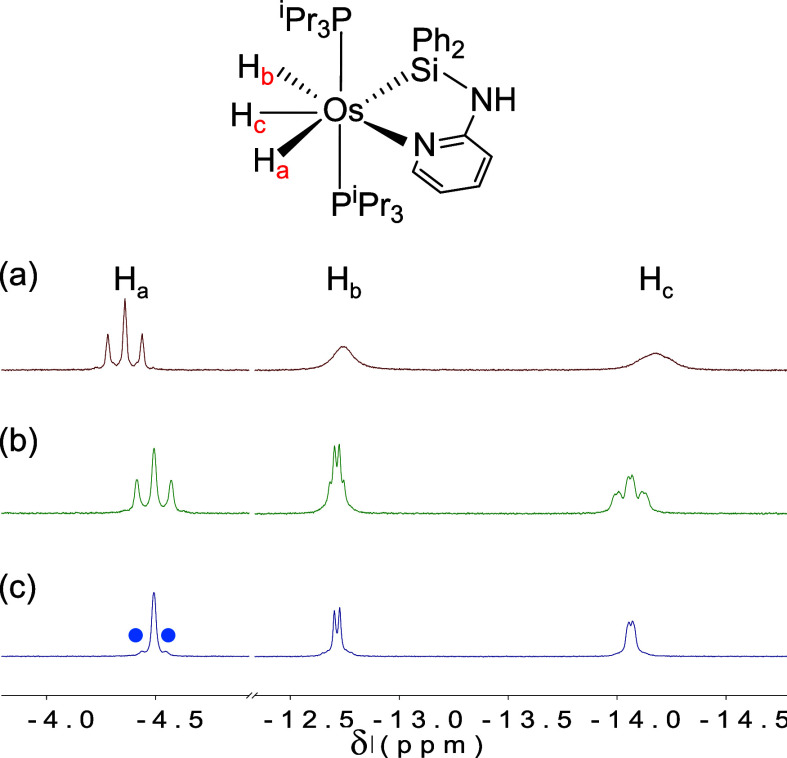
High field region of the ^1^H NMR spectra (300 MHz, toluene-*d*_8_) of complex **5** at 298 K (a) and at 243 K (b). (c) High field region of the ^1^H­{^31^P} NMR spectrum of **5** at 243 K. Blue dots denote the ^29^Si satellites.

This reaction constitutes a new methodology for the synthesis of transition-metal complexes containing monoanionic bidentate κ^2^-*N*,*Si* pyridine-silyl ligands.^35^ They are usually prepared by pyridine directed oxidative addition of pyridyl-2-yloxi silanes to low valent metal complexes,^33,36^ with a few exceptions. Thus, the synthesis of M­(η^5^-C_5_Me_5_)­{κ^2^-*N,Si*-Si­(*p*-tolyl)_2_O-py}­(CO)_2_ (M = Mo, W) by reaction of η^3^-α-silabenzyl species [M­(η^5^-C_5_Me_5_)­{κ^2^-*Si,C,C*-Si­(*p*-tolyl)_3_}­(CO)_2_ (M = Mo, W) with 2-substituted pyridines was described by Tobita and co-workers,^37^ while a nucleophilic substitution reaction at the Si–Cl bond of a chloro­(diphenyl)­silyl ligand in ruthenium complexes was reported by Roper and co-workers.^38^ These authors, taking advantage of the latter reaction, described the reaction of Os­(SiMeCl_2_)­Cl­(CO)­(PPh_3_)_2_ with two equiv of 2-aminopyridine to give Os­(κ^3^-*Si*,*N*,*N*-SiMe­[NH­(2-C_5_H_4_N)]_2_)­Cl­(CO)­(PPh_3_), that contains a tridentate N,Si,N ligand *fac* coordinated and results from a nucleophilic substitution reaction at the Si–Cl bonds of the dichloro­(methyl)­silyl ligand and the displacement of one of the phosphine ligands of the starting material.^39^ With the exception of this example, the rest of reported complexes containing a N,Si,N ligand are the result of the oxidative addition of a Si–H bond of an N,Si­(H),N proligand.^40^

In view of these precedents, we decided to explore the reactivity of the tetrahydride derivative OsH_4_(SiH_2_Ph)_2_(P^i^Pr_3_)_2_ (**2**),^28^ that has two phenylsilyl groups with two Si–H bonds available on each of them, toward 2-hydroxypyridine and 2-aminopyridine. Thus, the treatment of toluene solutions of **2** with 2.2 equiv of 2-hydroxypyridine or 2-aminopyridine for 4 h at 80 °C leads to complexes OsH_3_{κ^2^-*Si,N*-(SiPh­(Epy)-E-py)}­(P^i^Pr_3_)_2_ (E = O (**6**), NH (**7**)) (Scheme 7), that were isolated as white solids in moderate yields (57–75%). These derivatives arise from the alcoholysis (**6**) and aminolysis (**7**), respectively, of both Si–H bonds of one the silyl groups of complex **2**, release of phenylsilane and of two equiv of hydrogen, and coordination of the pyridine to the osmium center. Figure 6 shows a drawing of the X-ray structure of complex **6**.

**Scheme 7:**
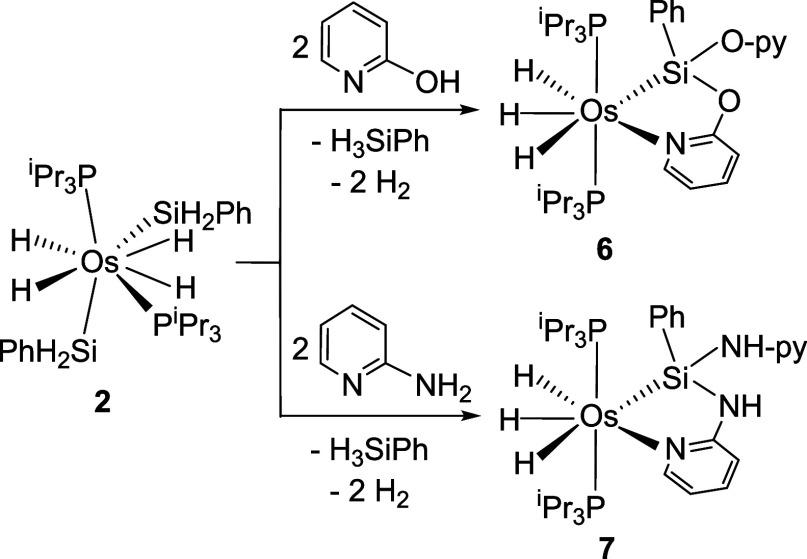
Reactions of 2 with 2-Hydroxypyridine and 2-Aminopyridine

**Figure 6:**
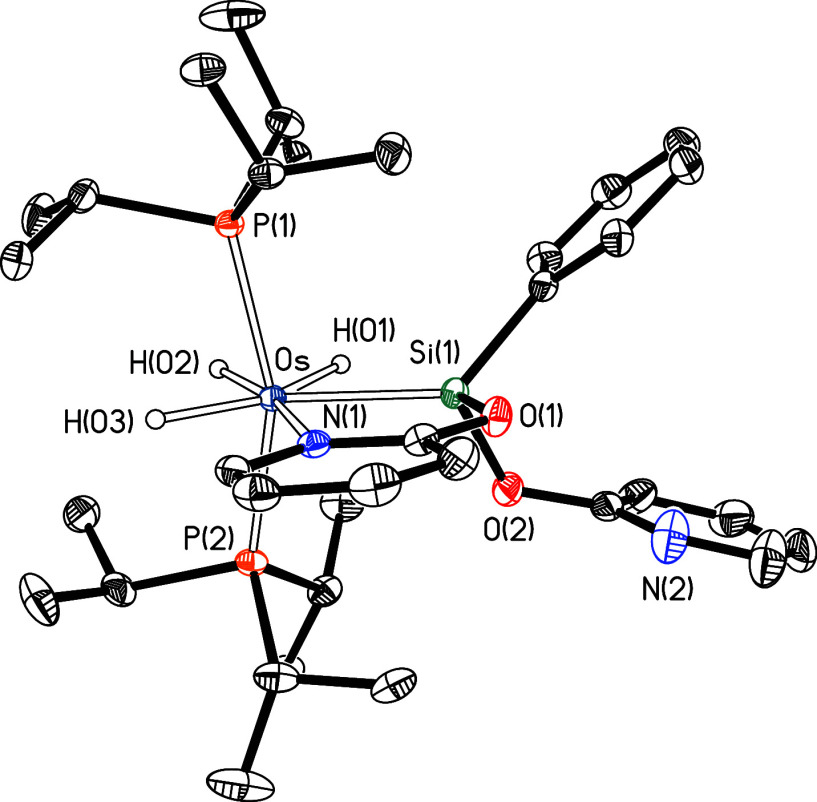
Molecular diagram of complex **6** (displacement ellipsoids shown at 50% probability). All hydrogen atoms (except the hydrides) are omitted for clarity. Selected bond distances (Å) and angles (°): Os–P(1) = 2.3420(4), Os–P(2) = 2.3407(4), Os–Si(1) = 2.3408(4), Os–N(1) = 2.1966(12), Si(1)–O(1) = 1.7192(10), Si(1)–O(2) = 1.6993(11); P(1)–Os–P(2) = 158.904(13), Si(1)–Os–N(1) = 77.40(3), Si(1)–Os–P(1) = 101.891(13), Si(1)–Os–P(2) = 98.145(14), N(1)–Os–P(1) = 92.64(3), N(1)–Os–P(2) = 98.14(3).

The structure proves the alcoholysis of both Si–H bonds, but there is no displacement of any of the phosphines, since only one of the pyridine rings is coordinated to the osmium center. The coordination geometry around the osmium atom is very similar to that described for compound **4** and can be rationalized as a distorted pentagonal bipyramid with the two phosphorus atoms of the phosphine ligands occupying axial positions (P(1)–Os–P(2) = 158.904(13)°). The osmium sphere is completed by the bidentate pyridine-2-yloxy-silyl ligand (Si(1)–Os–N(1) = 77.40(3)°) and the hydride ligands. The values of the Os–Si(1) (2.3408(4) Å) and Si(1)–O(1) bond distances (1.7192(10) Å) as well as the sum of the angles around the silicon atom excluding those forming with O(1), 340.5°, suggest an even smaller contribution of a base-stabilized resonance form related to **II** (Scheme 6) compared to complexes **3** and **4**. The results of the NBO and AIM analyses also point to that (Table S1).

The ^1^H NMR spectra of derivatives **6** and **7** are consistent with the structure depicted in Figure 6 and similar to those described for the related complexes **3** and **4**. In this case the coalescence of the two hydride resonances at higher field takes place at 328 K (**6**) and 323 K (**7**), and the activation barrier for the exchange between them is 14.8 kcal mol^–1^ for **6** and 14.5 kcal mol^–1^ for **7**. In the ^31^P­{^1^H} NMR spectra recorded in toluene-*d*_8_ at 253 K, and in agreement with the nonequivalence of the phosphine ligands induced by the different noncoordinated substituents of the silyl group (Ph and Opy/NHpy), AB spin systems around 32 ppm are observed. The chemical shifts of the resonances of the ^29^Si nuclei appear at 59.6 (**6**; by a 2D ^1^H,^29^Si HMBC NMR experiment) and 31.8 ppm (**7**), respectively.

### Tandem Hydrosilylation/Dehydrogenative Silylation of Salicylaldehydes

Since complex **1** catalyzes both the hydrosilylation of carbonyl compounds with diphenylsilane^28^ and the monoalcoholysis of this silane with alcohols, we wondered if this complex might be able to catalyze both processes to convert salicylaldehydes into 2,2-diphenyl-4*H*-1,3,2-benzodioxasiline type molecules (Chart 2). These molecules have been used in simultaneous twin polymerization with 2,2’-spiro­[4*H*-1,3,2-benzodioxasiline] derivatives to afford nanostructured ternary organic–inorganic hybrid materials^41^ and are prepared by stoichiometric reactions of *ortho*-hydroxybenzyl alcohol with the dichlorosilane. This reaction requires the presence of a base in order to neutralize the hydrochloric acid formed as a byproduct, generating one equivalent of salt per equivalent of silacycle.^41,42^

**Chart 2:**
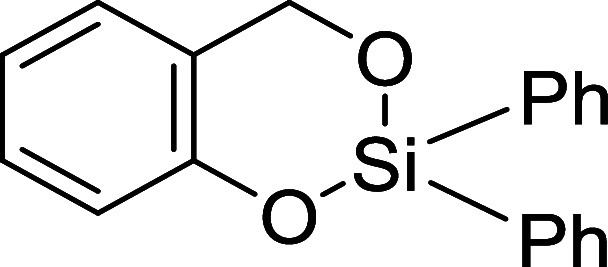
2,2-Diphenyl-4*H*-1,3,2-benzodioxasiline

The optimization of the tandem reaction was conducted using 2-hydroxy-4-methoxybenzaldehyde as model substrate (Table 3). Blank experiments confirmed that there is no reaction between diphenylsilane and this salicyladehyde in benzene-*d*_6_ at 80 °C in the absence of catalyst (entry 1). The benchmark reaction with 5 mol % of **1** at 80 °C exhibited the conversion to 7-methoxy-2,2-diphenyl-4*H*-benzo­[*d*]­[1,3,2]­dioxasiline in excellent yield (>99%) within 35 min (entry 2). The influence of light on the reaction was studied, and no significant changes were observed when the reaction was run in the dark (entry 3). The participation of radical species in the process is also excluded since the presence of a radical scavenger (TEMPO) does not affect the outcome of the reaction (entry 4). Further experiments were carried out in order to optimize the catalyst loading. Using 2.5 mol % of **1** the reaction is complete in 40 min (entry 5), while with 1 mol % after 60 min only 72% of the salicylaldehyde is converted into the silacycle (entry 6).

**Table 3:**
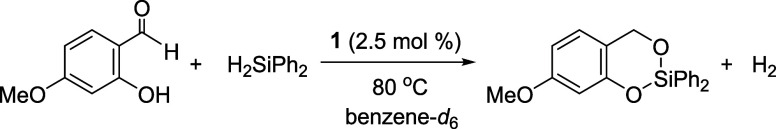
Tandem Hydrosilylation/Dehydrogenative Silylation of 2-Hydroxy-4-methoxybenzaldehyde Catalyzed by OsH_5_(SiHPh_2_)­(P^i^Pr_3_)_2_ (**1**)­^a^

**entry**	**variation from standard conditions**	**time (min)**	**conversion** ^a^
1	without catalyst	240	
2	5 mol % of **1**	35	>99
3	absence of light, 5 mol % of **1**	35	>99
4	presence of TEMPO (5%), 5 mol % of **1**	35	>99
5	none	40	>99
6	1 mol % of **1**	60	72

a Determined by ^1^H NMR of the crude reaction mixture using 1,4-dioxane as internal standard.

With the optimal conditions in hand (2.5 mol % **1**, 80 °C), we investigated the scope of this tandem hydrosilylation/dehydrogenative silylation of salicylaldehydes with diphenylsilane, and the results are summarized in Scheme 8. The reaction is applicable to various salicylaldehydes bearing different substituents on the phenyl ring, and the conversions to the corresponding 2,2-diphenyl-4*H*-1,3,2-benzodioxasilines are in all cases almost quantitative in times ranging from 35 to 140 min. The presence of an electron donating group (OMe, ^t^Bu) in position 4 of the aromatic ring leads to slightly shorter reaction times compared to that without substituent (40 and 35 min compared to 50 min). The beneficial effect of electron donating groups is also observed for those salicylaldehydes with a methyl or a methoxy moiety in position 5 (Me, OMe) that are converted in the corresponding silacycles **8d** and **8e** in 45 and 40 min, respectively, while the reactions with salicylaldehydes bearing electron withdrawing groups such as F and C­(O)­OMe in that position take longer times, 85 (**8f**) and 140 min (**8g**), respectively. Methylphenylsilane is also a suitable Si–H source for this process. As a proof of concept, under the same experimental conditions, salicylaldehyde reacts with this silane to give quantitatively the silacycle after 65 min (isolated yield 67%).

**Scheme 8:**
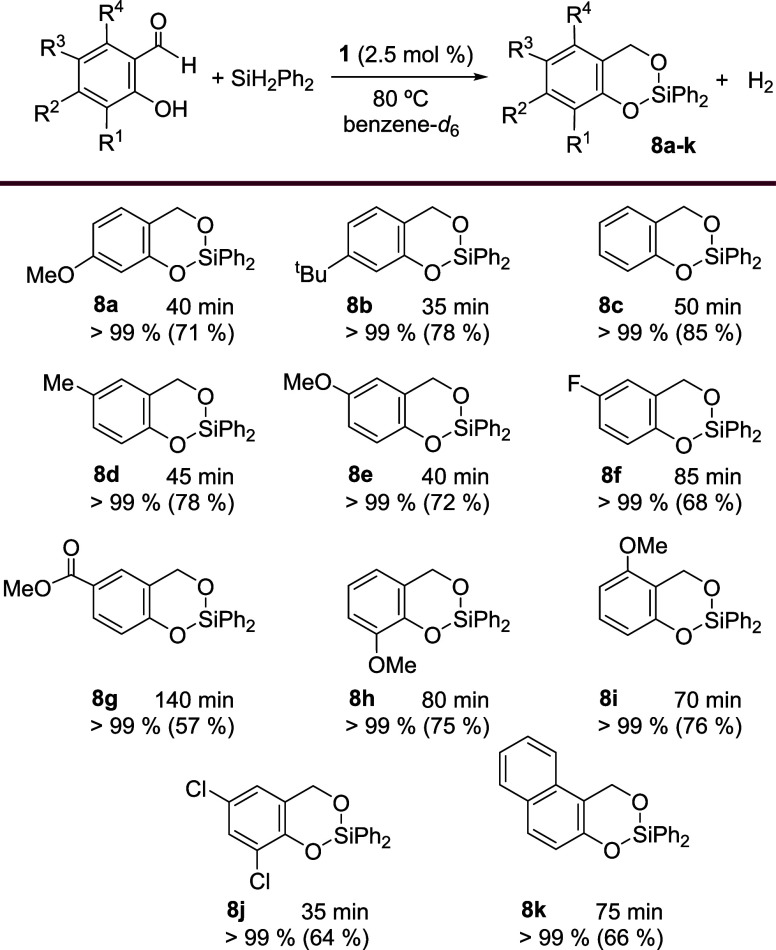
Tandem Dehydrogenative Silylation/Hydrosilylation of Salicyladehydes with Diphenylsilane^a^

a Conditions: **1** (7.1 × 10^–3^ mmol), H_2_SiPh_2_ (0.285 mmol), salicylaldehyde (0.285 mmol), and benzene-*d*_6_ (0.4 mL), 80 °C. The conversion yields were determined by ^1^H NMR of the crude reaction mixture using 1,4-dioxane as internal standard; isolated yields are given in parentheses.

### Mechanism of the Tandem Hydrosilylation/Dehydrogenative Silylation of Salicylaldehydes

In order to get insight into this tandem process we carried out DFT (B3LYP-D3/SDD­(f)-631g**) calculations using diphenylsilane and 2-hydroxy-5-methylbenzaldehyde as reagent models. The Δ*G* values were calculated in benzene (SMD) at 298.15 K and 1 atm. The first question that arises is which reaction takes place first, i.e., the dehydrogenative silylation of the OH group or the hydrosilylation of the aldehyde moiety? To answer this question both pathways have been computed. The dehydrogenative silylation of the OH group of 2-hydroxy-5-methyl-benzaldehyde occurs via a mechanism similar to the one described for phenol (*vide supra*). It starts from pentahydride **1** and 2-hydroxy-5-methylbenzaldehyde, which through a transition state related to **TS**_**1‑A**_ and located 36.3 kcal mol^–1^ above **1** (Figure S3), give rise to intermediate OsH_4_(η^2^-H_2_)­(P^i^Pr_3_)_3_ (**A**) and 2-[(diphenylsilyl)­oxy]-4-methyl-benzaldehyde. On the other hand, the energetically more feasible pathway for the hydrosilylation of the CHO group resembles that already computed for the hydrosilylation of benzaldehyde with diphenylsilane catalyzed by **1**.^28^ Thus, the tautomer of **1**, OsH_3_(η^2^-H_2_)­(SiHPh_2_)­(P^i^Pr_3_)_2_ (**B**), via transition state **TS**_**B–C**_ located 26.0 kcal mol^–1^ above **1** (Figure 7), hydrogenates through and outer-sphere mechanism the C=O double bond of the salicylaldehyde affording 2-(hydroxymethyl)-4-methylphenol and the silylene intermediate OsH_4_(=SiPh_2_)­(P^i^Pr_3_)_2_ (**C**). The comparison of these two processes shows that the least energetically demanding reaction is by far the hydrosilylation of the aldehyde (26.0 vs 36.3 kcal mol^–1^), indicating that within this tandem reaction the hydrosilylation takes place before the silylative dehydrogenation. Once the diol is formed, it enters in the cycle and, depending on which OH group of the diol coordinates to the silicon atom of the silylene ligand of intermediate **C**, two pathways can be anticipated (CH_2_OH: pathway a (blue); PhOH: pathway b (red); Figure 7). Both pathways share the same steps, similar to those previously reported for the hydrosilylation of benzaldehyde,^28^ although with different energy values. Thus, once one of the oxygen atoms coordinates to the silicon atom of the silylene (intermediates **D** ^**a**^ and **D** ^**b**^), the hydrogen atom of this alcohol is transferred to one of the hydrides, affording trihydride-(dihydrogen) oxygen-functionalized silyl derivatives **E** ^**a**^ and **E** ^**b**^, that isomerize to their pentahydride tautomers **F** ^**a**^ and **F** ^**b**^. Then, the intramolecular front-side nucleophilic attack of the remaining OH function of the O-substituent of the silyl group to the silicon atom of η^1^-coordinated silanes takes place. This attack occurs through transition states **TS** ^**a**^_**F‑A**_ and **TS** ^**b**^_**G‑A**_, respectively, in which the concerted and synchronous Si–H bond cleavage, Si–O bond formation, and Os­(η^2^-H_2_) formation take place. These transition states are similar to **TS**_**1‑A**_ (Figure 3b) and lead to the silacycle and to OsH_4_(η^2^-H_2_)­(P^i^Pr_3_)_2_ (**A**) directly (pathway a) or via η^1^-silane intermediate **G** ^**b**^. The reaction of **A** with diphenylsilane would release molecular hydrogen and regenerate complex **1**.

**Figure 7:**
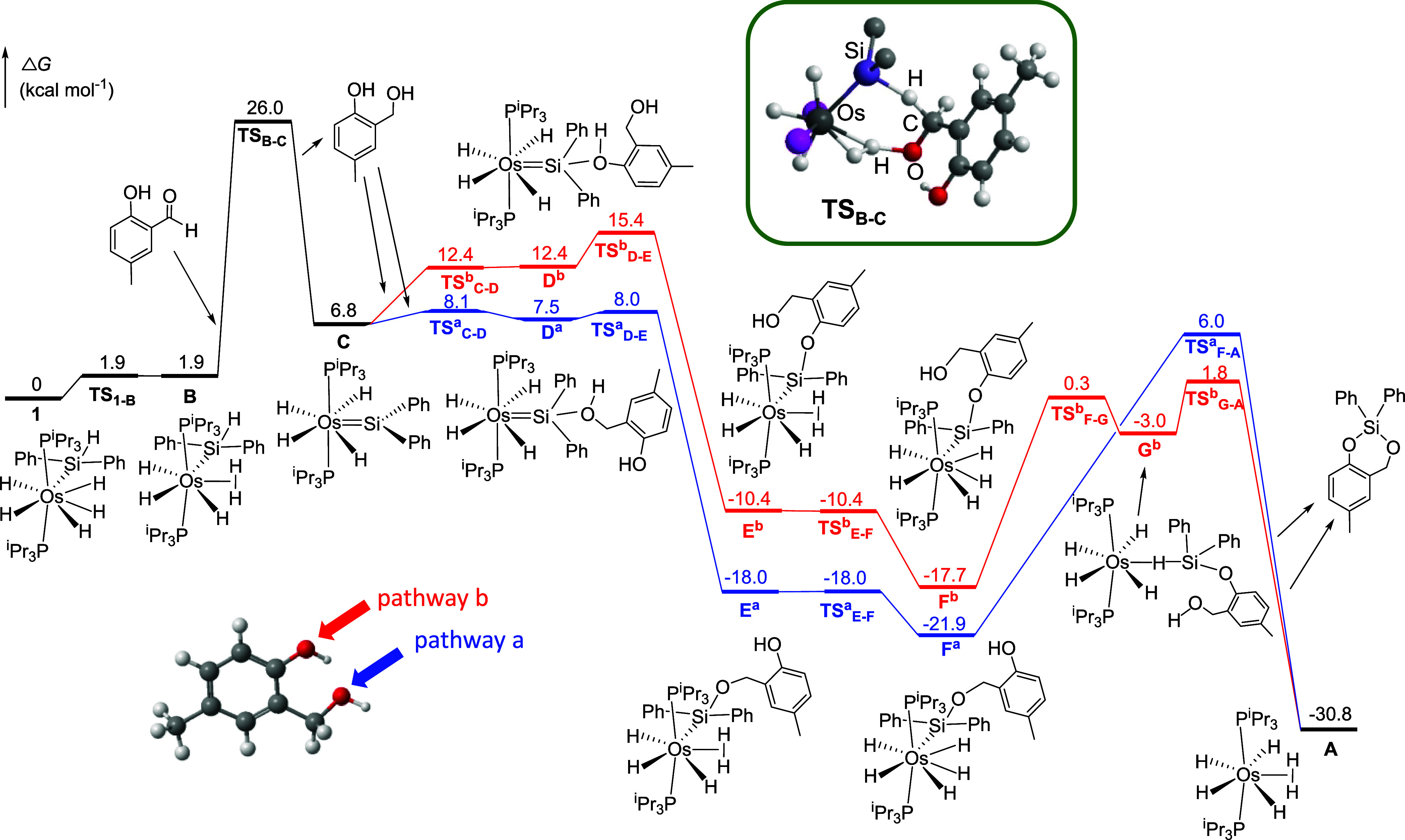
Computed energy profiles (Δ*G* in kcal mol^–1^) for the tandem hydrosilylation/dehydrogenative silylation of 2-hydroxy-5-methyl-benzaldehyde with diphenylsilane (CH_2_OH: pathway a (blue); PhOH: pathway b (red)). Inset: transition state for the hydrogenation of the aldehyde moiety of 2-hydroxy-5-methyl-benzaldehyde through an outer-sphere hydrogen transfer. Silyl and phosphine substituents are omitted for clarity.

Analysis of these reaction profiles shows that the two most energetically demanding steps are those that involve the formation of the Si–O bonds at the beginning (26.0 kcal mol^–1^) and at the end of the reaction (pathway a: 27.9 kcal mol^–1^; pathway b: 19.5 kcal mol^–1^). In the most favorable overall reaction pathway, i.e hydrosilylation of the aldehyde moiety followed by the dehydrogenative silylation of the Ph–OH function (pathway b), the rate-limiting step is the hydrogenation of the aldehyde, which is consistent with the fact that no reaction intermediates could be observed during the catalytic process.

## Concluding Remarks

This study shows that the pentahydride-silyl complex OsH_5_(SiHPh_2_)­(P^i^Pr_3_)_2_ is an efficient, although moderately active, catalyst precursor for the silylative dehydrogenation of alcohols with diphenylsilane to afford silyl ethers and molecular hydrogen. The DFT calculations suggest that the reactions take place via the nucleophilic attack of the alcohol to the silicon atom of the η^1^-coordinated silane of intermediate OsH_4_(η^1^-H–SiHPh_2_)­(P^i^Pr_3_)_2_, that provides a highly ordered transition state in which concerted and synchronous Si–H bond cleavage, Si–O bond formation, and Os-(η^2^-H_2_) formation take place, giving the monoalcoholysis product, HSi­(OR)­Ph_2_, and intermediate OsH_4_(η^2^-H_2_)­(P^i^Pr_3_)_3_, which reacts with diphenylsilane releasing hydrogen and regenerating the pentahydride-silyl derivative.

The stoichiometric alcoholysis and aminolysis of the Si–H bonds of OsH_5_(SiHPh_2_)­(P^i^Pr_3_)_2_ and OsH_4_(SiH_2_Ph)_2_(P^i^Pr_3_)_2_ with 2-hydroxypyridine and 2-aminopyridine leads to trihydride derivatives OsH_3_{κ^2^-*Si,N*-(SiPh_2_-E-py)}­(P^i^Pr_3_)_2_ and OsH_3_{κ^2^-*Si,N*-(SiPh­(Epy)-E-py)}­(P^i^Pr_3_)_2_ (E = O, NH), respectively. These reactions set up a new methodology for the synthesis of transition-metal complexes containing monoanionic bidentate κ^2^-*N*,*Si* pyridine-silyl ligands. The structural parameters, as well as the ^29^Si NMR chemical shifts of these derivatives, suggest a minor contribution of a base-silylene resonance form to better describe their Os–Si bonds. This has been confirmed by means of NBO and AIM analyses.

The ability of OsH_5_(SiHPh_2_)­(P^i^Pr_3_)_2_ to catalyze both the hydrosilylation of carbonyl derivatives, as well as the monoalcoholysis of diphenylsilane has allowed to develop an osmium-based tandem hydrosilylation/dehydrogenative silylation process of salicylaldehydes with diphenylsilane to afford 2,2-diphenyl-4*H*-1,3,2-benzodioxasiline type molecules. The DFT calculations suggest that the first step in this process is the outer-sphere hydrogenation of the aldehyde moiety of the salicylaldehydes to give the corresponding diol and tetrahydride-silylene OsH_4_(=SiPh_2_)­(P^i^Pr_3_)_2_. Next, two parallel pathways are plausible, depending on which oxygen atom of the two OH groups of the diol coordinates to the electrophilic silicon atom of the silylene. It was found that the silylative dehydrogenation of the Ph–OH function to afford a pentahydride silyl-O-functionalized, followed by the nucleophilic front-side intramolecular attack of the benzylic OH group to the silicon atom of an η^1^-coordinated silane gives the silacycle and intermediate OsH_4_(η^2^-H_2_)­(P^i^Pr_3_)_2_, which further reacts with diphenylsilane to release molecular hydrogen and regenerate OsH_5_(SiHPh_2_)­(P^i^Pr_3_)_2_.

In summary, while the activity of the pentahydride-silyl OsH_5_(SiHPh_2_)­(P^i^Pr_3_)_2_ in the silylative dehydrogenation of alcohols is not particularly noteworthly, its ability to catalyze the tandem hydrosilylation/dehydrogenative silylation of salicylaldehydes is remarkable. Other secondary silanes, such as methylphenylsilane, can be used as well in both processes, which expand the portfolio of reactions catalyzed by polyhydride complexes, highlighting their versatility.

## Experimental Section

### General

All reactions were carried out with exclusion of air using Schlenk-tube techniques or in a glovebox. No uncommon hazards are noted. Instrumental methods and X-ray details are given in the Supporting Information. In the NMR spectra (Figures S5–S105) the chemical shifts (in ppm) are referenced to residual solvent peaks (^1^H, ^13^C­{^1^H}-apt), external 85% H_3_PO_4_ (^31^P­{^1^H}) or external SiMe_4_ (^29^Si­{^1^H}), while *J* and *N* (*N* = *J*_P–H_ + *J*_P’‑H_ for ^1^H and *N* = *J*_P–C_ + *J*_P’‑C_ for ^13^C­{^1^H}) are given in hertz. OsH_5_(SiHPh_2_)­(P^i^Pr_3_)_2_ (**1**) and OsH_4_(SiH_2_Ph)_2_(P^i^Pr_3_)_2_ (**2**) were prepared according to the reported procedure.^28^

### General Procedure for the Monoalcoholysis of Diphenylsilane

The progress of the reaction was monitored using a reactor equipped with a pressure transducer (Man on the Moon series X102 kit). The procedure employed was as follows: under an argon atmosphere, a solution of **1** (0.014 mmol) in 1 mL of toluene and diphenylsilane (53 μL, 0.28 mmol) were incorporated into the reactor, which was closed and placed in an oil bath at 80 °C. Then, the pressure was monitored until a stable value was reached and the reactor tared. Subsequently the alcohol (0.28 mmol) was injected through a septum cap. In the case of solid alcohols, the alcohol was added first and diphenylsilane was injected. This moment was considered the initial time of the catalysis. The reactions were followed by measuring the pressure of the evolved hydrogen as a function of the time. Once the hydrogen evolution stopped, the pressure was released and the solution was then passed through a short plug of silica gel to remove the catalyst. Removal of the solvent gave the silyl ethers, that were analyzed by ^1^H NMR spectroscopy. The same experimental procedure was used for the experiments where methylphenylsilane was used.

### Recycling Experiment

The procedure employed was as follows: under an argon atmosphere, a solution of **1** (0.014 mmol) in 0.5 mL of toluene and diphenylsilane (0.55 mL, 2.96 mmol) were incorporated into the reactor equipped with a pressured transducer, which was closed and placed in an oil bath at 80 °C. Then, the pressure was monitored until a stable value was reached and the reactor tared. Subsequently benzyl alcohol (29.7 μL, 0.256 mmol) was injected through a septum cap. This moment was considered the initial time of the catalysis. The reaction was followed by measuring the pressure of the evolved hydrogen as a function of the time. Once the hydrogen evolution stopped, the pressure in the reactor was released, benzyl alcohol (29.7 μL, 0.256 mmol) is added through the septum cap and the process was repeated 10 consecutive runs without significant loss of catalytic activity.

#### Reaction of OsH_5_(SiHPh_2_)­(P^i^Pr_3_)_2_ (**1**) with Phenol: Preparation of OsH_5_{Si­(OPh)­Ph_2_}­(P^i^Pr_3_)_2_ (**3**)

Phenol (23 mg, 0.244 mmol) was added to a solution of 0.232 mmol of **1** in toluene (3 mL). The resulting solution was heated at 80 °C for 5 h. Afterward, the solution was dried under vacuum leading a yellowish oil. Addition of methanol (2 mL) afforded a white solid that was washed at −78 °C with methanol (3 mL) and dried under vacuum. Yield: 131.5 mg (72%). Colorless single crystals suitable for X-ray diffraction analysis were grown by slow diffusion of methanol into a solution of **2** in toluene at −25 °C. Anal. Calcd for C_36_H_62_OOsP_2_Si (%): C, 54.65; H, 7.90. Found: C, 54.40; H, 7.69. HRMS (electrospray, *m*/*z*) calcd. for C_36_H_60_OsOP_2_Si [M – 2H]^+^: 813.3390; found: 813.3382. IR (cm^–1^): ν­(Os–H) 1902 (m), ν­(C=C) 1593 (m). ^1^H NMR (300 MHz, C_6_D_6_): δ 8.20 (m, 4H, CH-arom), 7.35 (m, 4H, CH-arom), 7.23 (m, 2H, CH-arom), 7.11 (m, 2H, CH OPh), 6.97 (m, 2H, CH OPh), 6.84 (m, 1H, CH OPh), 1.96 (m, 6H, PC*H*(CH_3_)_2_), 1.16 (dvt, ^3^ *J*_H–H_ = 7.0, *N* = 13.9, 36H, PCH­(C*H*_3_)_2_), −9.47 (t, ^2^ *J*_H–P_ = 9.4, 5H, OsH_5_). *T*_1_(min) (ms, OsH_5_, 300 MHz, toluene-*d*_8_, 233 K): 145 ± 5 ms (−9.60 ppm). ^13^C­{^1^H}-apt NMR (75.429 MHz, C6D6, 298 K): 158.1 (s, C_q_ Ph), 148.7 (s, Cq Ph), 136.4, 128.9, 127.7, 126.5, 121.5, 120.0 (s, CH Ph), 28.7 (vt, *N* = 29.6, P*C*H­(CH_3_)_2_), 19.8 (s, PCH­(*C*H_3_)_2_). ^31^P­{^1^H} NMR (121.49 MHz, C_6_D_6_, 298 K): δ 44.6 (s). ^29^Si­{^1^H} NMR (59.63 MHz, C_6_D_6_, 298 K): δ 18.2 (s).

#### Reaction of OsH_5_(SiHPh_2_)­(P^i^Pr_3_)_2_ (**1**) with 2-Hydroxypyridine: Preparation of OsH_3_{κ^2^-*Si,N*-(SiPh_2_-Opy)}­(P^i^Pr_3_)_2_ (**4**)

2-hydroxypyridine (22 mg, 0.232 mmol) was added to a solution of 0.193 mmol of **1** in toluene (3 mL). The resulting solution was heated at 80 °C for 4 h. Afterward, the solution was dried under vacuum leading a yellowish residue. Addition of methanol (2 mL) afforded a white solid that was washed at −78 °C with methanol (3 mL) and dried under vacuum. Yield: 119 mg (78%). Colorless single crystals suitable for X-ray diffraction analysis were grown by slow diffusion of methanol into a solution of **3** in toluene at −25 °C. Anal. Calcd for C_35_H_59_NOOsP_2_Si (%): C, 53.20; H, 7.52; N, 1.77. Found: C, 53.33; H, 7.99; N, 1.68. HRMS (electrospray, *m*/*z*) calcd. for C_35_H_59_KNOOsP_2_Si [M + K]^+^: 830.3086; found: 830.3045. IR (cm^–1^): ν­(Os–H) 2082 (w), 1953 (m); ν­(C=C) 1605 (m). ^1^H NMR (300 MHz, C_6_D_6_, 298 K): δ 8.98 (dd, ^3^ *J*_H–H_ = 6.0, ^4^ *J*_H–H_ = 1.6, 1H, py), 8.02 (dd, ^3^ *J*_H–H_ = 8.0, ^4^ *J*_H–H_ = 1.3, 4H, *o*-Ph), 7.21 (m, 4H, *m*-Ph), 7.02 (tt, ^3^ *J*_H–H_ = 7.4, ^4^ *J*_H–H_ = 1.3, 2H, *p*-Ph), 6.97 (ddd, ^3^ *J*_H–H_ = 8.2, ^4^ *J*_H–H_ = 1.6, ^5^ *J*_H–H_ = 0.6, 1H, py), 6.89 (ddd, ^3^ *J*_H–H_ = 8.2, ^4^ *J*_H–H_ = 6.8, ^5^ *J*_H–H_ = 1.8, 1H, py), 5.89–5.85 (m, 1H, py), 1.72 (m, 6H, PC*H*(CH_3_)_2_), 1.06 (dvt, ^3^ *J*_H–H_ = 7.0, *N* = 13.5, 18H, PCH­(C*H*_3_)_2_), 0.96 (dvt, ^3^ *J*_H–H_ = 6.9, *N* = 11.5, 18H, PCH­(C*H*_3_)_2_), −4.32 (t, ^2^ *J*_H–P_ = 23.2, 1H, Os–H), −12.69 (broad signal, 1H, Os–H), −14.13 (broad signal, 1H, Os–H). ^1^H NMR (300 MHz, toluene-*d*_8_, 243 K, high field region): δ −4.48 (t, ^2^ *J*_H–P_ = 23.5, 1H, Os–H), −12.74 (dt, ^2^ *J*_H–P_ = 6.0, ^2^ *J*_H–H_ = 5.4, 1H, Os–H), −14.11 (dt, ^2^ *J*_H–P_ = 18.6, ^2^ *J*_H–H_ = 5.4, 1H, Os–H). *T*_1_(min) (ms, OsH_3_, 300 MHz, toluene-*d*_8_, 243 K): 170 ± 5 ms (−4.47 ppm), 141 ± 5 ms (−12.78 ppm), 109 ± 5 ms (−14.12 ppm). ^13^C­{^1^H}-apt NMR (75.429 MHz, C_6_D_6_, 298 K): 169.5 (s, C_q_ py), 159.2 (s, CH, py), 152.0 (s, Cq Ph), 136.8 (s, CH, py), 132.7, 127.0, 126.5 (s, CH Ph), 115.8, 111.0 (s, CH, py), 25.8 (vt, *N* = 24.4, P*C*H­(CH_3_)_2_), 21.3, 18.9 (both s, PCH­(*C*H_3_)_2_). ^31^P­{^1^H} NMR (121.49 MHz, C_6_D_6_, 298 K): δ 29.9 (s). ^29^Si­{^1^H} NMR (59.63 MHz, C_6_D_6_, 298 K): δ 55.9 (t, ^2^ *J*_Si–P_ = 6.4).

#### Reaction of OsH_5_(SiHPh_2_)­(P^i^Pr_3_)_2_ (**1**) with 2-Aminopyridine: Preparation of OsH_3_{κ^2^-*Si,N*-(SiPh_2_-NH-py)}­(P^i^Pr_3_)_2_ (**5**)

2-aminopyridine (22 mg, 0.232 mmol) was added to a solution of 0.193 mmol of OsH_5_(SiHPh_2_)­(P^i^Pr_3_)_2_ in toluene (3 mL). The resulting solution was heated at 80 °C for 4 h. Afterward, it was dried under vacuum leading a yellowish residue. Methanol (3 mL) was added and the resulting solution was stored at −20 °C overnight, obtaining a white precipitate. The supernatant solution was removed and the white solid was dried under vacuum. Yield: 100.6 mg (65%). Anal. Calcd for C_35_H_60_N_2_OsP_2_Si (%): C, 53.27; H, 7.66; N, 3.55. Found; C, 53.56; H, 8.08; N, 3.78. HRMS (electrospray, *m*/*z*) calcd. for C_35_H_58_N_2_OsP_2_Si [M – 2H]^+^: 788.3454; found: 788.3463. IR (cm^–1^): ν­(NH) 3402 (w), ν­(Os–H) 2087 (w), 1971 (w), ν­(C=C) 1605 (m). ^1^H NMR (300 MHz, C_6_D_6_, 298 K): δ 8.93 (d, ^3^ *J*_H–H_ = 5.8, 1H, py), 7.85 (dd, ^3^ *J*_H–H_ = 8.0, ^4^ *J*_H–H_ = 1.3, 4H, *o*-Ph), 7.24 (t, ^3^ *J*_H–H_ = 7.5, 4H, *m*-Ph), 7.08 (tt, ^3^ *J*_H–H_ = 7.4, ^4^ *J*_H–H_ = 1.3, 2H, *p*-Ph), 6.81–6.78 (m, 1H, py), 6.25 (d, ^3^ *J*_H–H_ = 8.3, 1H, py), 5.75–5.71 (m, 1H, py), 5.59 (s, 1H, NH), 1.76–1.65 (m, 6H, PC*H*(CH_3_)_2_), 1.07 (dvt, ^3^ *J*_H–H_ = 7.3, *N* = 13.3, 18H, PCH­(C*H*_3_)_2_), 0.99 (dvt, ^3^ *J*_H–H_ = 6.7, *N* = 11.8, 18H, PCH­(C*H*_3_)_2_), −4.24 (t, ^2^ *J*_H–P_ = 24.0, 1H, OsH), −12.94 (br, 1H, OsH), −14.48 (br, 1H, OsH). ^1^H NMR (300 MHz, toluene-*d*_8_, 243 K, high field region): δ −4.42 (t, ^2^ *J*_H–P_ = 23.6, 1H, OsH), −12.98 (dt, ^2^ *J*_H–H_ = 9.6, ^2^ *J*_H–P_ = 6.5, 1H, OsH), −14.51 (dt, ^2^ *J*_H–H_ = 9.6, ^2^ *J*_H–P_ = 19.8, 1H, OsH). *T*_1_(min) (ms, OsH_3_, 300 MHz, toluene-*d*_8_, 233 K): 147 ± 5 ms (−4.25 ppm), 123 ± 5 ms (−12.94 ppm), 99 ± 5 ms (−14.47 ppm). ^13^C­{^1^H}-apt NMR (75.429 MHz, C_6_D_6_, 298 K): δ 167.2 (s, C_q_, py), 160.8 (s, CH, py) 151.9 (s, C_q_, Ph), 134.8 (s, CH, py), 133.5, 126.7, 126.5 (all s, CH Ph), 112.3 (s, CH, py), 109.2 (s, CH, py), 25.8 (vt, *N* = 24.2, P*C*H­(CH_3_)_2_), 21.3, 18.9 (both s, PCH­(*C*H_3_)_2_). ^31^P­{^1^H} NMR (121.49 MHz, C_6_D_6_, 298 K): δ 29.2 (s). ^29^Si­{^1^H} NMR (59.63 MHz, C_6_D_6_, 298 K): δ 30.3 (t, ^2^ *J*_Si–P_ = 6.6).

#### Reaction of OsH_4_(SiH_2_Ph)_2_(P^i^Pr_3_)_2_ (**2**) with 2-Hydroxypyridine: Preparation of OsH_3_{κ^2^-*Si,N*-(SiPh­(Opy)-O-py)}­(P^i^Pr_3_)_2_ (**6**)

2-hydroxypyridine (20 mg, 0.21 mmol) was added to a solution of **2** (70 mg, 0.096 mmol) in toluene (2 mL). The resulting solution was heated at 80 °C for 4 h. Afterward, the solution was dried under vacuum leading a yellowish oil. Addition of methanol (1 mL) afforded a white precipitate, that was washed with further portions of methanol (2 × 2 mL) and dried under vacuum. Yield: 44 mg (57%). Colorless single crystals suitable for X-ray diffraction analysis were grown by slow diffusion of methanol into a solution of **6** in toluene at −25 °C. Anal. Calcd for C_34_H_58_N_2_O_2_OsP_2_Si (%): C, 50.60; H, 7.24; N, 3.47. Found: C, 50.45; H, 7.51; N, 3.84. HRMS (electrospray, *m*/*z*) calcd. for C_34_H_58_N_2_O_2_OsP_2_Si [M]^+^: 807.3274; found: 807.3237. IR (cm^–1^): ν­(Os–H) 2119 (w), 1984 (w). ^1^H NMR (300 MHz, C_6_D_6_, 298 K): δ 9.08 (dd, ^3^ *J*_H–H_ = 5.8, ^4^ *J*_H–H_ = 1.6, 1H, py), 8.02 (m, 2H, Ph), 7.22 (m, 2H, Ph), 7.06 (tt, ^3^ *J*_H–H_ = 7.4, ^4^ *J*_H–H_ = 1.3, 1H, Ph), 7.00–6.81 (5H, py), 6.26 (ddd, ^3^ *J*_H–H_ = 7.0, ^4^ *J*_H–H_ = 5.0, ^5^ *J*_H–H_ = 1.2, 1H, py), 5.87 (ddd, ^3^ *J*_H–H_ = 6.9, ^4^ *J*_H–H_ = 6.0, ^5^ *J*_H–H_ = 1.6, 1H, py), 2.39 (m, 3H, PC*H*(CH_3_)_2_), 1.63 (m, 3H, PC*H*(CH_3_)_2_), 1.32 (dvt, ^3^ *J*_H–H_ = 6.8, *N* = 13.5, 9H, PCH­(C*H*_3_)_2_), 1.18 (dvt, ^3^ *J*_H–H_ = 6.8, *N* = 13.5, 9H, PCH­(C*H*_3_)_2_), 0.97 (dvt, ^3^ *J*_H–H_ = 7.2, *N* = 13.5, 9H, PCH­(C*H*_3_)_2_), 0.92 (dvt, ^3^ *J*_H–H_ = 7.2, *N* = 13.5, 9H, PCH­(C*H*_3_)_2_, −3.77 (t, ^2^ *J*_H–P_ = 23.2, 1H, Os–H), −12.58 (broad signal, 1H, Os–H), −13.88 (broad signal, 1H, Os–H). ^1^H NMR (300 MHz, toluene-*d*_8_, 243 K, high field region): δ −3.89 (t, ^2^ *J*_H–P_ = 22.0, 1H, Os–H), −12.60 (dt, ^2^ *J*_H–P_ = 8.1, ^2^ *J*_H–H_ = 7.2, 1H, Os–H), −13.88 (dt, ^2^ *J*_H–P_ = 18.5, ^2^ *J*_H–H_ = 8.1, 1H, Os–H). *T*_1_(min) (ms, OsH_3_, 300 MHz, toluene-*d*_8_, 243 K): 180 ± 5 ms (−3.89 ppm), 148 ± 5 ms (−12.60 ppm), 119 ± 5 ms (−13.88 ppm). ^13^C­{^1^H}-apt NMR (75.429 MHz, C6D6, 298 K): δ 168.6 (s, Cq py), 163.1 (s, Cq py), 159.6 (s, CH py), 148.3 (s, CH Py), 147.1 (s, C_q_ Ph), 137.6, 137.0 (s, CH py), 133.8, 128.1, 126.6 (s, Ph), 116.2, 116.3, 113.9, 110.9 (s, CH py), 26.3 (dd, *J*_C–P_ = 13.7, *J*_C–P_ = 11.6, P*C*H­(CH_3_)_2_), 25.3 (dd, *J*_C–P_ = 13.9, *J*_C–P_ = 11.3, P*C*H­(CH_3_)_2_), 21.4, 21.0, 19.2, 18.9 (all s, PCH­(*C*H_3_)_2_). ^31^P­{^1^H} NMR (121.49 MHz, C_6_D_6_, 298 K): δ 32.6 (nonresolved AB spin system). ^31^P­{^1^H} NMR (121.49 MHz, toluene-*d*_8_, 253 K): AB spin system centered at 32.6 ppm (*J*_A‑B_ = 219 Hz, Δν = 119.3 Hz). ^1^H,^29^Si HMBC NMR (300 MHz, C_6_D_6_, 298 K): δ­(^29^Si) 59.6.

#### Reaction of OsH_4_(SiH_2_Ph)_2_(P^i^Pr_3_)_2_ (**2**) with 2-Aminopyridine: Preparation of OsH_3_{κ^2^-*Si,N*-(SiPh­(NHpy)-NH-py)}­(P^i^Pr_3_)_2_ (**7**)

2-aminopyridine (20 mg, 0.21 mmol) was added to a solution of **2** (70 mg, 0.096 mmol) in toluene (2 mL). The resulting solution was heated at 80 °C for 4 h. Afterward, the solution was dried under vacuum leading a yellowish residue. Addition of methanol (2 mL) afforded a white solid that was washed with further portions of cold methanol (3 × 2 mL) and dried under vacuum. Yield: 58 mg (75%). Anal. Calcd for C_34_H_60_N_4_OsP_2_Si (%): C, 50.72; H, 7.51; N, 6.96. Found: C, 50.48; H, 7.93; N, 7.28. HRMS (electrospray, *m*/*z*) calcd. for C_34_H_59_N_4_OsP_2_Si [M – H]^+^: 805.3594; found: 805.3601. ^1^H NMR (300 MHz, C_6_D_6_, 298 K): δ 9.05 (d, ^3^ *J*_H–H_ = 5.6, 1H, py), 8.17 (m, 2H, Ph), 7.39 (m, 2H, Ph), 7.28–7.23 (m, 1H, Ph), 7.06–7.01 (2H, py), 6.83 (m, 1H, py), 6.31–6.20 (m, 3H, py), 5.82 (m, 1H, py), 5.54 (s, 1H, NH), 2.19 (m, 3H, PC*H*(CH_3_)_2_), 1.80 (m, 3H, PC*H*(CH_3_)_2_), 1.38 (dvt, ^3^ *J*_H–H_ = 7.0, *N* = 13.8, 9H, PCH­(C*H*_3_)_2_) 1.25 (dvt, ^3^ *J*_H–H_ = 6.9, *N* = 11.5, 9H, PCH­(C*H*_3_)_2_), 1.12–1.06 (18H, PCH­(C*H*_3_)_2_), −3.93 (t, ^2^ *J*_H–P_ = 23.9, 1H, Os–H), −12.88 (broad signal, 1H, Os–H), −14.66 (broad signal, 1H, Os–H). ^1^H NMR (300 MHz, toluene-*d*_8_, 243 K high field region): δ −4.17 (t,^2^ *J*_H–P_ = 24.4, 1H, Os–H), −12.96 (dt, ^2^ *J*_H–H_ = 9.3, ^2^ *J*_H–P_ = 6.9, 1H, Os–H), −14.80 (dt, ^2^ *J*_H–H_ = 9.3, ^2^ *J*_H–P_ = 18.0, 1H, Os–H). *T*_1_(min) (ms, OsH_3_, 300 MHz, toluene-*d*_8_, 233 K): 162 ± 5 ms (−3.93 ppm), 115 ± 5 ms (−12.88 ppm), 103 ± 5 ms (−14.66 ppm). ^13^C­{^1^H}-apt NMR (75.429 MHz, C_6_D_6_, 298 K): δ 166.9 (s, C_q_ py), 160.6 (s, C_q_ py), 160.4 (s, CH py), 150.1 (t, *J*_C–P_ = 1.7, C_q_ Ph), 148.4, 136.4, 134.9 (all s, CH py), 134.3, 127.2, 126.6 (all s, CH Ph), 112.2, 111.9, 110.0, 109.4 (all s, CH py), 26.1 (dd, *J*_C–P_ = 14.1, *J*_C–P_ = 13.9, P*C*H­(CH_3_)_2_), 24.7 (dd, *J*_C–P_ = 13.5, *J*_C–P_ = 13.6, P*C*H­(CH_3_)_2_), 21.6, 21.1, 19.0, 18.8 (all s, PCH­(*C*H_3_)_2_). ^31^P­{^1^H} NMR (121.49 MHz, C_6_D_6_, 298 K): δ 32.0, 31.9 (nonresolved AB spin system). ^31^P­{^1^H} NMR (121.49 MHz, toluene-*d*_8_, 298 K): AB spin system centered at 32.1 ppm (*J*_A‑B_ = 226 Hz, Δν = 137.4 Hz). ^29^Si­{^1^H} NMR (59.63 MHz, C_6_D_6_, 298 K): δ 31.8 (t, ^2^ *J*_Si–P_ = 6.1).

### General Procedure for the Tandem Dehydrogenative Silylation/Hydrosilylation Reactions of Salicylaldehydes with Diphenylsilane

A 17.83 mM stock solution of **1** was prepared in C_6_D_6_. To an NMR tube 0.4 mL of this solution, H_2_SiPh_2_ (53 μL, 0.285 mmol), the corresponding salicylaldehyde (0.285 mmol) and dioxane (3.1 μL, 0.0356 mmol, internal standard) were added. The reaction mixture was heated at 80 °C and the progress of the reaction was followed by ^1^H NMR spectroscopy. After its completion, the mixture was exposed to air, passed through a short plug of silica and eluted with toluene. Evaporation of the solution afforded the products as oils. The same procedure was used for the reaction of salicylaldehyde with methylphenylsilane.

## Supplementary Material





## Notes

General information for the Experimental Section, structural analysis, computational details, and NMR spectra (PDF)
Atomic coordinates of optimized molecules (XYZ)
