# Supplementary material for: Si–O Bond Formation Mediated by an Osmium-Polyhydride: Dehydrogenative Silylation of Alcohols and Tandem Hydrosilylation/Dehydrogenative Silylation of Salicylaldehydes
Source: Inorg Chem. 2026 May 5;65(19):10734–48. doi: 10.1021/acs.inorgchem.6c01007 (PMC13188063; doi:10.1021/acs.inorgchem.6c01007)
Supplement: Supplementary file 1 [file ic6c01007_si_001.pdf]

SUPPORTING INFORMATION

Si−O Bond Formation Mediated by an Osmium-Polyhydride: Dehydrogenative Silylation of Alcohols and Tandem Hydrosilylation/ Dehydrogenative Silylation of Salicylaldehydes

Iñigo V. Alli, Enrique Oñate, and Montserrat Oliván *

Departamento de Química Inorgánica - Instituto de Síntesis Química y Catálisis Homogénea (ISQCH) - Centro de Innovación en Química Avanzada (ORFEO-CINQA), Universidad de Zaragoza - CSIC, 50009 Zaragoza, Spain

* e-mail: molivan@unizar.es

Contents:

- Experimental Section: General Information	S2
- Characterization Data of the Products of Monoalcholysis of Diphenylsilane and Methylphenylsilane	S3
- Characterization Data of the Tandem Hydrosilylation/Dehydrogenative Silylation of Salicylaldehydes with Diphenylsilane and Methylphenylsilane	S6
- Structural Analysis of Complexes **3**, **4**, and **6**	S9
- Computational Details	S11
- NMR spectra	S15
- References	S66

• Experimental Section: General Information

All reactions were carried out with exclusion of air using Schlenk-tube techniques or in a drybox. Pentane and toluene were obtained oxygen- and water-free from an MBraun solvent purification apparatus, while methanol was distilled prior to use. Diphenylsilane and methylphenylsilane were used as received. ^{1}H, $^{13}C\{^{1}H\}$, $^{31}P\{^{1}H\}$, and $^{29}Si\{^{1}H\}$ NMR spectra were recorded on Bruker 300 ARX, Bruker Avance 300 MHz, Bruker Avance 400 MHz, or Bruker Avance 500 MHz instruments. Chemical shifts (expressed in ppm) are referenced to residual solvent peaks (^{1}H, $^{13}C\{^{1}H\}$), external 85% H_3PO_4 ($^{31}P\{^{1}H\}$), $SiMe_4$ ($^{29}Si\{^{1}H\}$), or external $CFCl_3$ ($^{19}F\{^{1}H\}$ NMR). Coupling constant J and N ($N = J_{P-H} + J_{P'-H}$ for ^{1}H and $N = J_{P-C} + J_{P'-C}$ for $^{13}C\{^{1}H\}$) are given in hertz. Attenuated total reflection infrared spectra (ATR-IR) of solid samples were run on a PerkinElmer Spectrum 100 FT-IR spectrometer. C, H, and N analyses were carried out in a PerkinElmer 2400 CHNS/O analyzer. High-resolution electrospray mass spectra were acquired using a MicroTOF-Q hybrid quadrupole time-of-flight spectrometer (Bruker Daltonics, Bremen, Germany). $OsH_5(SiHPh_2)(P^iPr_3)_2$ (**1**) and $OsH_4(SiH_2Ph)_2(P^iPr_3)_2$ (**2**) were prepared by the published method.[1]

• Characterization Data of the Products of Monoalcoholysis of Diphenylsilane and Methylphenylsilane

HSi(OPh)Ph$_2$.[2] ^{1}H NMR (300 MHz, CDCl$_3$, 298 K): δ 7.68 (dd, J_{H-H} = 7.6, J_{H-H} = 1.5, 4H, CH arom), 7.43-7.35 (7H, CH arom), 7.21-7.16 (m, 2H, CH arom), 6.93 (m, 2H, CH arom), 5.73 (s, 1H, SiH).

HSi(OPh-4-OMe)Ph$_2$. ^{1}H NMR (300 MHz, CDCl$_3$, 298 K): δ 7.74 (m, 4H, CH arom), 7.51-7.42 (6H, CH arom), 6.93 (m, 2H, CH arom), 6.79 (m, 2H, CH arom), 5.78 (s, 1H, SiH), 3.79 (s, 3H, OMe). ^{13}C{^{1}H}-apt NMR (75.429 MHz, CDCl$_3$, 298 K): δ 154.5 (s, C$_q$ arom), 149.2 (s, C$_q$ arom), 134.7 (s, CH arom), 133.1 (s, C$_q$ arom), 130.7, 128.2, 119.9, 114.6 (all s, CH arom), 55.6 (s, CH$_3$, OMe). ^{29}Si{^{1}H} NMR (59.63 MHz, CDCl$_3$, 298 K): δ –19.4 (s). GC-MS (*m/z*): 306.1 (M$^+$).

HSi(OPh-4-^{t}Bu)Ph$_2$. ^{1}H NMR (300 MHz, CDCl$_3$, 298 K): δ 7.77 (m, 4H, CH arom), 7.52-7.44 (6H, CH arom), 7.29 (m, 2H, CH arom), 6.69 (m, 2H, CH arom), 5.81 (s, 1H, SiH), 1.34 (s, 9H, ^{t}Bu). ^{13}C{^{1}H}-apt NMR (75.429 MHz, CDCl$_3$, 298 K): δ 153.2 (s, C$_q$ arom), 144.5 (s, C$_q$ arom), 134.8 (s, CH arom), 133.2 (s, C$_q$ arom), 130.7, 128.2, 124.4, 118.6 (all s, CH arom), 34.2 (s, *C*(CH$_3$)$_3$), 31.6 (s, C(*C*H$_3$)$_3$). ^{29}Si{^{1}H} NMR (59.63 MHz, CDCl$_3$, 298 K): δ –21.2 (s). GC-MS (*m/z*): 332.2 (M$^+$).

HSi(OPh-4-Cl)Ph$_2$. ^{1}H NMR (300 MHz, CDCl$_3$, 298 K): δ 7.70 (m, 4H, CH arom), 7.53-7.40 (6H, CH arom), 7.17 (m, 2H, CH arom), 6.89 (m, 2H, CH arom), 5.73 (s, 1H, SiH). ^{13}C{^{1}H}-apt NMR (75.429 MHz, CDCl$_3$, 298 K): δ 154.0 (s, C$_q$ arom), 134.7 (s, CH arom), 132.4 (s, C$_q$ arom), 130.9, 129.5, 128.3 (all s, CH arom), 126.9 (s, C$_q$ arom), 120.6 (s, CH arom). ^{29}Si{^{1}H} NMR (59.63 MHz, CDCl$_3$, 298 K): δ –19.0 (s). GC-MS (*m/z*): 310.1 (M$^+$).

HSi(OPh-4-CF$_3$)Ph$_2$. ^{1}H NMR (300 MHz, CDCl$_3$, 298 K): δ 7.91 (m, 4H, CH arom), 7.68-7.55 (8H, CH arom), 7.26 (d, $^3J_{H-H}$ = 8.1, 2H, CH arom), 6.02 (s, 1H, SiH). ^{13}C{^{1}H}-apt NMR (75.429 MHz, CDCl$_3$, 298 K): δ 158.4 (q, $^5J_{C-F}$ = 1.4 C$_q$ arom), 134.9 (s, CH arom), 132.3 (s, C$_q$ arom), 131.2, 128.5 (all s, CH arom), 127.2 (q, $^3J_{C-F}$ = 3.9, CH arom), 124.6 (q, $^1J_{C-F}$ = 271.6, C$_q$ arom), 124.3 (q, $^2J_{C-F}$ = 33.9 C$_q$ arom), 119.2 (s, CH arom). ^{29}Si{^{1}H} NMR (59.63 MHz, CDCl$_3$, 298 K): δ –13.1 (s). ^{19}F{^{1}H} NMR (282.33 MHz, CDCl$_3$, 298 K): δ –61.4 (s). GC-MS (*m/z*): 344.0 (M$^+$).

HSi(OCH$_2$Ph)Ph$_2$.[3] ^{1}H NMR (300 MHz, CDCl$_3$, 298 K): δ 7.62 (dd, J_{H-H} = 7.6, J_{H-H} = 1.5, 4H, CH arom), 7.41-7.28 (11H, CH arom), 5.48 (s, 1H, SiH), 4.83 (s, 2H, CH$_2$).

HSi(O^{n}Pr)Ph$_2$.[4] ^{1}H NMR (300 MHz, CDCl$_3$, 298 K): δ 7.62 (dd, J_{H-H} = 7.4, J_{H-H} = 1.7, 4H, CH arom), 7.42-7.31 (6H, CH arom), 5.41(s, 1H, SiH), 3.71 (t, $^3J_{H-H}$ = 6.8, 2H, CH$_2$), 1.61 (m, 2H, CH$_2$), 0.91 (t, $^3J_{H-H}$ = 7.3, 3H, CH$_3$).

HSi(O^{n}Bu)Ph$_2$.[2] ^{1}H NMR (300 MHz, CDCl$_3$, 298 K): δ 7.60 (dd, J_{H-H} = 6.8, J_{H-H} = 1.9, 4H, CH arom), 7.39-7.32 (6H, CH arom), 5.46 (s, 1H, SiH), 3.73 (m, 1H, CH), 1.52-1.37 (6H, CH$_2$), 0.88-0.78 (6H, CH$_3$).

HSi[OCH(Et)^{n}Pr]Ph$_2$.[5] ^{1}H NMR (300 MHz, CDCl$_3$, 298 K): δ 7.60 (dd, J_{H-H} = 6.8, J_{H-H} = 1.9, 4H, CH arom), 7.39-7.32 (6H, CH arom), 5.46 (s, 1H, SiH), 3.73 (m, 1H, CH), 1.52-1.37 (6H, CH$_2$), 0.88-0.78 (6H, CH$_3$).

HSi(OCy)Ph$_2$.[2] ^{1}H NMR (300 MHz, CDCl$_3$, 298 K): δ 7.61 (dd, J_{H-H} = 7.4, J_{H-H} = 1.7, 4H, CH arom), 7.39-7.31 (6H, CH arom), 5.45 (s, 1H, SiH), 3.72 (tt, J_{H-H} = 9.2, J_{H-H} = 3.9, 1H, CH Cy), 1.84-1.15 (m, 10H, CH$_2$ Cy).

SiH[OCH(Me)Cy)]Ph$_2$.[6] ^{1}H NMR (300 MHz, CDCl$_3$, 298 K): δ 7.61 (dd, J_{H-H} = 7.4, J_{H-H} = 1.7, 4H, CH arom), 7.39-7.33 (6H, CH arom), 5.43 (s, 1H, SiH), 3.72 (dq, J_{H-H} = 9.2, J_{H-H} = 3.9, 1H, CH), 1.87-0.93 (14H, Cy + CH$_3$). ^{13}C{^{1}H}-apt NMR (75.429 MHz, CDCl$_3$, 298 K): δ 135.1 (s, C$_q$ arom), 134.7, 130.2, 128.0 (all s, CH arom), 75.1 (s, CH-O), 45.4 (s, CH Cy), 28.9, 28.7, 26.7, 26.4, 26.4 (s, CH$_2$ Cy), 20.3 (s, CH$_3$). ^{29}Si{^{1}H} NMR (59.63 MHz, CDCl$_3$, 298 K): δ –14.1 (s). GC-MS (*m/z*): 310.5 (M$^+$).

HSi(O^{t}Bu)Ph$_2$.[2] ^{1}H NMR (300 MHz, CDCl$_3$, 298 K): δ 7.68 (dd, J_{H-H} = 7.7, J_{H-H} = 1.8, 4H, CH arom), 7.46-7.38 (6H, CH arom), 5.63 (s, 1H, SiH), 1.40 (s, 9H, CH$_3$).

HSi(OPh)MePh.[7] ^{1}H NMR (300 MHz, CDCl$_3$, 298 K): δ 7.75 (m, 2H, CH arom), 7.52-7.44 (4H, CH arom), 7.31-7.25 (m, 2H, CH arom), 7.05-6.96 (4H, CH arom), 5.42 (q, J_{H-H} = 2.9, 1H, SiH), 0.66 (d, J_{H-H} = 2.9, 3H, CH$_3$).

HSi(OCH$_2$Ph)MePh.[8] ^{1}H NMR (300 MHz, CDCl$_3$, 298 K): δ 7.72 (m, 2H, CH arom), 7.51-7.34 (8H, CH arom), 5.19 (q, J_{H-H} = 2.9, 1H, SiH), 4.85 (s, 2H, CH$_2$), 0.57 (d, J_{H-H} = 2.9, 3H, CH$_3$).

HSi(O^{t}Bu)MePh.[9] ^{1}H NMR (300 MHz, $CDCl_3$, 298 K): δ 7.64 (m, 2H, CH arom), 7.41-7.37 (3H, CH arom), 5.16 (q, $J_{H\text{-}H}$ = 2.9, 1H, SiH), 1.31 (s, 9H, CH_3), 0.44 (d, $J_{H\text{-}H}$ =2.9, 3H, CH_3).

• Characterization Data of the Tandem Hydrosilylation/Dehydrogenative Silylation of Salicylaldehydes with Diphenylsilane and Methylphenylsilane

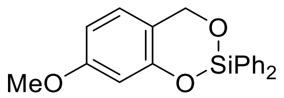

8a. ^{1}H NMR (300 MHz, $CDCl_3$, 298 K): δ 7.78-7.75 (m, 4H, Ph), 7.56-7.42 (6H, Ph), 6.96 (d, 1H, $^3J_{H\text{-}H}$ = 8.3, CH arom), 6.66 (d, $^4J_{H\text{-}H}$ = 2.5, 1H, CH arom), 6.56 (dd, $^3J_{H\text{-}H}$ = 8.3, $^4J_{H\text{-}H}$ = 2.5, 1H, CH arom), 5.10 (s, 2H, CH_2), 3.82 (s, 3H, OCH_3). $^{13}C\{^1H\}$-apt NMR (75.429 MHz, $CDCl_3$, 298 K): δ 160.5 (s, C_q arom), 154.2 (s, C_q arom) 134.8 (s, CH arom), 131.4 (s, C_q arom), 131.2, 128.1, 126.7, (all s, CH arom), 119.4 (s, C_q arom), 107.5, 105.0 (both s, CH arom), 64.3 (s, CH_2), 55.3 (s, OCH_3). $^{29}Si\{^1H\}$ NMR (59.63 MHz, $CDCl_3$, 298 K): δ –32.7 (s). GC-MS (*m/z*): 334.1 (M^+).

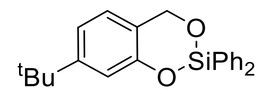

8b. ^{1}H NMR (300 MHz, $CDCl_3$, 298 K): δ 7.96-7.93 (m, 4H, Ph), 7.64-7.54 (6H, Ph), 7.30 (m, 1H, CH arom), 6.81 (2H, CH arom), 5.29 (s, 2H, CH_2), 1.50 (s, 9H, CH_3). $^{13}C\{^1H\}$-apt NMR (75.429 MHz, $CDCl_3$, 298 K): δ 153.1, 152.8 (both s, C_q arom) 135.1 (s, CH arom), 131.7 (s, C_q arom), 131.3, 128.2, 125.9 (all s, CH arom), 124.0 (s, C_q arom), 118.5, 116.8 (both s, CH arom), 64.5 (s, CH_2), 34.8 (s, C_q ^{t}Bu), 31.5 (s, CH_3 ^{t}Bu). $^{29}Si\{^1H\}$ NMR (59.63 MHz, $CDCl_3$, 298 K): δ –30.2 (s). GC-MS (*m/z*): 360.2 (M^+).

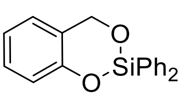

8c.[10] ^{1}H NMR (300 MHz, $CDCl_3$, 298 K): δ 7.80-7.77 (m, 4H, CH arom), 7.57-7.43 (6H, CH-rom), 7.30-7.25 (m, 1H, CH arom), 7.11-7.06 (m, 2H, CH arom), 7.00 (td, $J_{H\text{-}H}$ = 7.4, $J_{H\text{-}H}$ = 1.2, 1H, CH arom), 5.17 (s, 2H, CH_2). ^{29}Si $\{^1H\}$ NMR (59.63 MHz, $CDCl_3$, 298 K): δ –26.8 (s).

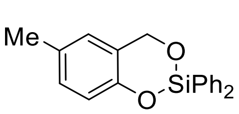

8d. ^{1}H NMR (300 MHz, $CDCl_3$, 298 K): δ 7.91-7.88 (m, 4H, Ph), 7.63-7.51 (6H, Ph), 7.18-7.09 (2H, CH arom), 6.96 (m, 1H, CH arom), 5.23 (s, 2H, CH_2), 2.42 (s, 3H, CH_3). $^{13}C\{^1H\}$-apt NMR (75.429 MHz, $CDCl_3$, 298 K): δ 151.3 (s, C_q arom), 135.0 (s, CH arom), 131.7 (s, C_q arom), 131.2 (s, CH arom), 130.7 (s, C_q arom), 129.6, 128.2 (both s, CH arom), 126.8 (s, C_q arom), 126.7, 119.4 (s, CH arom), 64.8 (s, CH_2), 20.7 (s, CH_3). ^{29}Si $\{^1H\}$ NMR (59.63 MHz, $CDCl_3$, 298 K): δ –32.5 (s). GC-MS (*m/z*): 318.1 (M^+).

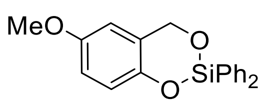

8e. ^{1}H NMR (300 MHz, $CDCl_3$, 298 K): δ 7.93-7.90 (m, 4H, CH arom), 7.62-7.52 (6H, CH arom), 7.15 (d, 1H, $^{3}J_{H\text{-}H}$ = 8.9, CH arom), 6.93 (dd, $^{3}J_{H\text{-}H}$ = 8.9, $^{4}J_{H\text{-}H}$ = 2.8, 1H, CH arom), 6.74 (d, $^{4}J_{H\text{-}H}$ = 2.8, 1H, CH arom), 5.25 (s, 2H, CH_2), 3.86 (s, 3H, OCH_3). $^{13}C\{^{1}H\}$-apt NMR (75.429 MHz, $CDCl_3$, 298 K): δ 154.2, 147.1 (both s, C_q arom) 135.0 (s, CH arom 131.7 (s, C_q arom), 131.3, 128.2 (both s, CH arom), 127.8 (s, C_q arom), 120.2, 114.4, 111.6 (all s, CH arom), 64.9 (s, CH_2), 55.8 (s, OCH_3). ^{29}Si $\{^{1}H\}$ NMR (59.63 MHz, $CDCl_3$, 298 K): δ –32.5 (s). GC-MS (*m/z*): 334.1 (M^+).

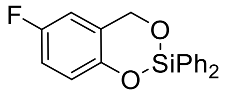

8f. NMR (300 MHz, $CDCl_3$, 298 K): δ 7.86-7.83 (m, 4H, CH arom), 7.62-7.49 (6H, CH arom), 7.09 (dd, $^{3}J_{H\text{-}H}$ = 8.8, $^{4}J_{H\text{-}F}$ = 4.8, 1H, CH arom), 7.01 (ddd, $^{3}J_{H\text{-}H}$ = 8.8, $^{4}J_{H\text{-}H}$ = 3.0, $^{3}J_{H\text{-}F}$ = 8.1, 1H, CH arom), 6.84 (dd, $^{4}J_{H\text{-}H}$ = 3.0, $^{3}J_{H\text{-}F}$ = 8.4, 1H, CH arom), 5.17 (s, 2H, CH_2). $^{13}C\{^{1}H\}$-apt NMR (75.429 MHz, $CDCl_3$, 298 K): δ 157.3 (d, $J_{C\text{-}F}$ = 240, C-F), 149.2 (s, C_q arom), 149.1 (s, C_q arom), 134.9, 131.4 (both s, CH arom), 131.2 (s, C_q arom), 128.26 (s, CH, Ph), 120.5 (d, $J_{C\text{-}F}$ = 7.8, CH arom), 115.5 (d, $J_{C\text{-}F}$ = 23.1, CH arom), 112.8 (d, $J_{C\text{-}F}$ = 23.6, CH arom), 64.4 (s, CH_2). ^{29}Si $\{^{1}H\}$ NMR (59.63 MHz, $CDCl_3$, 298 K): δ –32.1 (s). GC-MS (*m/z*): 322.1 (M^+).

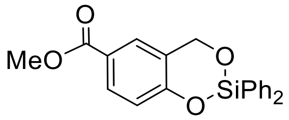

8g. ^{1}H NMR (300 MHz, $CDCl_3$, 298 K): δ 8.07 (dd, $J_{H\text{-}H}$ = 8.6, $J_{H\text{-}H}$ = 2.1, 1H, CH arom), 7.90 (d, $J_{H\text{-}H}$ = 2.1, 1H, CH arom), 7.87-7.84 (4H, CH arom), 7.61-7.49 (6H, CH arom), 7.19 (d, $J_{H\text{-}H}$ = 8.6, 1H, CH arom), 5.25 (s, 2H, CH_2), 3.98 (s, 3H, OCH_3). $^{13}C\{^{1}H\}$-apt NMR (75.429 MHz, C_6D_6, 298 K): δ 166.6 (s, C=O), 157.6 (s, C_q arom), 134.9, 131.5, 131.0 (all s, CH arom), 130.8 (s, C_q arom), 128.3 (s, CH arom), 127.0, 123.5 (both s, C_q arom), 119.7 (s, CH arom), 64.4 (s, CH_2), 52.0 (s, OCH_3). $^{29}Si\{^{1}H\}$ NMR (59.63 MHz, $CDCl_3$, 298 K): δ –31.6 (s). GC-MS (*m/z*): 362.1 (M^+).

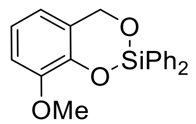

8h. ^{1}H NMR (300 MHz, $CDCl_3$, 298K): δ 7.78 (m, 4H, CH arom), 7.51-7.40 (6H, CH arom), 6.97-6.86 (2H, CH arom), 6.67 (m, 1H, CH arom), 5.14 (s, 2H, CH_2), 3.90 (s, 3H, OCH_3). $^{13}C\{^{1}H\}$-apt NMR (75.429 MHz, $CDCl_3$, 298 K): δ 150.3 (s, C_q arom), 143.0 (s, C_q arom) 135.1 (s, CH arom), 131.5 (s, C_q arom), 131.3 (s, CH arom), 128.3 (s, C_q arom), 128.2, 121.3, 118.4, 112.3 (all s, CH

arom), 64.6 (s, CH_2), 56.4 (s, OCH_3). $^{29}Si\{^1H\}$ NMR (59.63 MHz, $CDCl_3$, 298 K): δ –26.1 (s). GC-MS (*m/z*): 334.1(M^+).

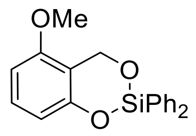

8i. ^1H NMR (300 MHz, $CDCl_3$, 298 K): δ 7.87-7.84 (m, 4H, CH arom), 7.59-7.47 (6H, CH arom), 7.25 (m, 1H, CH arom), 6.81 (dd, $^3J_{H-H}$ = 8.3, $^4J_{H-H}$ = 1.0, 1H, CH arom), 6.59 (dd, $^3J_{H-H}$ = 8.3, $^4J_{H-H}$ = 1.0, 1H, CH arom), 5.33 (s, 2H, CH_2), 3.86 (s, 3H, OCH_3). $^{13}C\{^1H\}$-apt NMR (75.429 MHz, $CDCl_3$, 298 K): δ 156.3 (s, C_q arom), 153.9 (s, C_q arom) 134.9 (s, CH arom), 131.5 (s C_q arom), 131.2, 128.5, 128.1 (all s, CH arom), 115.1 (s C_q arom), 112.5, 103.5 (both s, CH arom), 60.0 (s, CH_2), 55.6 (s, OCH_3). ^{29}Si $\{^1H\}$ NMR (59.63 MHz, $CDCl_3$, 298 K): δ –27.8 (s). GC-MS (*m/z*): 334.1 (M^+).

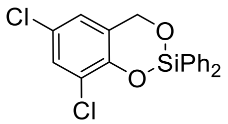

8j. ^1H NMR (300 MHz, $CDCl_3$, 298 K): δ 7.87-7.84 (m, 4H, CH arom), 7.63-7.41 (7H, CH arom), 7.00 (d, $^4J_{H-H}$ = 2.4, CH arom), 5.15 (s, 2H, CH_2). $^{13}C\{^1H\}$-apt NMR (75.429 MHz, $CDCl_3$, 298 K): δ 148.0 (s, C_q arom), 134.8 (s, CH arom), 131.5 (s, CH arom), 130.4 (s, C_q arom), 128.6 (s, C_q arom), 129.1 (s, CH arom), 128.2 (s, CH arom), 126.0, 125.1 (both s, C_q arom), 124.6 (s, CH arom), 64.1 (s, CH_2). $^{29}Si\{^1H\}$ NMR (59.63 MHz, $CDCl_3$, 298 K): δ –25.2 (s). GC-MS (*m/z*): 304.0 (M^+ – 2Cl + 2H).

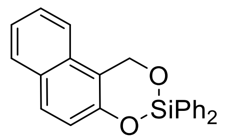

8k. ^1H NMR (300 MHz, $CDCl_3$, 298K): δ 7.58-7.75 (6H, CH-arom), 7.68 (1H, $^3J_{H-H}$ = 8.7, CH-arom), 7.54-7.38 (7H, CH-arom), 7.28 (2H, CH-arom), 5.68 (s, 2H, CH_2). $^{13}C\{^1H\}$-apt NMR (75.429 MHz, $CDCl_3$, 298 K): δ 151.0 (s, C_q arom), 135.1 (s, CH arom), 132.5 (s, C_q arom), 131.5 (s, CH arom), 130.8 (s, C_q arom), 129.5 (s, C_q arom), 129.5, 128.9, 128.4, 126.9, 124.0, 121.6, 121.2 (all s, CH arom), 117.7 (s, C_q arom), 62.5 (s, CH_2). ^{29}Si $\{^1H\}$ NMR (59.63 MHz, $CDCl_3$, 298 K): δ –27.7 (s). GC-MS (*m/z*): 354.2 (M^+).

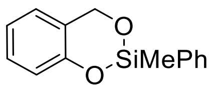

2-Methyl-2-phenyl-4*H*-1,3,2-benzodioxasilin.[10] ^1H NMR (300 MHz, $CDCl_3$, 298 K): δ 7.76 (m, 2H, CH arom), 7.55-7.43 (4H, CH arom), 7.34-7.29 (1H, CH arom), 7.11-7.01 (2H, CH arom) 5.12 (s, 2H, CH_2), 0.72 (s, 3H, CH_3).

• Structural Analysis of Complexes 3, 4, and 6

X-ray data were collected on a D8 Venture Bruker diffractometers (Mo radiation, λ = 0.71073 Å). The crystals were mounted under oil in a MiTeGen mount and cooled to 100(2) K with an open-flow nitrogen gas (Oxford Cryosystems). Data were corrected for absorption by using a multiscan method applied with the SADABS program.[11] The structures were solved by direct methods and refined by full-matrix least squares on F^2 with SHELXL2019,[12] including isotropic and subsequently anisotropic displacement parameters. The hydrogen atoms were observed in the last Fourier Maps or calculated, and refined freely or using a restricted riding model. The hydride ligands were located in the last difference Fourier maps and refined with restricted distance to osmium atoms (1.59(1) Å).

Crystal data for **3** (CCDC 2517083): $C_{36}H_{62}OOsP_2Si$, M_W 791.08, colorless, irregular block (0.119 x 0.097 x 0.080 mm^3), monoclinic, space group $P2_1/c$, *a*: 18.8802(7) Å, *b*: 11.2951(4) Å, *c*: 17.1328(6) Å, *β*: 93.6336(13)°, *V* = 3646.3(2) Å^3, *Z* = 4, *Z'* = 1, D_{calc}: 1.441 g cm^{-3}, F(000): 1624, T = 100(2) K, μ 3.644 mm^{-1}. 101617 measured reflections (2θ: 3-57°, ω and φ scans 0.5°), 9043 unique (R_{int} = 0.0394); min./max. transm. factors 0.610/0.746. Final agreement factors were R^1 = 0.0306 (7771 observed reflections, I > 2σ(I)) and wR^2 = 0.0718; data/restraints/parameters 9043/4/397; GoF = 1.062. Largest peak and hole 2.428 (close to Os atoms) and -1.730 e/ Å^3.

Crystal data for **4** (CCDC 2517085): $C_{35}H_{59}NOOsP_2Si$, M_W 790.06, colorless, irregular block, (0.147 x 0.099 x 0.066 mm^3), monoclinic, space group $P2_1/n$, *a*: 11.6911(4) Å, *b*: 17.1469(5) Å, *c*: 18.4537(6) Å, *β*: 95.0889(13)°, *V* = 3684.8(2) Å^3, *Z* = 4, *Z'* = 1, D_{calc}: 1.424 g cm^{-3}, F(000): 1616, T = 100(2) K, μ 3.606 mm^{-1}. 126309 measured reflections (2θ: 3-57°, ω and φ scans 0.5°), 9125 unique (R_{int} = 0.0343); min./max. transm. factors 0.608/0.746. Final agreement factors were R^1 = 0.0135 (8791 observed reflections, I > 2σ(I)) and wR^2 = 0.0295; data/restraints/parameters 9125/2/391; GoF = 1.065. Largest peak and hole 0.403 (close to Os atoms) and -0.494 e/ Å^3.

Crystal data for **6** (CCDC 2517084): $C_{34}H_{58}N_2O_2OsP_2Si$, CH_4O, M_W 839.09, colorless, irregular block, (0.211 x 0.133 x 0.080 mm^3), monoclinic, space group $P2_1/c$, *a*: 18.7518(11) Å, *b*: 11.3362(6) Å, *c*: 17.9667(10) Å, *β*: 98.534(2)°, *V* = 3777.0(4) Å^3, *Z* = 4, *Z'* = 1, D_{calc}: 1.476 g cm^{-3}, F(000): 1720, T = 100(2) K, μ 3.527 mm^{-1}. 156768 measured

reflections (2θ: 3-57°, ω and φ scans 0.5°), 9362 unique (R_{int} = 0.0382); min./max. transm. factors 0.548/0.746. Final agreement factors were R^1 = 0.0144 (9117 observed reflections, $I > 2\sigma(I)$) and wR^2 = 0.0348; data/restraints/parameters 9362/2/419; GoF = 1.043. Largest peak and hole 0.399 (close to Os atoms) and -0.783 e/ $Å^3$.

• Computational Details

All mechanistic calculations were performed at the DFT level using the B3LYP functional[13] supplemented with the Grimme's dispersion correction D3[14] as implemented in Gaussian09.[15] The osmium atoms were described by means of an effective core potential SDD for the inner electron[16] and its associated double-ζ basis set for the outer ones, complemented with a set of f-polarization functions for osmium.[17] The 6-31G** basis set was used for all the other atoms.[18] The SMD continuum model was used to model the effects of the solvent (benzene). Reactants, intermediates, and products were also characterized by frequency calculations and has positive definite Hessian matrices thus confirming that the computed structure is a minimum on the potential energy surface. Transition states were identified by having one imaginary frequency in the Hessian matrix. It was confirmed that transition states connect with the corresponding intermediates by means of application of an eigenvector corresponding to the imaginary frequency and subsequent optimization of the resulting structures. Gibbs energies were computed at 298.15 K and 1 atmosphere. All values collected in figures correspond to Gibbs energies in kcal mol^{-1}.

AIM and NBO calculations were performed with the AIMII[19] and NBO7[20] programs employing the geometry optimizations performed at the BP86 level[21] in conjunction with def2-TZVPP basis sets,[22] with inclusion of dispersion interactions using the Grimme's D3 term.[11]

Table S1. NBO and AIM Selected Properties for Complex 6

Os-Si bond

$d(Os\text{-}Si)_{exp}$ (Å)	$d(Os\text{-}Si)_{DFT}$ (Å)	Δq(Os-Si) (e)	WBI (Os-Si)	$\nabla^2\rho(r)$ (e $Å^{-3}$)	ρ(r)	ε(r)	$\|V(r)\|/G(r)$
2.3408(4)	2.3655	1.81	0.475	0.068	0.133	0.052	2.66

Si-O bond

$d(Si\text{-}O)_{exp}$ (Å)	$d(Si\text{-}O)_{DFT}$ (Å)	Δq(Si-O) (e)	WBI (Si-O)	$\nabla^2\rho(r)$ (e $Å^{-3}$)	ρ(r)	ε(r)	$\|V(r)\|/G(r)$	$E^{(2)}$ LP(O)→LV(Si) (kcal mol^{-1})
1.719(1)	1.756	2.54	0.449	0.491	0.108	0.007	1.24	61.63 (LP3→LV2)<br>62.13 (LP3→LV3)

Table S2. Energies of Optimized Molecules

Optimized Molecule	Energy: sum of electronic and thermal Free Energies (Hartree/Molecule)
**1**	-2240.647252
**$TS_{1\text{-}A}$**	-2548.0244
**A**	-1487.919066
**$TS_{A\text{-}1}$**	-2241.779784
**$TS_{(1\text{-}A)b}$**	-2587.318612
**$TS_{A\text{-}3}$**	-2548.050309
**3**	-2546.91826
**PhOH**	-307.418519
**$PhCH_2OH$**	-346.70433
**H_2SiPh_2**	-753.893109
**$HSi(OPh)Ph_2$**	-1060.160587
**$HSi(OCH_2Ph)Ph_2$**	-1099.447311
**H_2**	-1.179418
**$TS_{(1\text{-}B)}$**	-2240.644902
**B**	-2240.644231
**$TS_{(B\text{-}C)}$**	-2700.657092
**C**	-2239.462287
**$TS^a_{(C\text{-}D)}$**	-2700.685627
**D^a**	-2700.686575
**$TS^a_{(D\text{-}E)}$**	-2700.685653
**E^a**	-2700.727182
**$TS^a_{(E\text{-}F)}$**	-2700.727966
**F^a**	-2700.733444
**$TS^a_{(F\text{-}A)}$**	-2700.688907
**$TS^b_{(C\text{-}D)}$**	-2700.678796
**D^b**	-2700.678682
**$TS^b_{(D\text{-}E)}$**	-2700.673986
**E^b**	-2700.715058
**$TS^b_{(E\text{-}F)}$**	-2700.673986
**F^b**	-2700.726736
**$TS^b_{(F\text{-}G)}$**	-2700.697954
**G^b**	-2700.703184
**$TS^b_{(G\text{-}A)}$**	-2700.695662
**$TS_{(1\text{-}A)c}$**	-2700.64071
**2-hydroxy-5-methylbenzaldehyde**	-460.051229
**2-(hydroxymethyl)-4-methylphenol**	-461.22532
**6-methyl-2,2-diphenyl-4H-benzo[*d*][1,3,2]dioxasiline**	-1212.828466
Optimized Molecule	Energy: E(RB-P86) (Hartree/Molecule)
**3-TZ**	-2548.445675
**4-TZ**	-2563.329497
**5-TZ**	-2543.440329

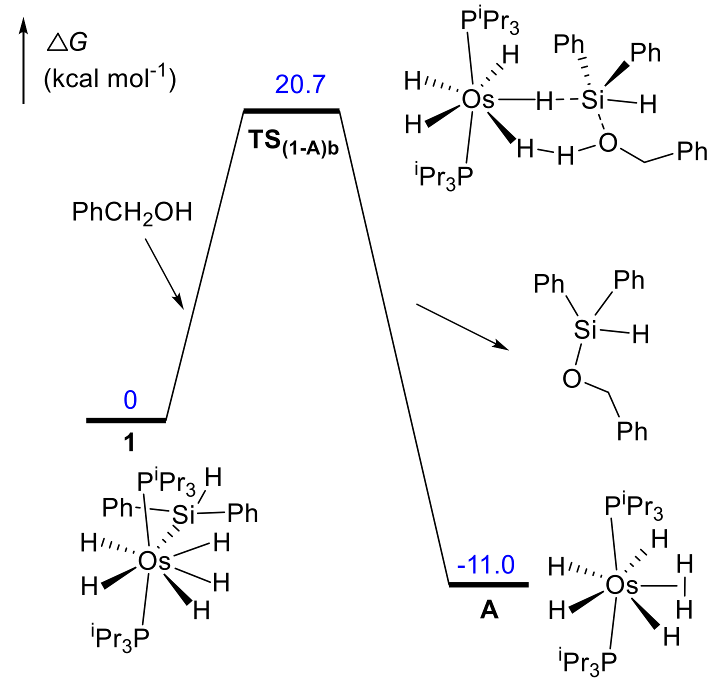

Figure S1. Energy profile (kcal mol^{-1}) for the nucleophilic attack of benzyl alcohol to pentahydride-silyl $OsH_5(SiHPh_2)(P^iPr_3)_2$ (**1**).

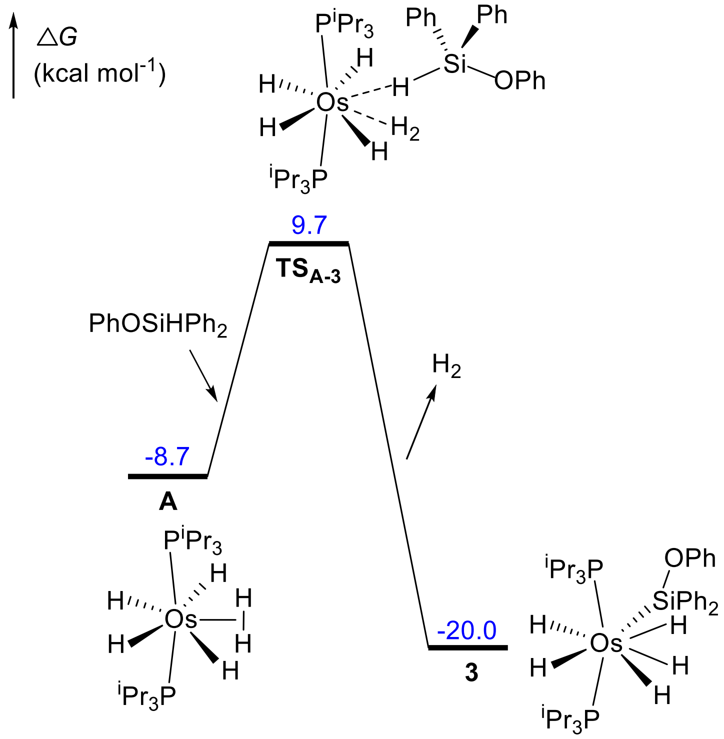

Figure S2. Energy profile (kcal mol^{-1}) for the reaction of intermediate **A** with $HSi(OPh)Ph_2$ to afford $OsH_5\{Si(OPh)Ph_2\}(P^iPr_3)_2$ (**3**).

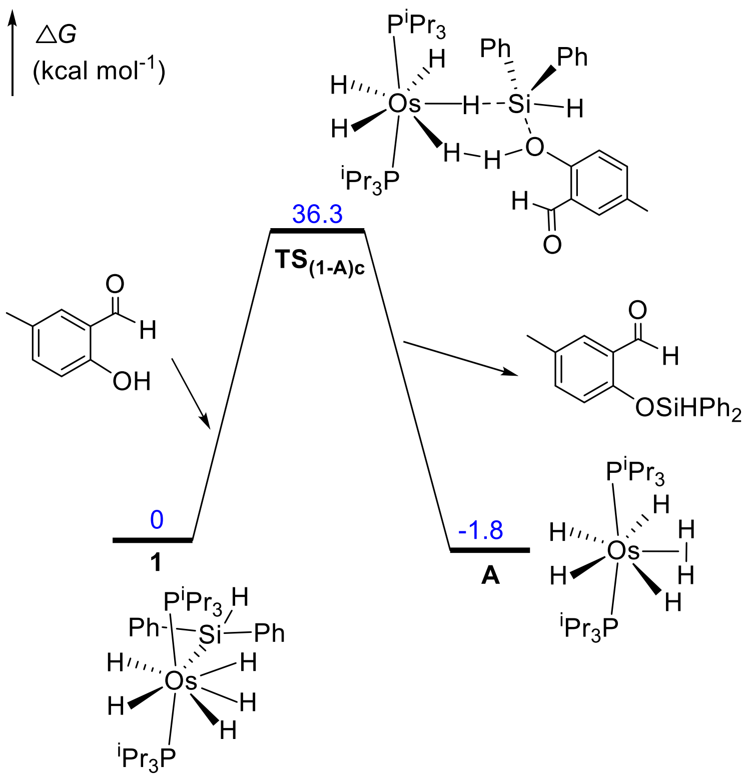

Figure S3. Energy profile (kcal mol^{-1}) for the nucleophilic attack of 2-hydroxy-5-methylbenzaldehyde to pentahydride-silyl $OsH_5(SiHPh_2)(P^iPr_3)_2$ (**1**).

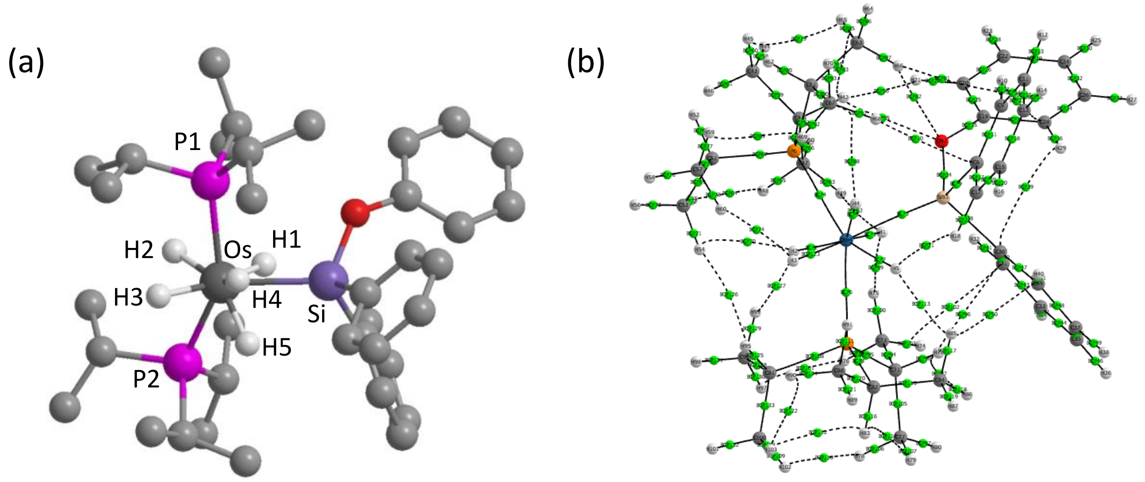

Figure S4. (a) DFT optimized structure of **3**. Hydrogen atoms (except hydrides) are omitted for clarity. Selected bond lengths (Å) and angles (deg): Os–P1 = 2.389, Os–P2 = 2.401, Os–Si = 2.434, Os–H1 = 1.669, Os–H2 = 1.664, Os–H3 = 1.675, Os–H4 = 1.624, Os–H5 = 1.644; P1–Os–P2 = 146.1, P1–Os–Si = 95.5, P2–Os–Si = 110.5. (b) AIM analysis for complex **3**. The solid lines connecting the atomic nuclei are the bond paths, while the small green spots indicate the corresponding bond critical points (legend: H (white), Si (violet), C (gray), P (purple), O (red), and Os (dark gray)).

• **NMR spectra**

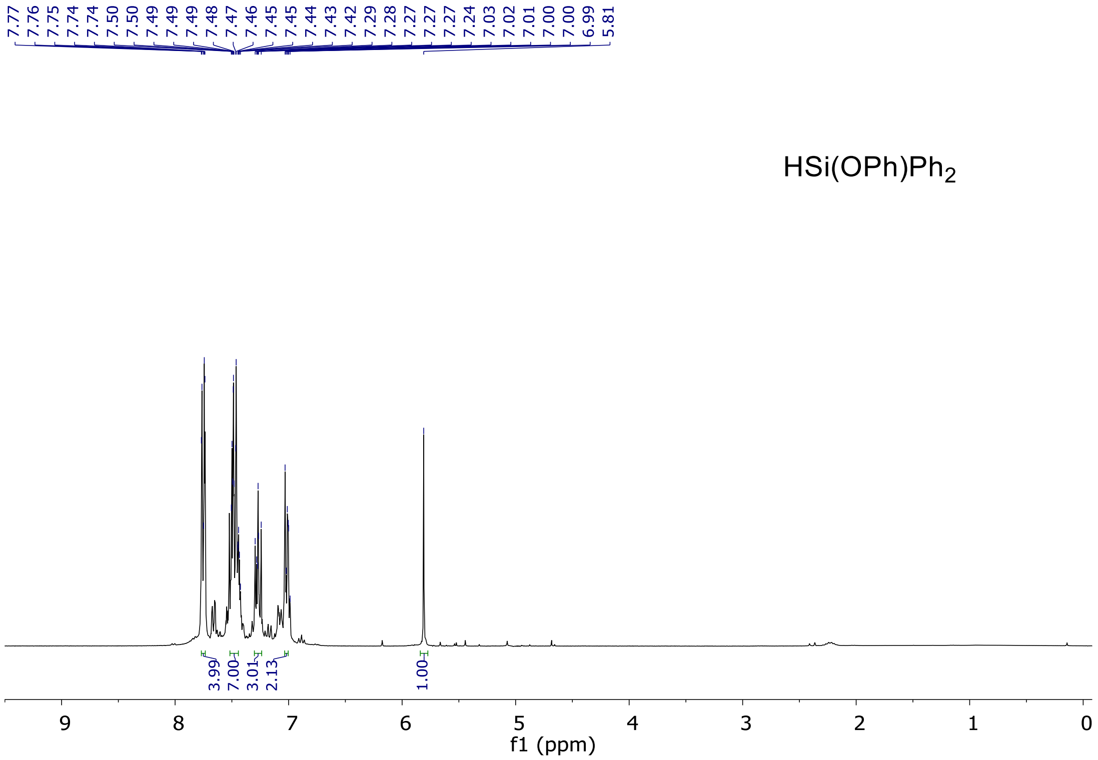

Figure S5. ^{1}H NMR spectrum (300 MHz, $CDCl_3$, 298 K) of phenoxydiphenylsilane.

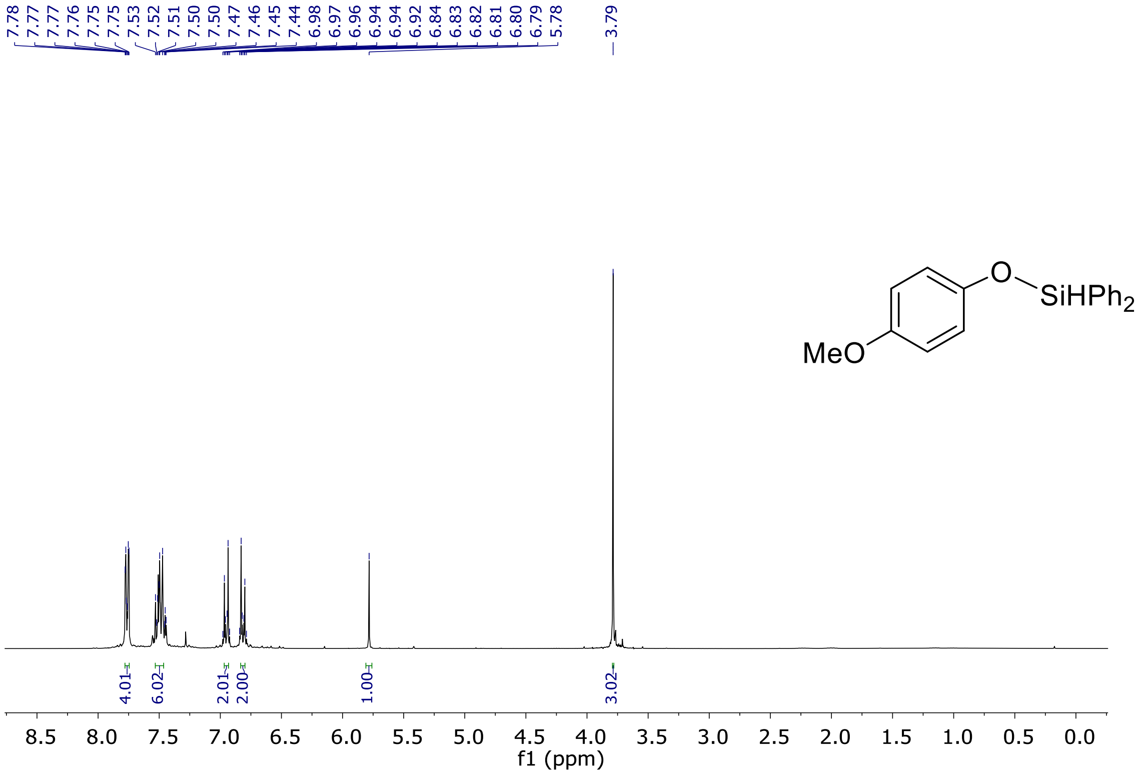

Figure S6. ^{1}H NMR spectrum (300 MHz, $CDCl_3$, 298 K) of (4-methoxyphenoxy)diphenylsilane

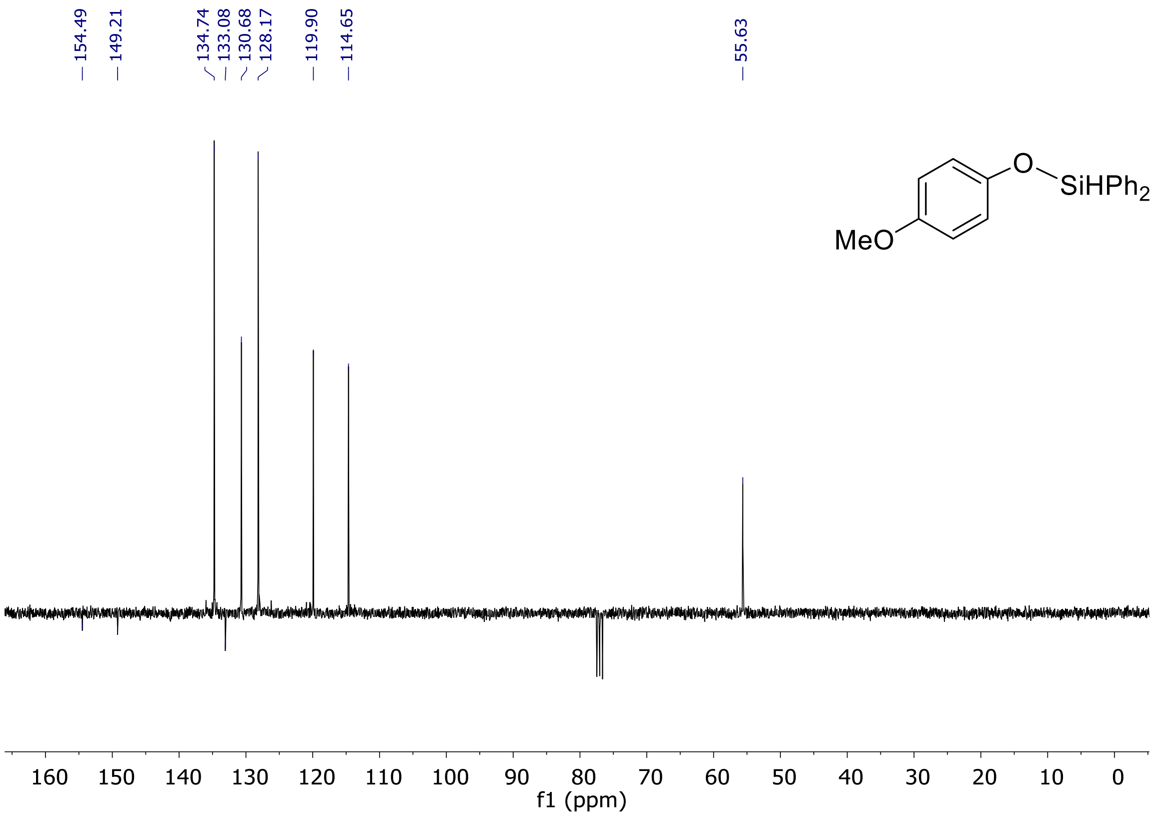

Figure S7. $^{13}C\{^{1}H\}$-apt NMR spectrum (75.429 MHz, $CDCl_3$, 298 K) of (4-methoxyphenoxy)diphenylsilane.

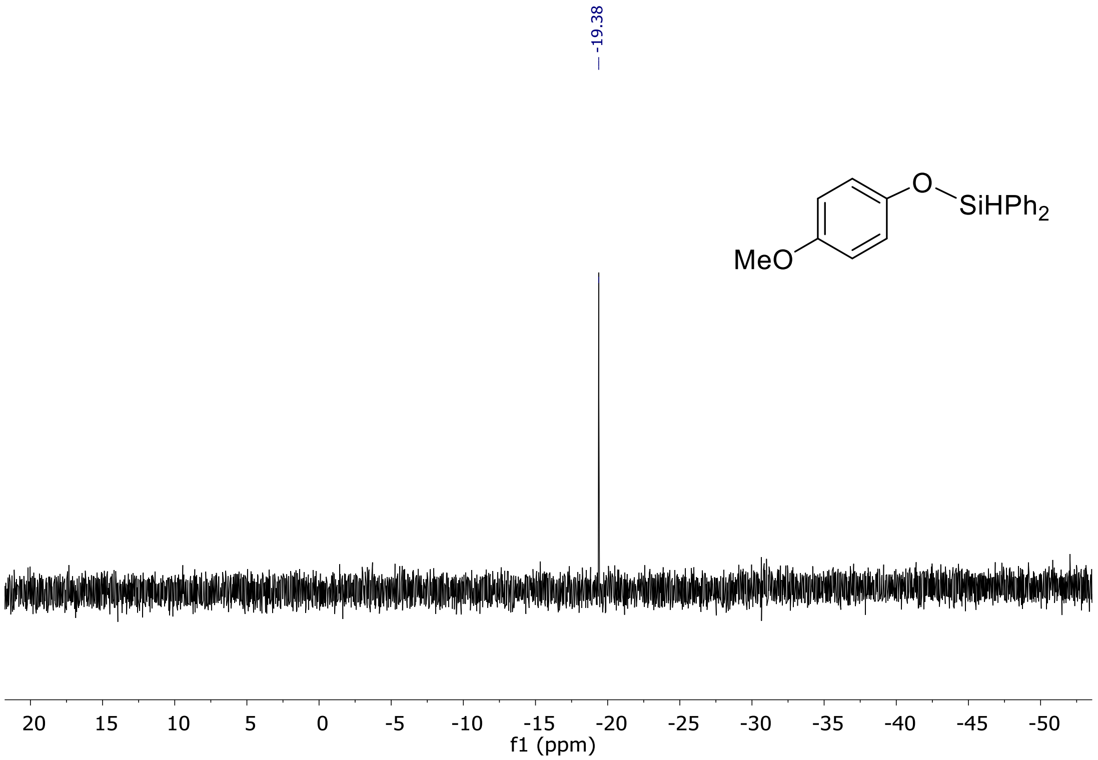

Figure S8. $^{29}Si\{^{1}H\}$ NMR spectrum (59.63 MHz, $CDCl_3$, 298 K) of (4-methoxyphenoxy)diphenylsilane.

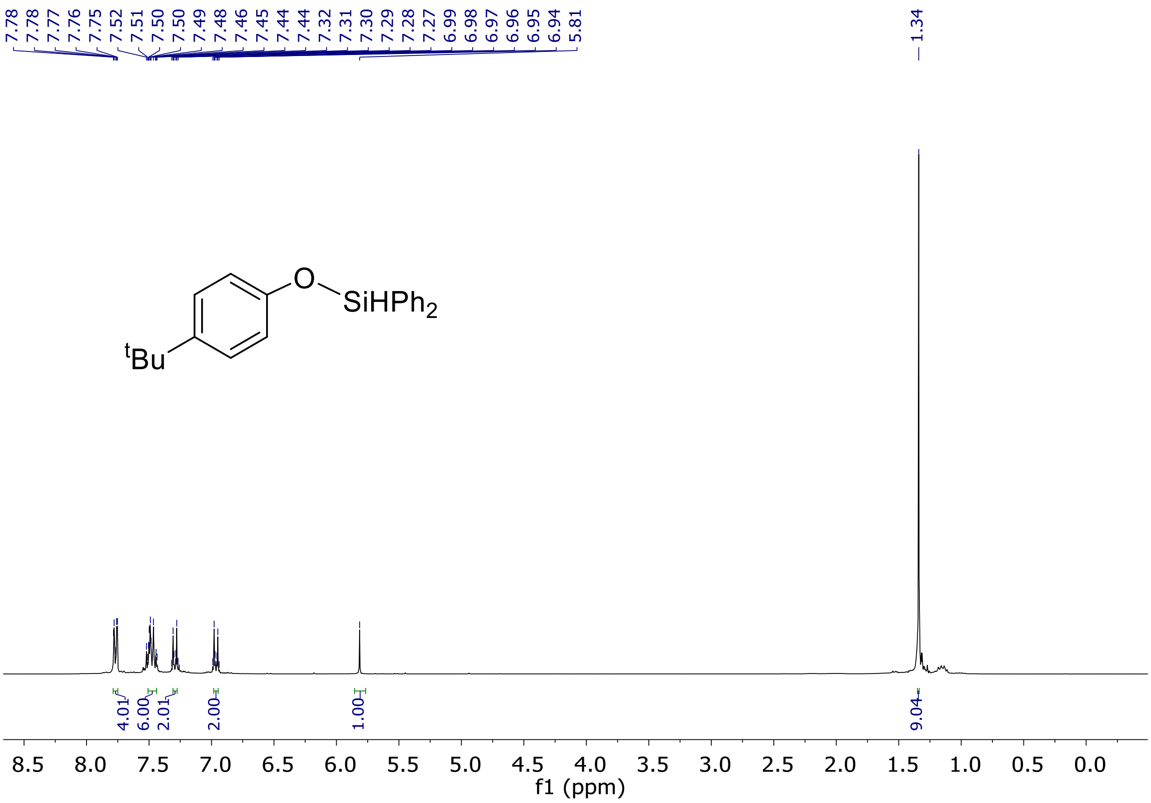

Figure S9. ^{1}H NMR spectrum (300 MHz, $CDCl_3$, 298 K) of (4-*t*-butylphenoxy)diphenylsilane.

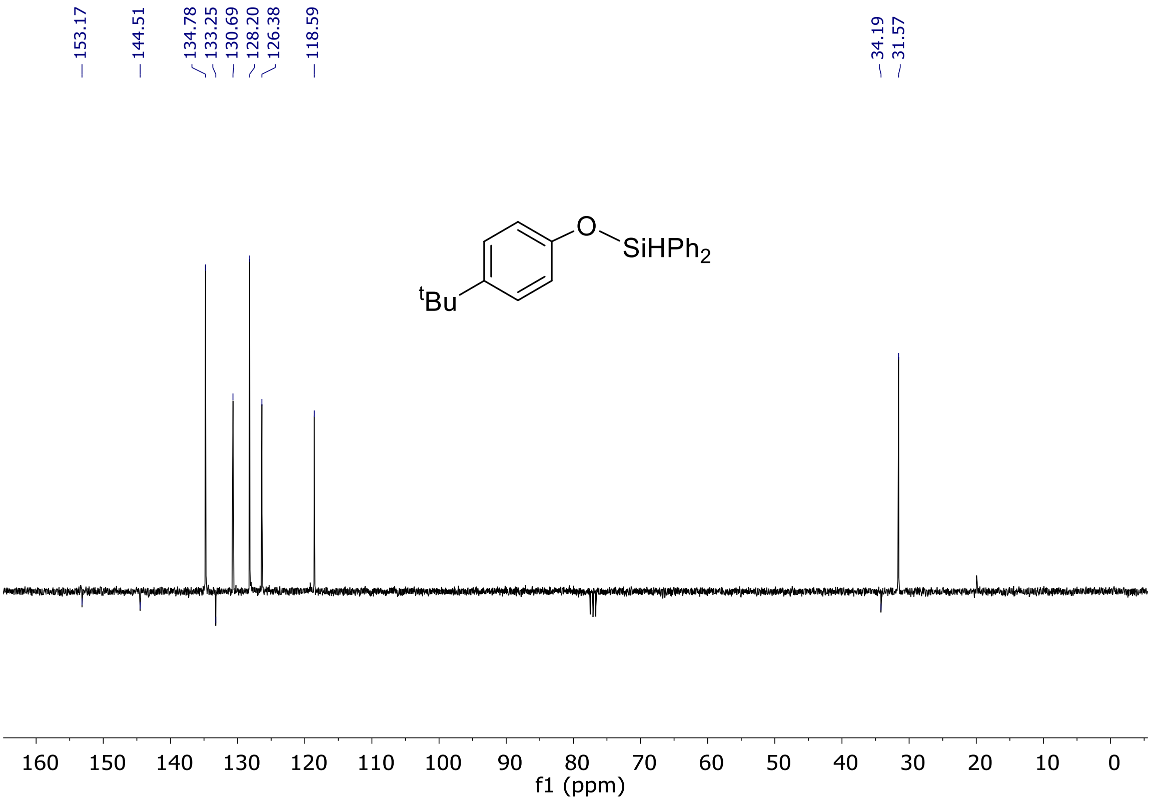

Figure S10. $^{13}C\{^{1}H\}$-apt NMR spectrum (75.429 MHz, $CDCl_3$, 298 K) of (4-*t*-butylphenoxy)diphenylsilane.

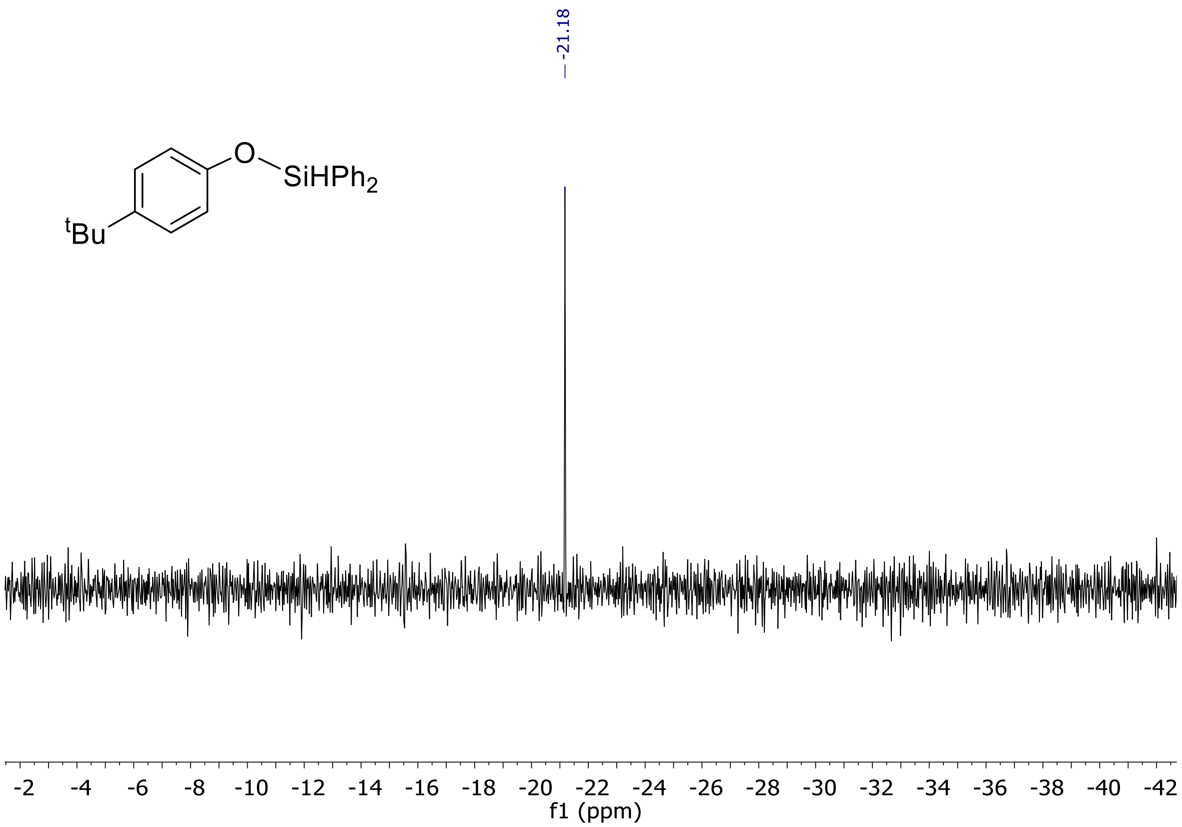

Figure S11. $^{29}Si\{^{1}H\}$ NMR spectrum (59.63 MHz, $CDCl_3$, 298 K) of (4-*t*-butylphenoxy)diphenylsilane.

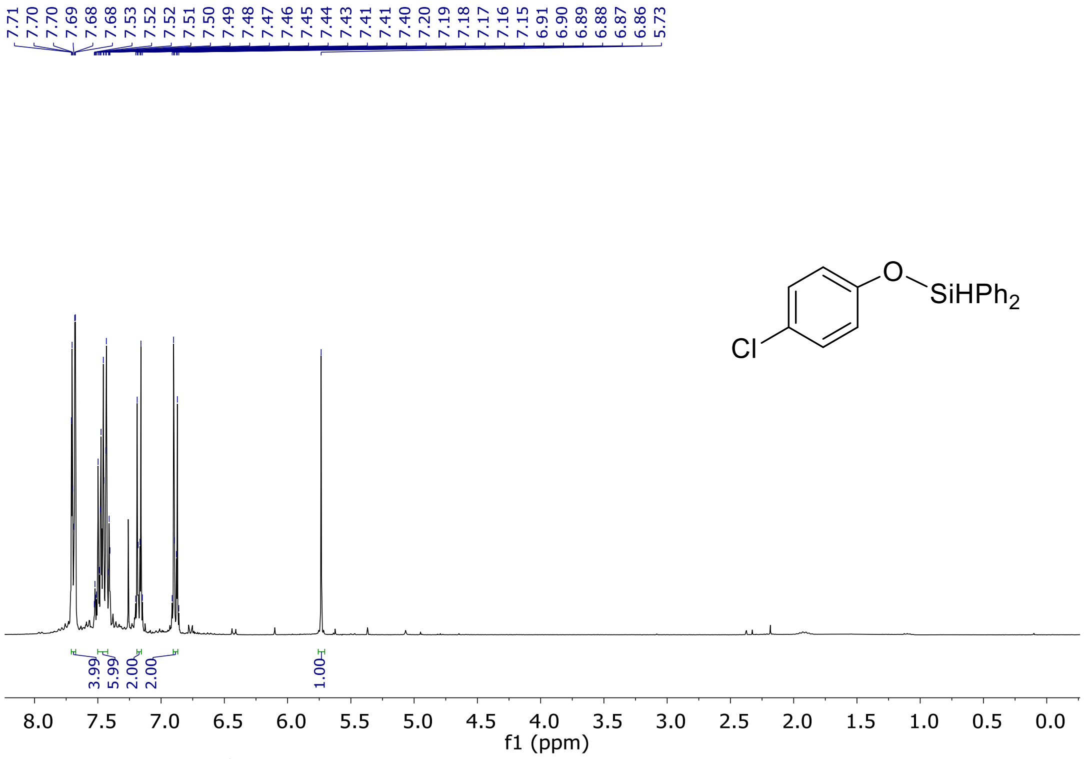

Figure S12. ^{1}H NMR spectrum (300 MHz, $CDCl_3$, 298 K) of (4-chlorophenoxy)diphenylsilane.

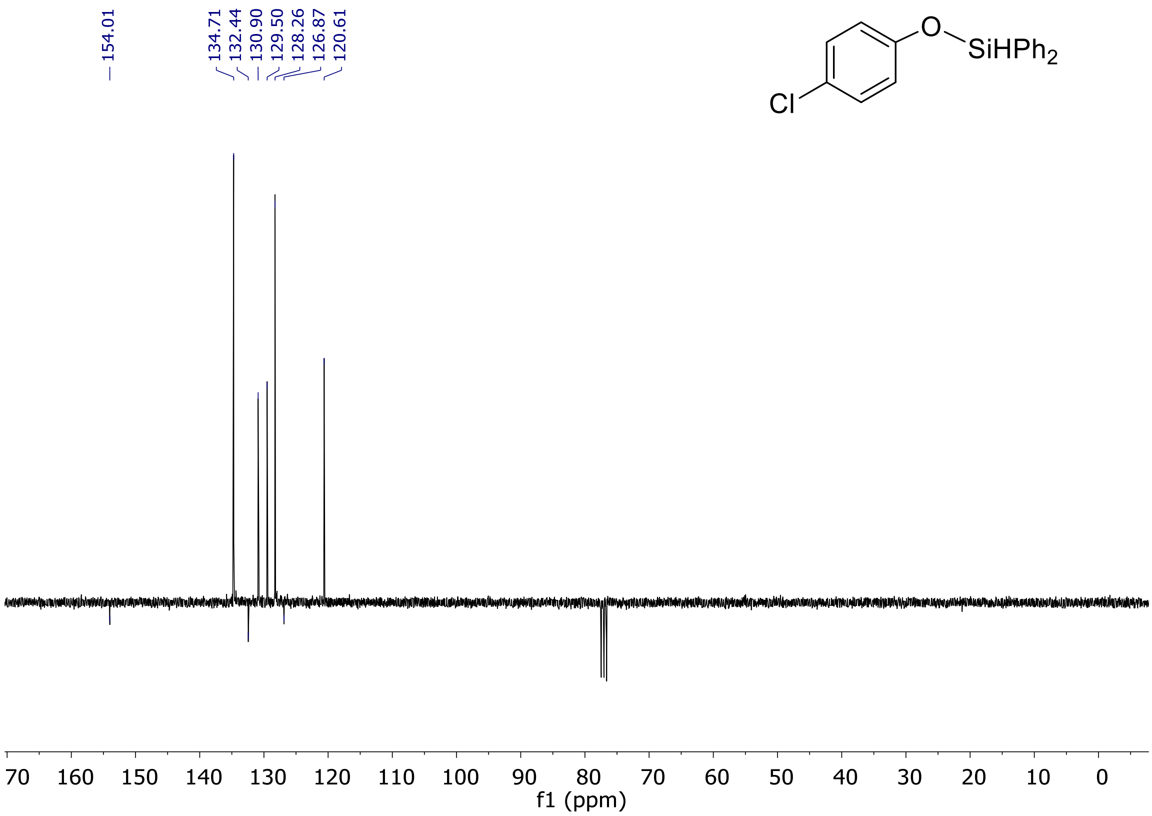

Figure S13. $^{13}C\{^{1}H\}$-apt NMR spectrum (75.429 MHz, $CDCl_3$, 298 K) of (4-chlorophenoxy)diphenylsilane.

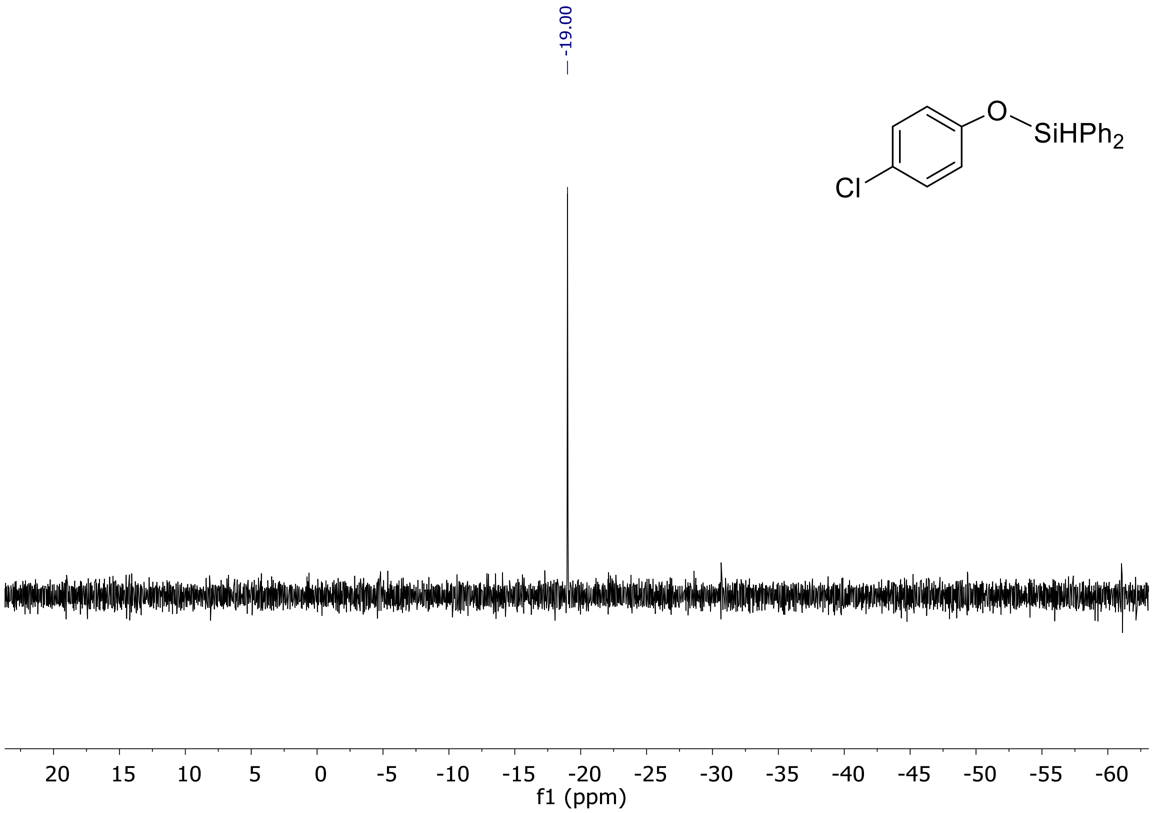

Figure S14. $^{29}Si\{^{1}H\}$ NMR spectrum (59.63 MHz, $CDCl_3$, 298 K) of (4-chlorophenoxy)diphenylsilane.

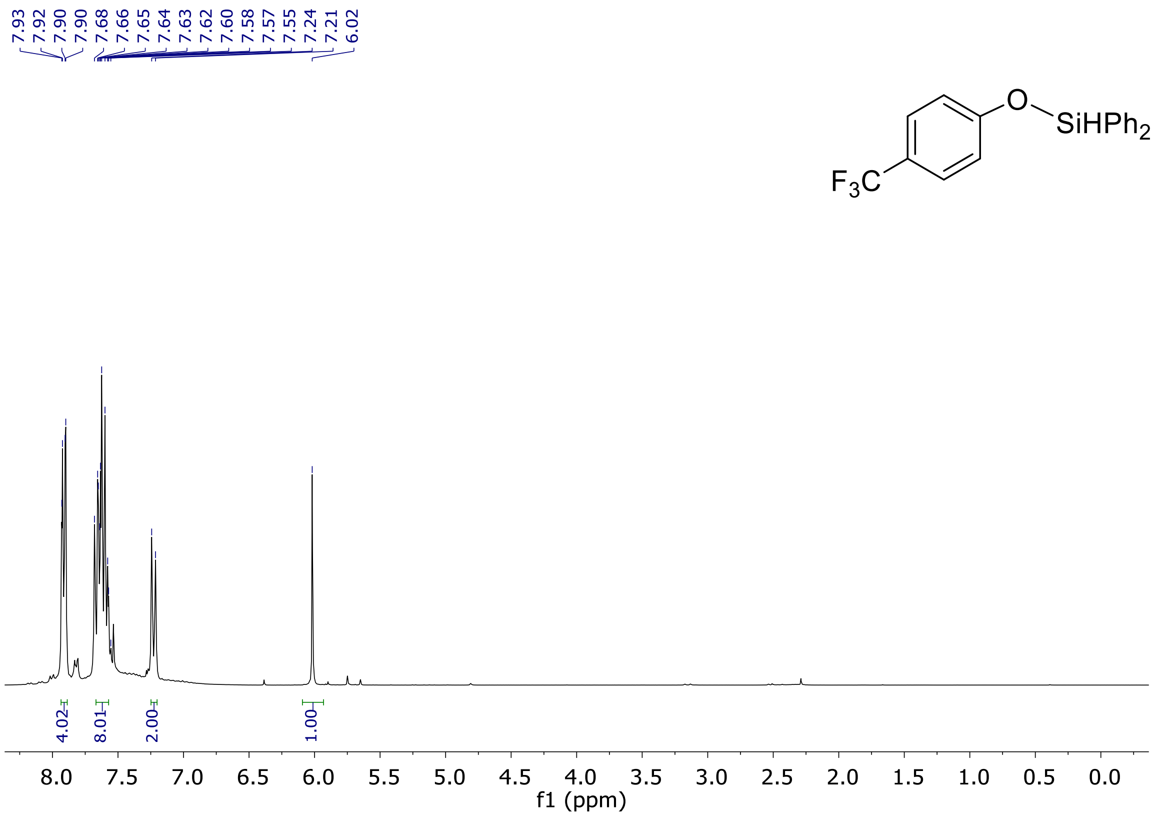

Figure S15. ^{1}H NMR spectrum (300 MHz, $CDCl_3$, 298 K) of (4-trifluoromethylphenoxy)diphenylsilane.

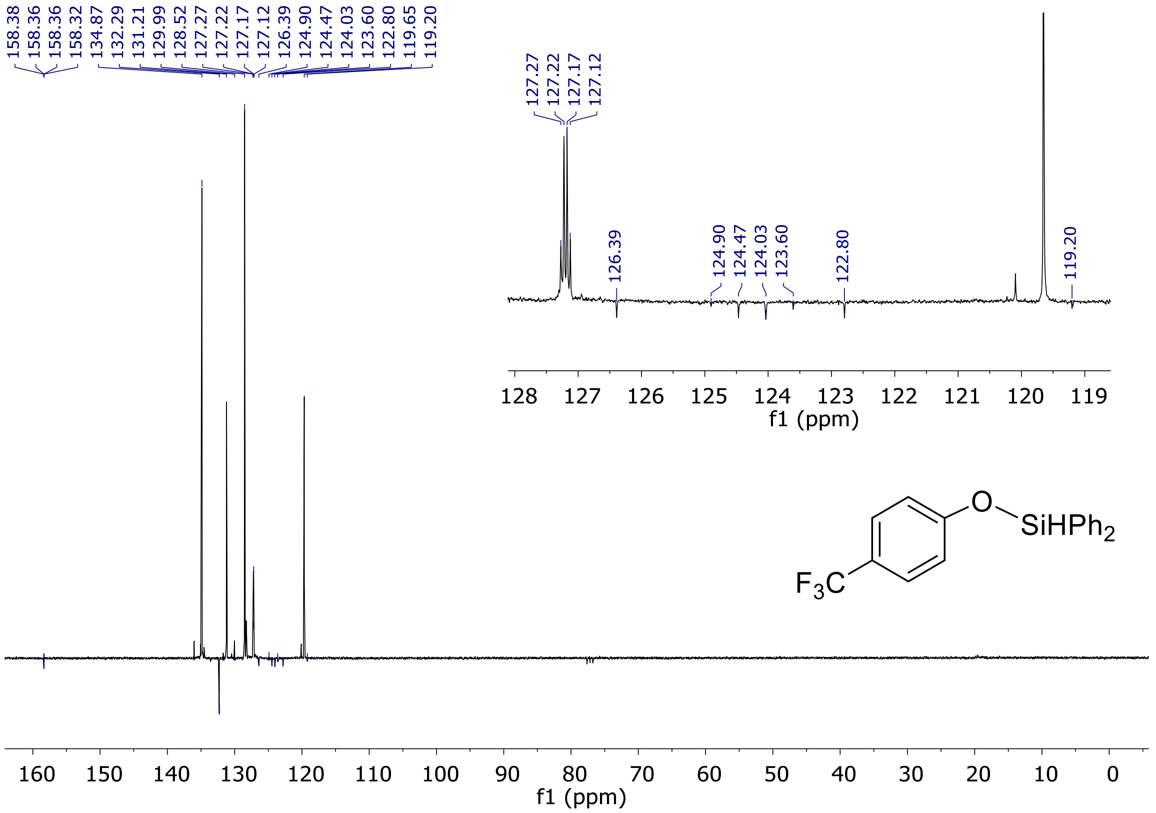

Figure S16. $^{13}C\{^{1}H\}$-apt NMR spectrum (75.429 MHz, $CDCl_3$, 298 K) of (4-trifluoromethylphenoxy)diphenylsilane.

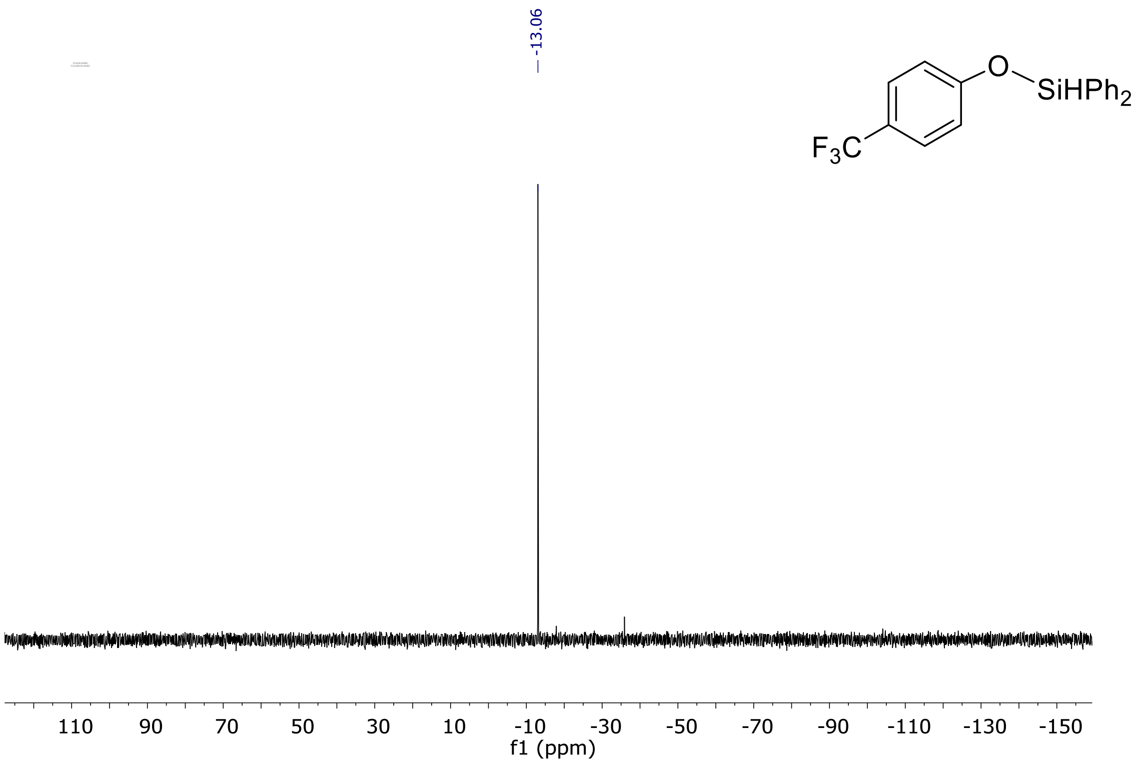

Figure S17. $^{29}Si\{^1H\}$ NMR spectrum (59.63 MHz, $CDCl_3$, 298 K) of (4-trifluoromethylphenoxy)diphenylsilane.

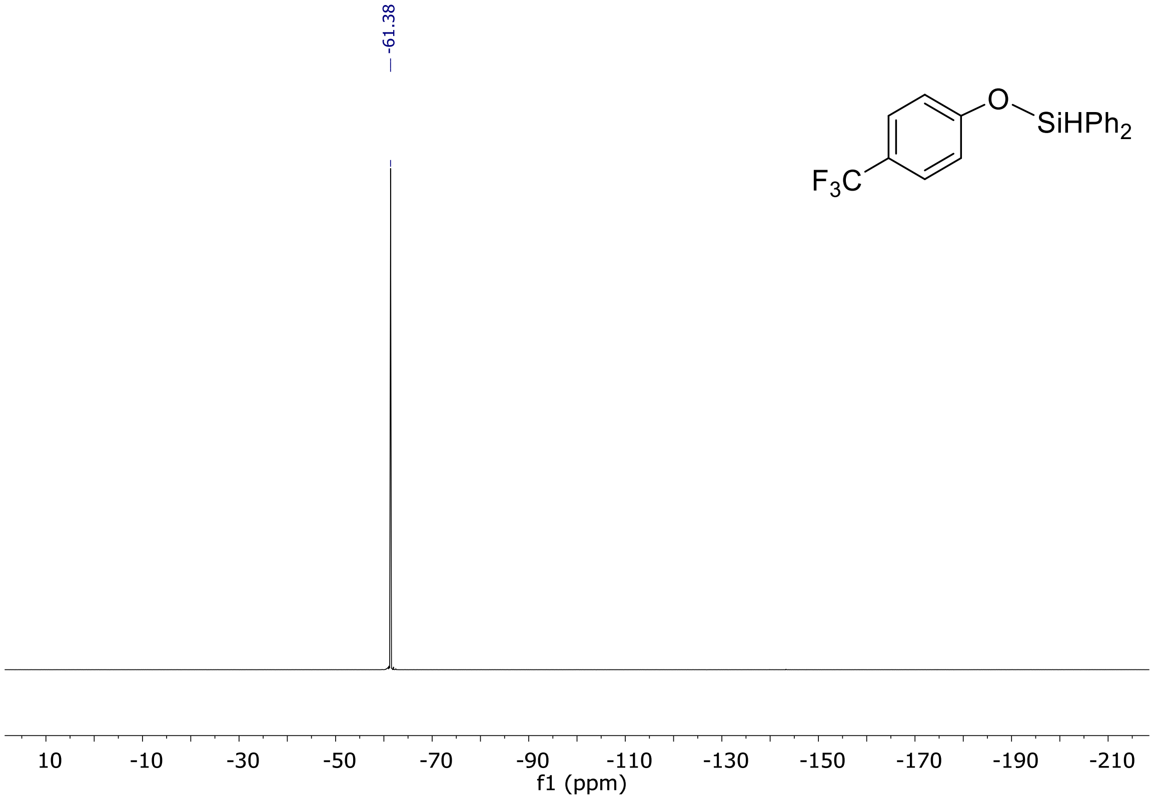

Figure S18. $^{19}F\{^1H\}$ NMR spectrum (282.33 MHz, $CDCl_3$, 298 K) of (4-trifluoromethylphenoxy)diphenylsilane.

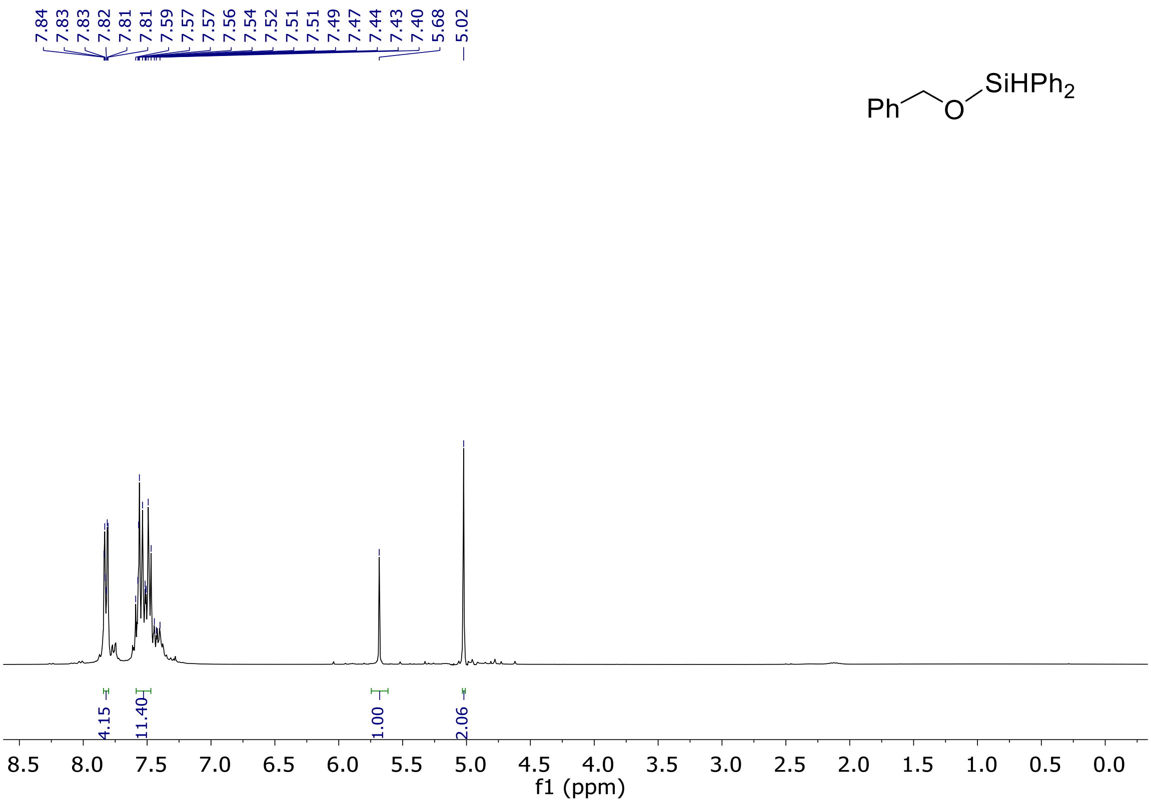

Figure S19. ^{1}H NMR spectrum (300 MHz, $CDCl_3$, 298 K) of (benzyloxy)diphenylsilane.

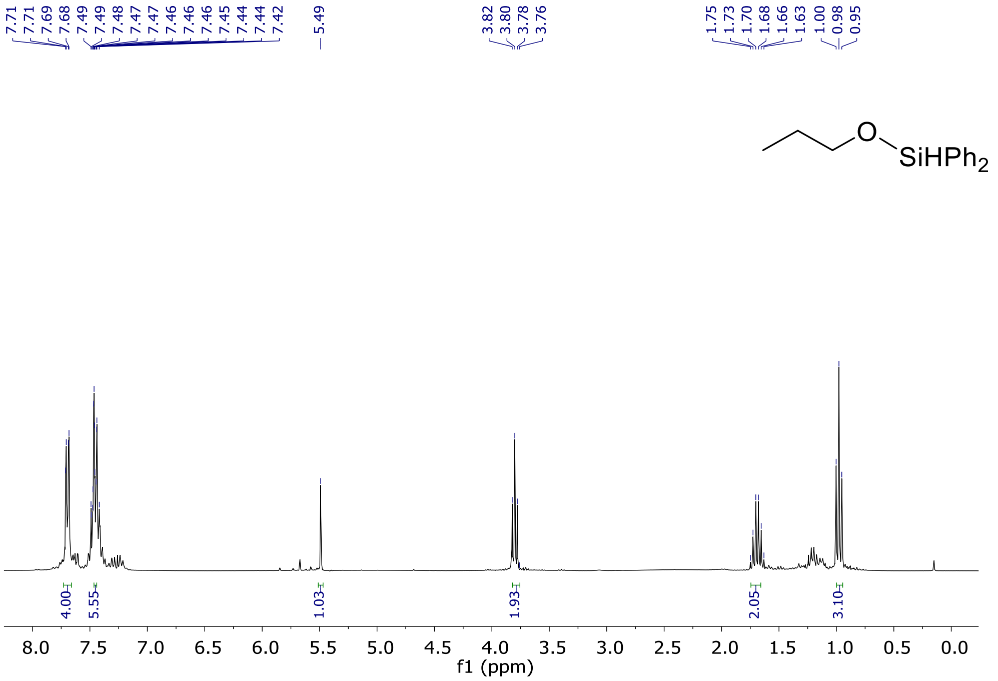

Figure S20. ^{1}H NMR spectrum (300 MHz, $CDCl_3$, 298 K) of diphenyl(propoxy)silane.

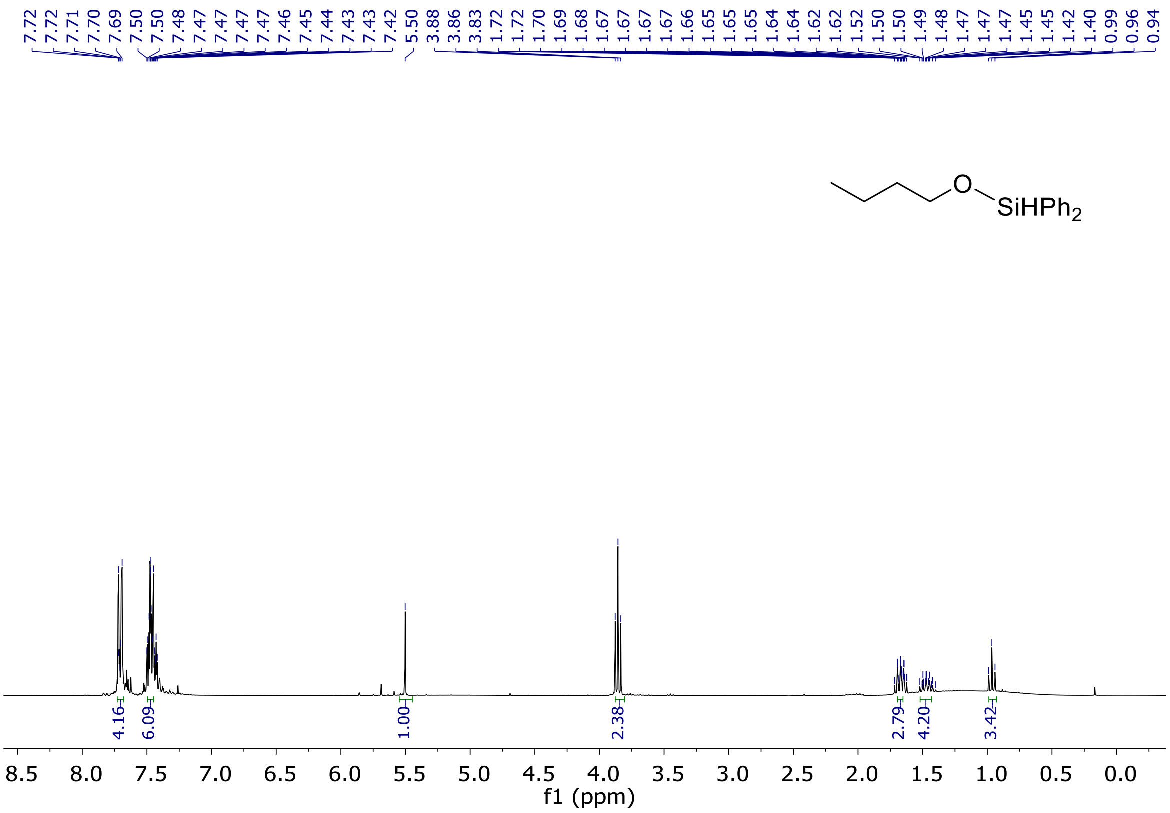

Figure S21. ^{1}H NMR spectrum (300 MHz, CDCl$_3$, 298 K) of diphenyl(butoxy)silane.

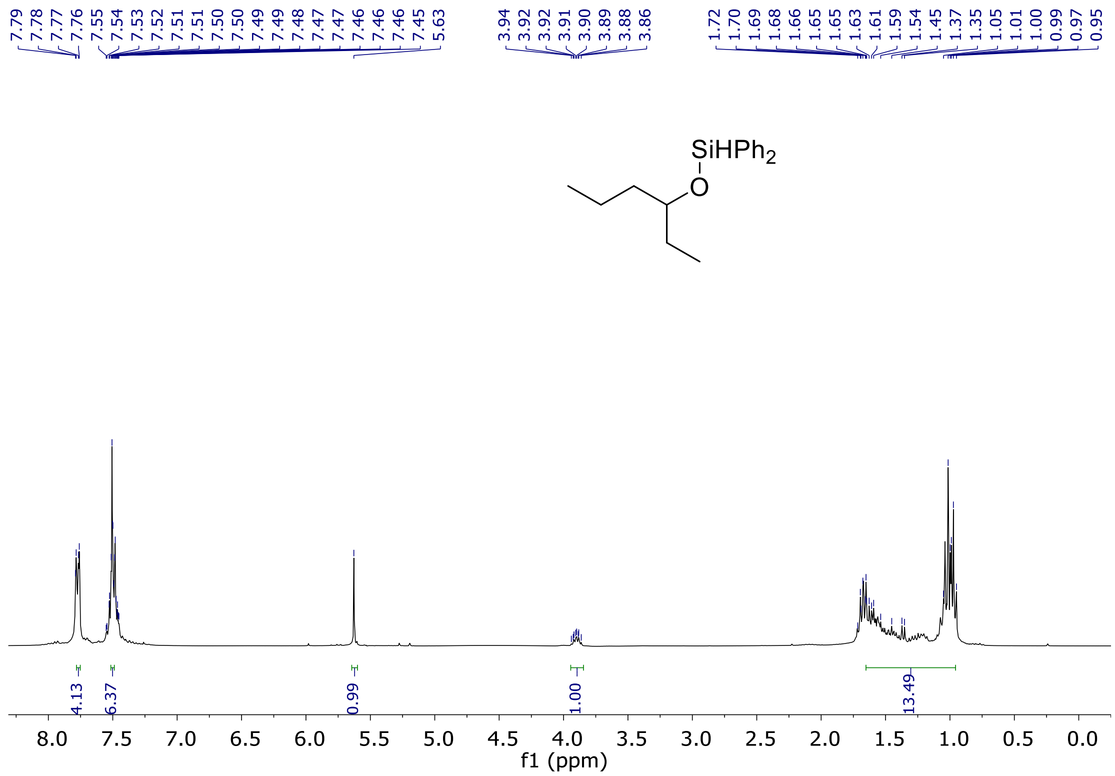

Figure S22. ^{1}H NMR spectrum (300 MHz, CDCl$_3$, 298 K) of (hexan-3-yloxy)diphenylsilane.

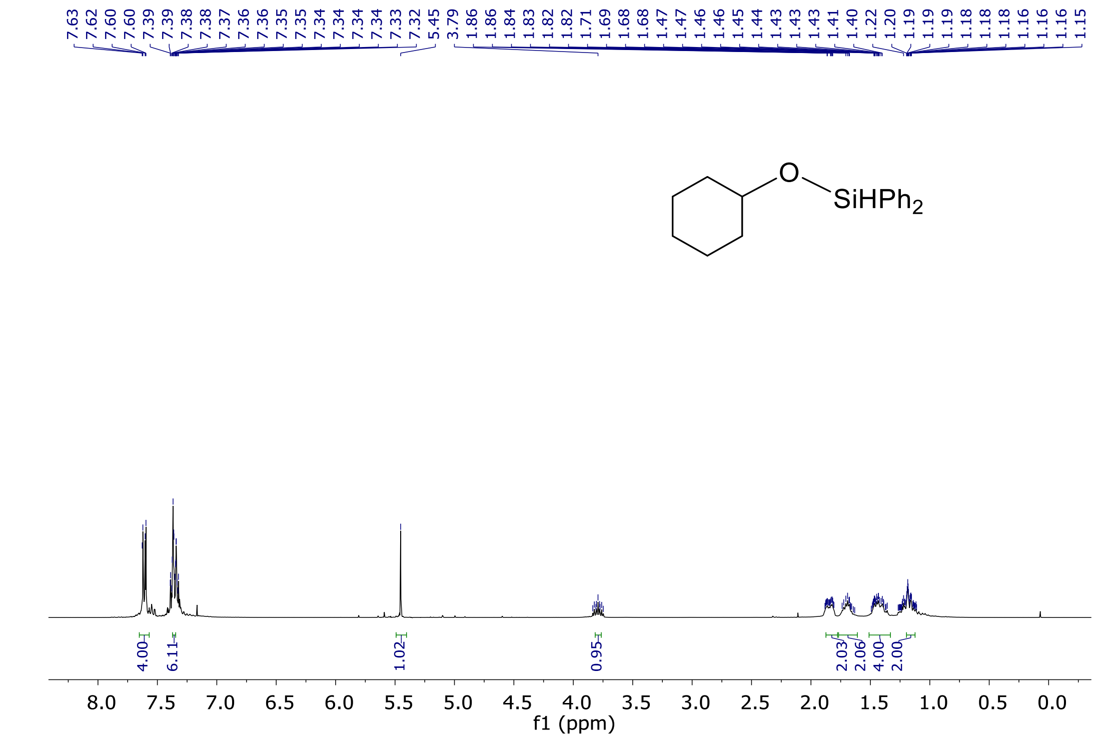

Figure S23. ^{1}H NMR spectrum (300 MHz, $CDCl_3$, 298 K) of (cyclohexyloxy)diphenylsilane.

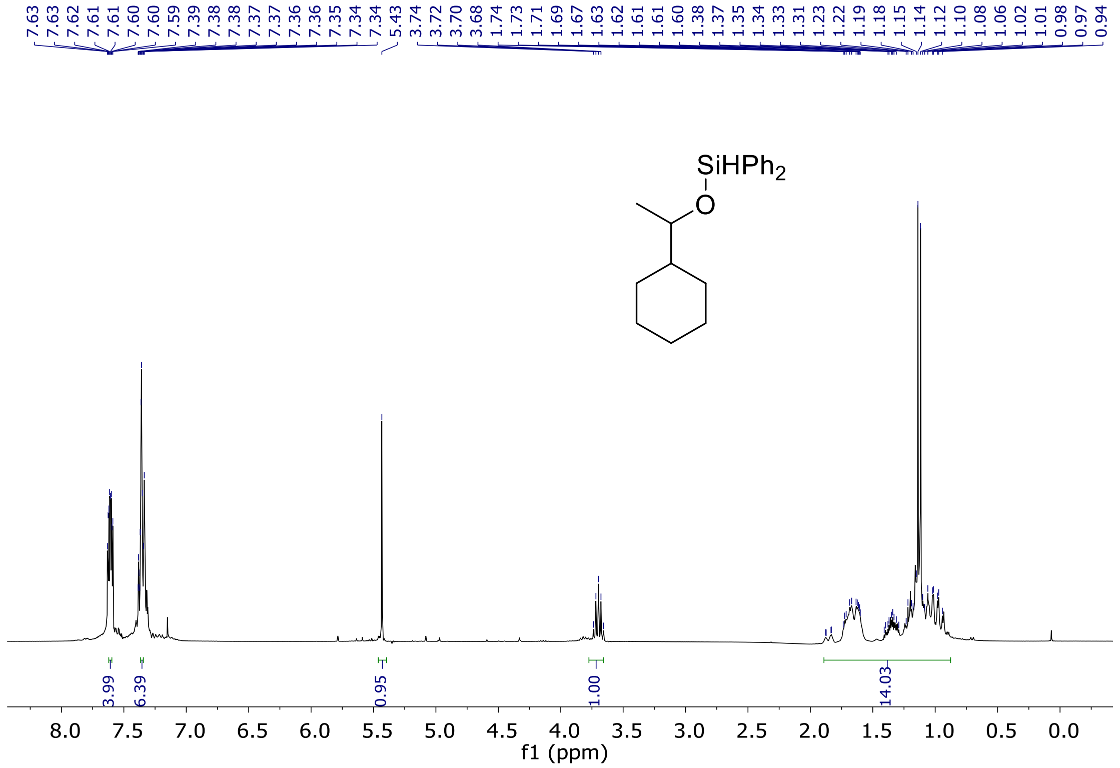

Figure S24. ^{1}H NMR spectrum (300 MHz, $CDCl_3$, 298 K) of (1-cyclohexylethoxy)diphenylsilane.

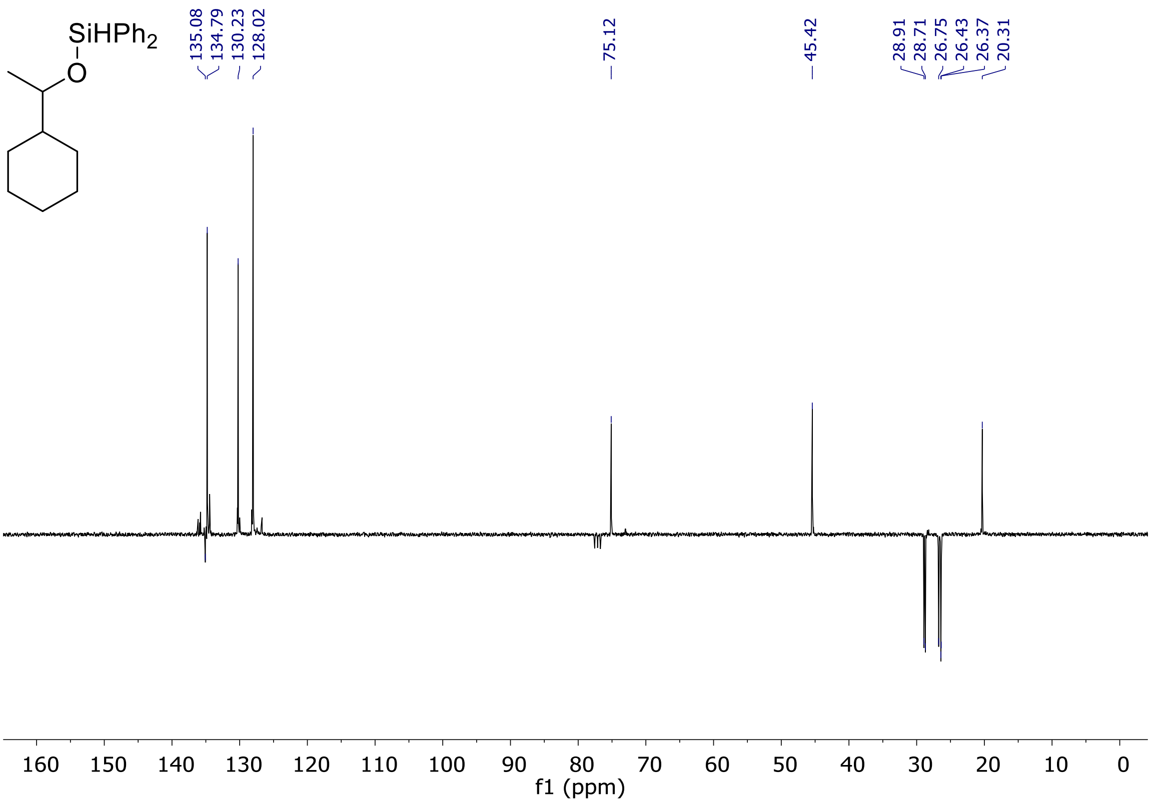

Figure S25. $^{13}C\{^{1}H\}$-apt NMR spectrum (75.429 MHz, $CDCl_3$, 298 K) of (1-cyclohexylethoxy)diphenylsilane.

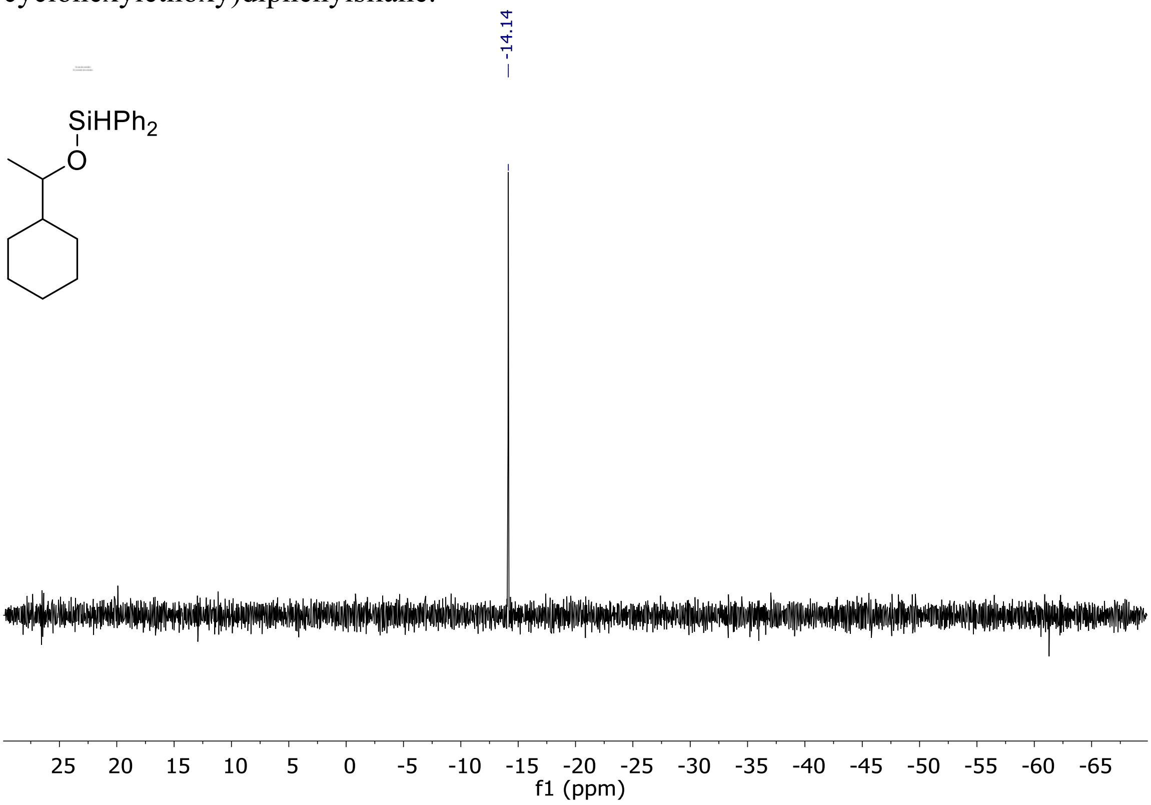

Figure S26. $^{29}Si\{^{1}H\}$ NMR spectrum (59.63 MHz, $CDCl_3$, 298 K) of (1-cyclohexylethoxy)diphenylsilane.

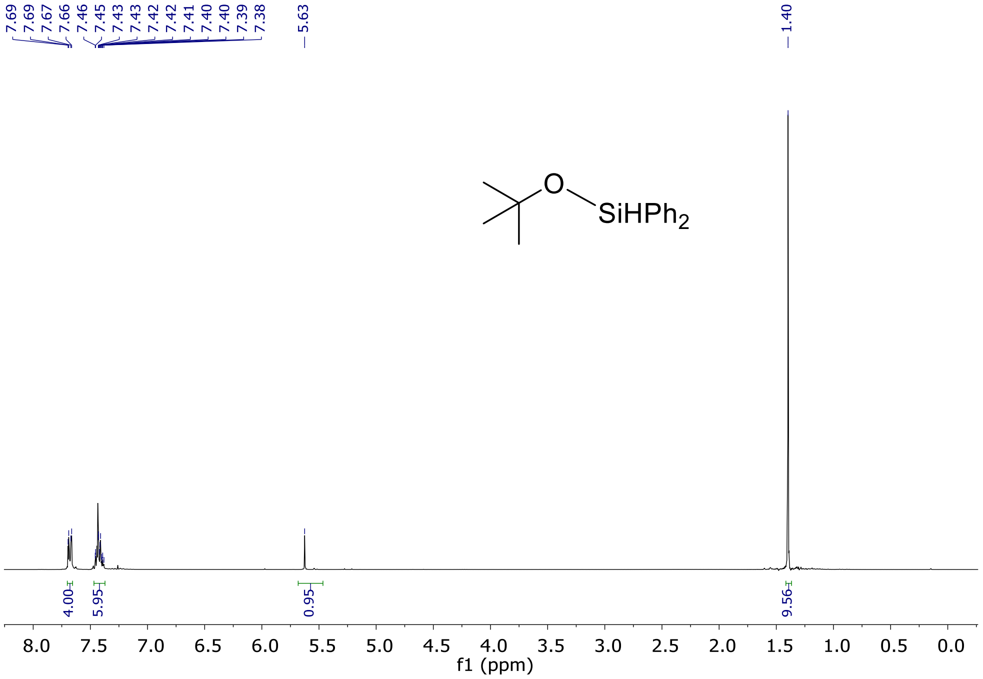

Figure S27. ^{1}H NMR spectrum (300 MHz, $CDCl_3$, 298 K) of *tert*-butoxydiphenylsilane.

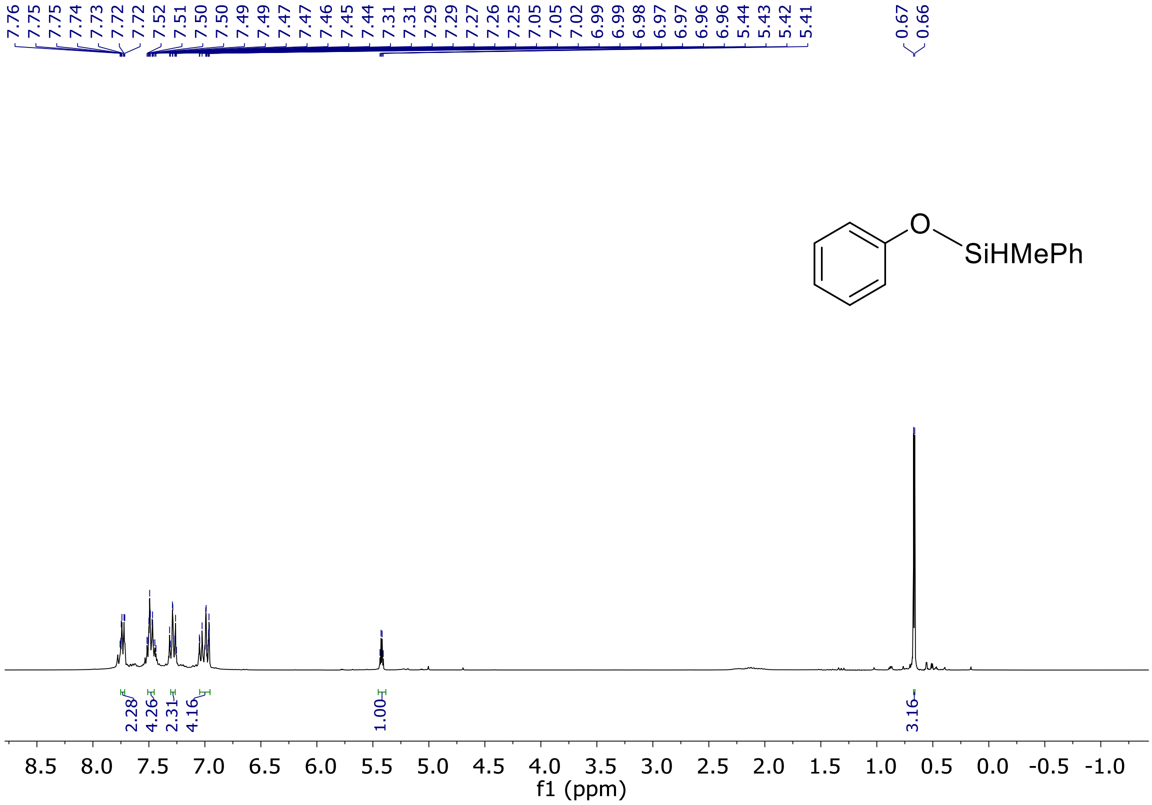

Figure S28. ^{1}H NMR spectrum (300 MHz, $CDCl_3$, 298 K) of phenoxymethylphenylsilane.

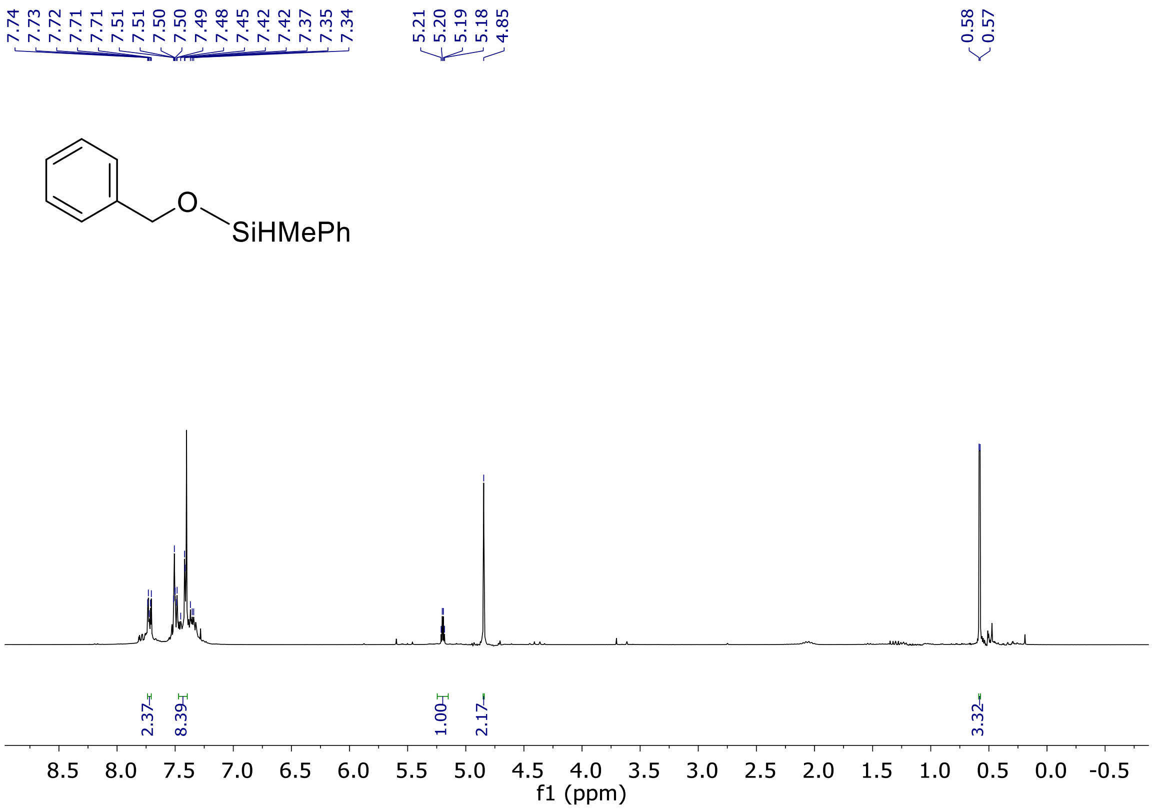

Figure S29. ^{1}H NMR spectrum (300 MHz, $CDCl_3$, 298 K) of (benzyloxy)methylphenylsilane.

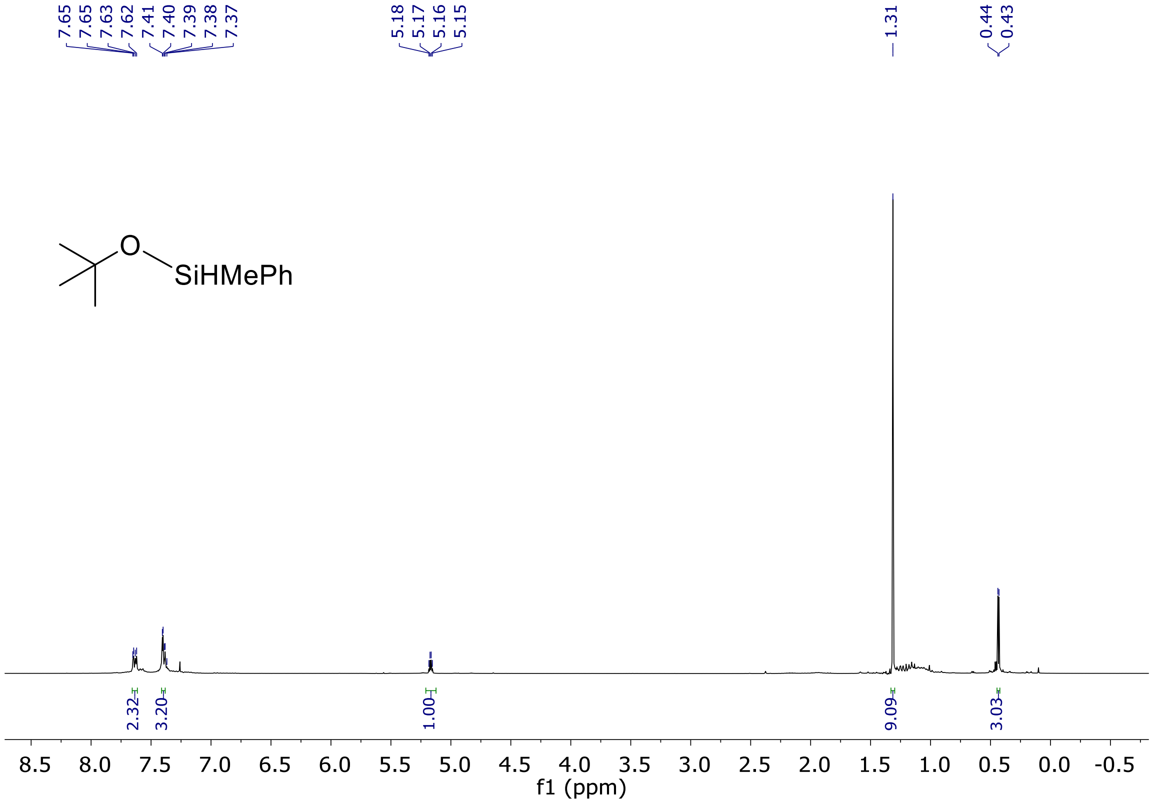

Figure S30. ^{1}H NMR spectrum (300 MHz, $CDCl_3$, 298 K) of *tert*-butoxymethyphenylsilane.

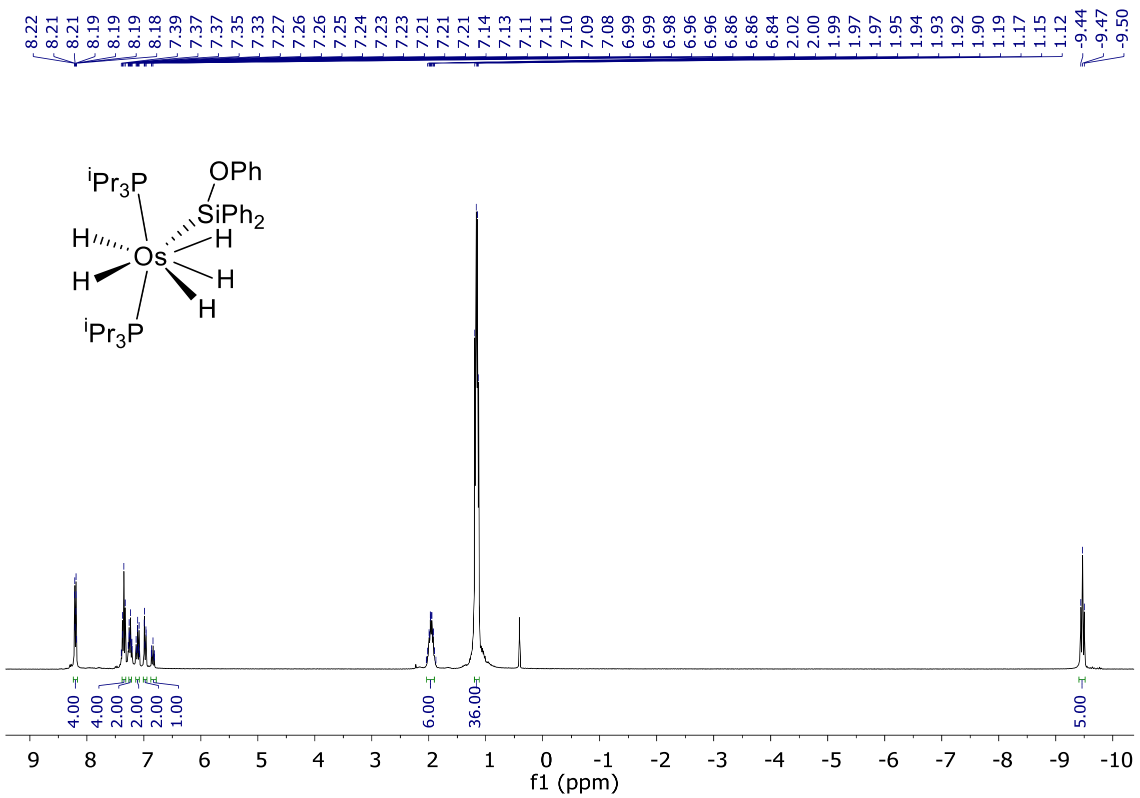

Figure S31. ^{1}H NMR spectrum (300 MHz, C_6D_6, 298 K) of complex **3**.

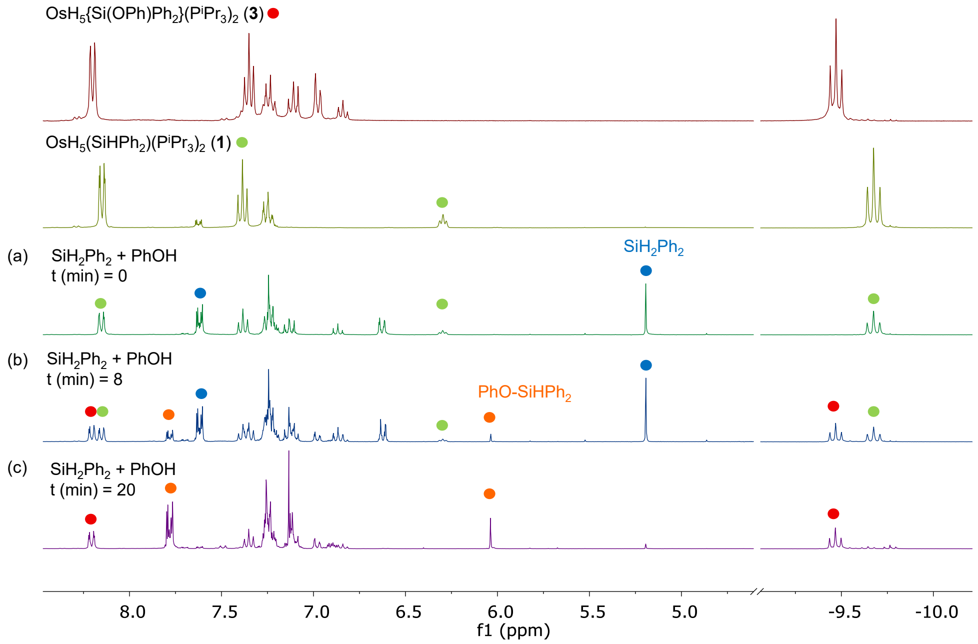

Figure S32. ^{1}H NMR spectra (300 MHz, C_6D_6, 298 K) of the reaction of monoalcoholysis of phenol (8.2 mg, 0.087mmol) with H_2SiPh_2 (10.2 µL, 0.087 mmol) with 20 mol % of catalyst **1** recorded after 0 (a) 8 (b) and 20 (c) min of reaction at 80 ºC. ^{1}H NMR spectra of complexes **3** and **1** are included on top of the Figure for comparative purposes. Selected NMR resonances of **1** (green spots), **3** (red spots), diphenylsilane (blue spots), and phenoxydiphenylsilane (orange spots).

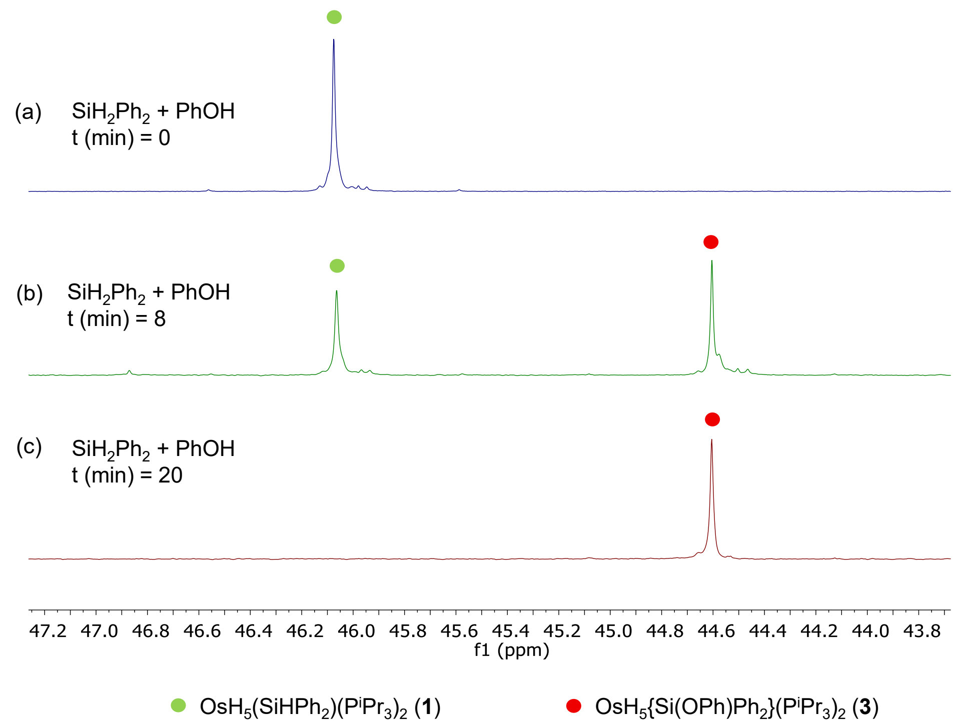

Figure S33. $^{31}P\{^{1}H\}$ NMR spectra (121.49 MHz, C_6D_6, 298 K) of the reaction of monoalcoholysis of phenol (8.2 mg, 0.087 mmol) with H_2SiPh_2 (10.2 µL, 0.087 mmol) with 20 mol % of catalyst **1** recorded before heating (a), after 8 (b), and 20 (c) min of reaction at 80 ºC. Resonance of **1** is denoted with a green spot, while that of **3** with a red spot.

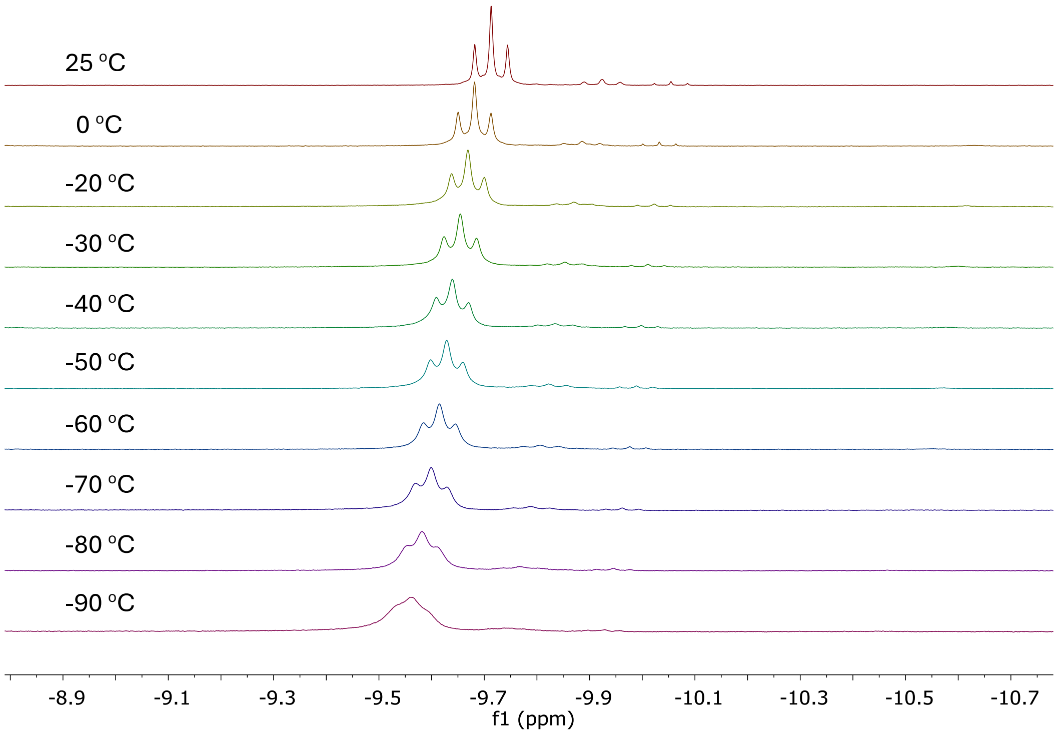

Figure S34. High field region of the ^{1}H NMR spectra (300.13 MHz, toluene-d_8) of complex **3** as a function of the temperature.

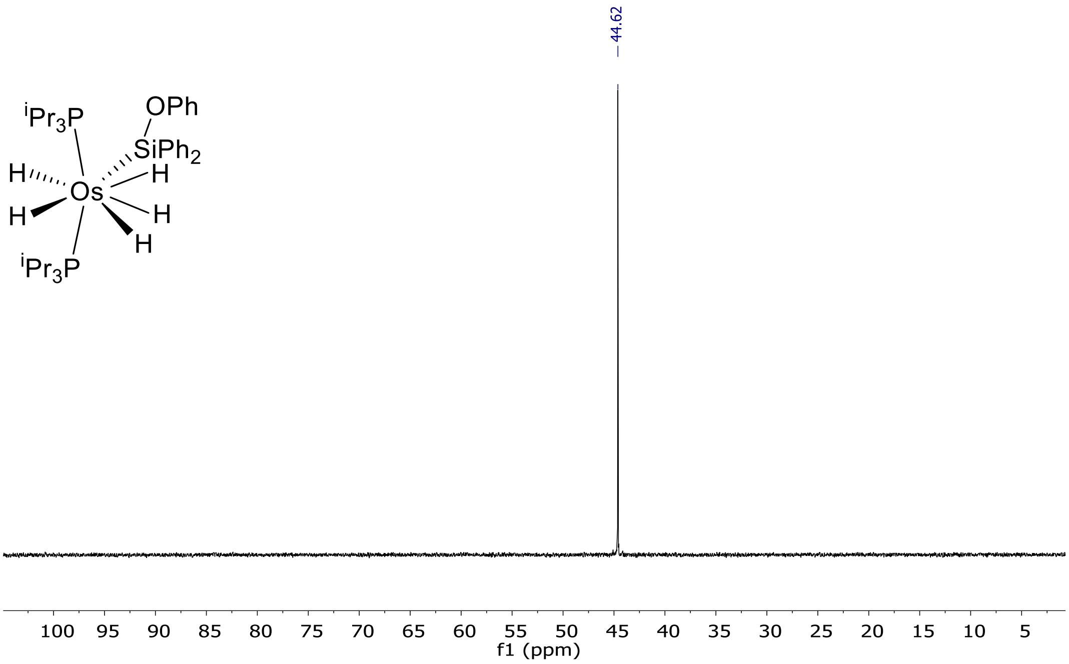

Figure S35. $^{31}P\{^1H\}$ NMR spectrum (121.49 MHz, C_6D_6, 298 K) of complex **3**.

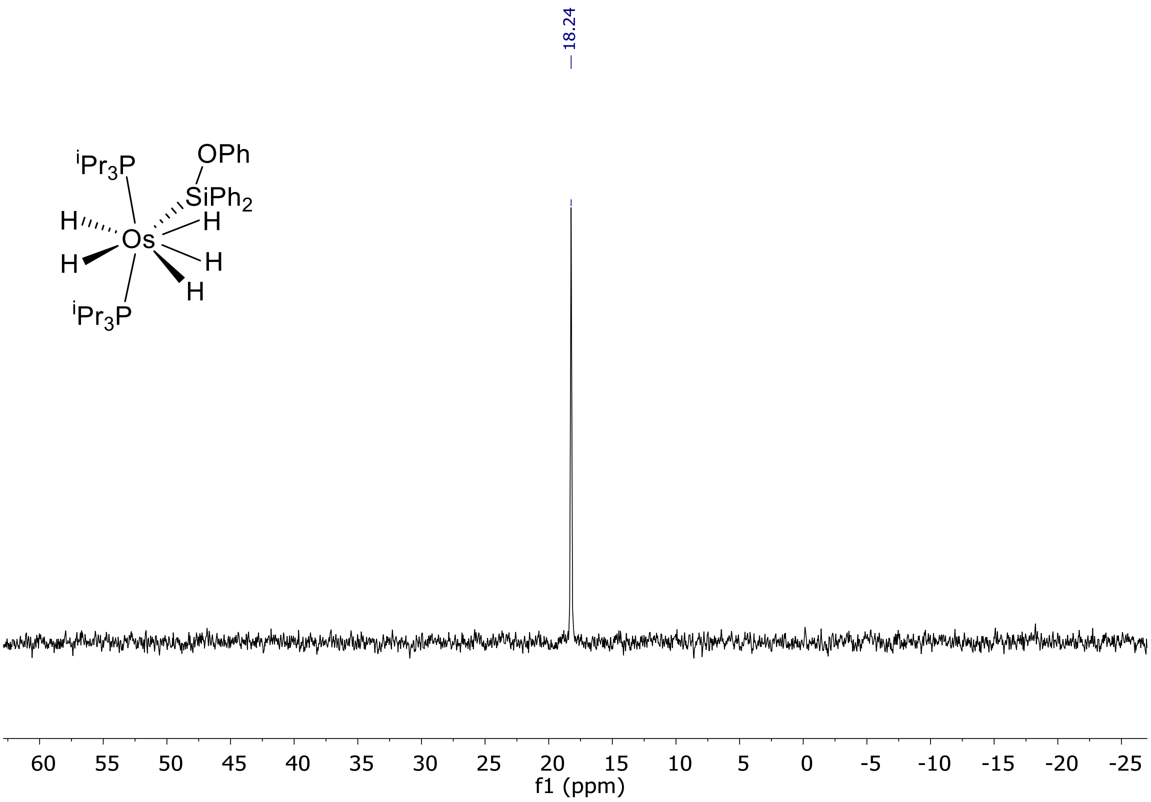

Figure S36. $^{29}Si\{^1H\}$ NMR spectrum (59.63 MHz, C_6D_6, 298 K) of complex **3**.

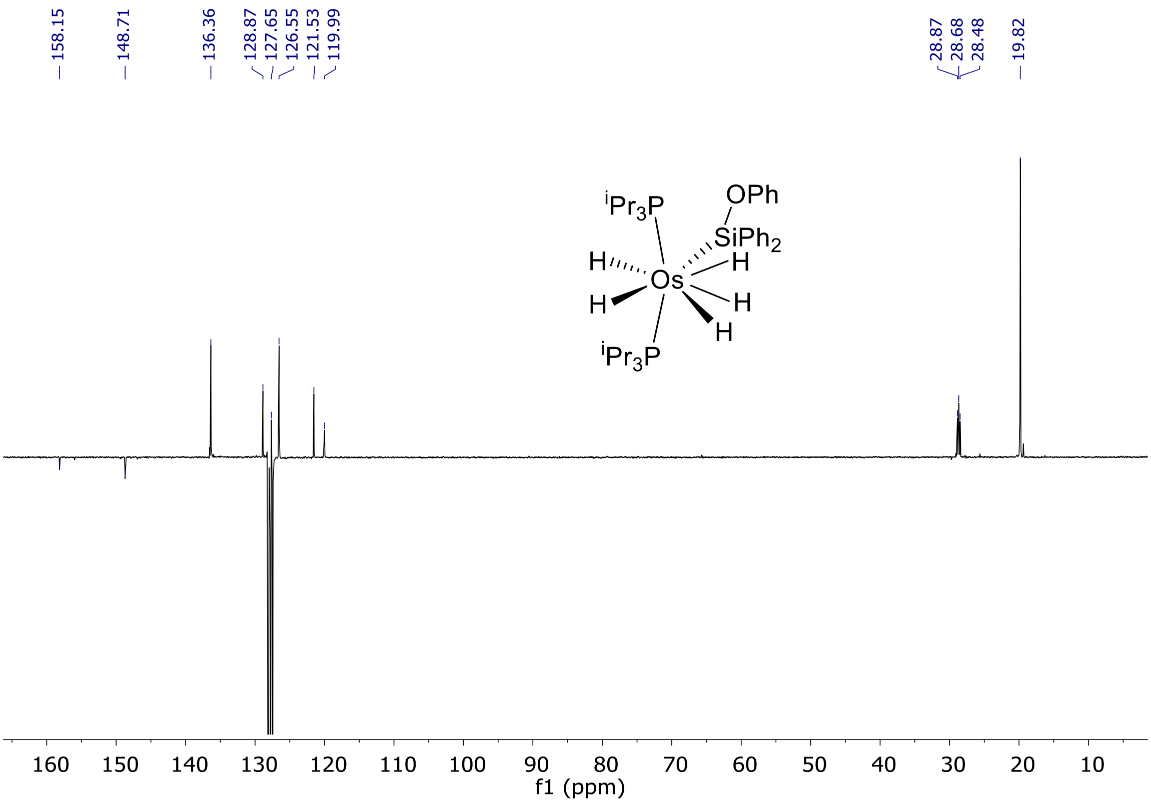

Figure S37. $^{13}C\{^{1}H\}$-apt NMR spectrum (75.429 MHz, C_6D_6, 298 K) of complex **3**.

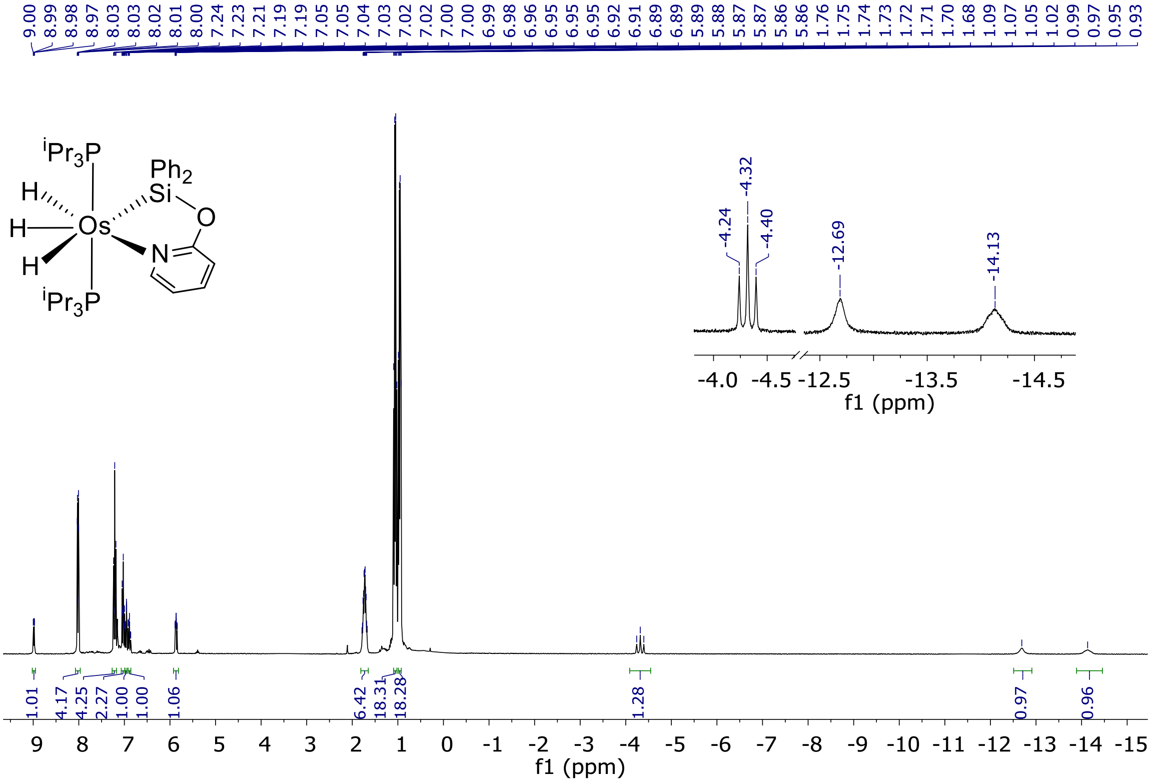

Figure S38. ^{1}H NMR spectrum (300 MHz, C_6D_6, 298 K) of complex **4**.

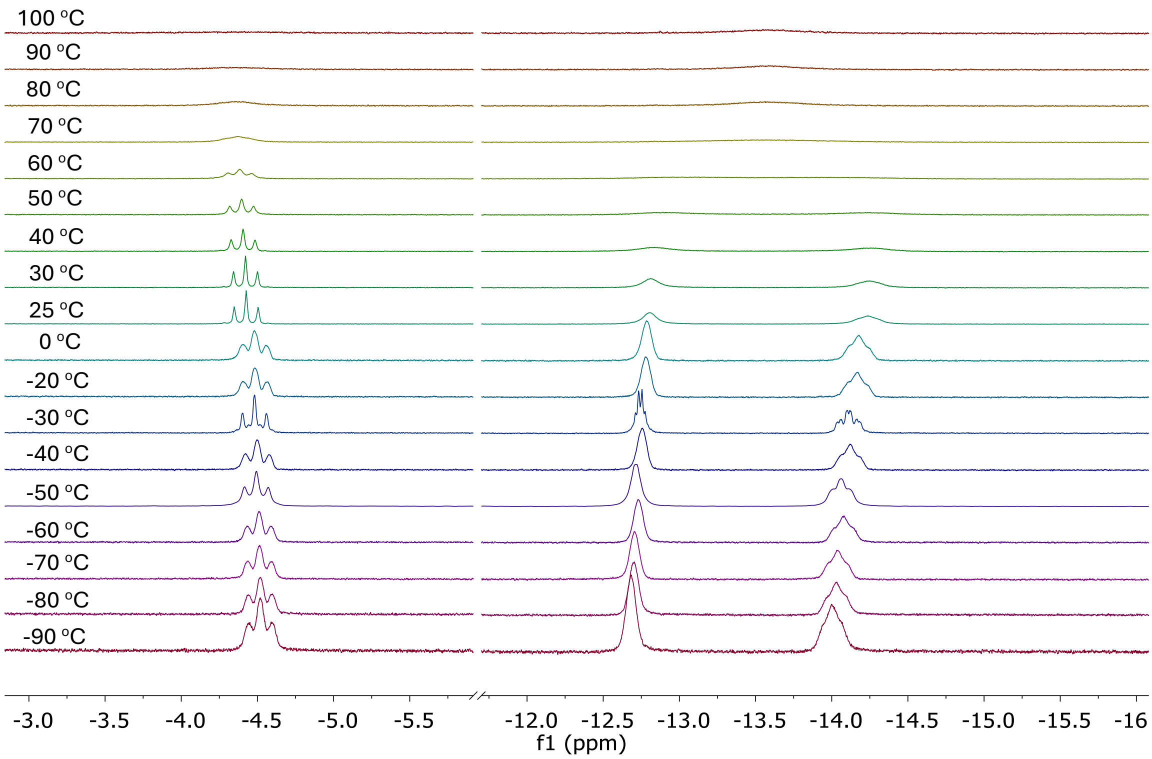

Figure S39. High field region of the ^{1}H NMR spectra (300.13 MHz, toluene-d_8) of compound **4** as a function of the temperature.

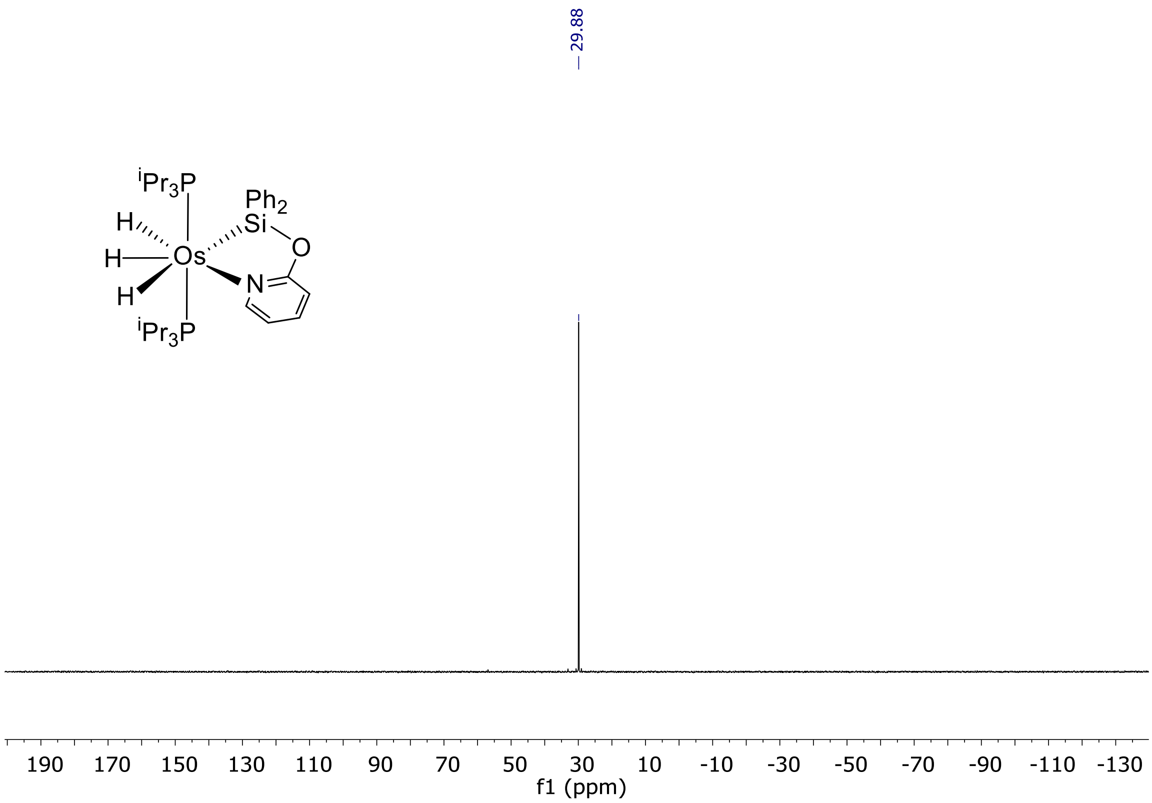

Figure S40. $^{31}P\{^{1}H\}$ NMR spectrum (121.49 MHz, C_6D_6, 298 K) of complex **4**.

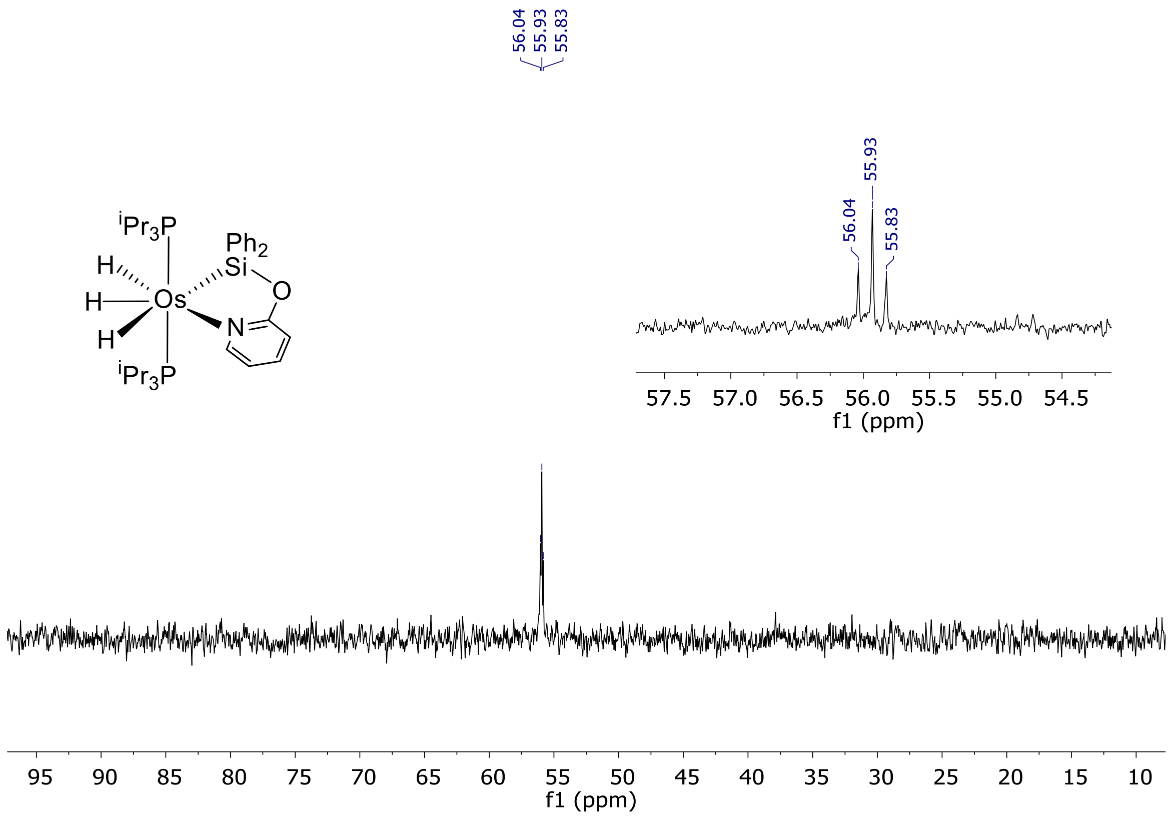

Figure S41. $^{29}Si\{^1H\}$ NMR spectrum (59.63 MHz, C_6D_6, 298 K) of complex **4**.

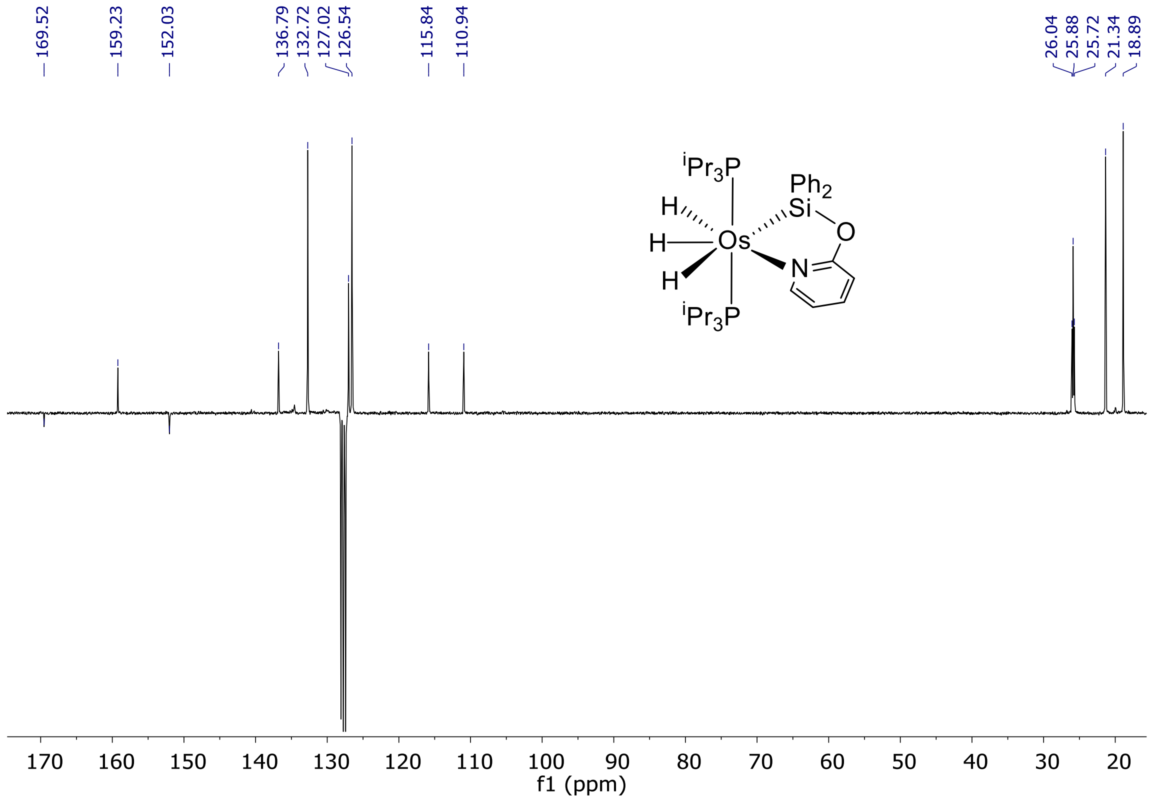

Figure S42. $^{13}C\{^1H\}$-apt NMR spectrum (75.429 MHz, C_6D_6, 298 K) of complex **4**.

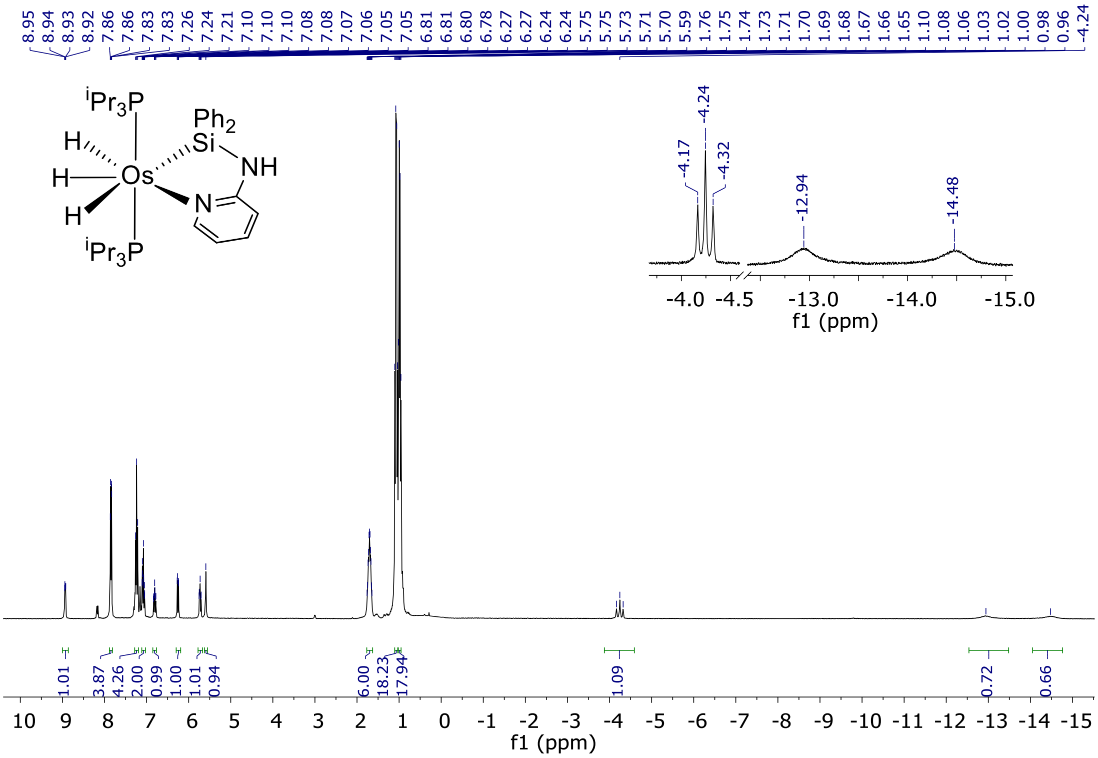

Figure S43. ^{1}H NMR spectrum (300 MHz, C_6D_6, 298 K) of complex **5**.

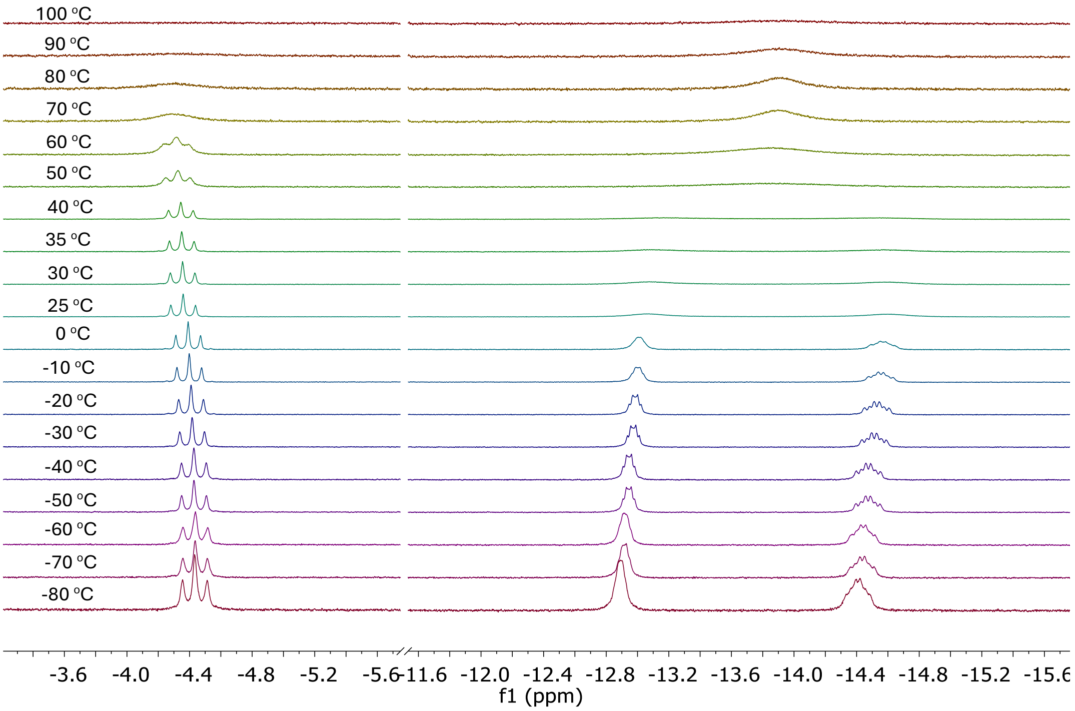

Figure S44. High field region of the ^{1}H NMR spectra (300.13 MHz, toluene-d_8) of compound **5** as a function of the temperature.

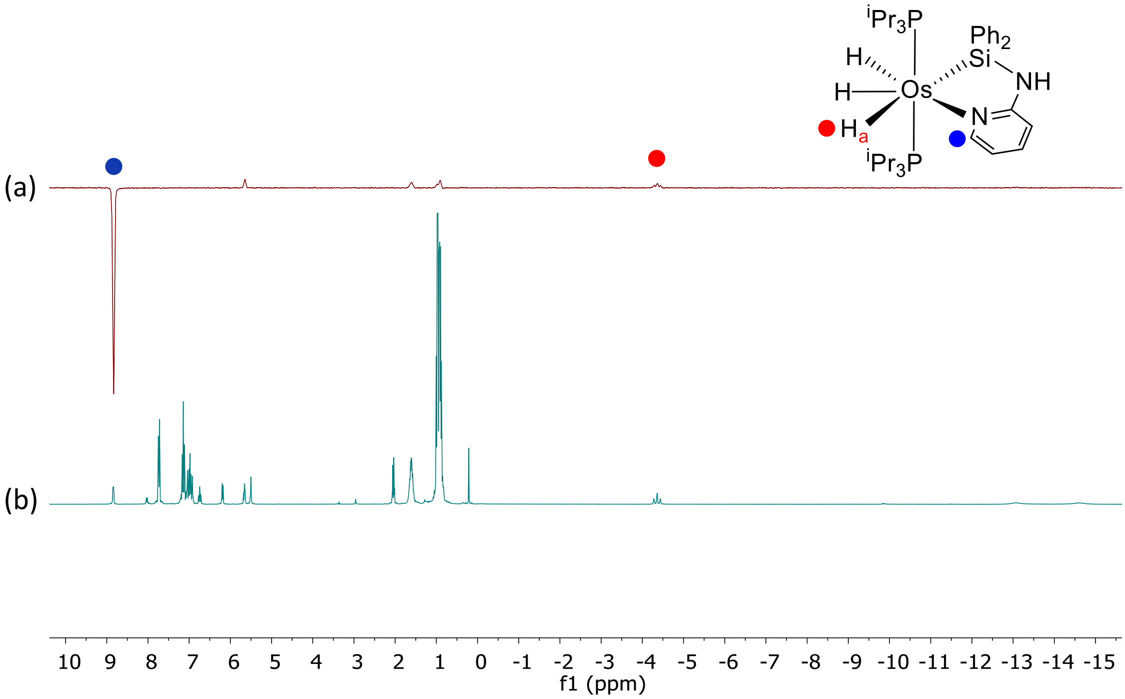

Figure S45. (a) One-dimensional ^{1}H selective NOESY experiment (d8 = 500 ms) and (b) ^{1}H NMR spectrum (300 MHz, toluene-d_8, 298 K) of compound **5**.

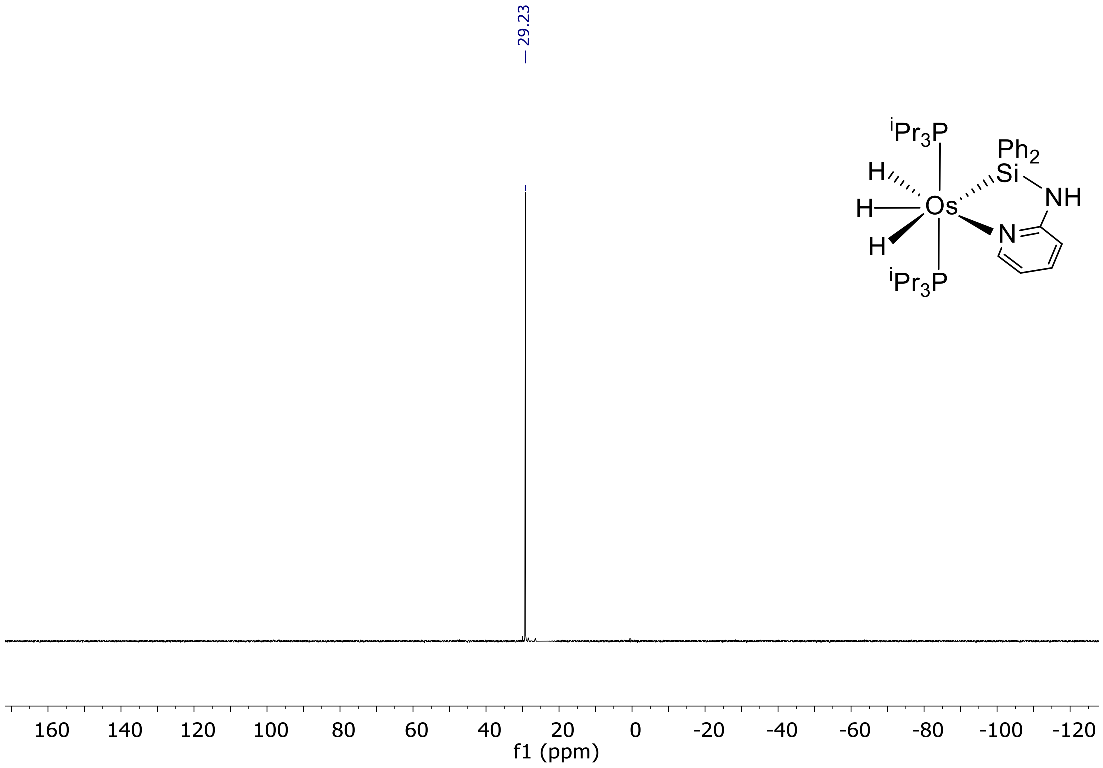

Figure S46. ^{31}P{^{1}H} NMR spectrum (121.49 MHz, C_6D_6, 298 K) of complex **5**.

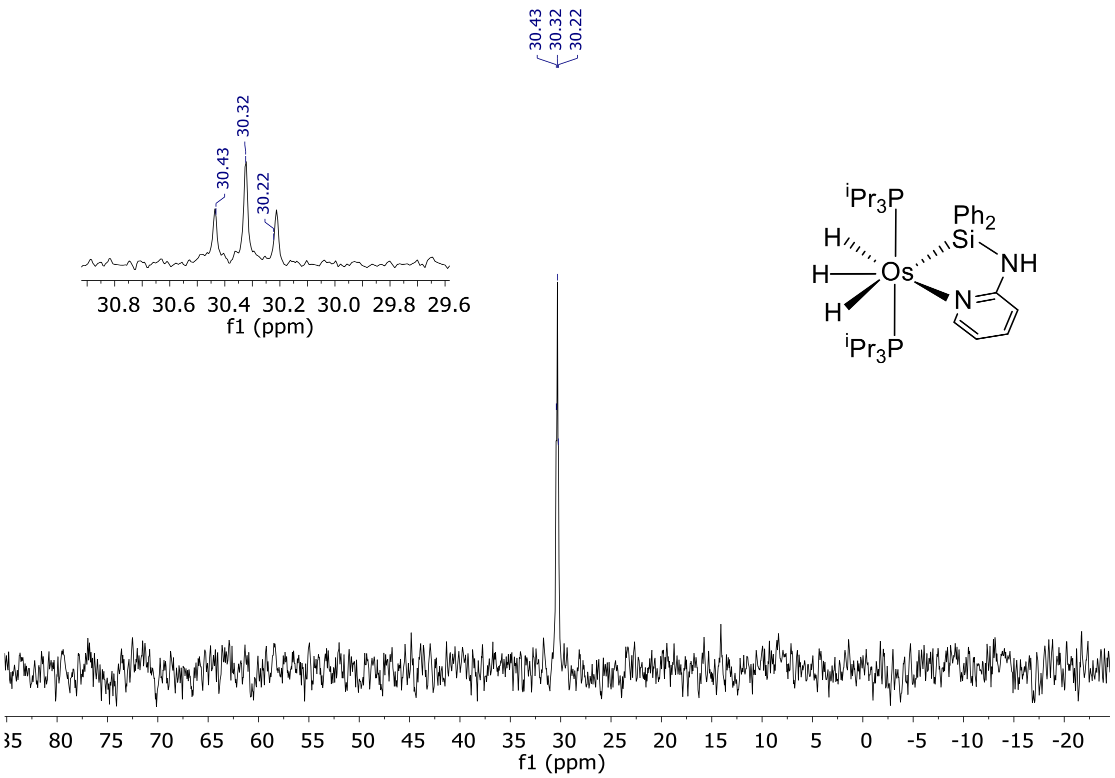

Figure S47. $^{29}Si\{^{1}H\}$ NMR spectrum (59.63 MHz, C_6D_6, 298 K) of complex **5**.

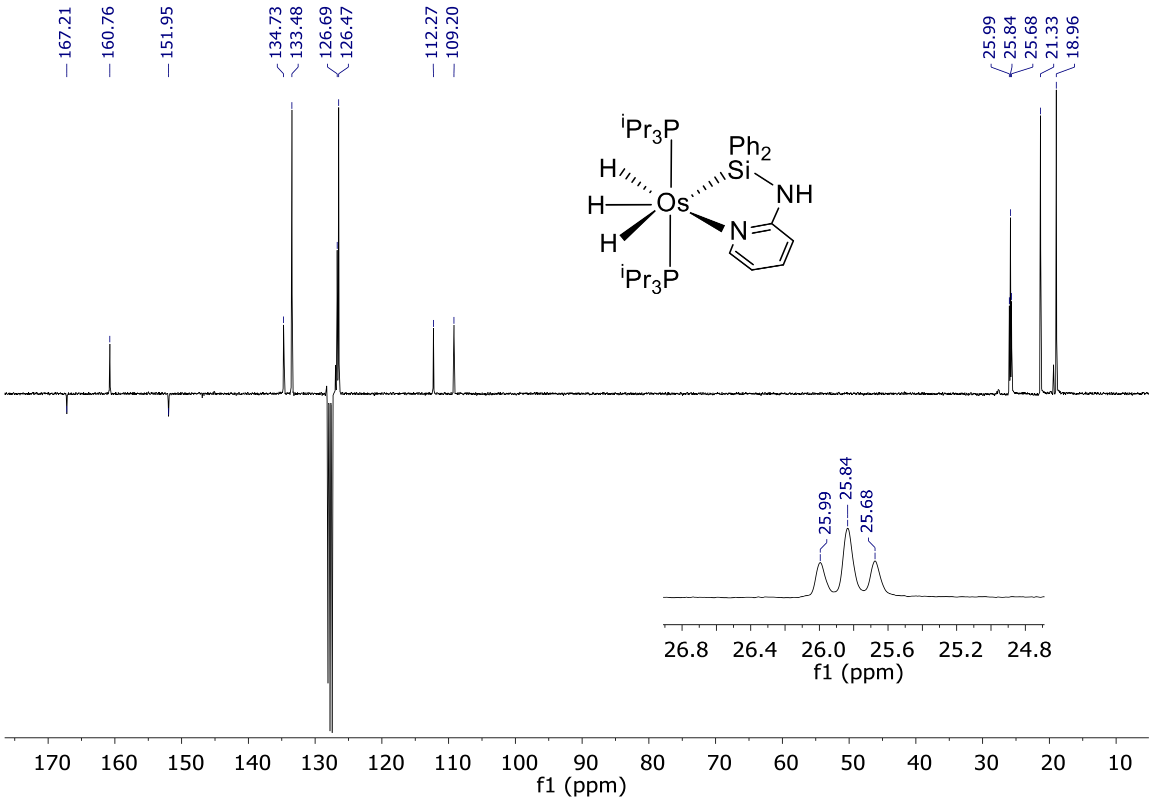

Figure S48. $^{13}C\{^{1}H\}$-apt NMR spectrum (75.429 MHz, C_6D_6, 298 K) of complex **5**.

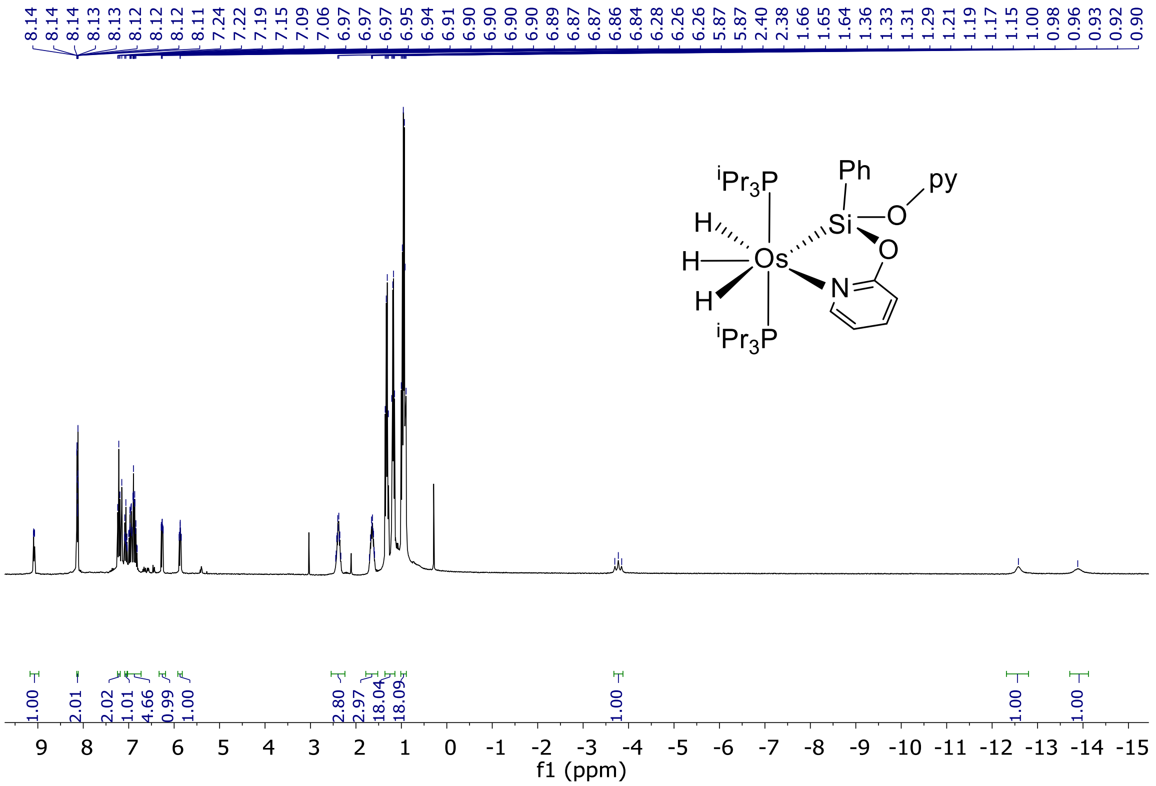

Figure S49. ^{1}H NMR spectrum (300 MHz, C_6D_6, 298 K) of complex **6**.

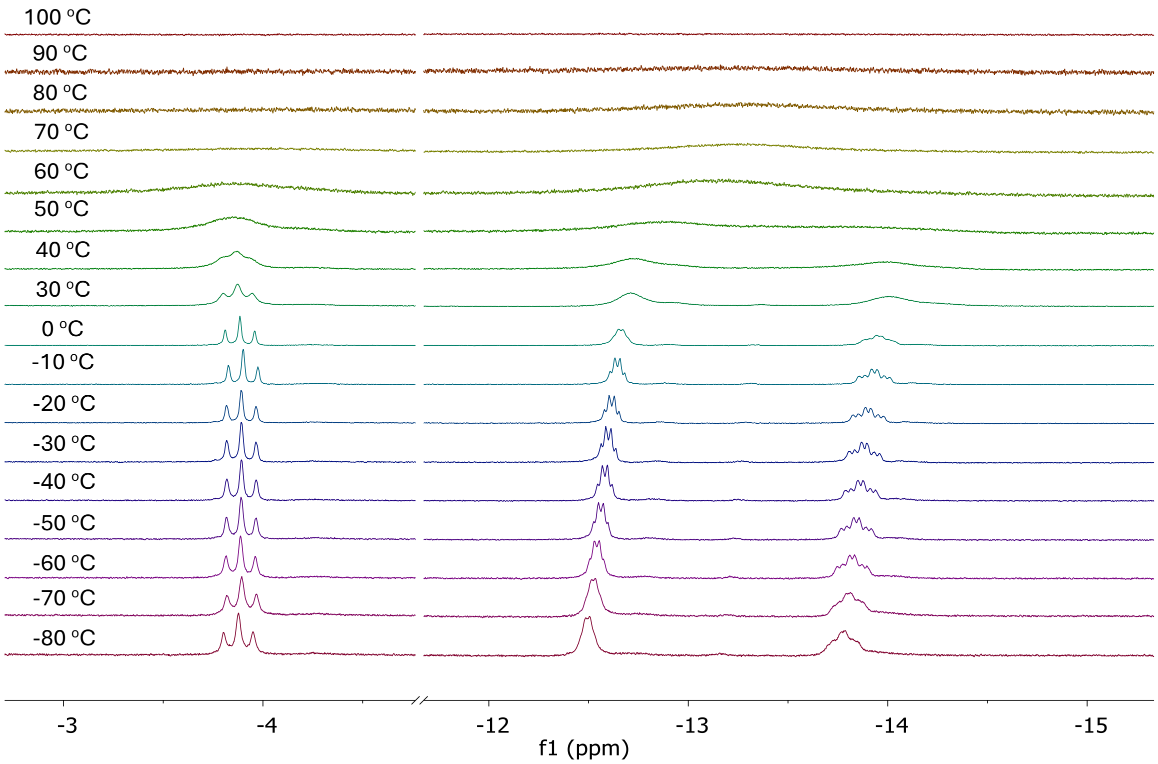

Figure S50. High field region of the ^{1}H NMR spectra (300.13 MHz, toluene-d_8) of compound **6** as a function of the temperature.

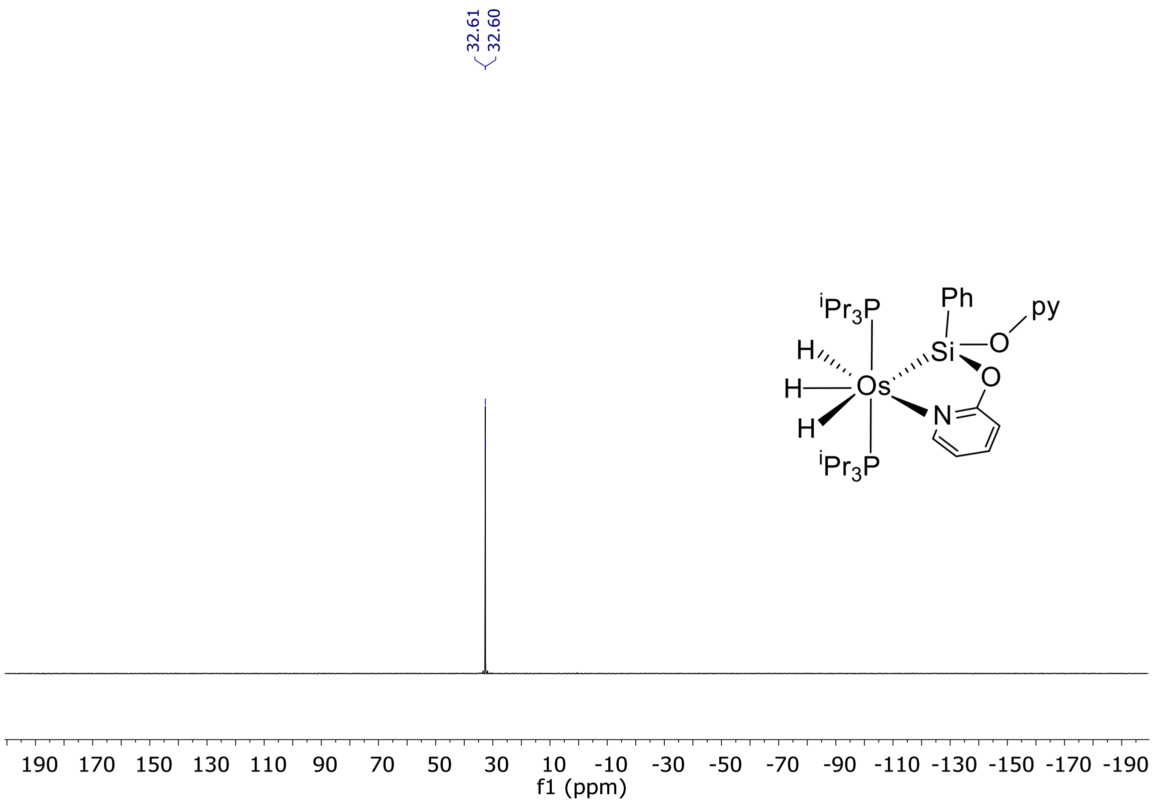

Figure S51. $^{31}P\{^{1}H\}$ NMR spectrum (121.49 MHz, C_6D_6, 298 K) of complex **6**.

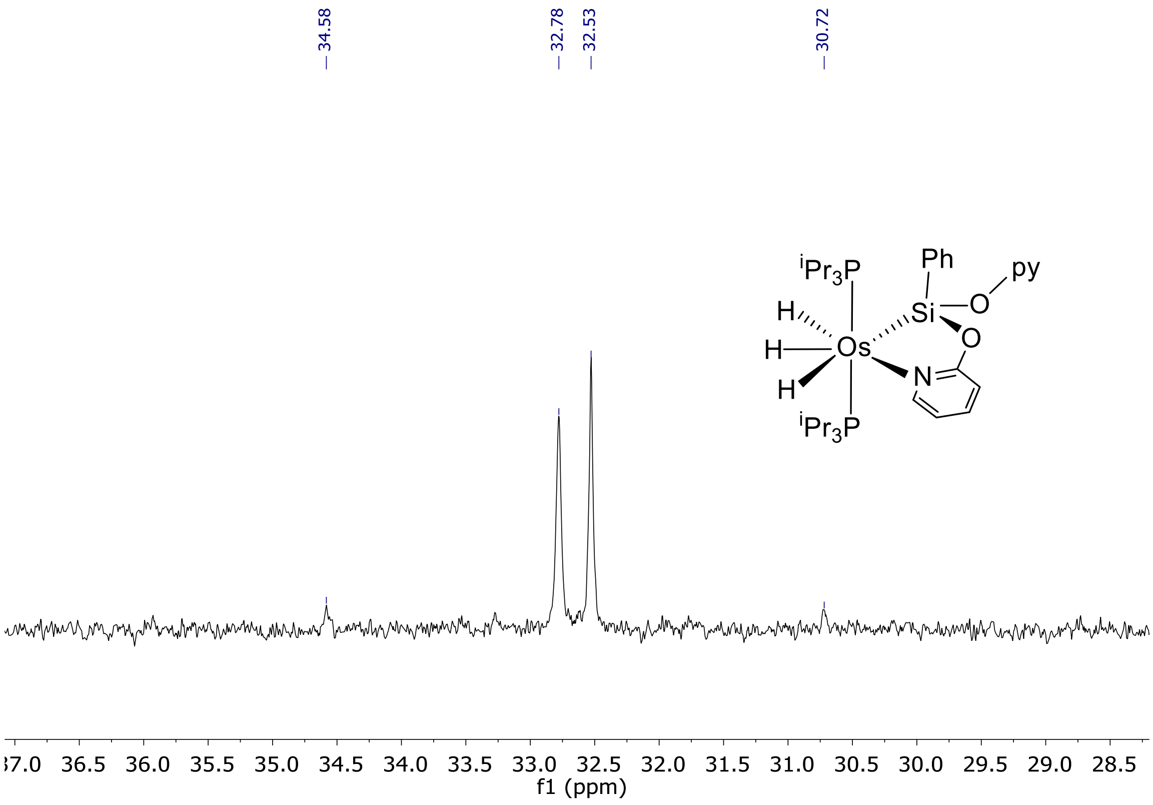

Figure S52. $^{31}P\{^{1}H\}$ NMR spectrum (121.49 MHz, toluene-d_8, 253 K) of complex **6**.

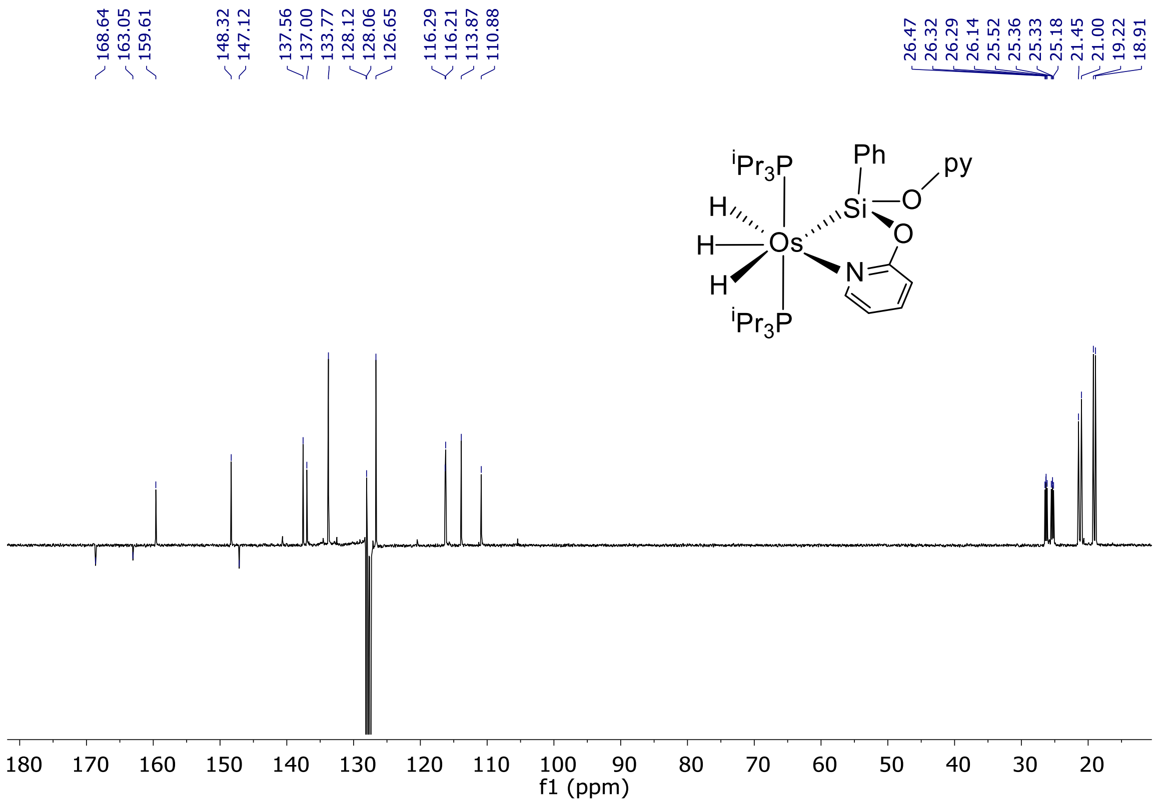

Figure S53. $^{13}C\{^{1}H\}$-apt NMR spectrum (75.429 MHz, C_6D_6, 298 K) of complex **6**.

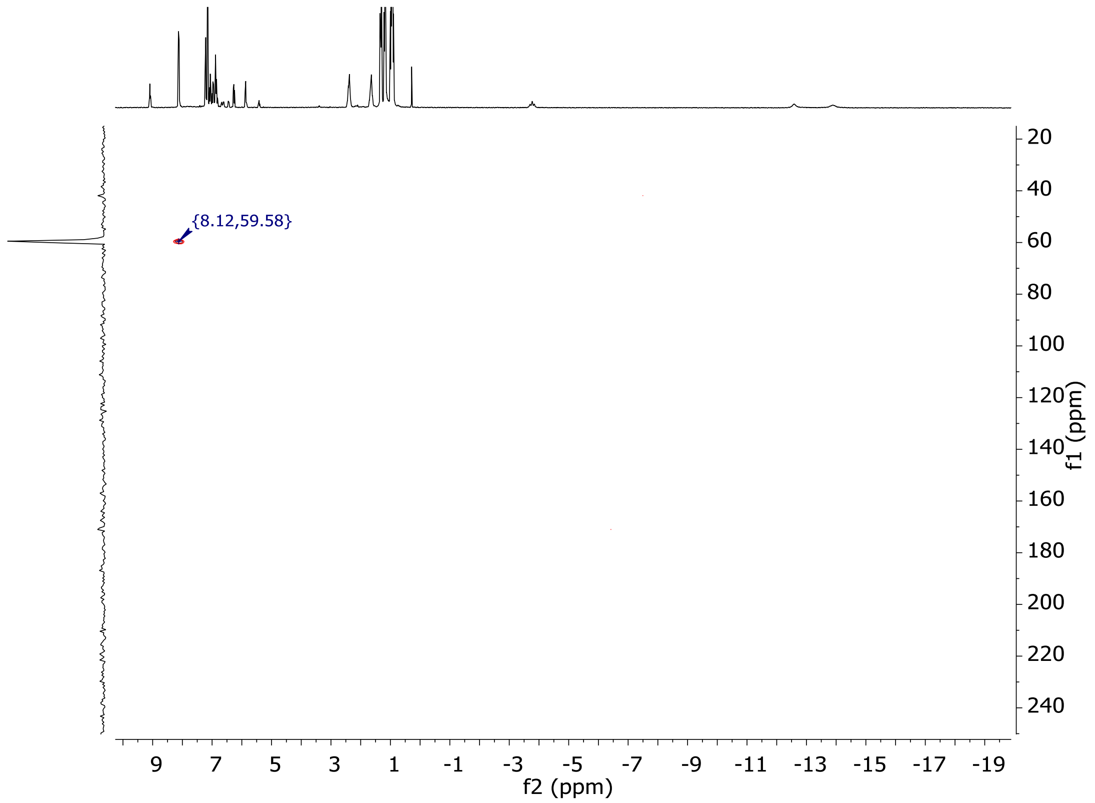

Figure S54. ^{1}H(300 MHz)-^{29}Si(59.63 MHz) HMBC 2D correlation NMR spectrum (C_6D_6, 298 K) of complex **6**.

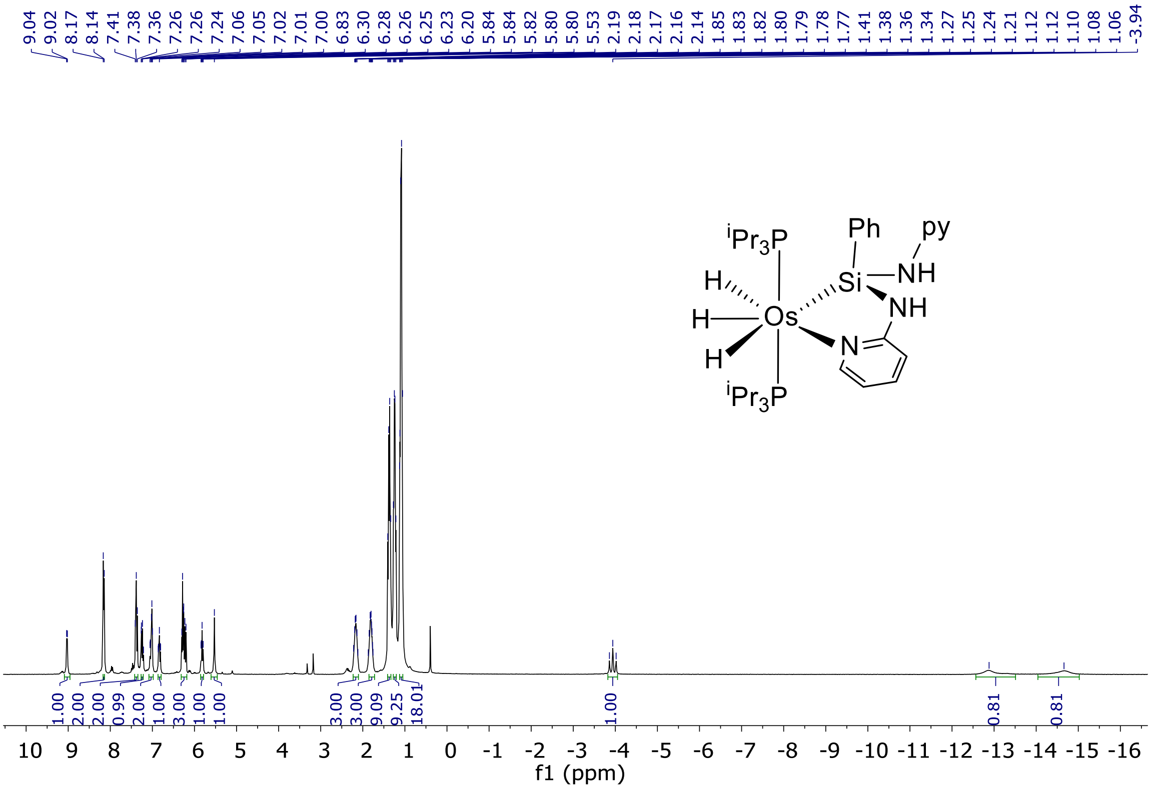

Figure S55. ^{1}H NMR spectrum (300 MHz, C_6D_6, 298 K) of complex **7**.

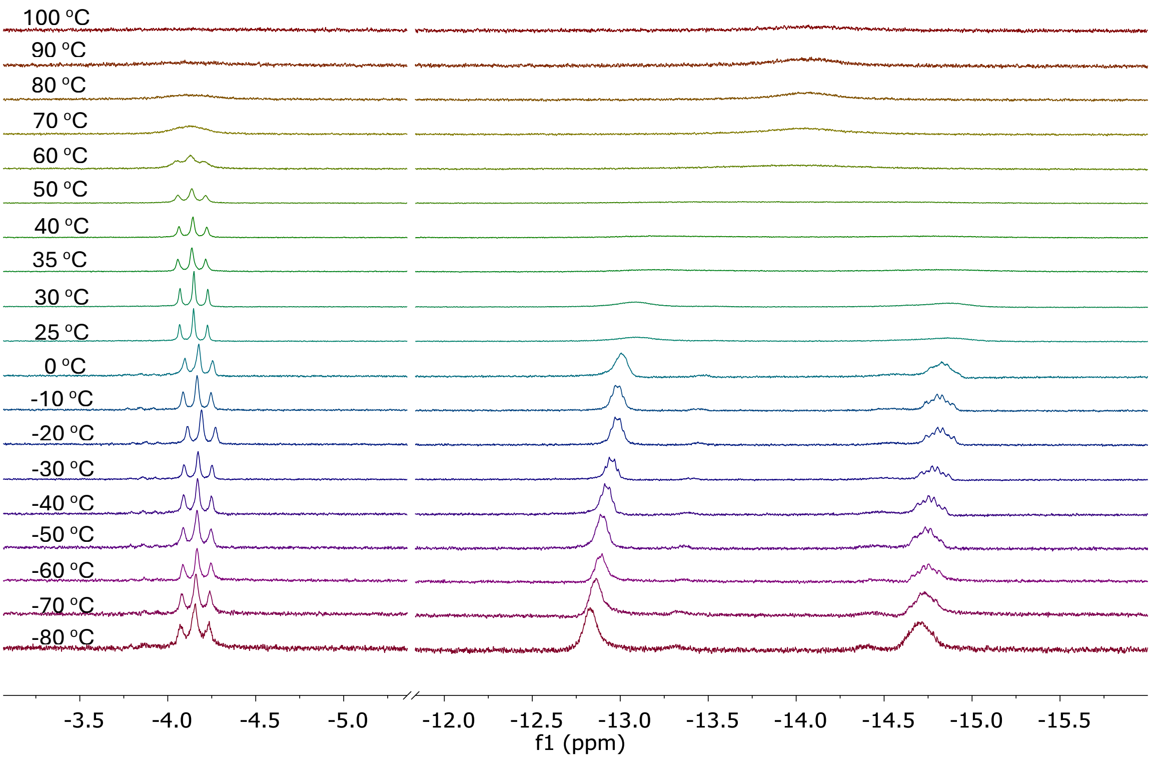

Figure S56. High field region of the ^{1}H NMR spectra (300.13 MHz, toluene-d_8) of compound **7** as a function of the temperature.

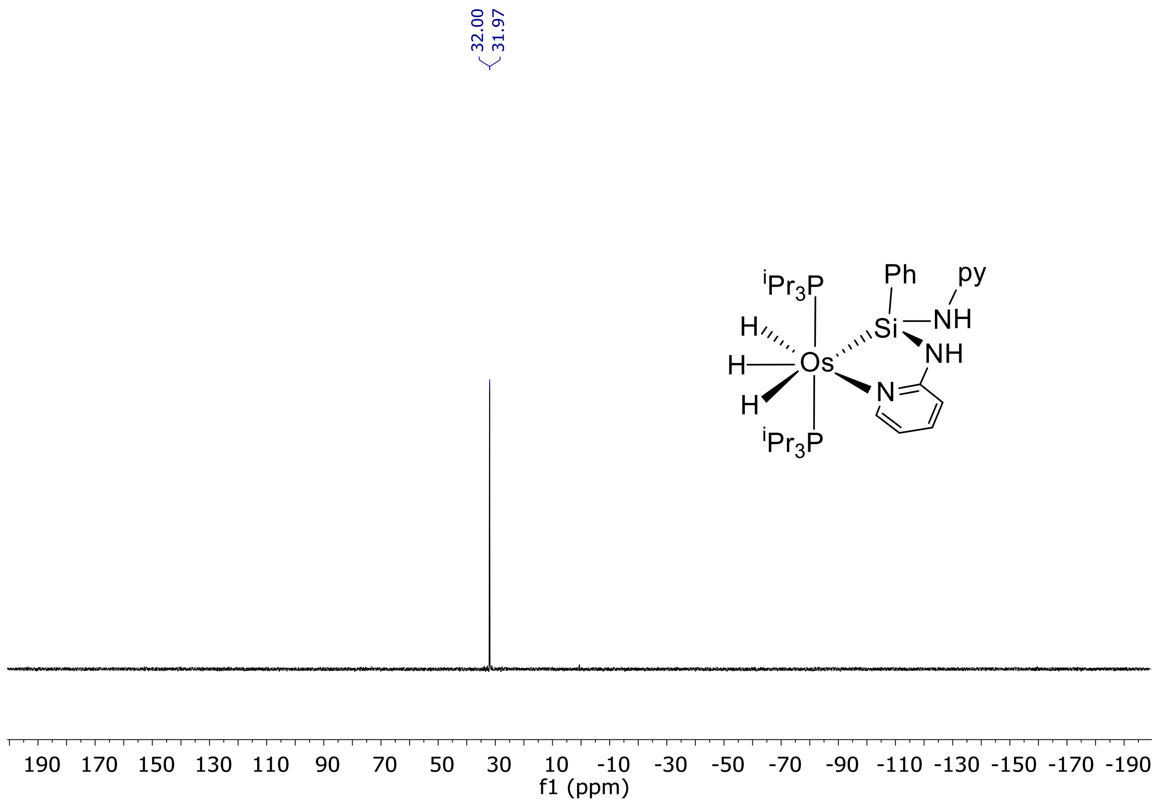

Figure S57. $^{31}P\{^{1}H\}$ NMR spectrum (121.49 MHz, C_6D_6, 298 K) of complex **7**.

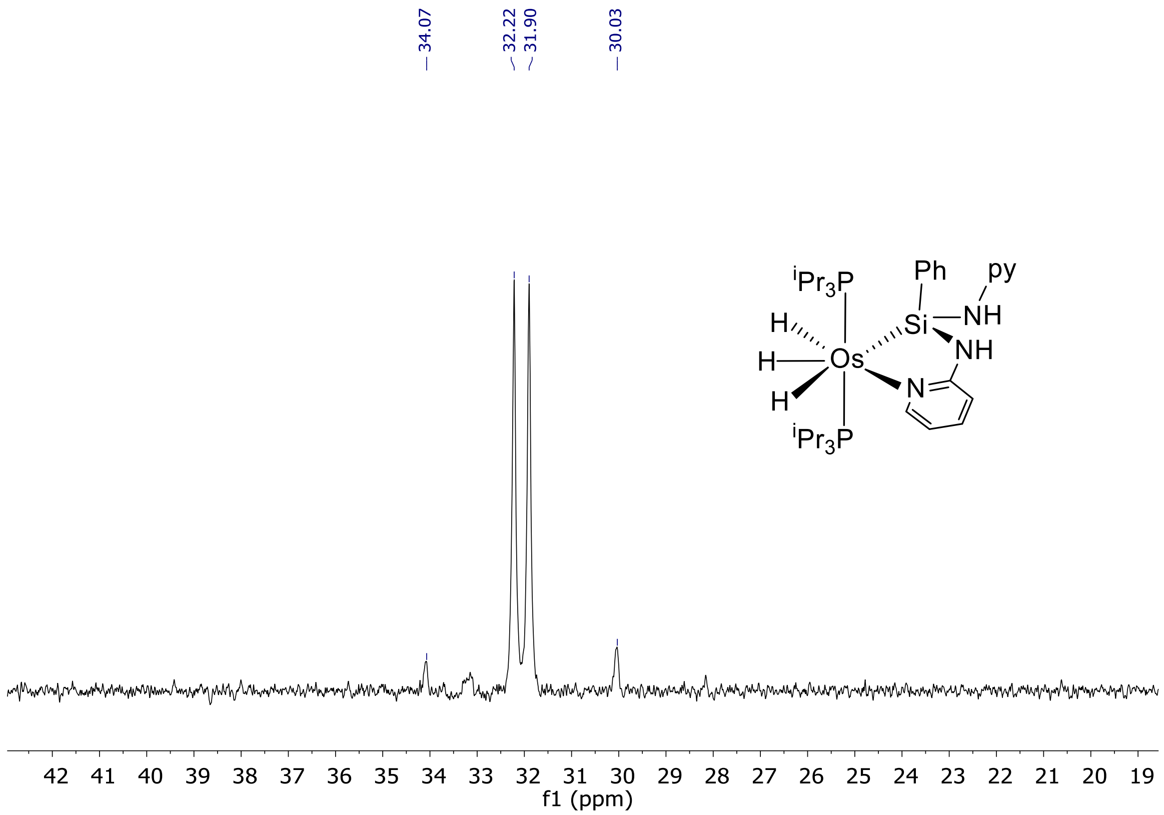

Figure S58. $^{31}P\{^{1}H\}$ NMR spectrum (121.49 MHz, toluene-d_8, 253 K) of complex **7.**

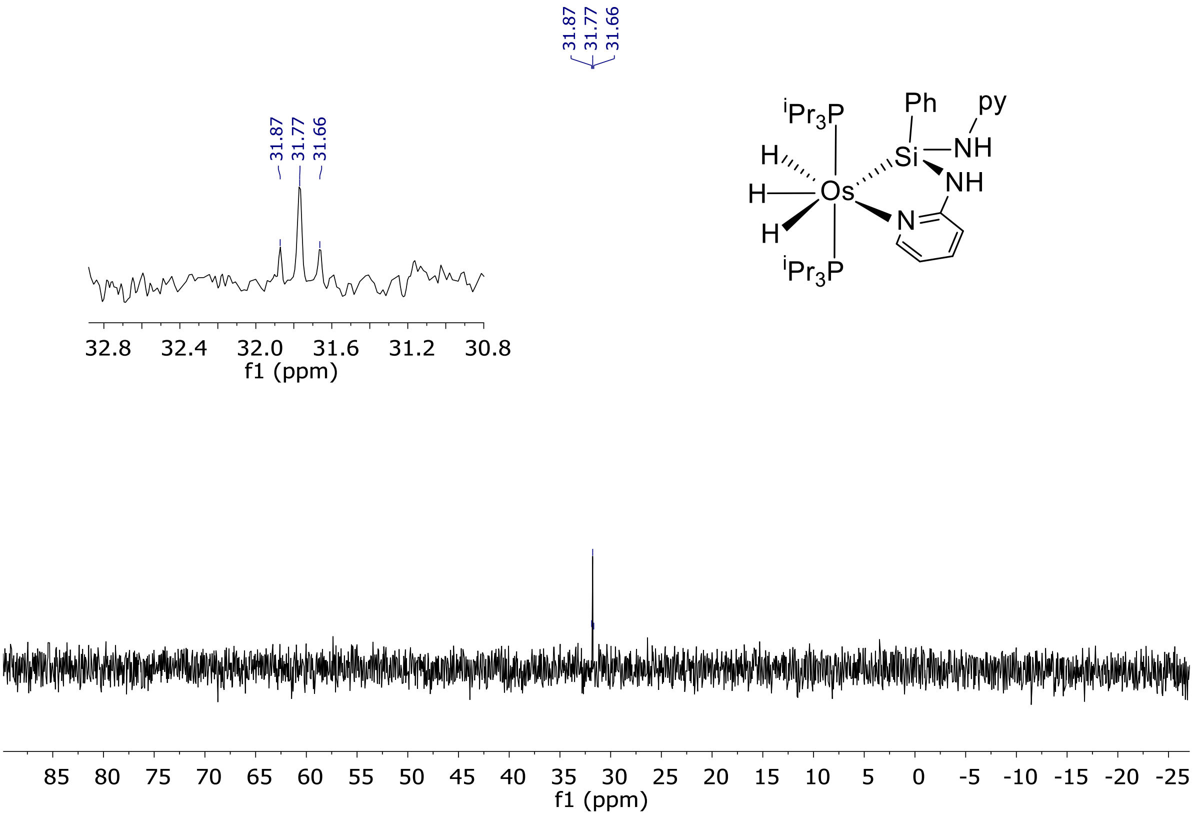

Figure S59. $^{29}Si\{^1H\}$ NMR spectrum (59.63 MHz, C_6D_6, 298 K) of complex **7**.

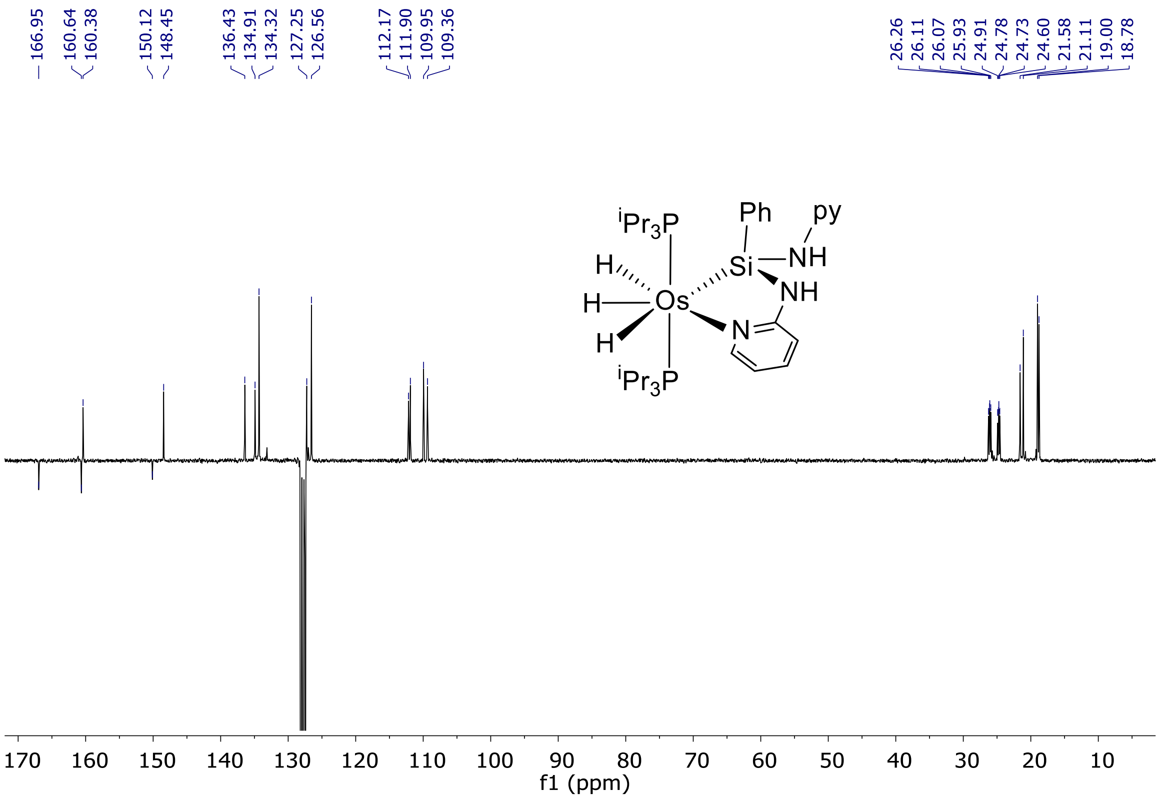

Figure S60. $^{13}C\{^1H\}$-apt NMR spectrum (75.429 MHz, C_6D_6, 298 K) of complex **7**.

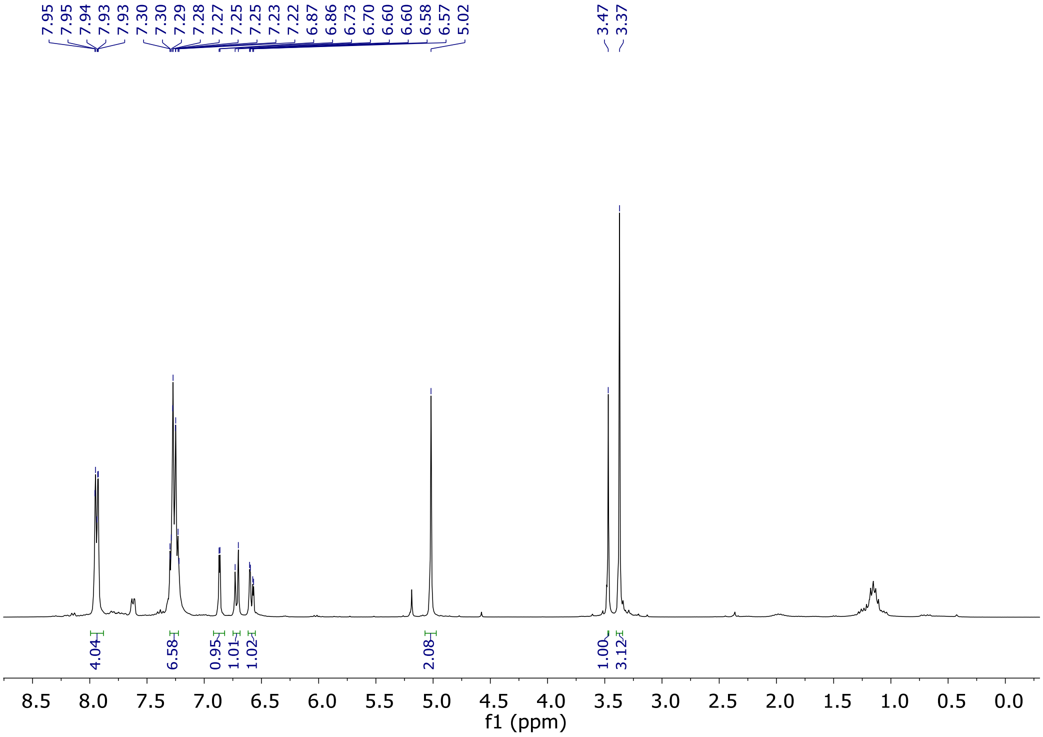

Figure S61. ^{1}H NMR spectrum (300 MHz, benzene-d_6, 298 K) of the crude reaction mixture of the tandem hydrosilylation/dehydrogenative silylation of 4-methoxy-2-hyddroxy-benzaldehyde with H_2SiPh_2 to give **8a**. Dioxane (δ 3.47 ppm; internal standard).

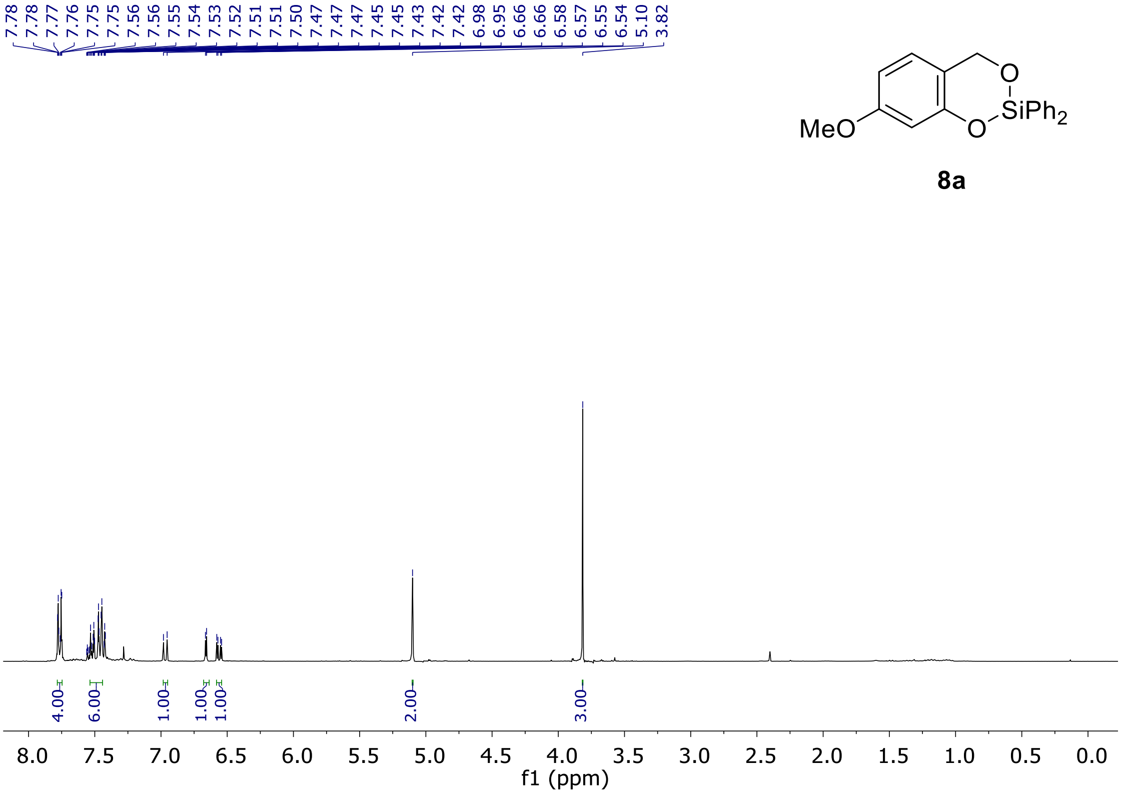

Figure S62. ^{1}H NMR spectrum (300 MHz, $CDCl_3$, 298 K) of **8a**.

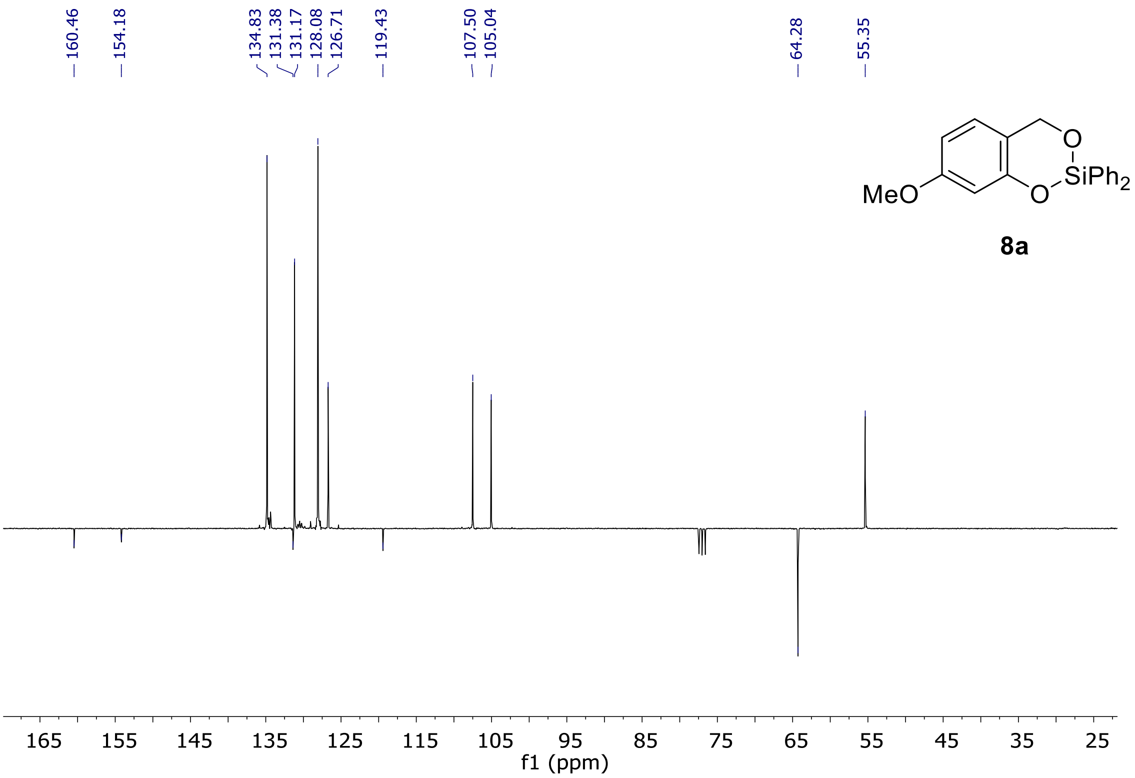

Figure S63. $^{13}C\{^1H\}$-apt NMR spectrum (75.429 MHz, C_6D_6, 298 K) of **8a**.

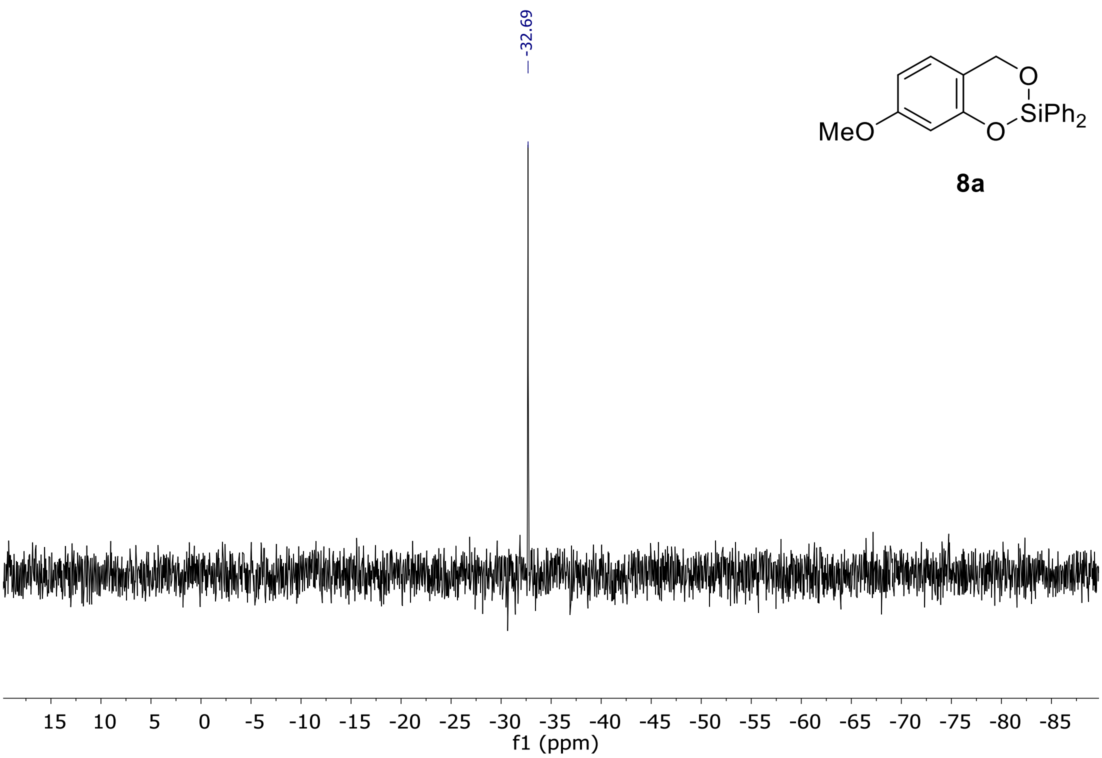

Figure S64. $^{29}Si\{^1H\}$ NMR spectrum (59.63 MHz, $CDCl_3$, 298 K) of **8a**.

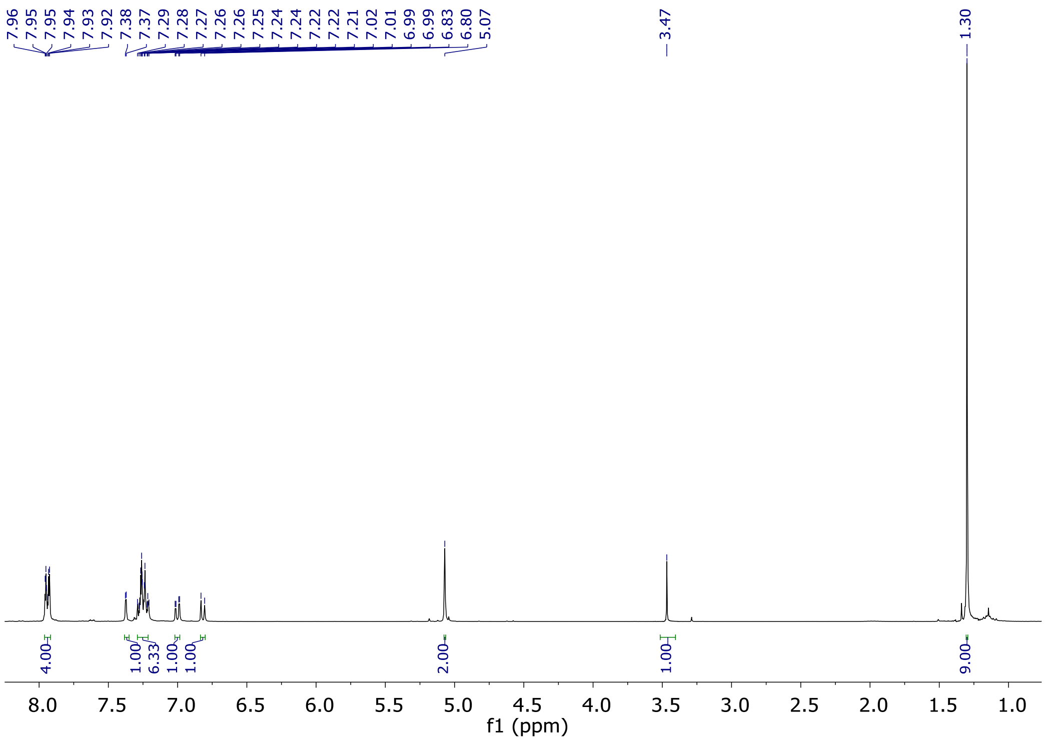

Figure S65. ^{1}H NMR spectrum (300 MHz, benzene-d_6, 298 K) of the crude reaction mixture of the tandem hydrosilylation/dehydrogenative silylation of 4-*t*-butyl-2-hydroxybenzaldehyde with H_2SiPh_2 to give **8b**. Dioxane (δ 3.47 ppm; internal standard).

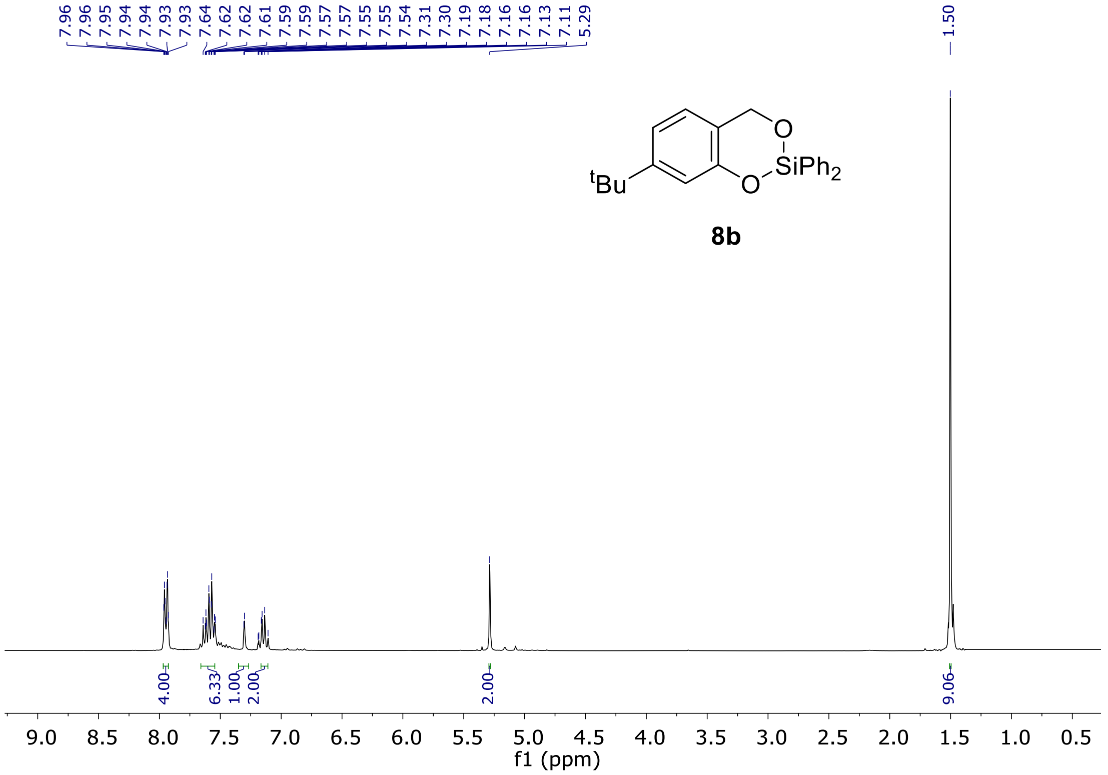

Figure S66. ^{1}H NMR spectrum (300 MHz, $CDCl_3$, 298 K) of **8b**.

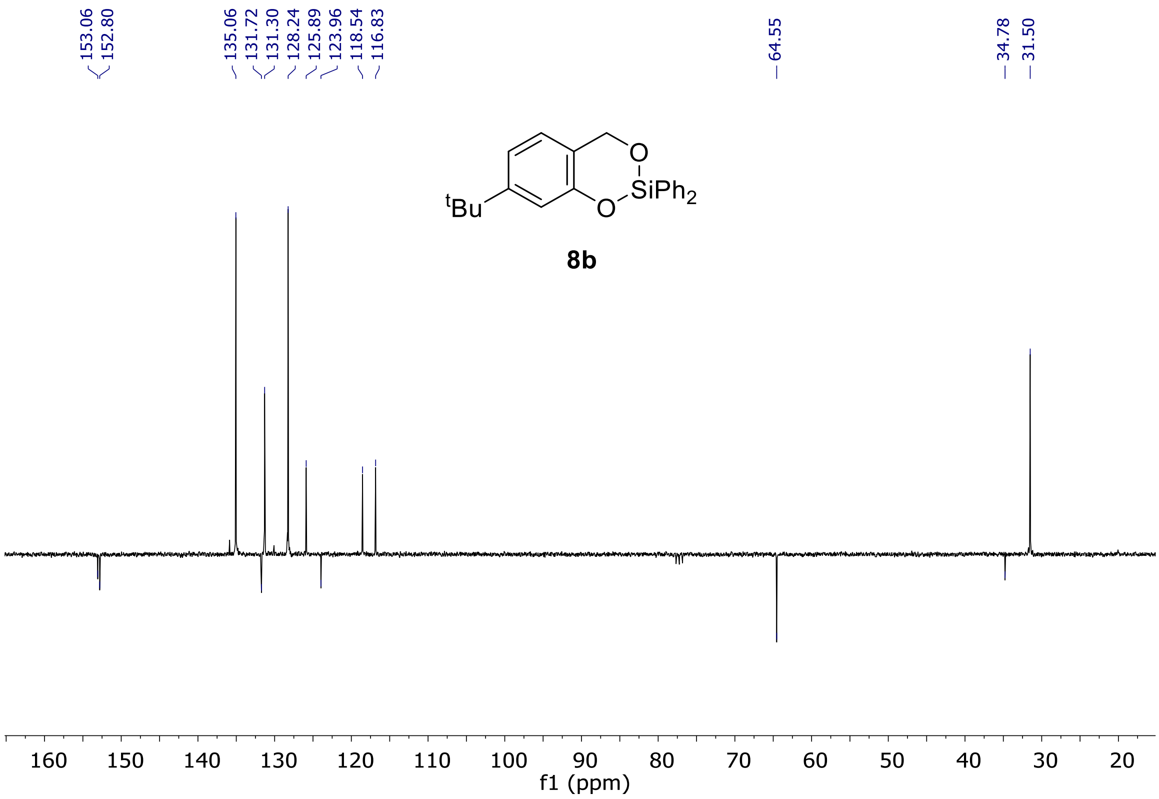

Figure S67. $^{13}C\{^{1}H\}$-apt NMR spectrum (75.429 MHz, C_6D_6, 298 K) of **8b**.

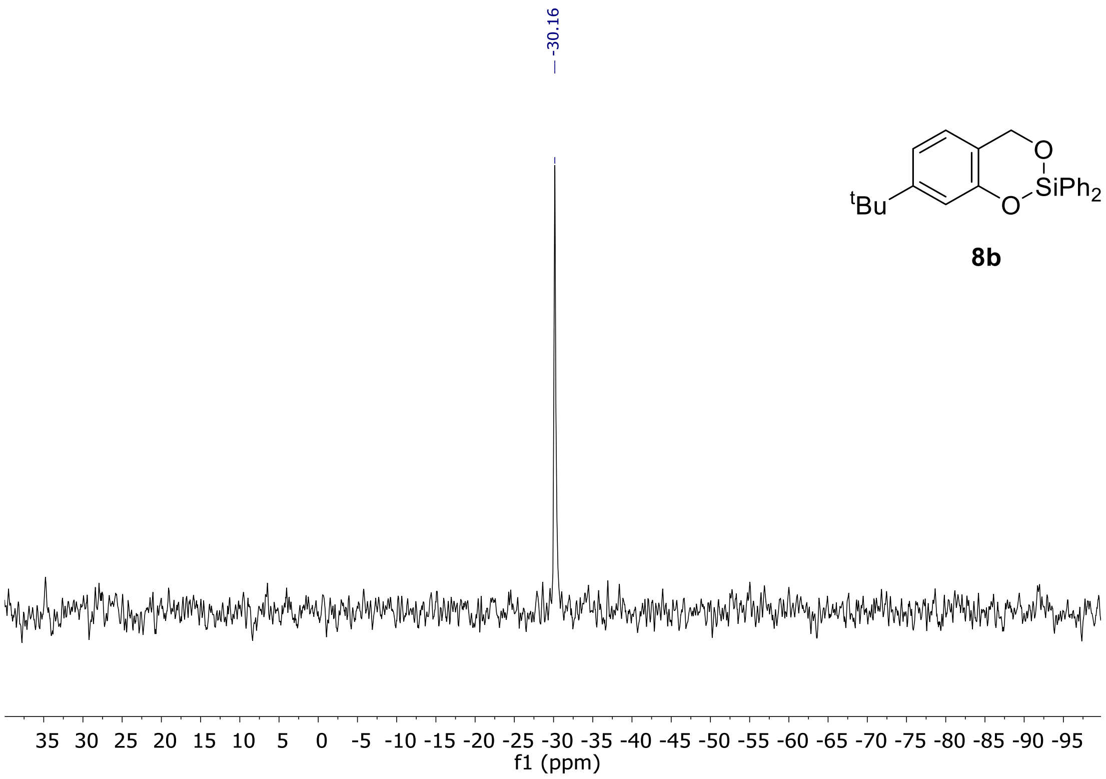

Figure S68. $^{29}Si\{^{1}H\}$ NMR spectrum (59.63 MHz, $CDCl_3$, 298 K) of **8b**.

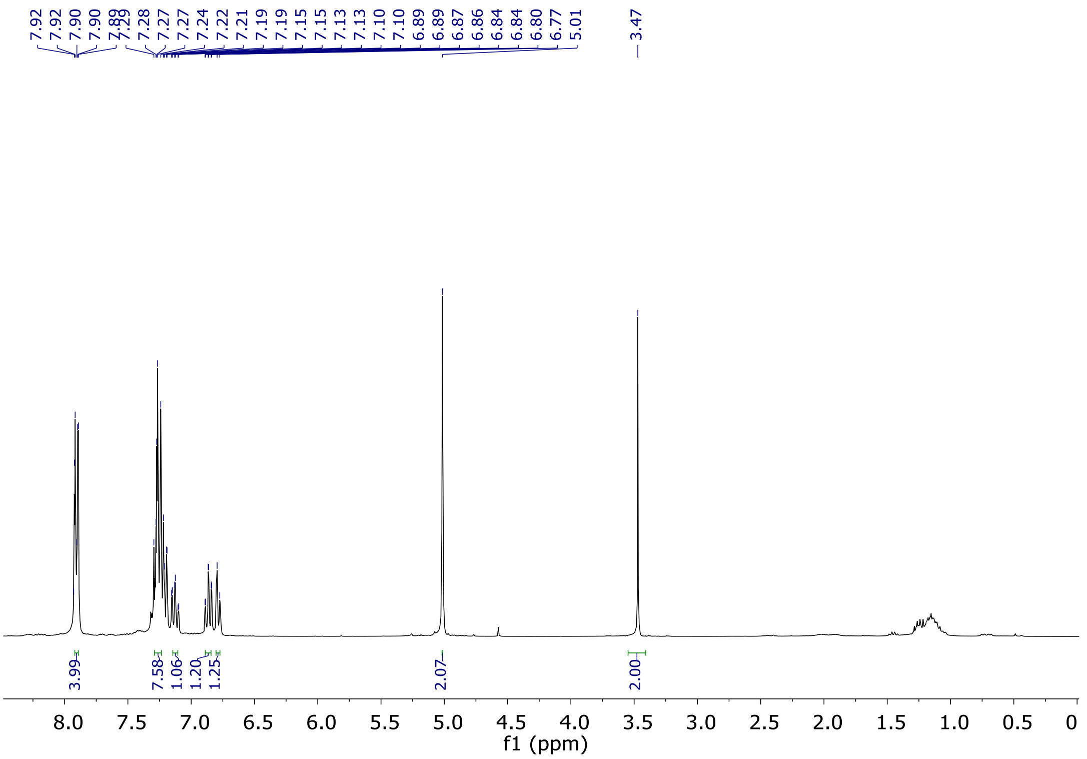

Figure S69. ^{1}H NMR spectrum (300 MHz, benzene-d_6, 298 K) of the crude reaction mixture of the tandem hydrosilylation/dehydrogenative silylation of salicylaldehyde with H_2SiPh_2 to give **8c**. Dioxane (δ 3.47 ppm; internal standard).

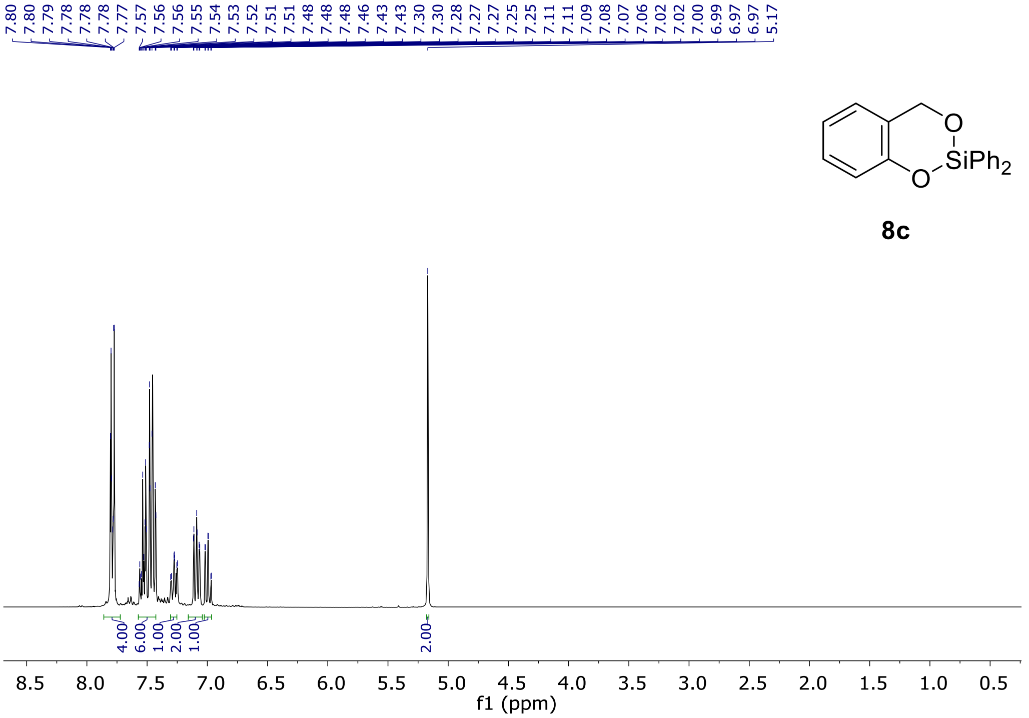

8c

Figure S70. ^{1}H NMR spectrum (300 MHz, $CDCl_3$, 298 K) of **8c**.

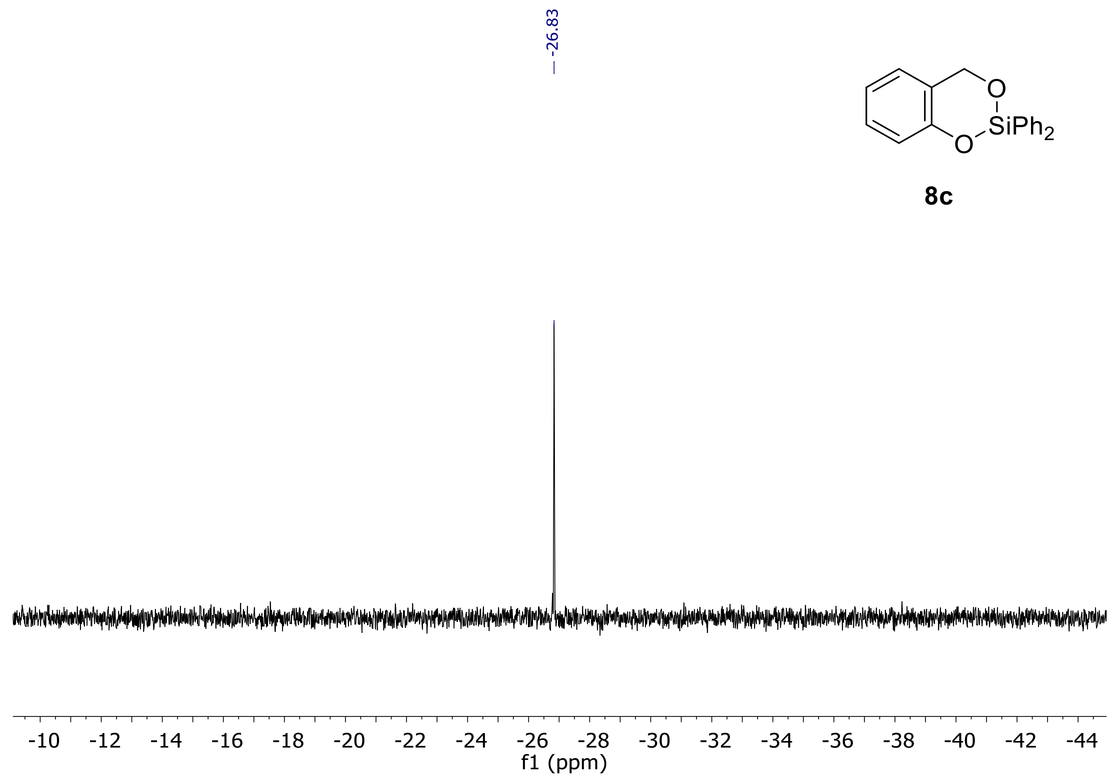

Figure S71. ^{29}Si {^1H} NMR spectrum (59.63 MHz, $CDCl_3$, 298 K) of **8c**.

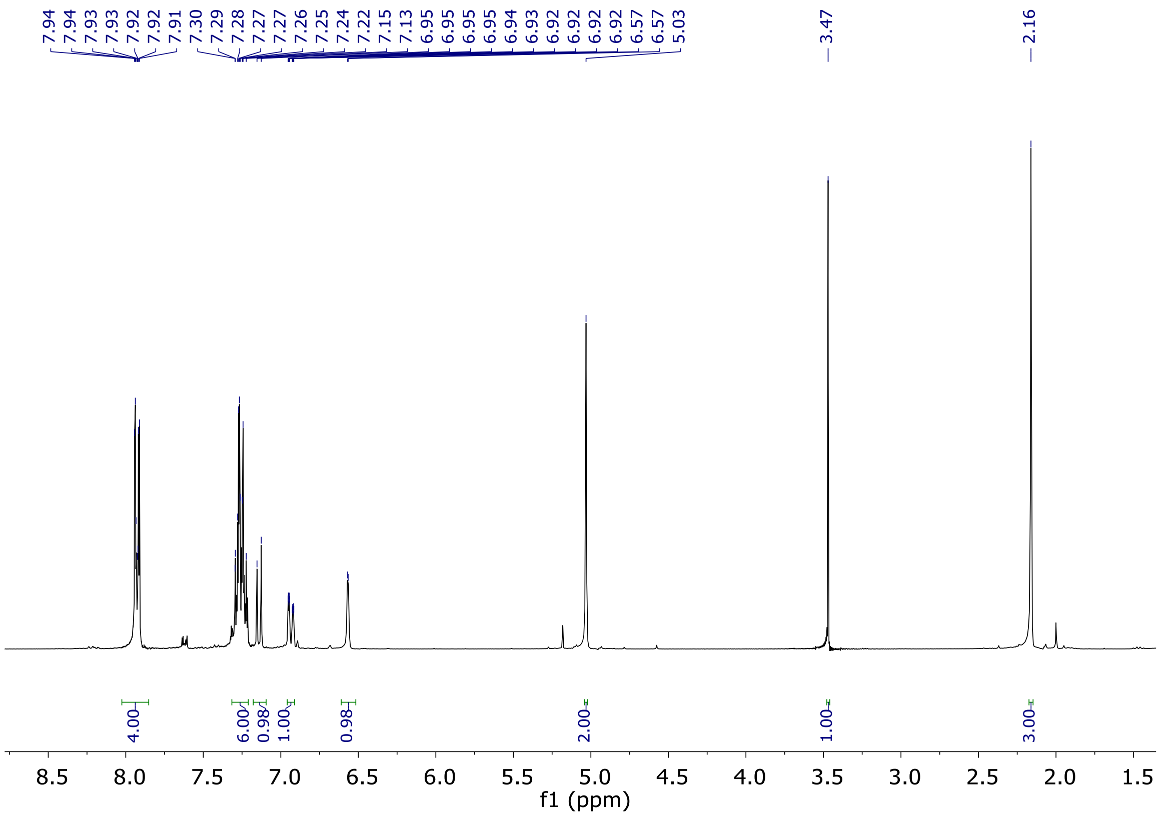

Figure S72. ^1H NMR spectrum (300 MHz, benzene-d_6, 298 K) of the crude reaction mixture of the tandem hydrosilylation/dehydrogenative silylation of 5-methyl-2-hydroxybenzaldehyde with H_2SiPh_2 to afford **8d**. Dioxane (δ 3.47 ppm; internal standard).

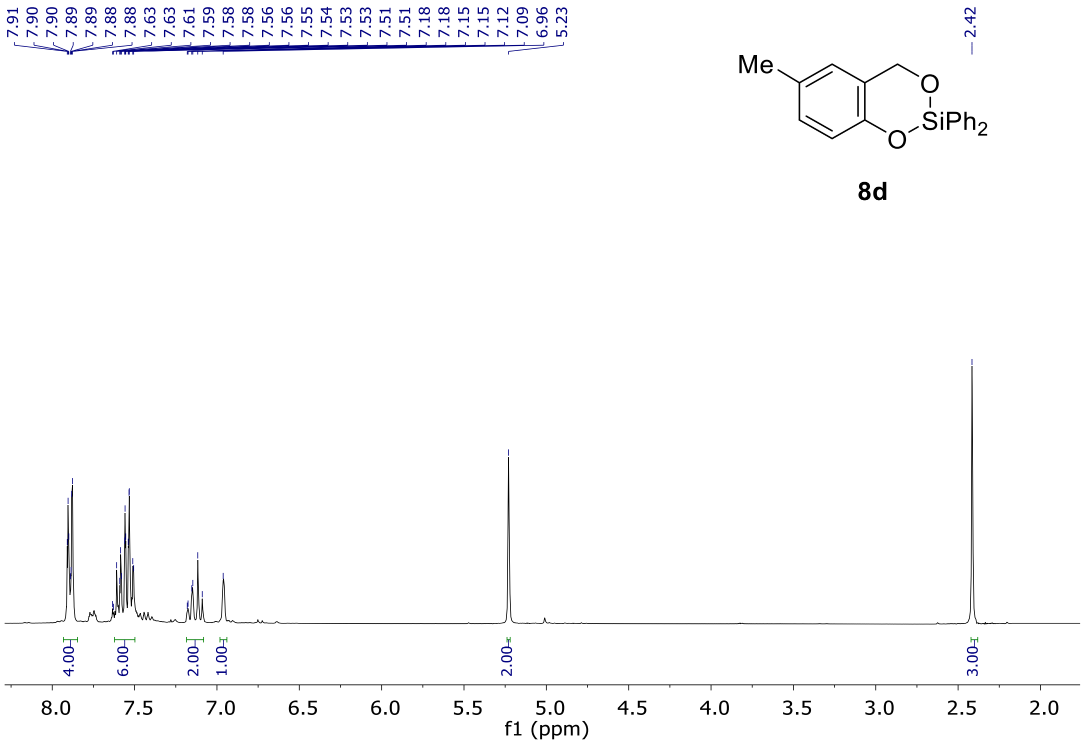

Figure S73. ^{1}H NMR spectrum (300 MHz, $CDCl_3$, 298 K) of **8d**.

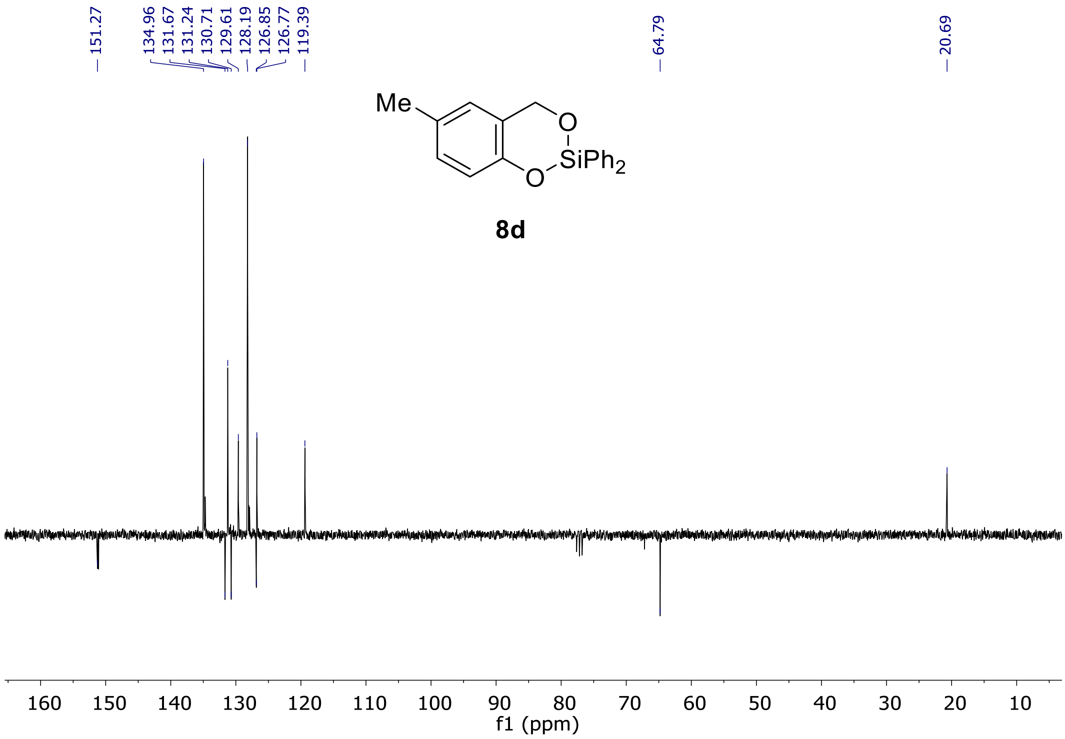

Figure S74. ^{13}C{^{1}H}-apt NMR spectrum (75.429 MHz, C_6D_6, 298 K) of **8d**.

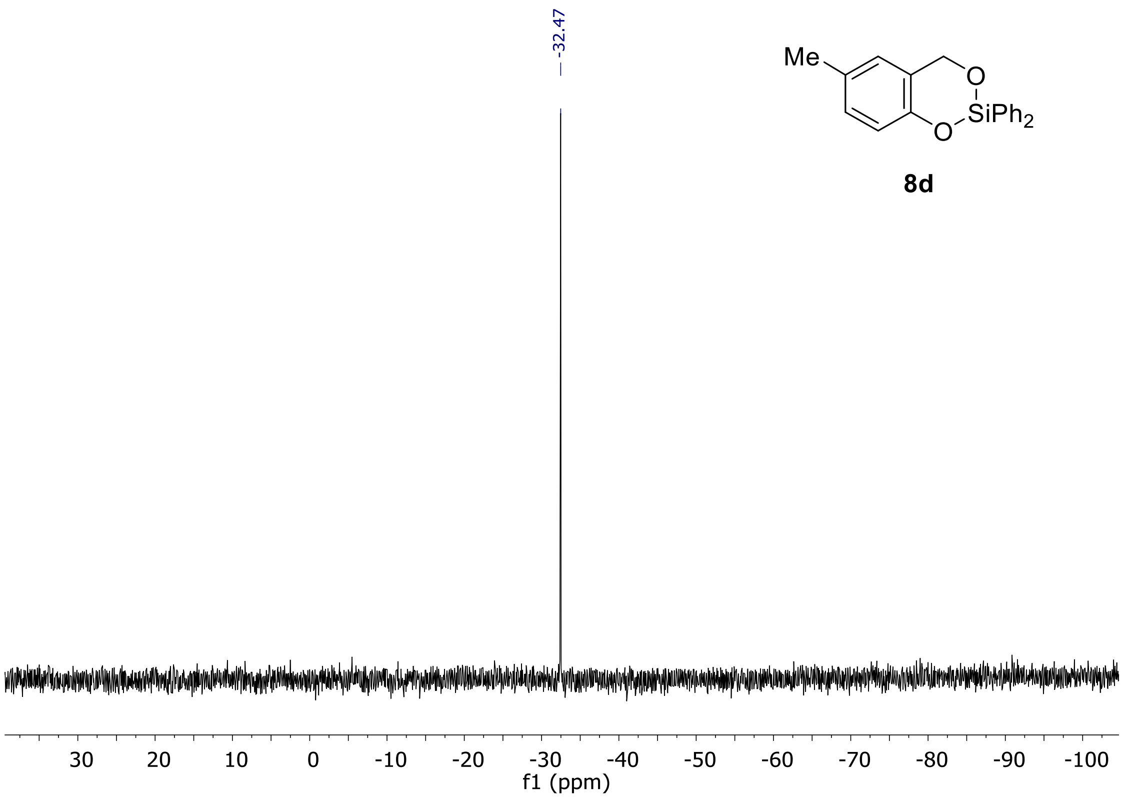

Figure S75. $^{29}Si\{^{1}H\}$ NMR spectrum (59.63 MHz, $CDCl_3$, 298 K) of **8d**.

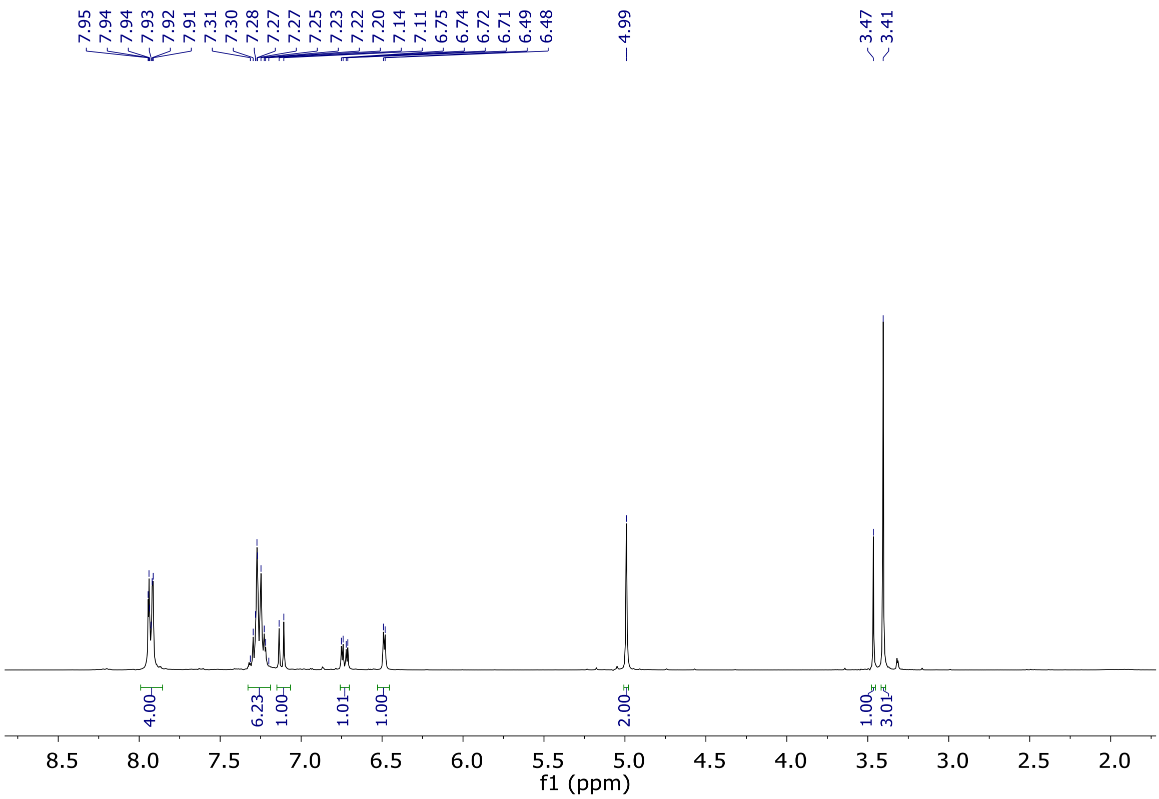

Figure S76. ^{1}H NMR spectrum (300 MHz, benzene-d_6, 298 K) of the crude reaction mixture of the tandem hydrosilylation/dehydrogenative silylation of 5-methoxy-2-hydroxybenzaldehyde with H_2SiPh_2 to afford **8e**. Dioxane (δ 3.47 ppm; internal standard).

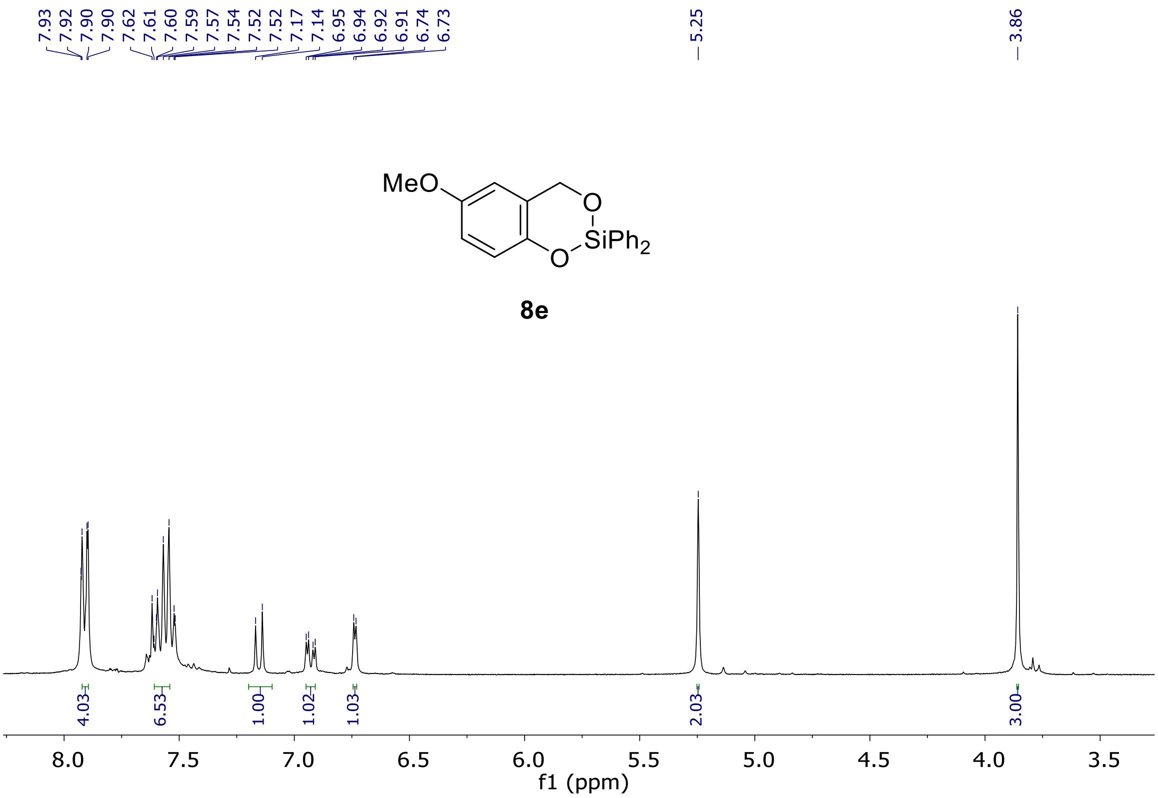

Figure S77. ^{1}H NMR spectrum (300 MHz, $CDCl_3$, 298 K) of **8e**.

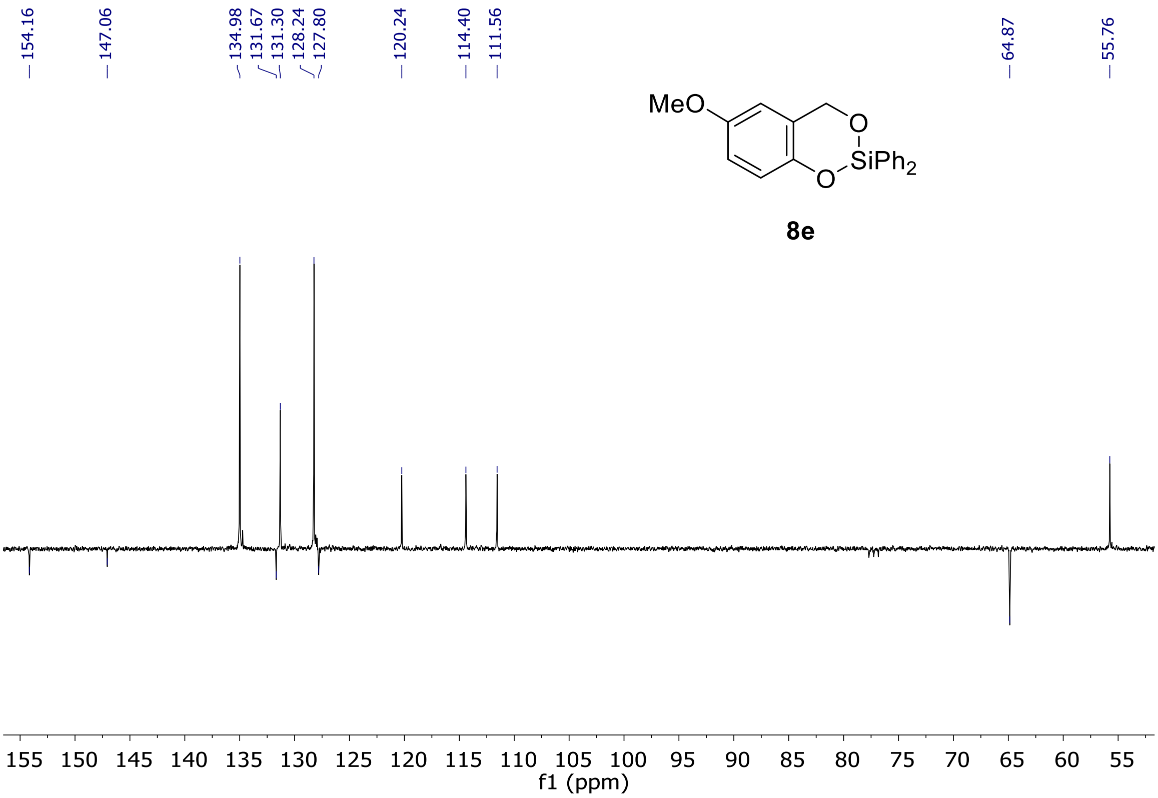

Figure S78. $^{13}C\{^{1}H\}$-apt NMR spectrum (75.429 MHz, C_6D_6, 298 K) of **8e**.

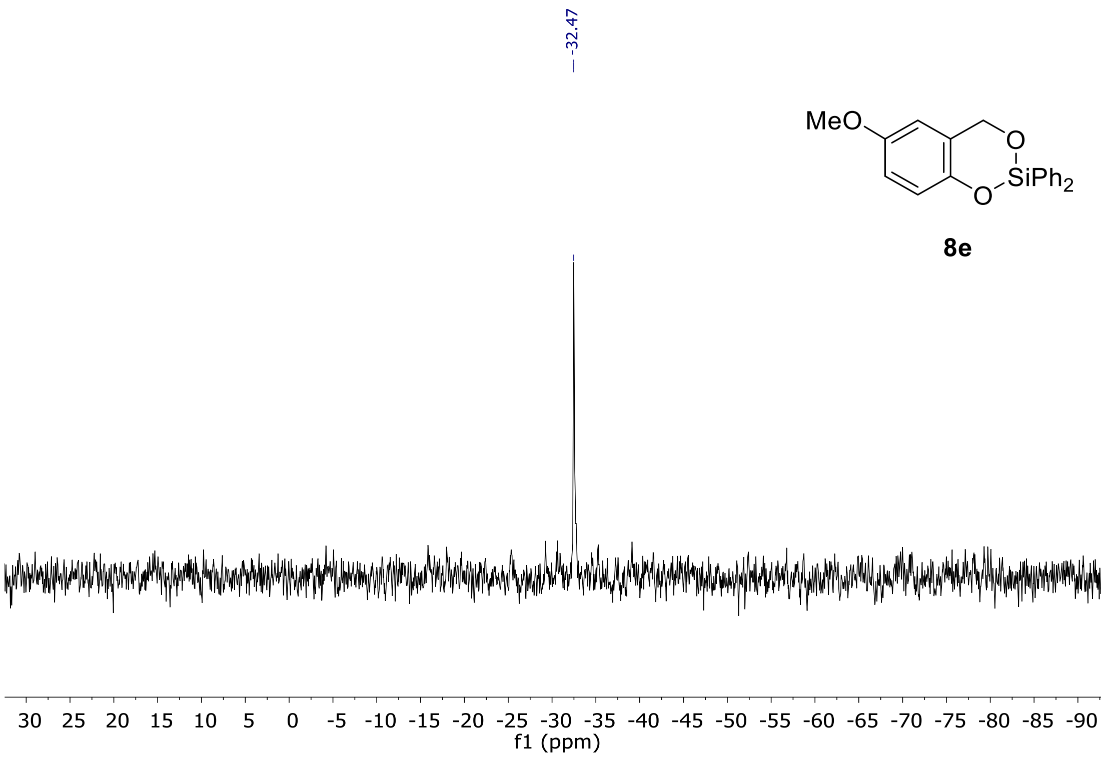

Figure S79. $^{29}Si\{^{1}H\}$ NMR spectrum (59.63 MHz, $CDCl_3$, 298 K) of **8e**.

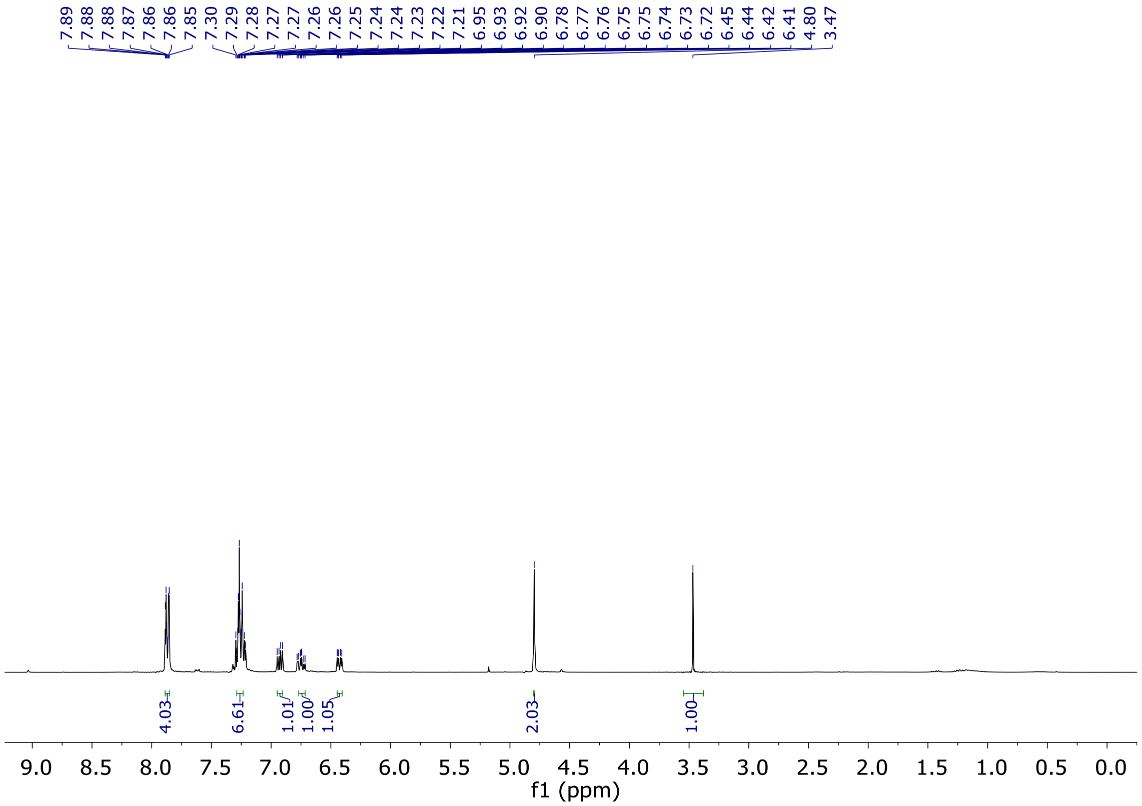

Figure S80. ^{1}H NMR spectrum (300 MHz, benzene-d_6, 298 K) of the crude reaction mixture of the tandem hydrosilylation/dehydrogenative silylation of 5-fluoro-2-hydroxybenzaldehyde with H_2SiPh_2 to afford **8f**. Dioxane (δ 3.47 ppm; internal standard).

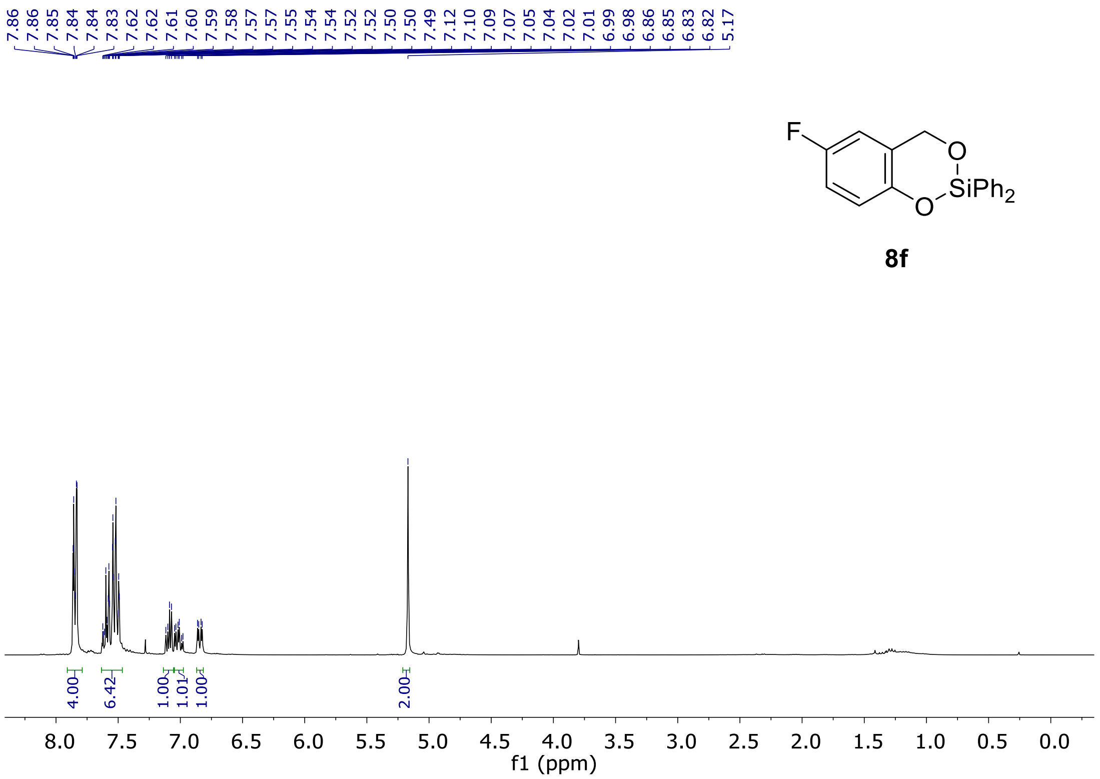

Figure S81. ^{1}H NMR spectrum (300 MHz, $CDCl_3$, 298 K) of **8f**.

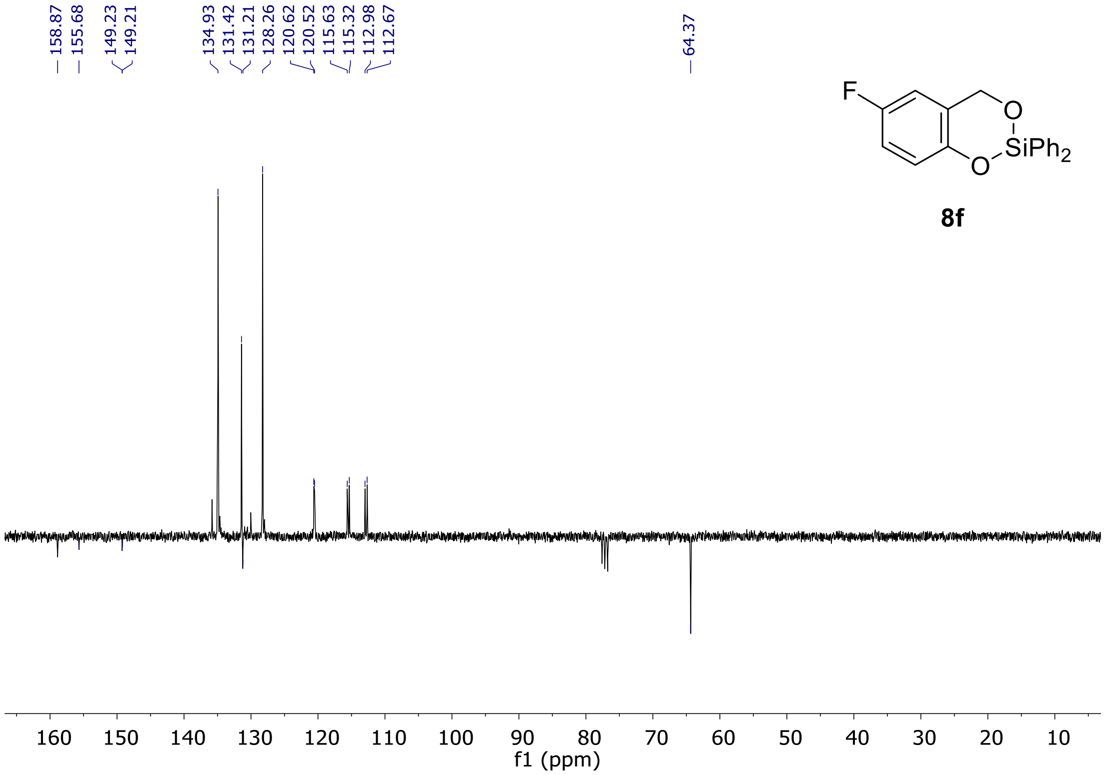

Figure S82. $^{13}C\{^{1}H\}$-apt NMR spectrum (75.429 MHz, C_6D_6, 298 K) of **8f**.

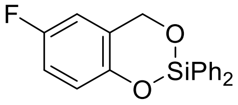

8f

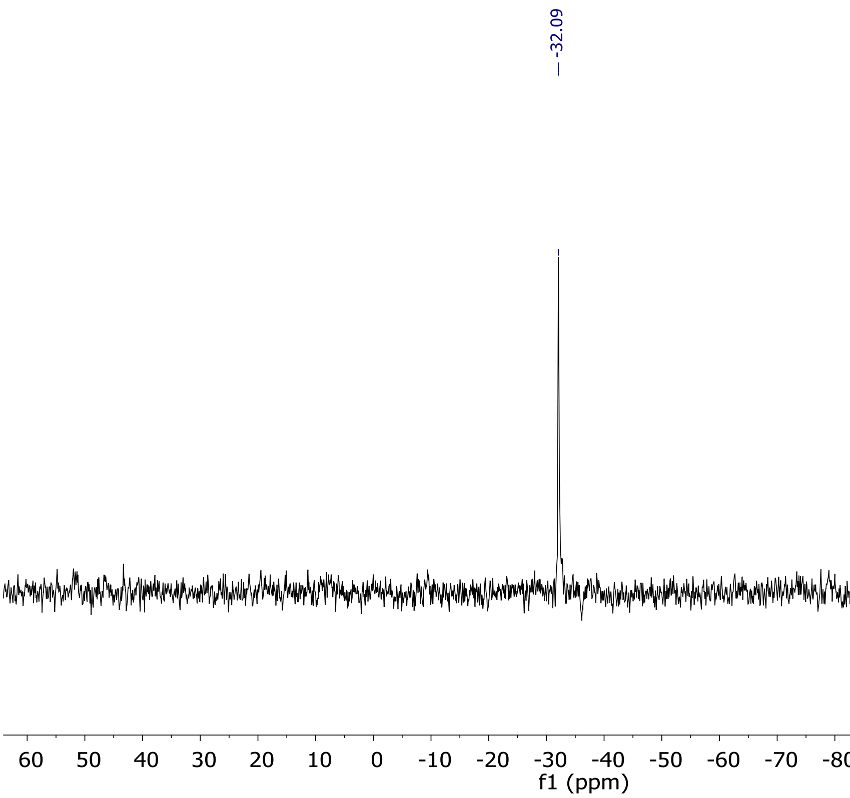

Figure S83. $^{29}Si\{^{1}H\}$ NMR spectrum (59.63 MHz, $CDCl_3$, 298 K) of **8f**.

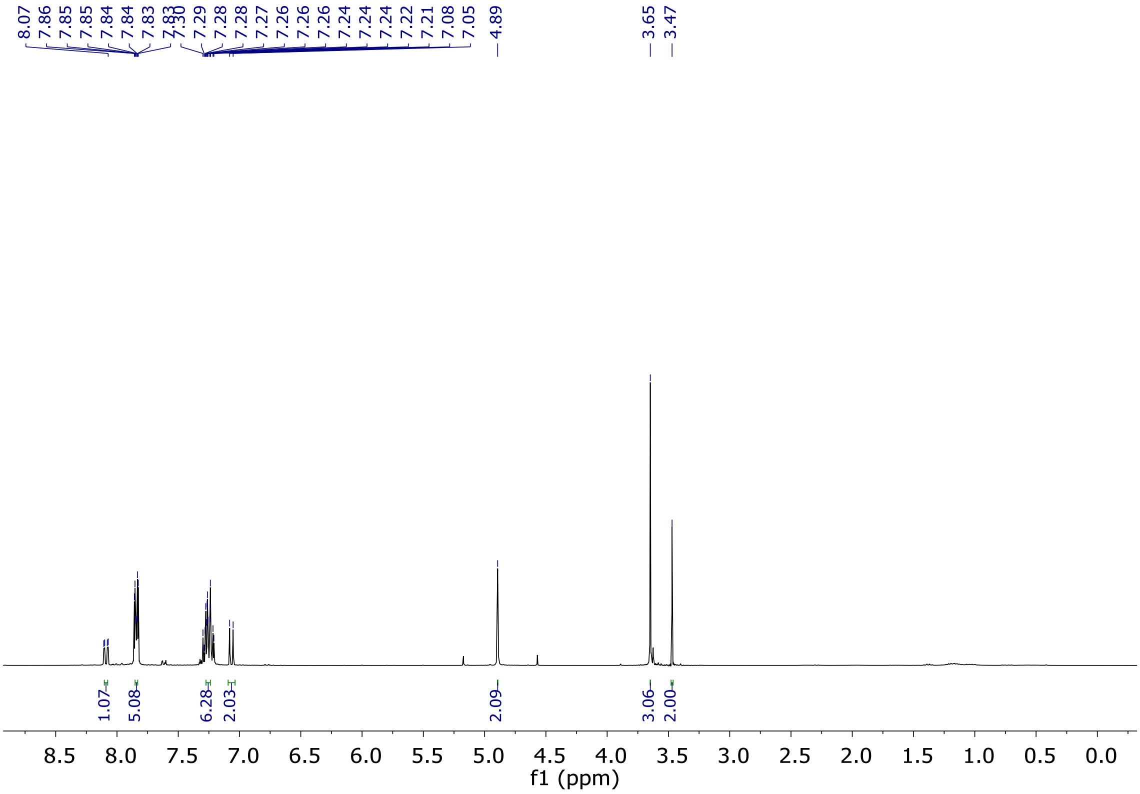

Figure S84. ^{1}H NMR spectrum (300 MHz, benzene-d_6, 298 K) of the crude reaction mixture of the tandem hydrosilylation/dehydrogenative silylation of methyl 3-formyl-4-hydroxybenzoate with H_2SiPh_2. Dioxane (δ 3.47 ppm; internal standard).

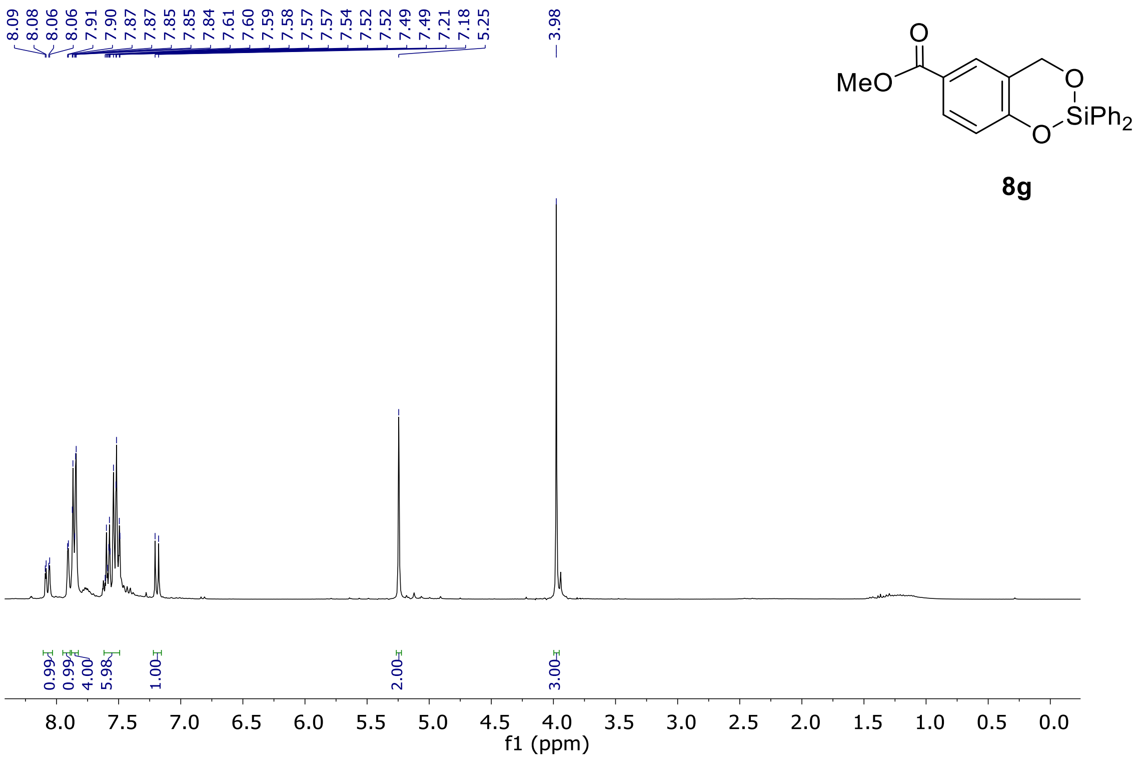

Figure S85. ^{1}H NMR spectrum (300 MHz, $CDCl_3$, 298 K) of **8g**

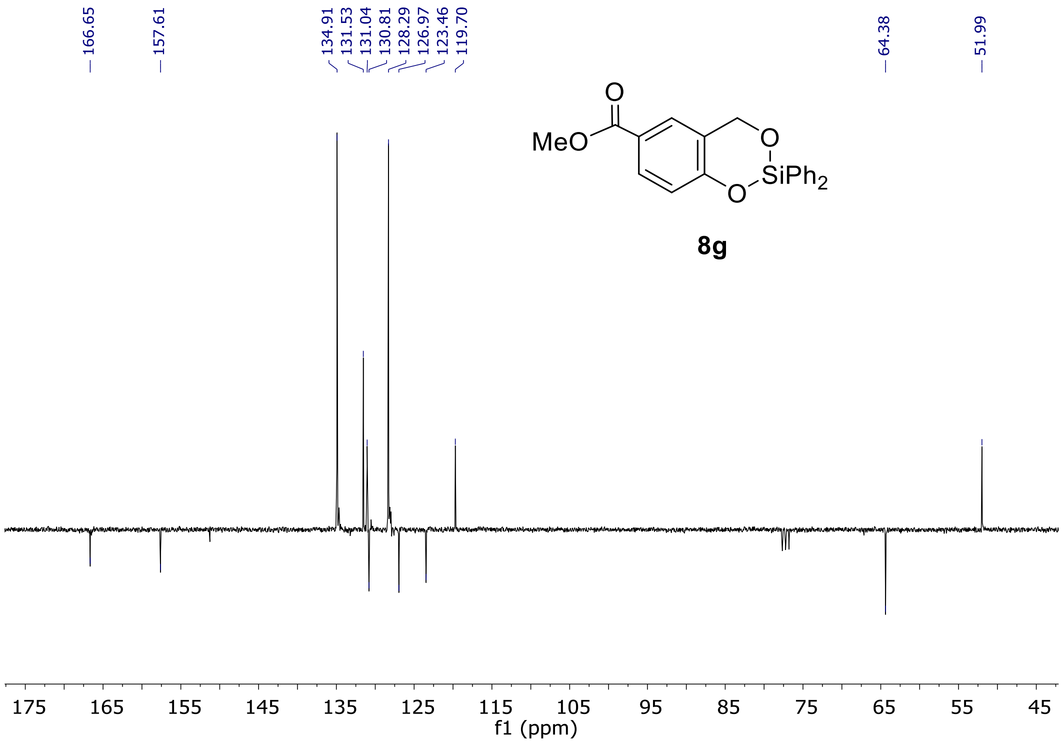

Figure S86. $^{13}C\{^{1}H\}$-apt NMR spectrum (75.429 MHz, C_6D_6, 298 K) of **8g**.

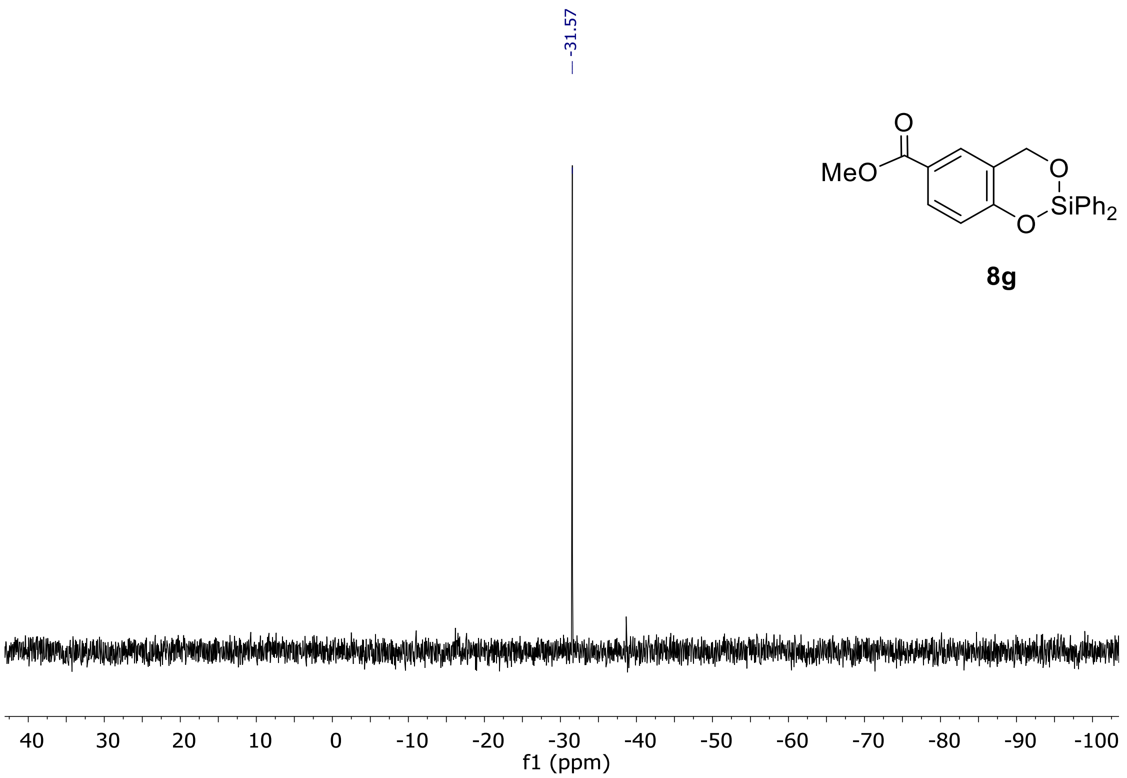

Figure S87. $^{29}Si\{^{1}H\}$ NMR spectrum (59.63 MHz, $CDCl_3$, 298 K) of **8g**.

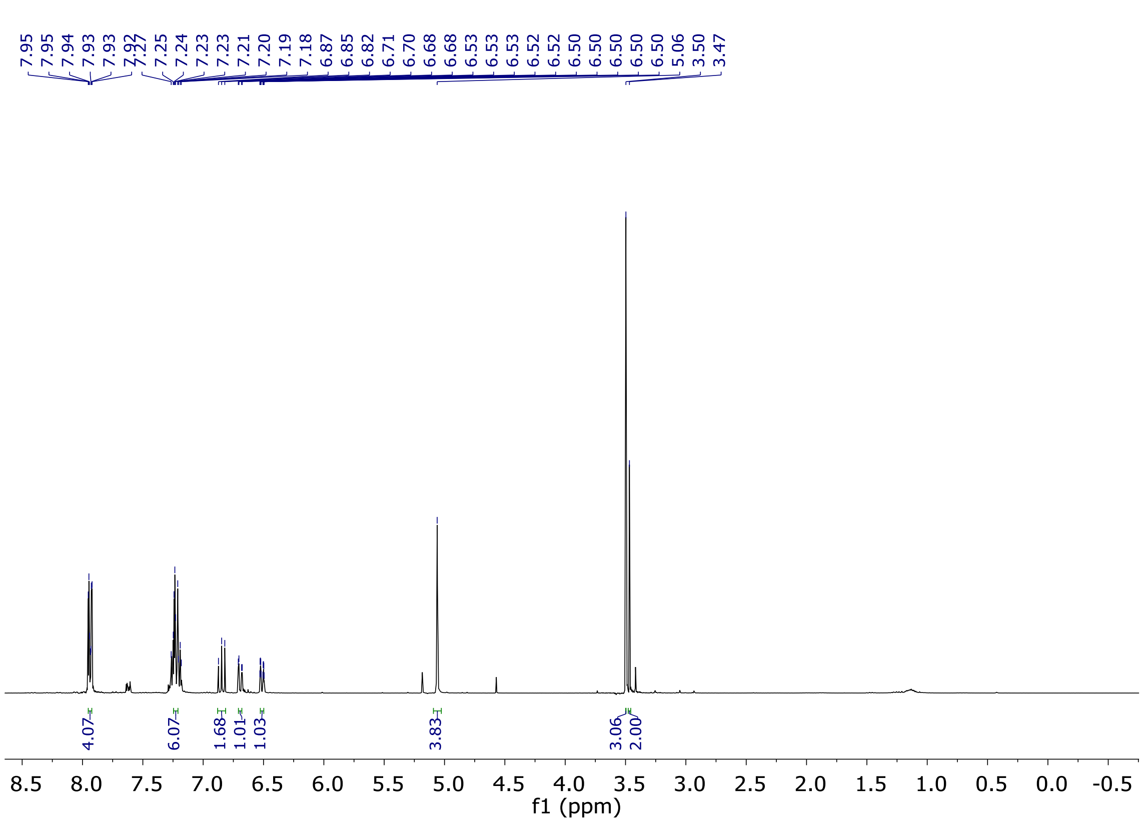

Figure S88. ^{1}H NMR spectrum (300 MHz, benzene-d_6, 298 K) of the crude reaction mixture of the tandem hydrosilylation/dehydrogenative silylation of 2-hydroxy-3-methoxybenzaldehyde with H_2SiPh_2 to afford **8h**. Dioxane (δ 3.47 ppm; internal standard).

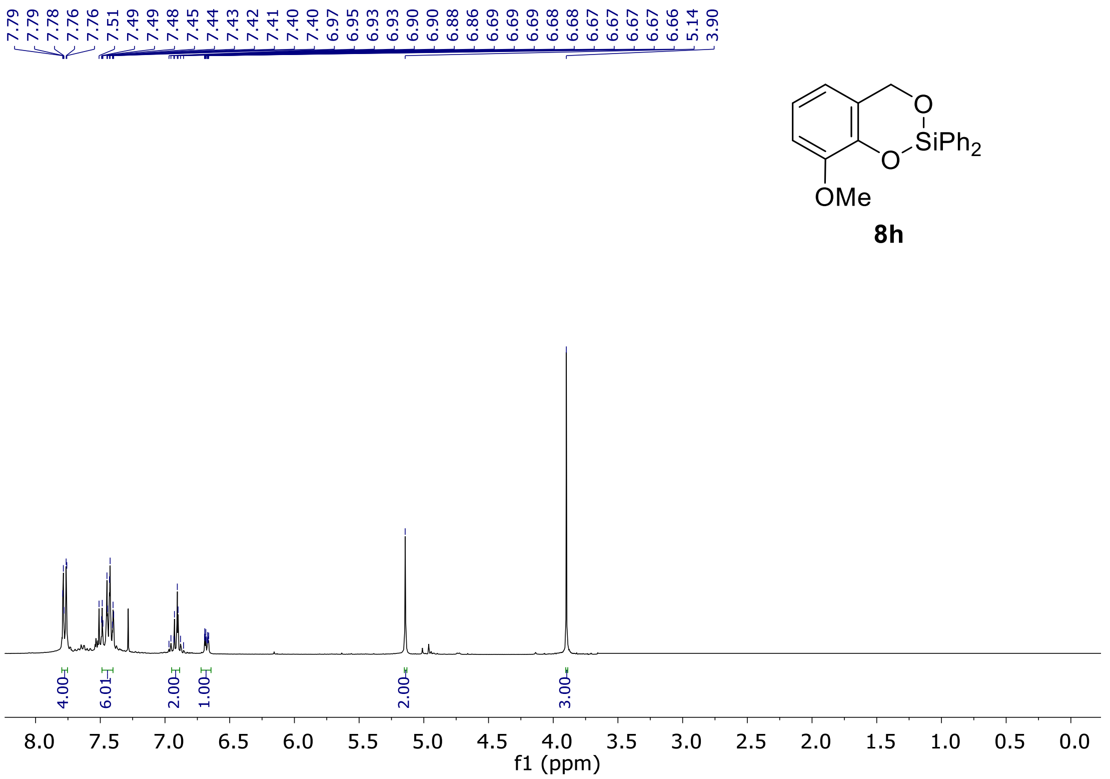

Figure S89. ^{1}H NMR spectrum (300 MHz, $CDCl_3$, 298 K) of **8h**.

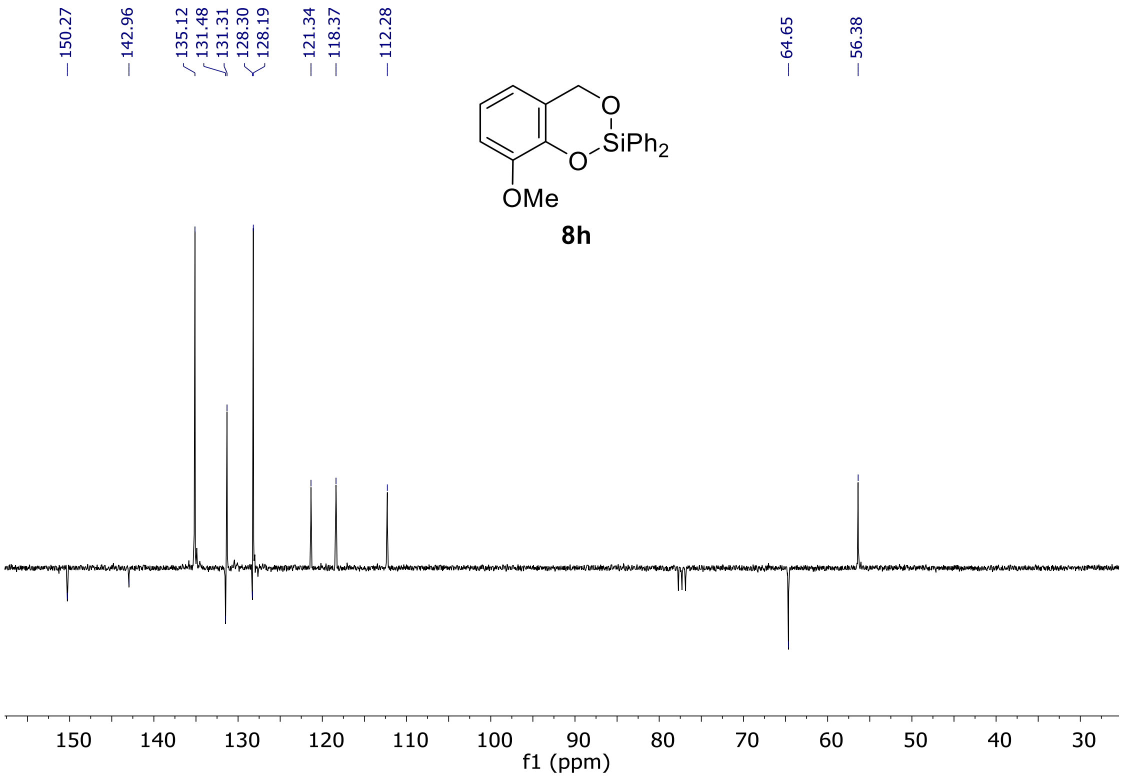

Figure S90. $^{13}C\{^{1}H\}$-apt NMR spectrum (75.429 MHz, C_6D_6, 298 K) of **8h**.

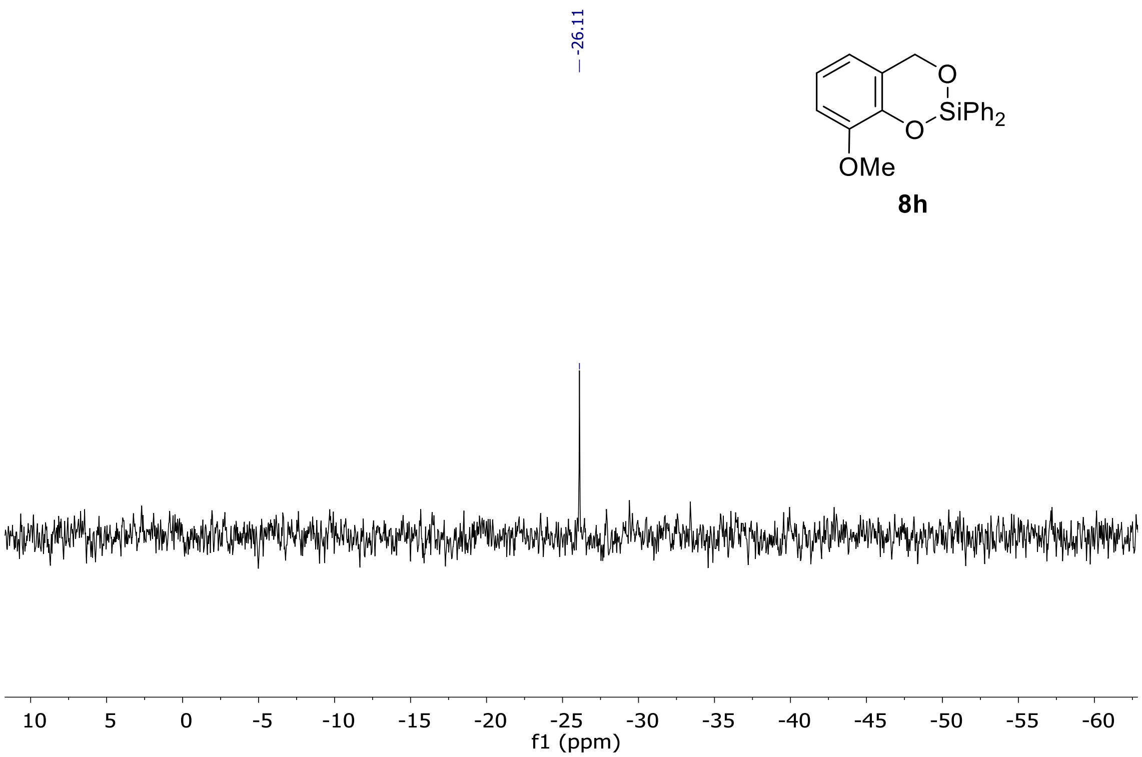

Figure S91. ^{29}Si{^{1}H} NMR spectrum (59.63 MHz, $CDCl_3$, 298 K) of **8h**.

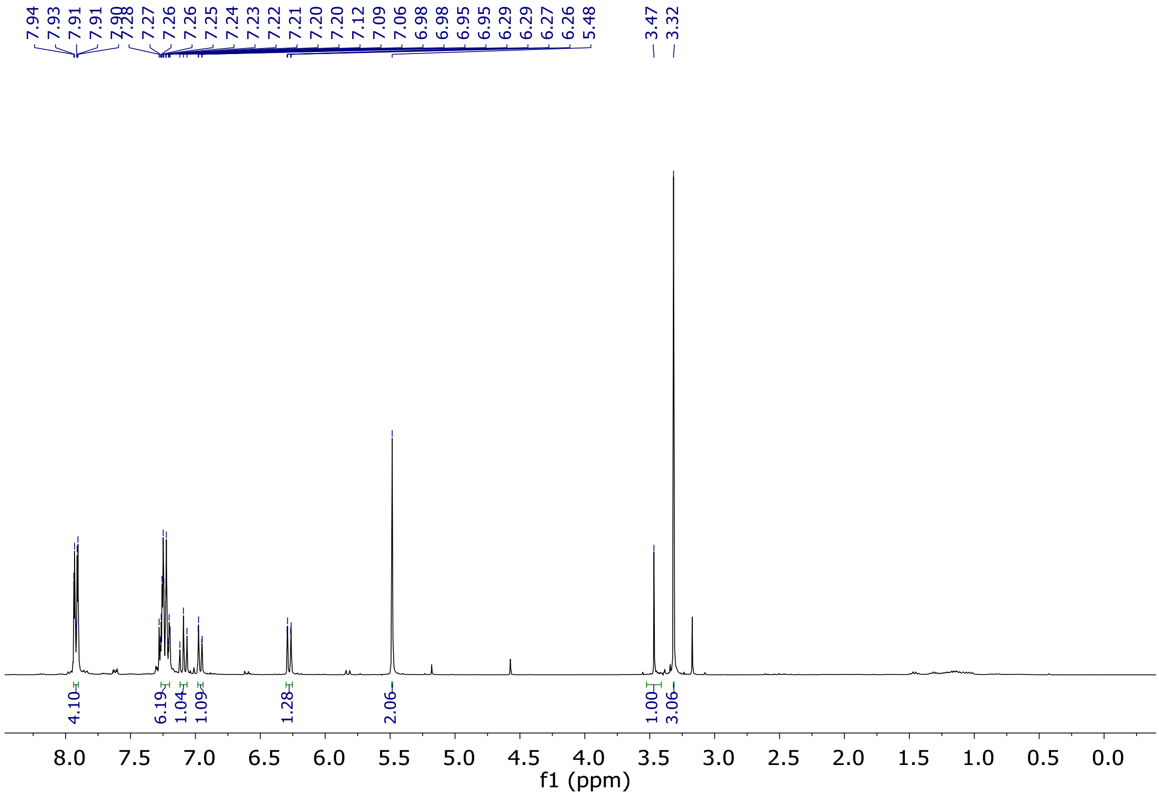

Figure S92. ^{1}H NMR spectrum (300 MHz, benzene-d_6, 298 K) of the crude reaction mixture of the tandem hydrosilylation/dehydrogenative silylation of 2-hydroxy-6-methoxybenzaldehyde with H_2SiPh_2 to afford **8i**. Dioxane (δ 3.47 ppm; internal standard).

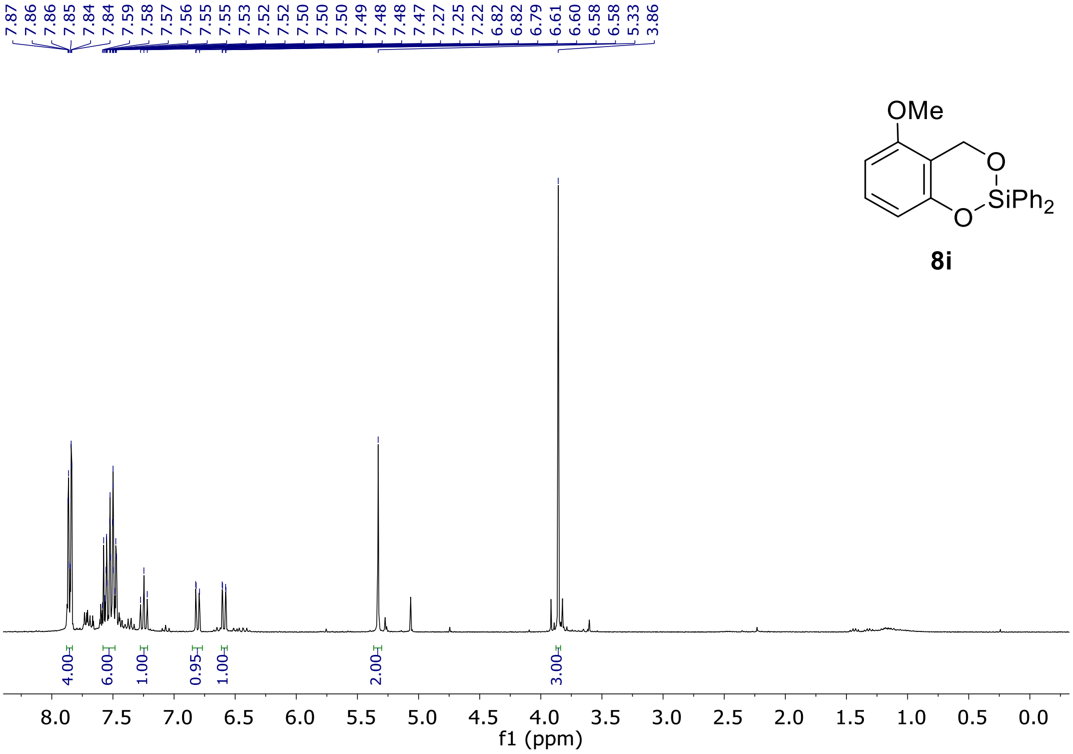

Figure S93. ^{1}H NMR spectrum (300 MHz, $CDCl_3$, 298 K) of **8i**.

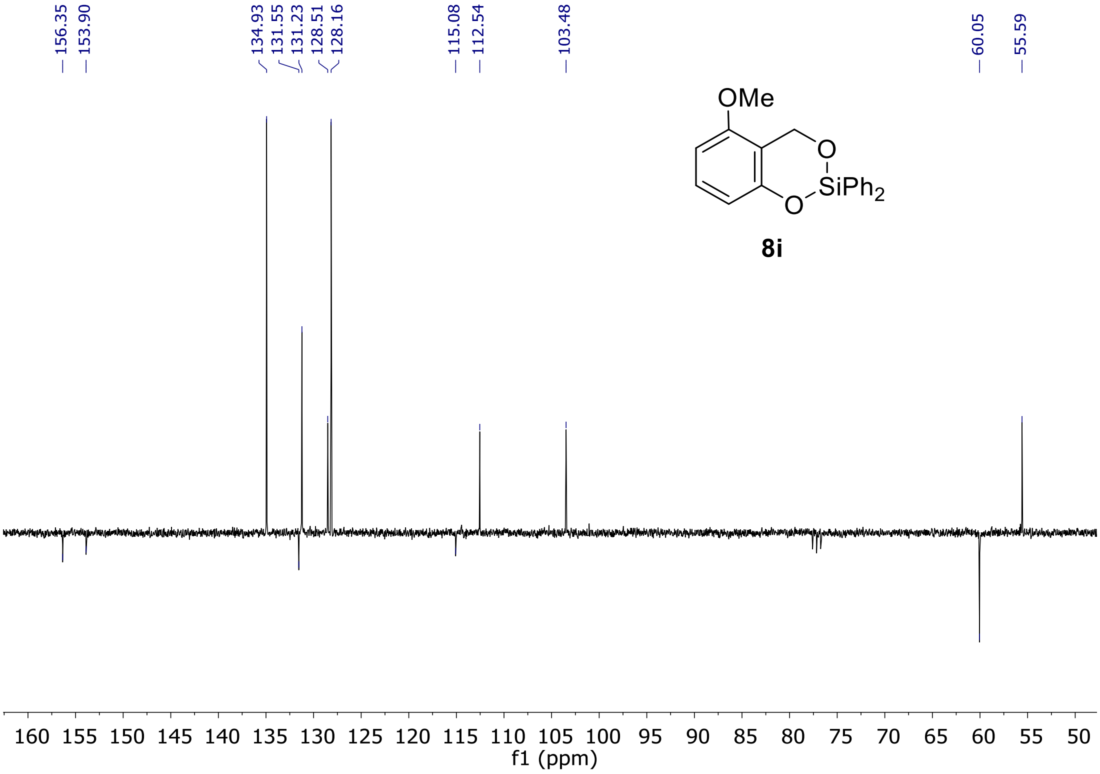

Figure S94. $^{13}C\{^{1}H\}$-apt NMR spectrum (75.429 MHz, C_6D_6, 298 K) of **8i**.

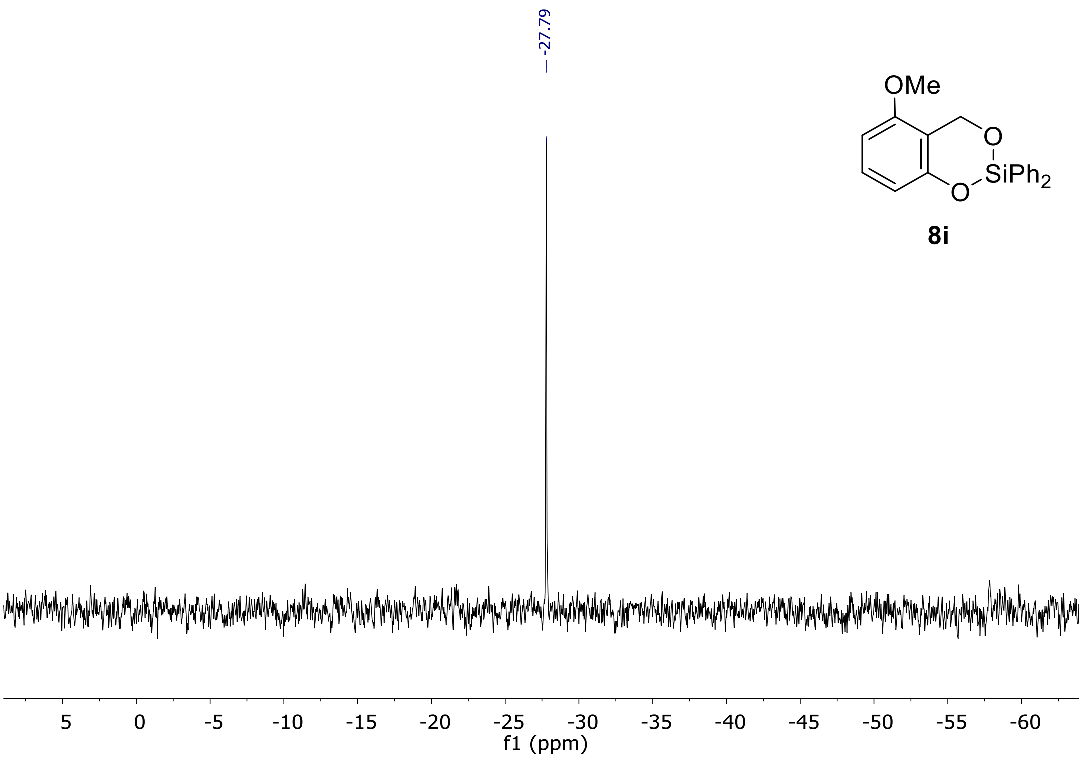

Figure S95. $^{29}Si\{^1H\}$ NMR spectrum (59.63 MHz, $CDCl_3$, 298 K) of **8i**.

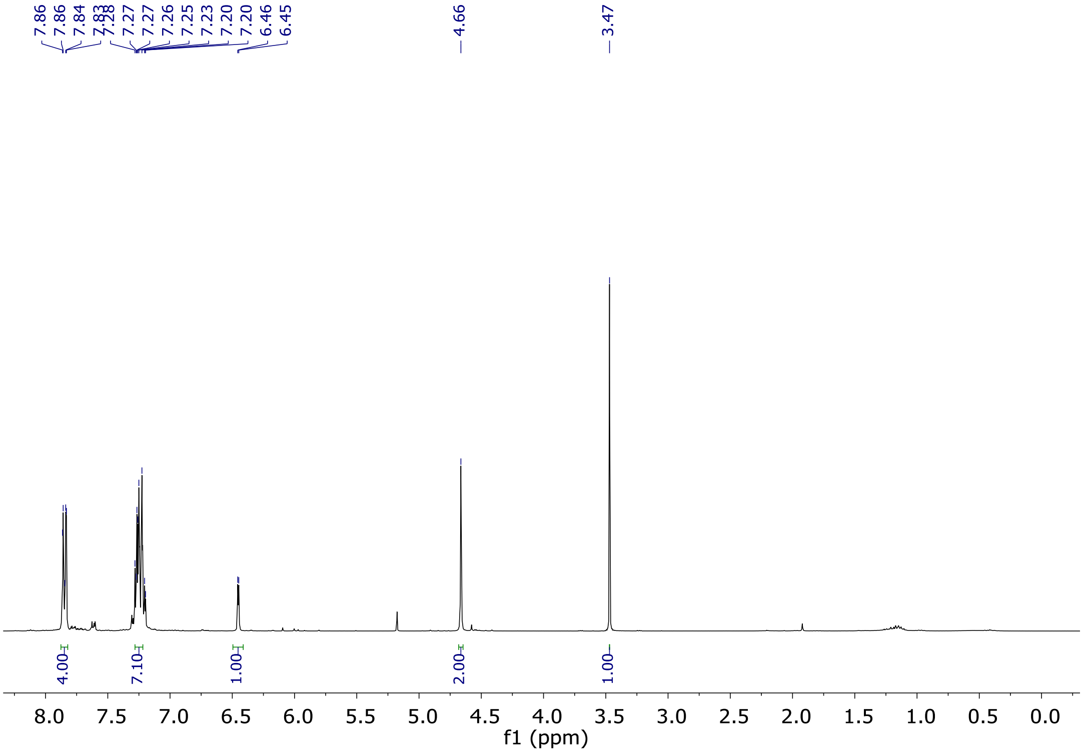

Figure S96. ^1H NMR spectrum (300 MHz, benzene-d_6, 298 K) of the crude reaction mixture of the tandem hydrosilylation/dehydrogenative silylation of 2-hydroxy-3,5-dichlorobenzaldehyde with H_2SiPh_2 to afford **8j**. Dioxane (δ 3.47 ppm; internal standard).

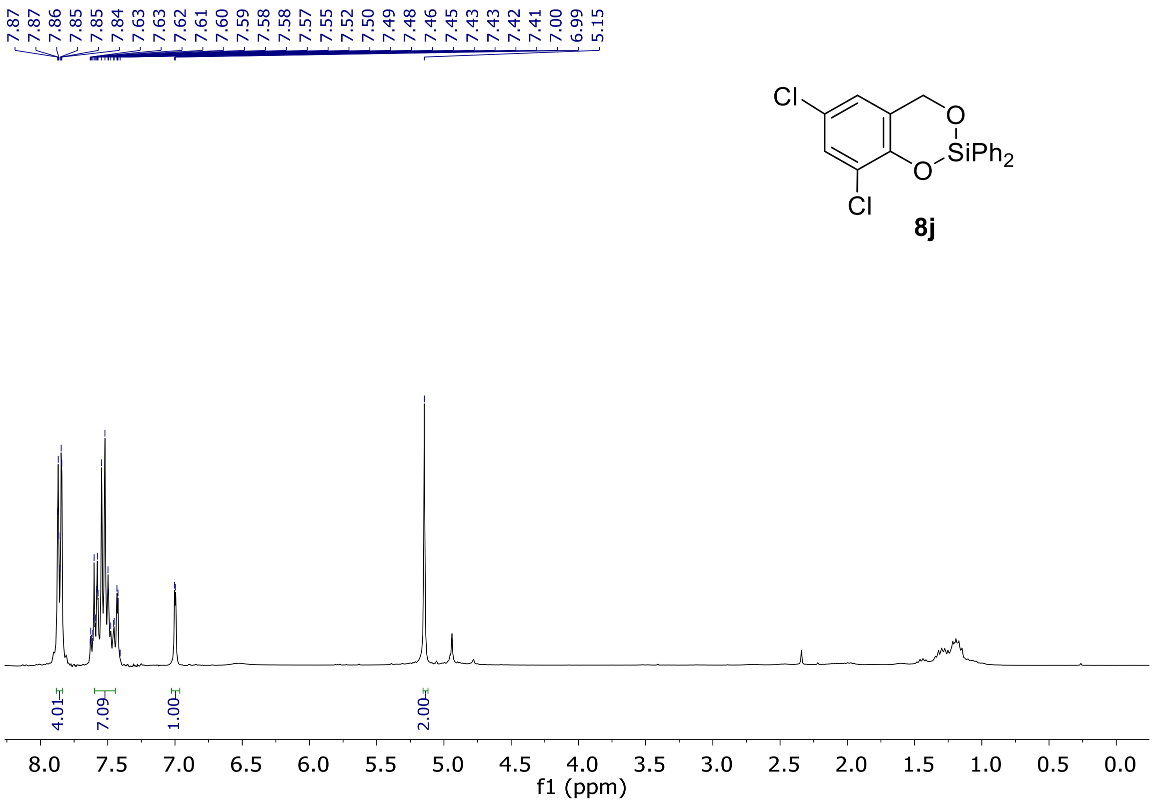

Figure S97. ^{1}H NMR spectrum (300 MHz, $CDCl_3$, 298 K) of **8j**.

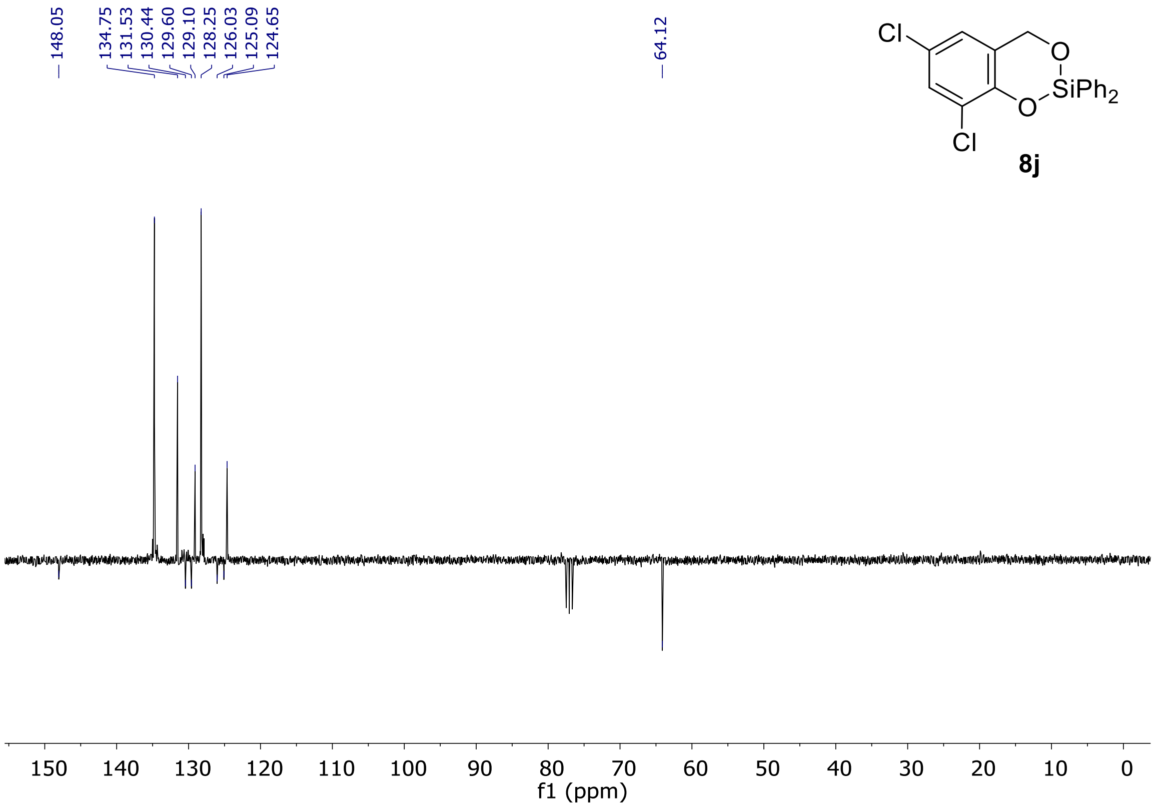

Figure S98. $^{13}C\{^{1}H\}$-apt NMR spectrum (75.429 MHz, C_6D_6, 298 K) of **8j**.

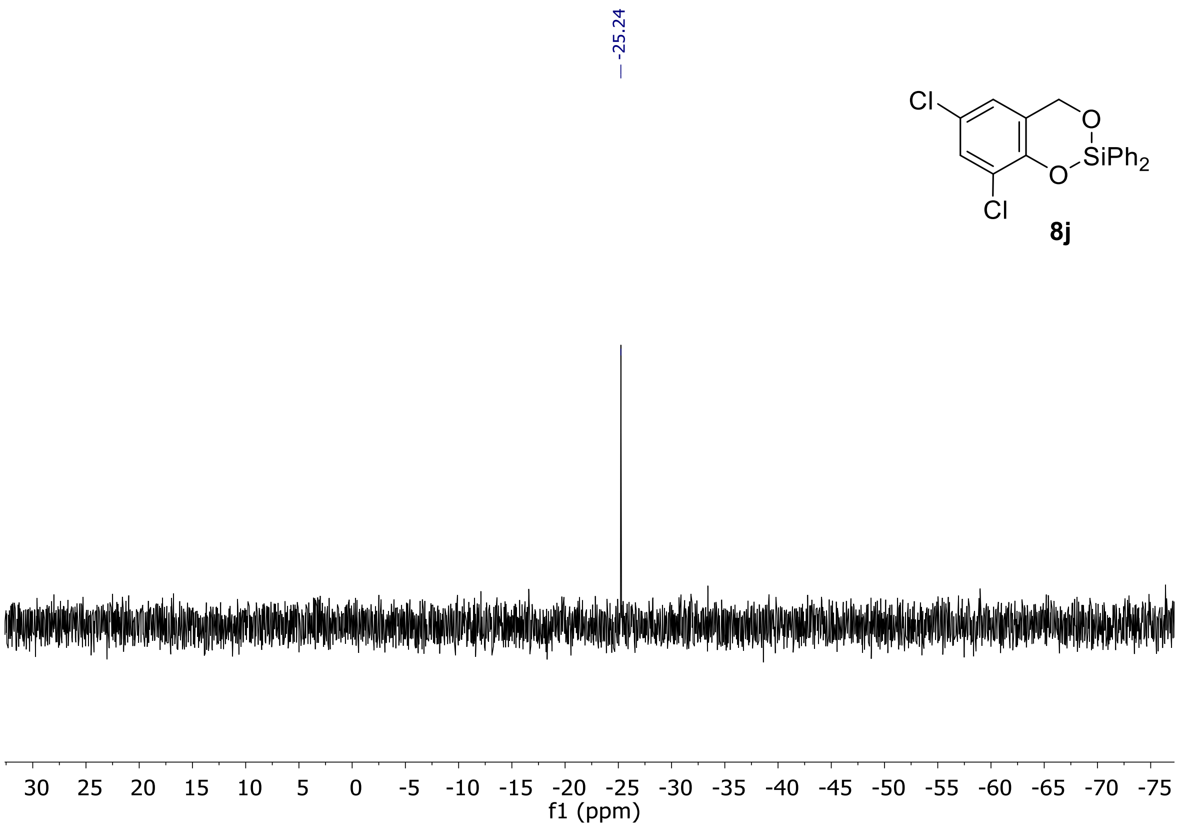

Figure S99. $^{29}Si\{^1H\}$ NMR spectrum (59.63 MHz, $CDCl_3$, 298 K) of **8j**.

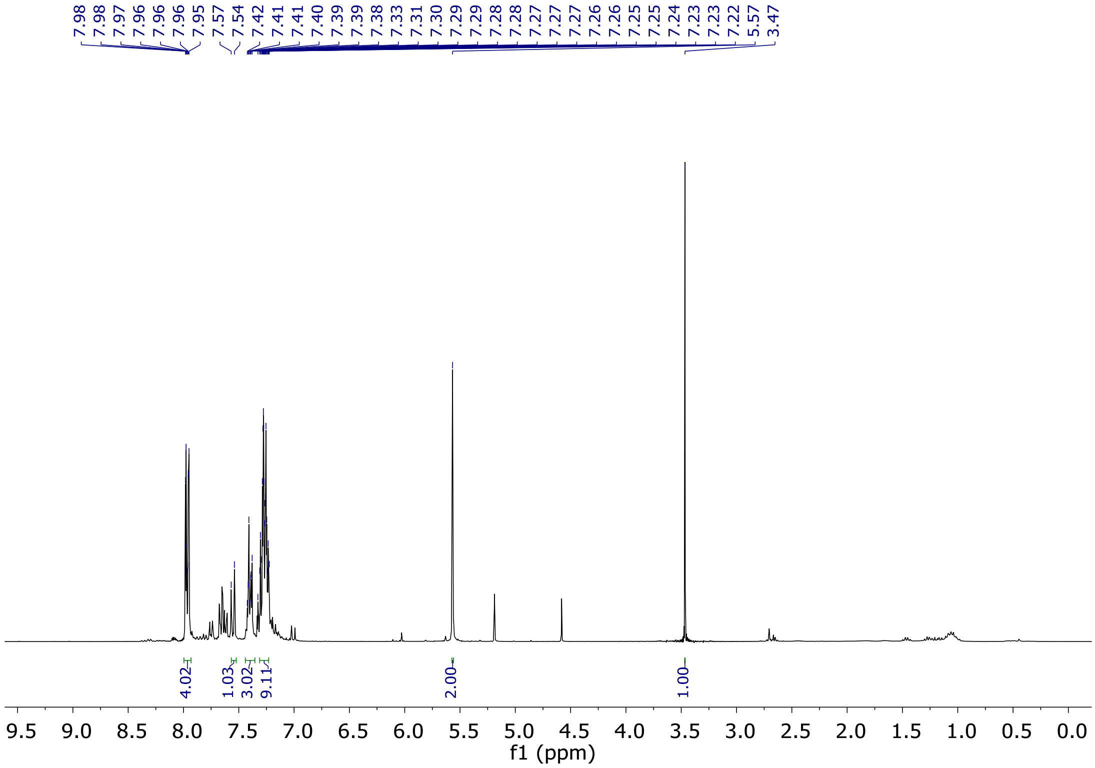

Figure S100. ^1H NMR spectrum (300 MHz, benzene-d_6, 298 K) of the crude reaction mixture of the tandem hydrosilylation/dehydrogenative silylation of 2-hydroxynaphthalene-1-carbaldehyde with H_2SiPh_2. Dioxane (δ 3.47 ppm; internal standard).

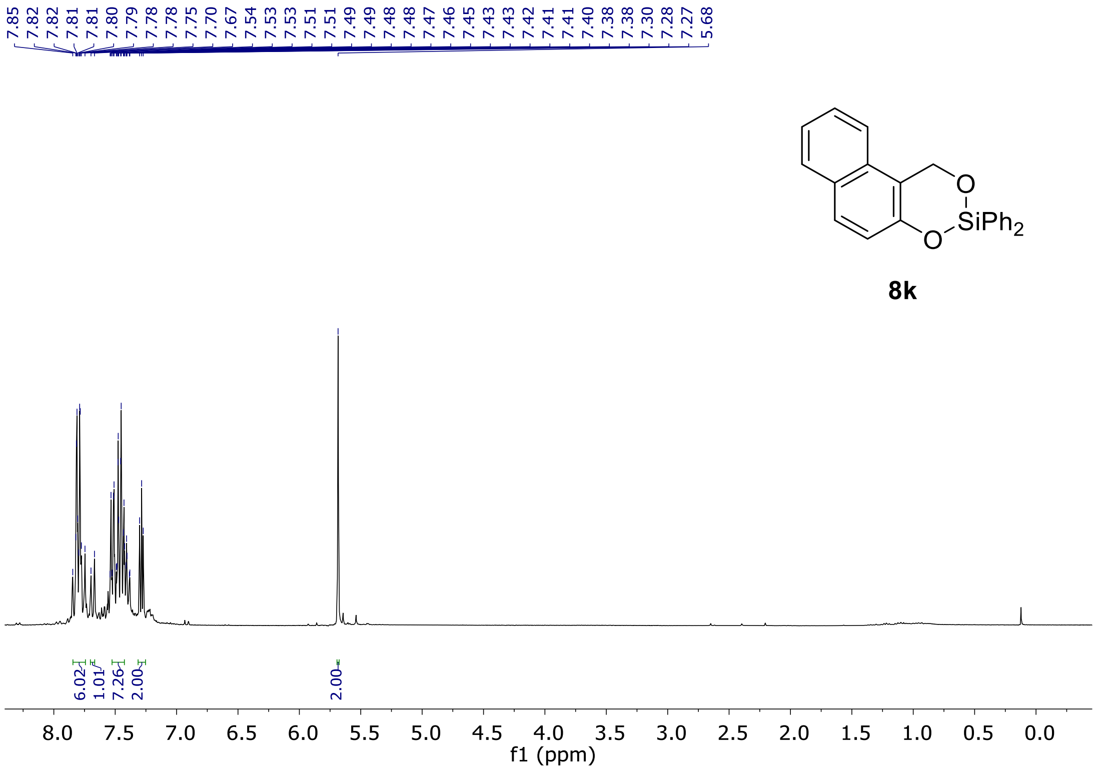

Figure S101. ^{1}H NMR spectrum (300 MHz, $CDCl_3$, 298 K) of **8k**.

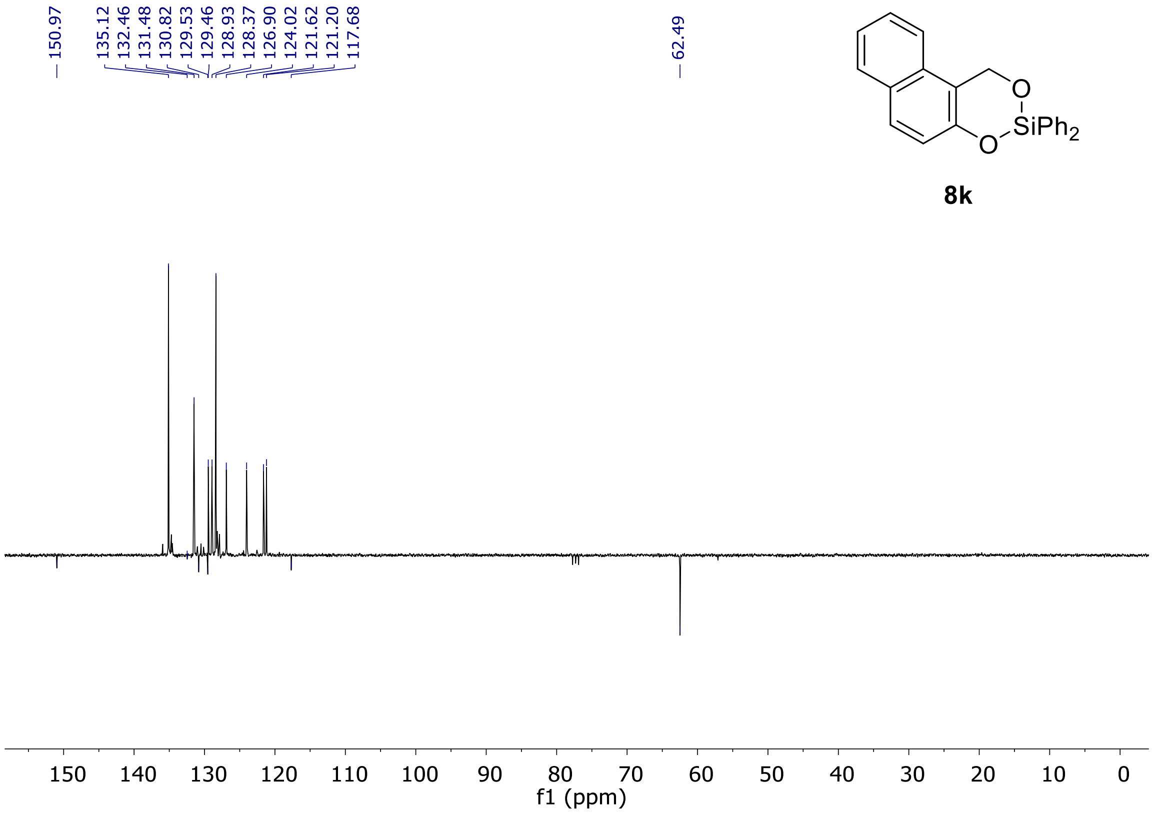

Figure S102. $^{13}C\{^{1}H\}$-apt NMR spectrum (75.429 MHz, C_6D_6, 298 K) of **8k**.

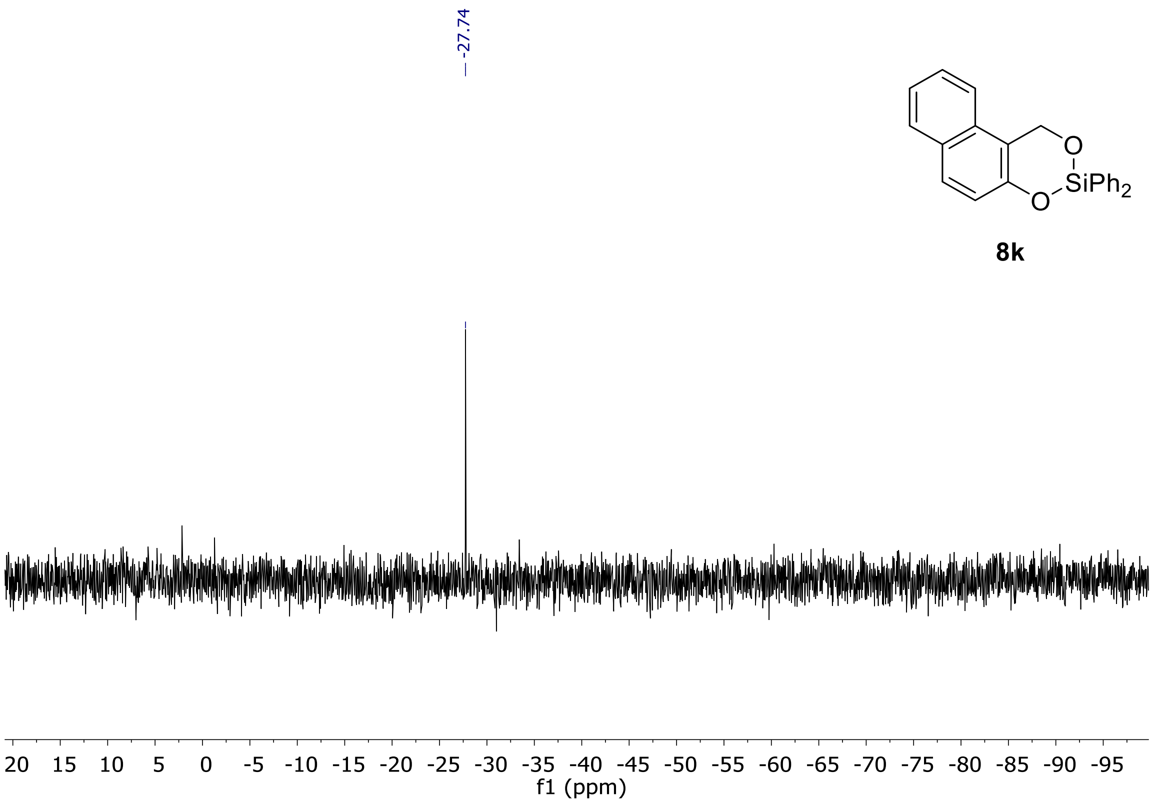

Figure S103. $^{29}Si\{^{1}H\}$ NMR spectrum (59.63 MHz, $CDCl_3$, 298 K) of **8k**.

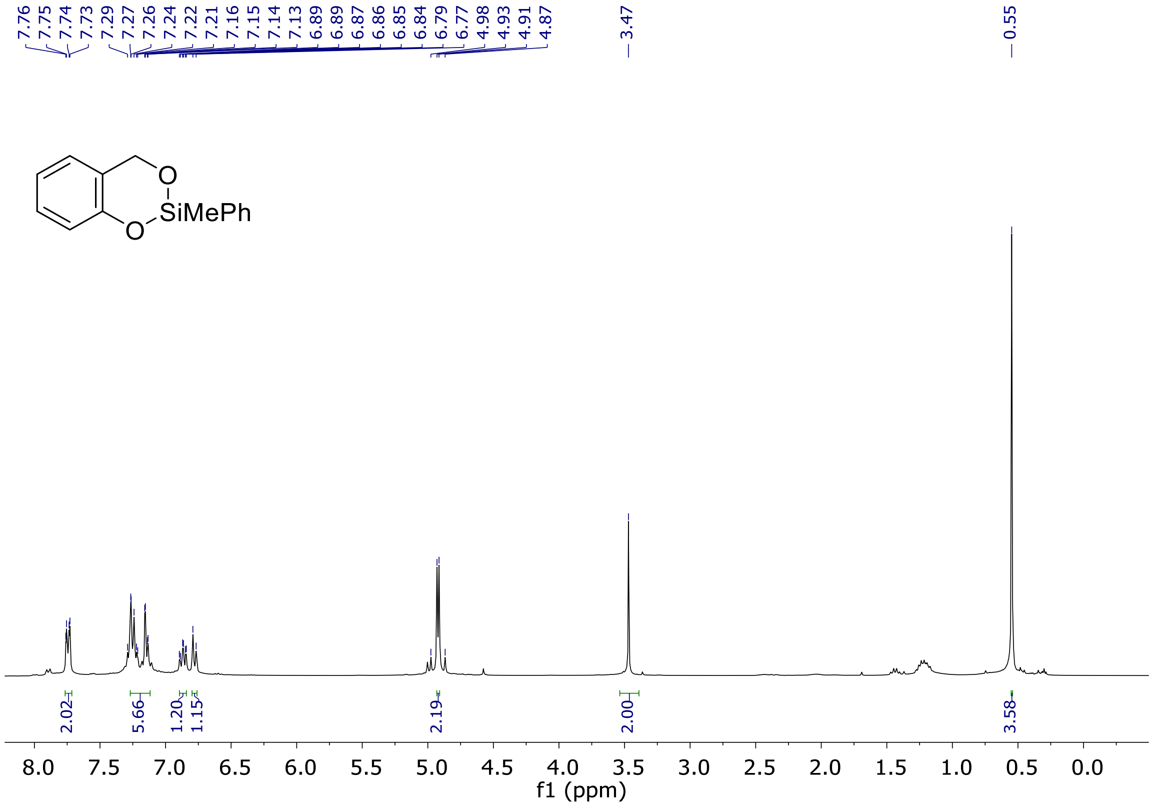

Figure S104. ^{1}H NMR spectrum (300 MHz, benzene-d_6, 298 K) of the crude reaction mixture of the tandem hydrosilylation/dehydrogenative silylation of salicylaldehyde with $H_2SiMePh$. Dioxane (δ 3.47 ppm; internal standard).

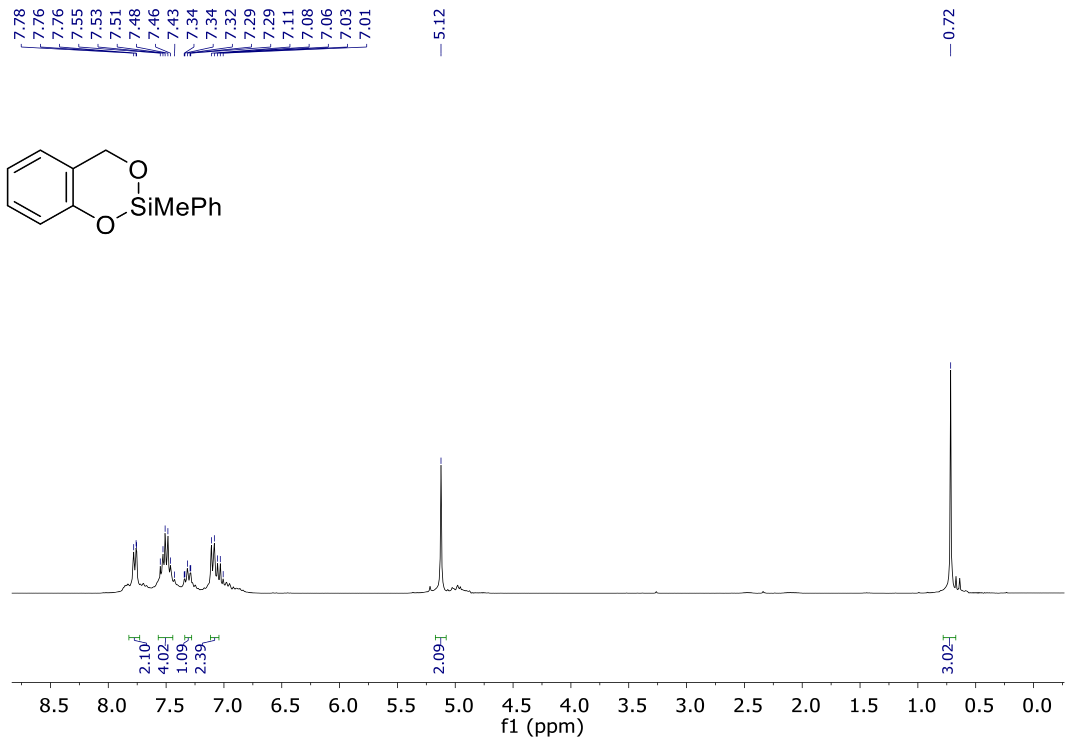

Figure S105. ^{1}H NMR spectrum (300 MHz, $CDCl_3$, 298 K) of 2-methyl-2-phenyl-4*H*-1,3,2-benzodioxasilin.

• References

(1) Alli, I. V.; Oñate, E.; Oliván, M. Polyhydride-Osmium-Mediated Hydrosilylation of Carbonyl Compounds: Intermediates and Mechanism. *Organometallics* **2025**, *44*, 2749-2762.

(2) Esteruelas, M. A.; Oliván, M.; Vélez, A. POP-Pincer Silyl Complexes of Group 9: Rhodium versus Iridium. *Inorg. Chem.* **2013**, *52*, 12108-12119.

(3) Yang, J.; Tilley, T. D. Efficient Hydrosilylation of Carbonyl Compounds with the Simple Amide Catalyst [Fe{N(SiMe$_3$)$_2$}$_2$]. *Angew. Chem. Int. Ed.* **2010**, *49*, 10186-10188.

(4) Nesbit, M. A.; Suess, D. L.; Peters, J. C. E–H bond activations and hydrosilylation catalysis with iron and cobalt metalloboranes. *Organometallics* **2015**, *34*, 4741-4752.

(5) Albright, A.; Gawley, R. E. Application of a C2-Symmetric Copper Carbenoid in the Enantioselective Hydrosilylation of Dialkyl and Aryl-Alkyl Ketones. *J. Am. Chem. Soc.* **2011**, *133*, 19680-19683.

(6) This compound has been previously reported. In an early report, the ^{1}H NMR resonances in CCl_4 of the SiH (δ 5.37 ppm) and SiOCH (δ 3.66 ppm) protons were given. See: Ojima; I.; Kogure, T.; Kumagai, M; Horiuchi, S.; Sato, T. Reduction of carbonyl compounds via hydrosilylation. 2. Asymmetric reduction of ketones via hydrosilylation catalyzed by a Rhodium(I) complex with chiral phosphine ligands. *J. Organomet. Chem.* **1976**, *122*, 83-97. In a more recent publication where this compound is prepared no NMR data were given. See: Tan, M.; Zhang, Y.; Ying, J. Y. Hydrosilylation of Ketone and Imine over Poly-N-Heterocyclic Carbene Particles. *Adv. Synth. Catal.* **2009**, *351*, 1390-1394.

(7) Szafoni, E.; Kuciński, K.; Hreczycho, G. Cobalt-catalyzed synthesis of silyl ethers via cross-dehydrogenative coupling between alcohols and hydrosilanes. *Green Chem. Lett. Rev.* **2022**, *15*, 757-764.

(8) Hosseinpour, Z.; Bockfeld, D.; Frank; R.; Tamm, M. Zinc Hydride Complexes Stabilized by Anionic *N*-Heterocyclic Carbenes for Hydrosilylation ofAldehydes and Ketones. *Adv. Synth. Catal.* **2026**, *368*, e70208.

(9) Mukherjee, D.; Thompson, R. R.; Ellern, A.; Sadow, A. D. Coordinatively Saturated Tris(oxazolinyl)borato Zinc Hydride-Catalyzed Cross Dehydrocoupling of Silanes and Alcohols. *ACS Catal.* **2011**, *1*, 698-702.

(10) Weißhuhn, J.; Mark, T.; Martin, M.; Müller, P.; Seifert, A.; Spange, S. Ternary organic–inorganic nanostructured hybrid materials by simultaneous twin polymerization. *Polym. Chem.* **2016**, *7*, 5060-5068.

(11) Blessing, R. H. *Acta Crystallogr.* **1995**, *A51*, 33. SADABS: Area-detector absorption correction; Bruker- AXS, Madison, WI, 1996.

(12) SHELXL-2019/6. Sheldrick, G. M. *Acta Cryst.* **2008**, *A64*, 112-122.

(13) (a) Lee, C.; Yang, W.; Parr, R. G. Development of the Colle-Salvetti correlationenergy formula into a functional of the electron density. *Phys. Rev. B* **1988**, *37*, 785-789. (b) Becke, A. D. Density-functional exchange-energy approximation with correct asymptotic behavior. *J. Chem. Phys.* **1993**, *98*, 5648-5652. (c) Stephens, P. J.; Devlin, F. J.; Chabalowski, C. F.; Frisch, M. J. Ab Initio Calculation of Vibrational Absorption and Circular Dichroism Spectra Using Density Functional Force Fields. *J. Phys. Chem.* **1994**, *98*, 11623-11627.

(14) Grimme, S.; Antony, J.; Ehrlich, S.; Krieg, H. A consistent and accurate ab initio parametrization of density functional dispersion correction (DFT-D) for the 94 elements H-Pu. *J. Chem. Phys.* **2010**, *132*, 154104.

(15) Gaussian 09, Revision D.01, Frisch, M. J.; Trucks, G. W.; Schlegel H. B.; Scuseria, G. E.; Robb, M. A.; Cheeseman, J. R.; Scalmani, G.; Barone, V.; Mennucci, B.; Petersson, G. A.; Nakatsuji, H.; Caricato, M.; Li, X.; Hratchian, H. P.; Izmaylov, A. F.; Bloino, J.; Zheng, G.; Sonnenberg, J. L.; Hada, M.; Ehara, M.; Toyota, K.; Fukuda, R.; Hasegawa, J.; Ishida, M.; Nakajima, T.; Honda, Y.; Kitao, O.; Nakai, H.; Vreven, T.; Montgomery, J. A.; Peralta, Jr., J. E.; Ogliaro, F.; Bearpark, M.; Heyd, J. J.; Brothers, E.; Kudin, K. N.; Staroverov, V. N.; Keith, T.; Kobayashi, R.; Normand, J.; Raghavachari, K.; Rendell, A.; Burant, J. C.; Iyengar, S. S.; Tomasi, J.; Cossi, M.; Rega, N.; S43 Millam, J. M.; Klene, M.; Knox, J. E.; Cross, J. B.; Bakken, V.; Adamo, C.; Jaramillo, J.; Gomperts, R.; Stratmann, R. E.; Yazyev, O.; Austin, A. J.; Cammi, R.; Pomelli, C.; Ochterski, J. W.; Martin, R. L.; Morokuma, K.; Zakrzewski, V. G.; Voth, G. A.; Salvador, P.; Dannenberg, J. J.; Dapprich, S.; Daniels, A. D.; Farkas, O.; Foresman, J. B.; Ortiz, J. V.; Cioslowski, J.; Fox, D. J. Gaussian, Inc., Wallingford CT, 2013.

(16) Andrea, D.; Häußermann, U. M.; Dolg, M.; Stoll, H.; Preuss, H. Energy adjusted ab initio pseudopotentials for the second and third row transition elements. *Theor. Chim. Acta* **1990**, *77*, 123-141.

(17) Ehlers, A. W.; Bohme, M.; Dapprich, S.; Gobbi, A.; Hollwarth, A.; Jonas, V.; Kohler, K. F.; Stegmann, R.; Veldkamp, A.; Frenking, G. A set of f-polarization functions for pseudo-potential basis sets of the transition metals SC-Cu, Y-Ag and La-Au. *Chem. Phys. Lett.* **1993**, *208*, 111-114.

(18) (a) Hehre, W. J.; Ditchfield, R.; Pople, J. A. Self-Consistent Molecular Orbital Methods. XII. Further Extensions of Gaussian-Type Basis Sets for Use in Molecular Orbital Studies of Organic Molecules. *J. Chem. Phys.* **1972**, *56*, 2257-2261. (b) Francl, M. M.; Pietro, W. J.; Hehre, W. J.; Binkley, J. S.; Gordon, M. S.; DeFrees, D. J.; Pople,

J. A. Self-consistent molecular orbital methods. XXIII. A polarization-type basis set for second-row elements. *J. Chem. Phys.* **1982**, *77*, 3654-3665.

(19) AIMAII (Version 19.10.12), Todd A. Keith, TK Gristmill Software, Overland Park KS, USA, 2019.

(20) NBO 7.0. Glendening, E. D.; Badenhoop, J, K.; Reed, A. E.; Carpenter, J. E.; Bohmann, J. A.; Morales, C. M.; Karafiloglou, P.; Landis, C. R.; and Weinhold, F. Theoretical Chemistry Institute, University of Wisconsin, Madison, USA, 2018

(21) (a) Becke, A. D. Density-functional exchange-energy approximation with correct asymptotic behavior. *Phys. Rev. A* **1988**, *38*, 3098-3100. (b) Perdew, J. P. Density-functional approximation for the correlation energy of the inhomogeneous electron gas. *Phys. Rev. B* **1986**, *33*, 8822-8824.

(22) Weigend F., Ahlrichs, F. R. Phys. Balanced Basis Sets of Split Valence, Triple Zeta Valence and Quadruple Zeta Valence Quality for H to Rn: Design and Assessment of Accuracy. *Chem. Chem. Phys.* **2005**, *7*, 3297-3305.
